# Self-Resetting Bistable Redox Molecular Machines for Fullerene Recognition

**DOI:** 10.1021/acs.orglett.2c01856

**Published:** 2022-07-29

**Authors:** Adriana Sacristán-Martín, Daniel Miguel, Héctor Barbero, Celedonio M. Álvarez

**Affiliations:** GIR MIOMeT, IU CINQUIMA/Química Inorgánica, Facultad de Ciencias, Universidad de Valladolid, Valladolid E47011, Spain

## Abstract

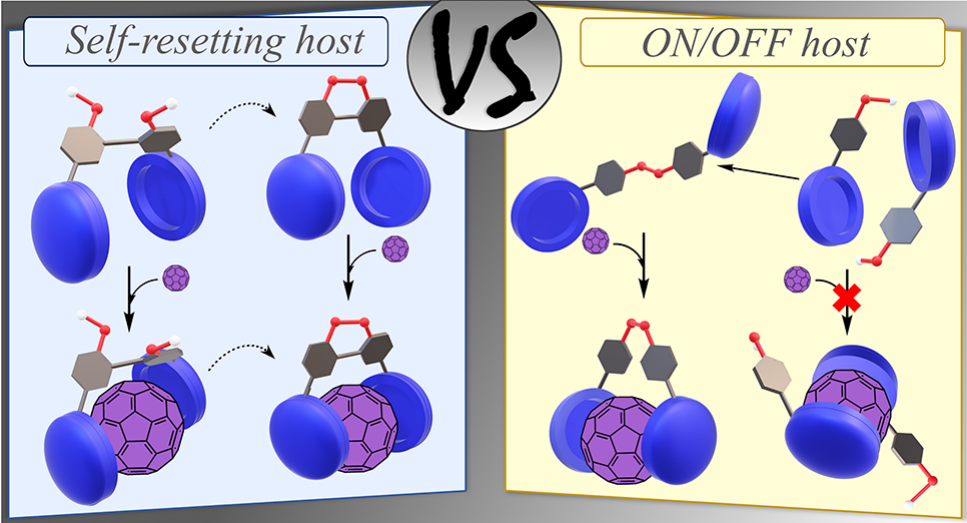

Addressing control over molecular machines resulting in variable output modulation by mimicking nature mechanisms is a current hot topic. The exploitation of reversibility in thiol/disulfide motifs in chemical systems flanked by nonplanar corannulene moieties capable to recognize fullerenes is presented herein. Two redox-based machines have been conceived for this purpose: an ON/OFF switch that activates its binding properties upon dimerization and a self-resetting (i.e., with an automated backward process) host that substantially modulates its affinity.

The rise of artificial machines that operate at the atomic level has brought to us a flourishment of models and concepts unimaginable two decades ago.^1,2^ Defined as multistable chemical systems that undergo relative movement of their constituent parts as a response to an external stimulus to perform observable work, we have been witnesses of the surge of multiple applications in a variety of fields.^3−5^ The source of the majority of concepts comes from nature, and therefore, it is not surprising that the most sophisticated operational principles are bioinspired.^6,7^

Spurred by our initial efforts into recently developed bistable systems (i.e., molecular machines that influence the system as a function of the state with an ON/OFF mechanism) in the form of switchable molecular tweezers bearing a recognition motif (corannulene) whose affinity toward fullerenes is photonically^8^ or chemically^9^ controlled, we decided to exploit a different chemical effector distinct from metal complexation such a redox event. The model we propose herein, again driven by nature’s inspirational influence, is based on the formation and cleavage of disulfide (S–S) bridges.^10^ It is well-known that this motif is present in a vast assortment of biomolecules, especially in proteins, whose function is to provide stability to the molecule favoring the maturation (correct folding) progress toward their native state.^10,11^ The variety of protocols to include the S–S motif, either chemically or electrochemically, in synthetic compounds is widespread^12,13^ and has been successfully applied in a variety of outstanding concepts.^14,15^ Corannulene chalcogenides,^16^ especially those bearing sulfur as the distinctive heteroatom, have been extensively studied due to their special electronic features when compared to the parent buckybowl, leading to materials with distinctive properties.^17−20^ One of the most interesting characteristics is their electron donation ability to form stable adducts with fullerenes.^21−23^

With our goal in mind, we focused our attention on the synthetic simplicity of *p*-corannulyl-thioanisole (**4**) and the ease of accessing its oxidized versions.^24^ We envisioned the formation of the corresponding thiophenol (**5-SH**) by deprotection to test the possibility of carrying out in situ redox switching and fullerene receptor abilities upon dimerization. Moreover, the inclusion of an additional restriction in a biphenyl fragment bearing two −SH groups to explore the intramolecular redox behavior as well as the impact on conformational landscape was considered. The principle of this proof of concept is depicted in Scheme 1a. Monomer **5-SH**, despite being slightly electron-rich, would not be able to bind fullerenes by itself unless it was highly adorned with donor arms,^21−23^ or linked to another corannulene group to cooperatively work.^25−27^ Once dimerized after oxidation, the new S–S bond restriction would bring two corannulene units together, furnishing fullerene recognition abilities for host **5**_**2**_**-SS**, albeit weak due to the large rotational freedom of such a structure (Scheme 1a). Conversely, the scenario could change in biphenyl-2,2′-dithiol **13-SH**, whose additional C–C bond restriction limits the conformational freedom around that bond rotation. A dihedral angle (θ) between 70 and 90° would be expected, with a torsional barrier that is high enough to limit the conformational population to a few geometries at room temperature. This would probably lead to an enhanced fullerene binding owing to a lower deformation energy penalty. Whenever oxidized to the corresponding disulfide **13-SS**, the second restriction would totally block the conformational freedom around the C–C bond, yielding a well-defined host for fullerene in a tweezer-like structure (θ ≈ 30°), locating both corannulene groups so that a preformed cavity is present and a better recognition would be expected (Scheme 1b).

**Scheme 1:**
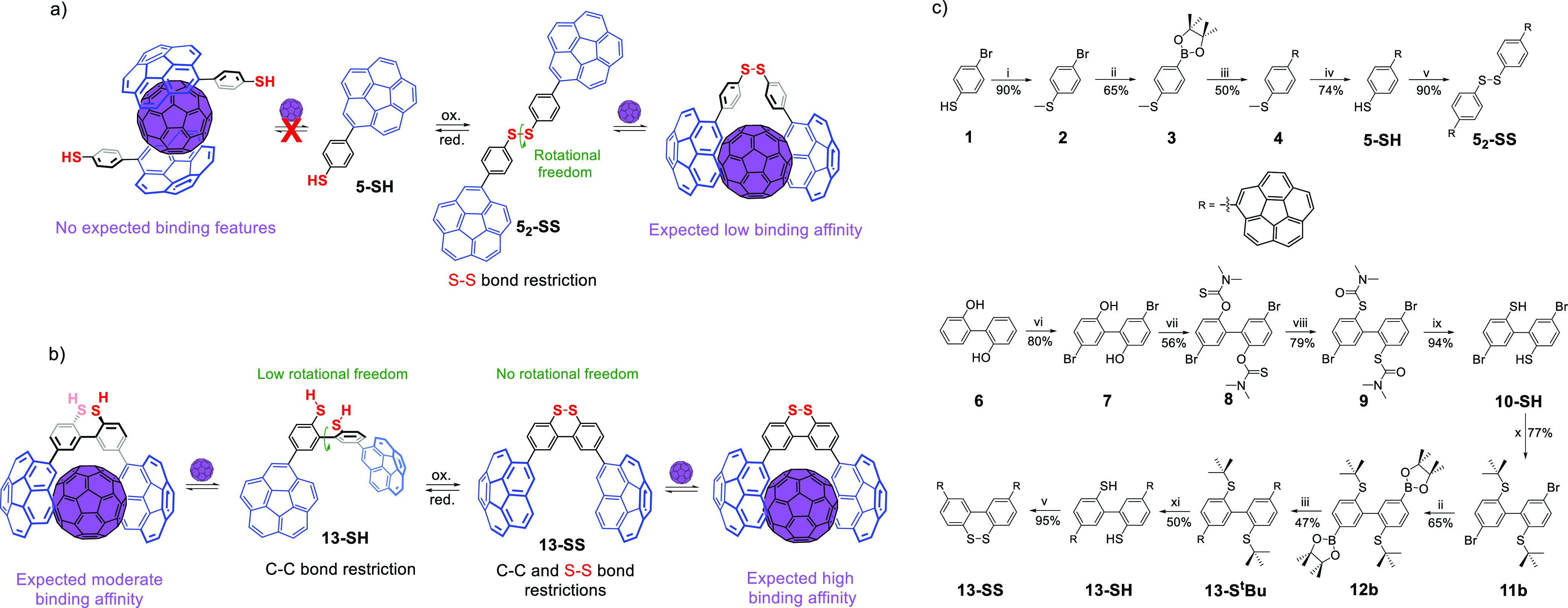
Proof of Concept for Redox Switchable Hosts for Fullerenes Based on (a) Dimerization or (b) Conformational Mobility Restriction; (c) Synthetic Route toward Target Compounds Reagents and conditions: (i) MeI, Na_2_CO_3_, CH_3_CN; (ii) B_2_(pin)_2_, [PdCl_2_(dppf)], KOAc, dioxane, MW, 170 °C; (iii) Br-Cora, [PdCl_2_(dppf)], ^t^BuONa, toluene, MW, 130 °C; (iv) ^t^BuSNa, DMF, 160 °C; (v) I_2_, NEt_3_, CH_2_Cl_2_; (vi) Br_2_, CH_2_Cl_2_; (vii) NaH, ClCSNMe_2_, DMF, 105 °C; (viii) NMP, 280 °C; (ix) LiAlH_4_, THF, reflux; (x) ^t^BuCl, AlBr_3_, CH_2_Cl_2_; (xi) 2-nitrobenzenesulfenyl chloride, THF, AcOH.

Compound **5-SH** was achieved by deprotection of the thiol group from intermediate **4**, which was prepared as described by Stuparu and co-workers.^24^ Simple oxidation with iodine provided dimer **5**_**2**_**-SS** almost quantitatively.^28^ Biphenyls **13-SH** and **13-SS** were obtained from 2,2′-biphenol (**6**), which, after a 4-step procedure, provided intermediate **10-SH** to which corannulene groups were attached via the corresponding ^t^butyl thioeter furnishing compound **13-S**^**t**^**Bu**, which, after the corresponding deprotection and oxidation, resulted in expected hosts **13-SH** and **13-SS**, respectively (Scheme 1c). Methyl thioether was also utilized as in compound **4** route, but it turned out to be unsuccessful.^28^ Nonetheless, alkylarylthioethers were also subjected to supramolecular association analyses to complement studied molecular machines.

All new synthesized compounds were fully characterized in solution by usual spectroscopic techniques as well as in solid state for some intermediates (Figures S1–S158).^28^ All species feature, in toluene, absorption bands in the violet region with maxima around 300 nm corresponding to π–π* transitions, as expected for this kind of compounds.^17,18,29^ Emission bands are structureless ranging from 432 to 438 nm with low to moderate quantum yields (up to 40%). Lifetimes decay monoexponentially within a range from 6.46 to 8.87 ns.^28^ More interestingly, CV measurements allowed us to get a deeper understanding about the redox behavior of these hosts. Anodic scan provides one-electron irreversible oxidation potentials (E^*a*^) of the corresponding thiolates leading to radical species Ar–S^•^ between −0.62 and −0.47 V, which immediately dimerize giving rise to Ar–SS–Ar species.^30^ This result confirms that a mild oxidant (such as I_2_) in basic medium is enough to play the role of the chemical effector. Likewise, the cathodic scan of disulfide compounds yields two-electron irreversible reduction potentials (*E*^*c*^), furnishing the corresponding thiolate species Ar–S^–^,^30^ above −1.70 V, suggesting that a strong reductant would be necessary for the molecular machine, albeit harsh conditions would not be required. As an example, CV plots for the couple **13-SH**/**13-SS** are provided in Figure 1.^28^ Moreover, most of the species containing corannulene show a low to moderate anodic shift on their first reduction potential (*E*^1^) when compared to corannulene under the same conditions.^24^

**Figure 1:**
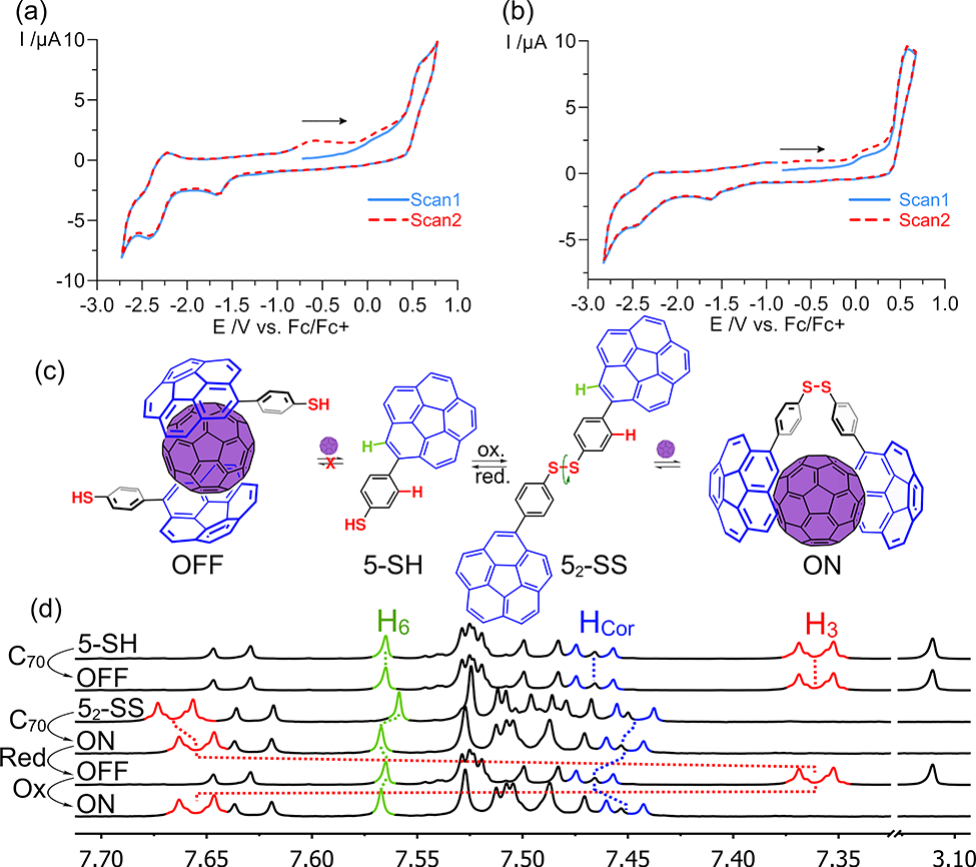
Cyclic voltammogram of hosts (a) **13-SH** and (b) **13-SS** in deaerated DMF at a concentration of 1.0 mM containing a solution of NBu_4_PF_6_ (0.10 M). Scan rate of 100 mV s^–1^. Potentials are referenced against Fc/Fc^+^. (c) ON/OFF behavior of molecular machine **5-SH**/**5**_**2**_**-SS**. (d) Example of ^1^H NMR spectral changes of the redox process and the supramolecular binding to C_70_ in toluene-d_8_ at 298 K.^31^ Red signal corresponds to phenylene protons, whereas green and blue signals belong to corannulene moieties.

Once we harnessed the electrochemical behavior of reported molecules, we then sought out the recognition capabilities of the **5-SH**/**5**_**2**_**-SS** couple. As expected, compound **5-SH** is unable to undergo fullerene recognition by itself (example in Figure 1c)^21−23^ as evidenced by the unchanged ^1^H NMR spectrum in toluene-d_8_ at 298 K after the addition of excess (10 equiv) of C_60_ (or C_70_) (Figures S159 and S160). The possibility of binding through self-aggregation was neglected as well (Figure S163). Conversely, compound **5**_**2**_**-SS** displays a distinct behavior. After the addition of excess (10 equiv) of the guest, several proton chemical shifts changed, indicating that the fullerene recognition event is taking place. Generally, phenylene tether protons experiment an upfield shift (Figure 1d, red signals), whereas corannulene moieties protons are shifted downfield (Figure 1d, green and blue signals). As predicted, dimer **5**_**2**_**-SS**, despite being poorly preorganized, is yet capable to adapt the structure to establish an attractive interaction with fullerenes. It is therefore possible to say the system is in the ON state. In situ reduction to monomer **5-SH** with LiAlH_4_ provides its original spectral feature, showing again no affinity for fullerenes, thus concluding that the system is now in the OFF state. Oxidation with I_2_ recovers inclusion complex **C**_**60**_**@5**_**2**_**-SS** whose ^1^H NMR spectrum is identical to the previous one, completing the thermodynamic cycle.^28,31^ These results clearly show that the pair **5-SH**/**5**_**2**_**-SS** behaves as a redox-based bistable ON/OFF molecular machine whose fullerene recognition capabilities are present only upon dimerization. Additionally, supramolecular association constants were determined by ^1^H NMR titration experiments in toluene-d_8_ at 298 K^28^ (Figures S164–S167) furnishing values of 1.87 ± 0.03 × 10^2^ M^–1^ and 7.24 ± 0.17 × 10^2^ M^–1^ for C_60_ and C_70_, respectively, according to a 1:1 stoichiometry. Despite the lack of restriction for the molecular tweezer **5**_**2**_**-SS** (i.e., with a poorly preorganized cavity), it yet shows a moderate affinity toward fullerenes and a slight preference for C_70_ over C_60_. We then focused our attention to the **13-SH**/**13-SS** couple. Structurally, the difference with respect to the previous species is the presence of a restriction in the form of a C–C bond linking both aryl units. This provides (1) a built-in tweezer-like structure with partial preorganization of a cavity between two corannulene groups and (2) the possibility to attach or release an additional restriction in the form of a S–S bond with the appropriate redox effectors. Hence, the difference in binding affinity would come from the accessibility of suitable conformations for fullerene recognition in the reduced form (**13-SH**) as the oxidized species (**13-SS**) would not allow torsions around the C–C bond. In situ switching between both oxidation states was also achieved (Figures S161 and S162). As expected, both states of the machine showed positive interaction with fullerenes. Titration experiments returned values of association constants for host **13-SH** of 1.90 ± 0.02 × 10^3^ M^–1^ and 2.03 ± 0.01 × 10^3^ M^–1^ for C_60_ and C_70_, respectively, whereas they were 3.93 ± 0.04 × 10^2^ M^–1^ and 4.65 ± 0.07 × 10^2^ M^–1^ for host **13-SS** (Figures S176–S183). Contrary to the initial assumptions, the behavior has been reversed as host **13-SH**, clearly less preorganized than host **13-SS**, showed near one order of magnitude of higher affinity (almost 4 kJ mol^–1^ difference on average). This effect might be the result of a limited, yet favorable, rotational landscape for compound **13-SH**, absent in its oxidized form, allowing it to achieve a better adaptation for fullerene recognition. Moreover, alkylthioether intermediates (**13-SMe** and **13-S**^**t**^**Bu**) showed similar behavior to that for **13-SH** (Figures S168–S175).

To shed light to this feature, we carried out DFT computational calculations^28^ to explore the potential energy surface (PES) in toluene of compound **13-SH** around the C–C bond connecting both aryl moieties. A wide range of conformations with θ between 47 and 143° lie below 20 kJ mol^–1^ (Figure S189), allowing the possibility to effortlessly access a synclinal conformation that furnishes a suitable cavity to host a fullerene molecule. Alkylthioether intermediates display a similar behavior albeit with a narrower set of torsion angles. Inclusion complexes **C**_**60**_**@13-SH** and **C**_**60**_**@13-SS** geometries were optimized, and their modeled structures are shown in Figure 2c. The former showed a θ of 88.3°, very close to the ideal right angle minimizing repulsion between tweezer ortho hydrogens and fullerene moiety. Corannulene bowls have an optimum distance of 12.4 Å between pentagon centroids. A similar rationale can be done for alkylthioethers **13-SMe** and **13-S**^**t**^**Bu**.^28^ The latter inclusion complex (**C**_**60**_**@13-SS**), however, given the additional restriction imposed by the disulfide link, is incapable to adapt the host as it only reaches a θ of 42.1°, resulting in a nonoptimum preorganized cavity with a shorter corannulene distance (12.1 Å). These differences might be responsible for experimental binding affinities since a great enthalpy or entropy variation (including possible desolvation penalty) is not expected due to their similarity in structure. Furthermore, natural energy decomposition analysis (NEDA)^28^ corroborates it by showing a substantial drop in interaction energy (*E*_int_ of 6.3 kJ mol^–1^). Computed molecular adduct **C**_**60**_**@5**_**2**_**-SS** has a similar arrangement to that of **C**_**60**_**@13-SH** (Figure 2c), although with a longer corannulenes distance (13.0 Å). Its lower association constant is then probably due to a deformation energy penalty, absent in all the other hosts, as commented above. Qualitative depiction of non-covalent interaction (NCI) plots suggests these observations as well.^28^

**Figure 2:**
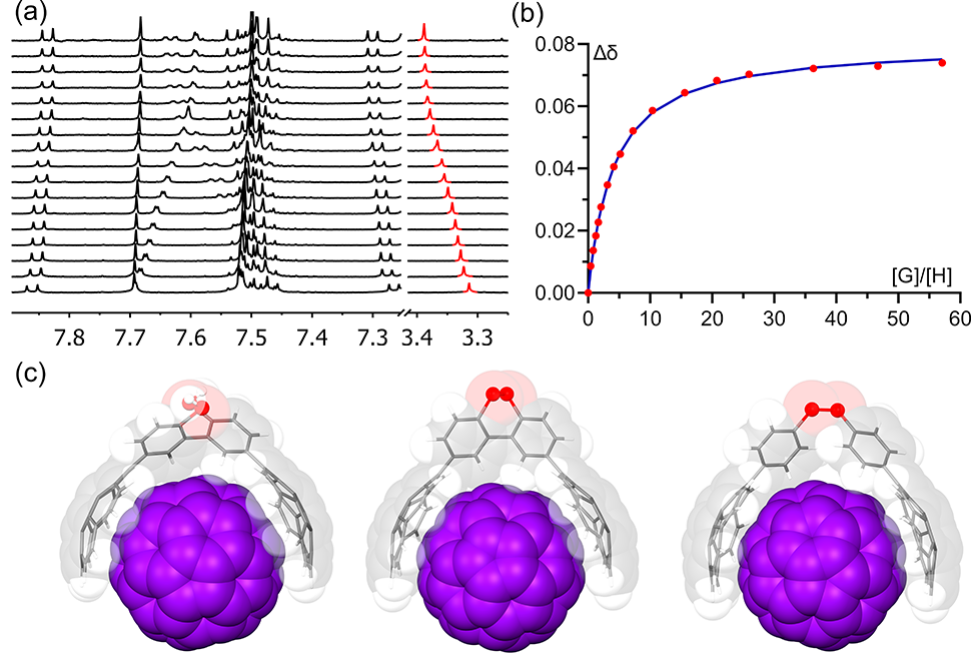
(a) Zoomed region of the ^1^H NMR spectra for the supramolecular titration of **13-SH** with C_60_ in toluene-d_8_ at 298 K. (b) Plot of the changes in the chemical shift (Δδ) of selected proton (in red) against molar fraction of the guest (C_60_). Blue line corresponds to the nonlinear fitting of Δδ to a 1:1 binding isotherm. (c) DFT-optimized geometry of assemblies **C**_**60**_**@13-SH** (left), **C**_**60**_**@13-SS** (center), and **C**_**60**_**@5**_**2**_**-SS** (right).

Compound **13-SH** shows an additional interesting feature. When such a species is left to stand for several hours in an open-air environment, atmospheric O_2_ gradually oxidizes it, returning host **13-SS**. This observation implies that the couple **13-SH**/**13-SS** has a dual behavior that depends on the absence or presence of molecular oxygen. In the first case, it behaves as a “regular” bistable switching machine, whereas in the second case, it becomes a self-resetting machine as it can go back in the **13-SH** → **13-SS** direction when switched to the opposite by an external stimulus (Figure 3a). Additionally, it has been found that the presence of a fullerene accelerates, by a factor of 2, the kinetics of the process as measured constant rises from 5.9 × 10^–3^ h^–1^ to 1.2 × 10^–2^ h^–1^ in host **13-SH** and complex **C**_**70**_**@13-SH**, respectively, considering a first-order kinetics (Figure 3b and 3c).^28^ We tentatively propose that the formation of the supramolecular adduct fixes the conformation of host **13-SH**, allowing the close proximity between both sulfur atoms and favoring the formation of the disulfide bond.^32−34,35^ Furthermore, this behavior was not observed for monomer **5-SH** as there is no host–guest complexation.

**Figure 3:**
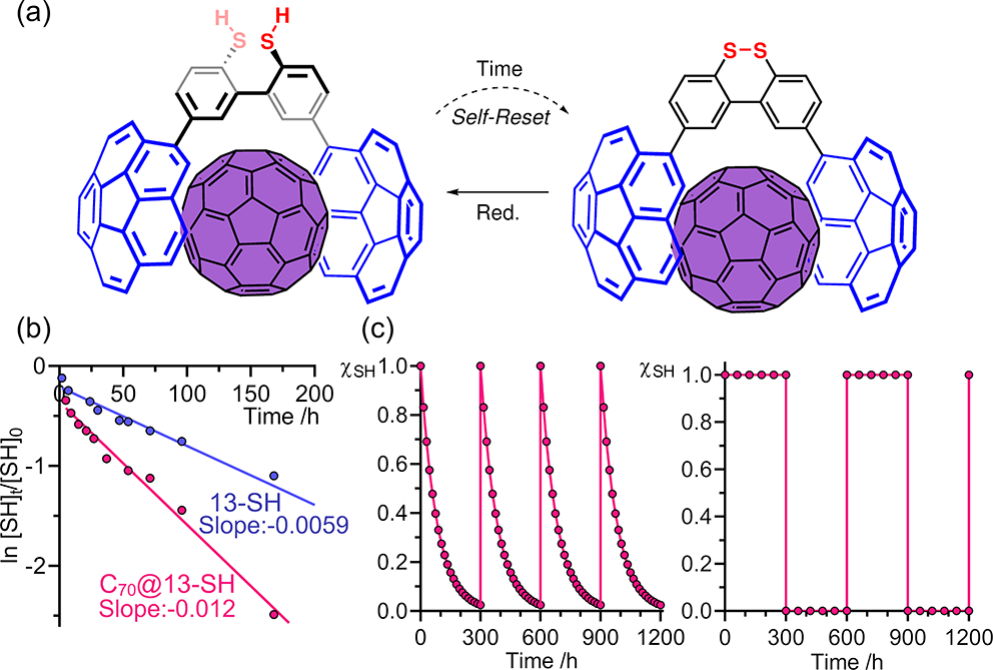
(a) Self-resetting behavior of molecular machine **13-SH**/**13-SS**. (b) Linear fitting plot of oxidation rate constant of compound **13-SH** in the presence (pink) or absence (blue) of C_70_. (c) Comparison of the molar fraction variation of compound **13-SH** throughout different self-resetting (left) or conventional (right) redox cycles.

In summary, two redox-based molecular machines capable of modulating their affinity toward fullerenes by means of disulfide bond formation/cleavage have been developed and studied. A bistable ON/OFF host capable of “activating” its binding properties upon dimerization is presented along with a molecular machine with dual behavior (ON/OFF vs self-resetting) that modulates its supramolecular affinity owing to the conformational restrictions imposed by each state.

## Supporting Information Available

Detailed synthetic procedures, full characterization by 1D and 2D NMR along with HR-MS spectra, UV–vis and emission experiments details including decay lifetimes and quantum yields, cyclic voltammetry experiments, single-crystal X-ray diffraction analysis, dilution experiments, in situ and ex situ reduction/oxidation cyclic process description, association constant measurements, kinetic experiments, computational calculation details (PDF)
