# Supplementary material for: Self-Resetting Bistable Redox Molecular Machines for Fullerene Recognition
Source: Org Lett. 2022 Jul 29;24(32):5879–83. doi: 10.1021/acs.orglett.2c01856 (PMC9400385; doi:10.1021/acs.orglett.2c01856)
Supplement: Supplementary file 1 — ol2c01856_si_001.pdf [file ol2c01856_si_001.pdf]

Supporting Information

Self-resetting bistable redox molecular machines for fullerene recognition

Adriana Sacristán-Martín, Daniel Miguel, Héctor Barbero* and Celedonio M. Álvarez*

GIR MIOMeT, IU CINQUIMA/Química Inorgánica, Facultad de Ciencias, Universidad de Valladolid, Valladolid, E47011, Spain.

Table of Contents

Experimental Procedures 2
General methods 2
Synthesis overview 3
Synthetic procedures and characterization details 5
NMR and HR-MS spectra 13
UV-Vis absorption and emission spectra 88
Fluorescence decay lifetimes and Quantum Yields 90
Cyclic Voltammograms 91
X-ray structures and Crystallographic Tables 95
In situ switching procedure 98
Dilution experiments 101
Association constants measurements 102
Kinetic Studies 114
Computational Calculation Details 117
Notes and References 138

Experimental Procedures

General methods

Reagents were purchased from regular suppliers and used without further purification. 1-Bromocorannulene (Br-cora) was acquired from Synoi Chemicals (http://synoichemicals.uva.es/). Solvents were of analytical grade or spectrophotometric grade. They were either used as purchased or dried according to procedures described elsewhere.[1-2] Microwave reactions were carried out with an Anton Paar Monowave 300 Reactor using tightly capped flasks G10 (for volumes up to 10 mL) especially designed for the apparatus. All reactions under inert atmosphere (when needed) were performed with standard Schlenk techniques. They were also used as a preliminary step for degassing microwave flasks when inert atmosphere was necessary in microwave reactions. Column chromatography separations were carried out by using Silica gel 60 (particle size 0.040-0.063 mm; 230-400 mesh; Merck, Germany) as the stationary phase and TLCs were performed on precoated silica gel plates (0.25 mm thick, 60 F254, Merck, Germany) and observed under UV light. Purifications by centrifugation were performed in an Ortoalresa UNICEN centrifuge. NMR spectra were recorded on Agilent DD2 500 and Agilent MR 400 instruments. ^{1}H and ^{13}C NMR chemical shifts are reported in parts per million (ppm) and are referenced to TMS, using residual solvent peak as an internal reference. Coupling constants (*J*) are reported in hertz (Hz). Standard abbreviations used to indicate multiplicity: s = singlet, d = doublet, m = multiplet, dd = doublet of doublets. ^{1}H and ^{13}C assignments were performed by utilizing 2D NMR methods (gCOSY, gDQFCOSY, band selective ROESY, NOESY, band selective HSQC, band selective HMBC, gradient crisis HSQC and gradient crisis HMBC). High resolution mass spectra were recorded at mass spectrometry service of the Laboratory of Instrumental Techniques of the University of Valladolid (L.T.I., www.laboratoriotecnicasinstrumentales.es). A MALDI-TOF system (MALDI-TOF) Bruker Autoflex Speed (N_2 laser (337 nm, pulse energy 100 μJ, 1 ns), acceleration voltage 19 kV, reflector positive mode) was used. Trans-2-[3-(4-tert-butylphenyl)-2-methyl-2-propenylidene]malonitrile (DCTB) and 1,8-dihydroxy-9(10H)-anthracenone (dithranol) were used as matrixes. A UPLC-MS system (UPLC: Waters ACQUITY H-class UPLC; MS: Bruker Maxis Impact) by electrospray ionization (ESI positive and negative) was utilized as well. HRMS spectra were analyzed using Bruker DataAnalysis 4.1© (www.bruker.com). Steady state UV/Vis absorption spectroscopy was carried out on a Perkin Elmer Lambda 265 spectrophotometer, whereas emission spectroscopy was performed on a Cary Eclipse Fluorescence, using quartz cuvettes with a path length of 1.0 cm in toluene as the solvent. Time-resolved fluorescence measurements were carried out using the single-photon counting technique with ns time resolution. A high repletion pulsed light source is used to excite the sample and the photons emitted are processed using the TCC1 card in the computer. Fluorescence decays were obtained with the Time Correlated Single Photon Counting (TCSPC) and MCP-PMT counter module (TCC2) of the FLS980 spectrometer (Edinburgh Instruments). The excitation source was diode laser with excitation wavelength 280 nm for all samples. EPLs-lasers produce picosecond duration pulses < 1ns at repetition rates up to 20 MHz (50ns). Emission slit used was 8 nm. Fluorescence decays were analyzed with the method of non-linear least squares iterative deconvolution and the quality of the fits was judged by the values of the reduced Chi-square (χ^2) and the autocorrelation function of the residuals using the FAST (Advanced Fluorescence Lifetime Analysis Software) program provided by the equipment. To measure the photoluminescence quantum yield (QY) the FLS980 fluorescence spectrometer is equipped with an integrating sphere. A rectangular 10 mm cuvette was used for the fluorescence measurements. All data were measured at 25 ºC. Cyclic voltammetry was carried out using a PalmSens4 potentiostat, with a 0.10 M solution of tetrabutylammonium hexafluorophosphate (NBu_4PF_6) as the supporting electrolyte in DMF as the solvent at a scan rate of 100mV/s in all the experiments. Solutions were deaerated with a nitrogen stream prior to each measurement. Experiments were performed in a one-compartment cell equipped with a glassy carbon electrode, a silver wire counter electrode, and an Ag/AgCl wire as pseudo-reference electrode. All potentials were referenced against the ferrocene/ferrocenium couple (Fc/Fc^+) after each experiment. Diffraction data were collected using an Oxford Diffraction Supernova diffractometer equipped with an Atlas CCD area detector and a four-circle κ goniometer. For the data collection, a Mo-microfocused source with multilayer optics were used. Data integration, scaling, and empirical absorption correction were performed using the CrysAlisPro software package and the structure was solved and refined with SHELX in OLEX2. Graphics were made with MERCURY.

Synthesis overview

The synthetic strategy to achieve target compounds relies on two similar set of steps, yet with significant differences.

On the one hand, to get the **5-SH/5₂-SS** couple, we followed a modified method, as reported by Stuparu[3] that starts from *p*-bromothiophenol (**1**) which, after being protected in the form of a thioanisole (**2**), it is involved in a Suzuki-Miyaura cross coupling with the appropriate borylated intermediate **3** that ultimately leads to target species **4**. The deprotection with sodium tertbutylthiolate results in monomer **5-SH** in 74% yield which can be practically quantitatively oxidized to dimer **5_2-SS** with a mild oxidant such as iodine, in basic medium (Scheme S 1).

On the other hand, the access to compounds **13-SH** and **13-SS** started with 2,2'-biphenol (**6**) which, after a 4-step procedure, provides intermediate **10-SH** in a decent overall yield of 42% (Scheme S 2). The latter is not appropriate for a subsequent Suzuki C-C cross coupling due to the catalyst poisoning properties of the substrate as it acts as a chelating ligand for Pd. In our aim to get access to the target compound in the lowest number of steps, we first oxidized compound **10-SH**, again with I_2 in basic medium, to obtain species **10-SS**, apparently amenable to corannulene grafting. However, neither borylation nor cross coupling occurred, most likely due to catalyst poisoning again. Therefore, intermediate **10-SH** was protected by methylation, mirroring what succeeded for the **5-SH/5_2-SS** couple, followed by a successful borylation process and, finally, coupled to give rise to compound **13-SMe** (Scheme S 3). Such a species did not furnish its expected deprotected version (**13-SH**) after deprotection by already described means[4] or with other strong reducing reagents, such as sodium naphthalene.[5] An intractable mixture was obtained in all cases, most likely due to metylthiolate group loss and/or corannulene reduction.[6] We then turned our attention to a bulkier alkyl group, such as tert-butyl, to protect thiol groups. Thus, the same synthetic sequence was subjected to intermediate **10-SH**, furnishing corannulene derivative **13-S^{t}Bu**, which was conveniently deprotected with 2-nitrobenzenesulfenyl chloride,[7-8] providing target host **13-SH** with a moderate, but sufficient yield of 50%. Its oxidized version (**13-SS**) can also be quantitatively obtained with iodine in basic medium (Scheme S 4).

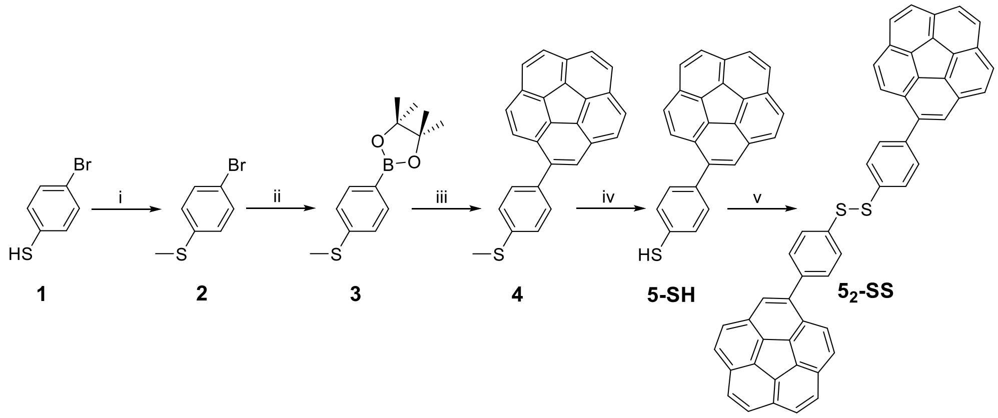

Scheme S 1. Synthesis route to achieve compounds **2**, **3**, **4**, **5-SH** and **5_2-SS**. Reagents and conditions: (i) MeI, Na_2CO_3, CH_3CN; (ii) $B_2(pin)_2$, [$PdCl_2$(dppf)], KOAc, dioxane, MW, 170 °C; (iii) Br-Cora, [$PdCl_2$(dppf)], ^{t}BuONa, toluene, MW, 130 °C; (iv) ^{t}BuSNa, DMF, 160 °C; (v) I_2, NEt_3, CH_2Cl_2.

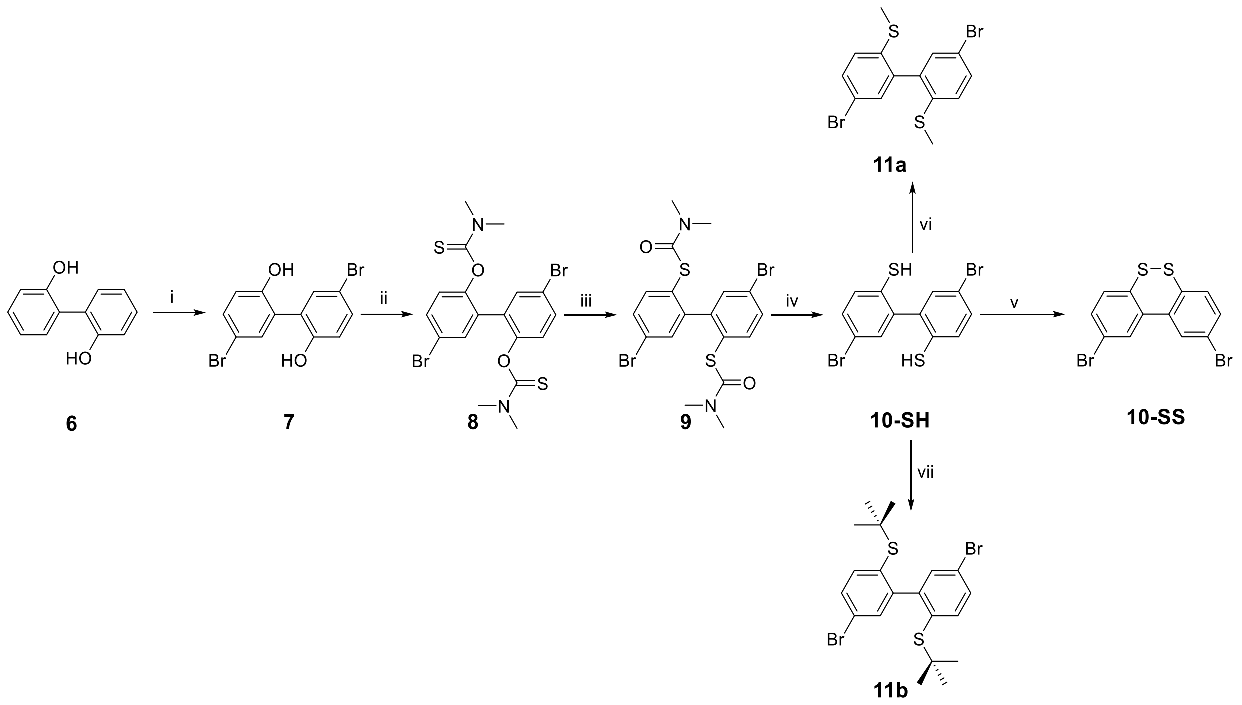

Scheme S 2. Synthesis route to achieve compounds **7**, **8**, **9**, **10-SH**, **10-SS**, **11a** and **11b**. Reagents and conditions: (i) Br_2, CH_2Cl_2; (ii) NaH, ClCSNMe$_2$, DMF, 105 °C; (iii) NMP, 280 °C; (iv) $LiAlH_4$, THF, reflux; (v) I_2, NEt_3, CH_2Cl_2; (vi) NaH, MeI, THF, CH_2Cl_2; (vii) ^{t}BuCl, $AlBr_3$, CH_2Cl_2.

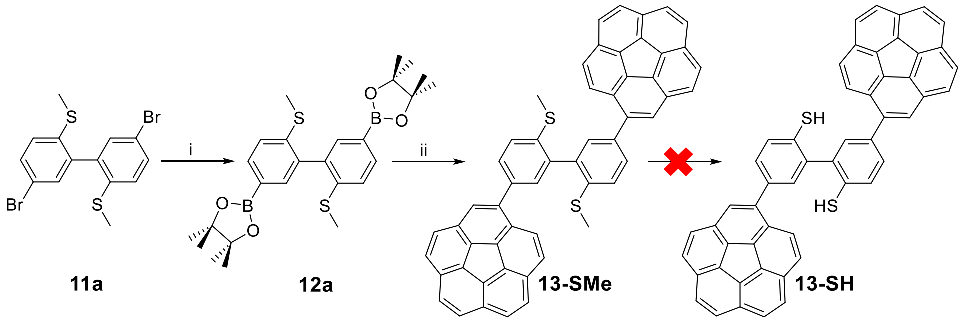

Scheme S 3. Synthesis route to achieve compounds **12a** and **13-SMe** and failed attempt to achieve compound 13-SH. Reagents and conditions: (i) $B_2(pin)_2$, [PdCl$_2$(dppf)], KOAc, dioxane, MW, 170 °C; (ii) Br-Cora, [PdCl$_2$(dppf)], ^{t}BuONa, toluene, MW, 130 °C.

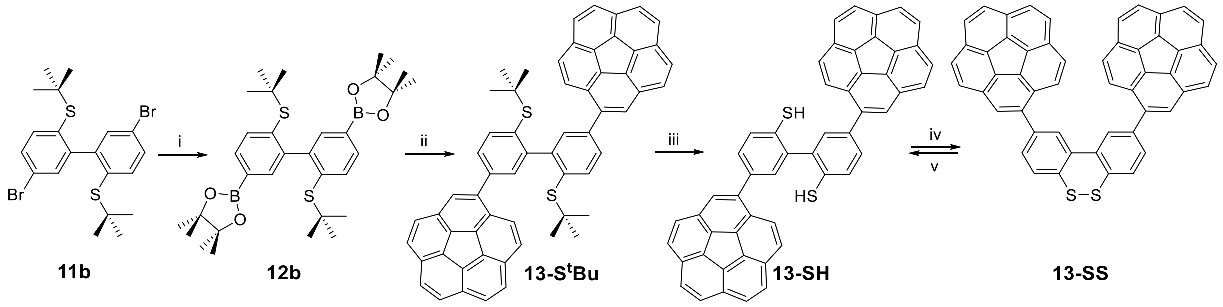

Scheme S 4. Synthesis route to achieve compounds **12b**, **13-S^{t}Bu**, **13-SH** and **13-SS**. Reagents and conditions: (i) $B_2(pin)_2$, [PdCl$_2$(dppf)], KOAc, dioxane, MW, 170 °C; (ii) Br-Cora, [PdCl$_2$(dppf)], ^{t}BuONa, toluene, MW, 130 °C; (iii) 2-Nitrobenzenesulfenyl chloride, THF, AcOH; (iv) I_2, NEt_3, CH_2Cl_2; (v) $NaBH_4$, THF, MeOH.

Synthetic procedures and characterization details

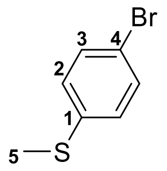

Compound 2

Compound **2** was prepared according to literature and spectral data were in agreement to those previously reported [9-10] with 90% yield. ^{1}H NMR (500 MHz, $CDCl_3$) δ 7.39 (d, *J* = 8.7 Hz, 2H, H), 7.12 (d, *J* = 8.6 Hz, 2H, H), 2.47 (s, 3H, H_5).^{13}C NMR (101 MHz, $CDCl_3$) δ 137.8 (C_1), 131.9 (C_3), 128.3 (C_2), 118.8 (C_4), 16.1 (C_5).

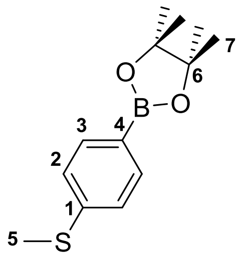

Compound 3

Compound **2** (0.20 g, 1.0 mmol), bis(pinacolato)diboron (0.25 g, 1.5 mmol), [$PdCl_2$(dppf)] (37 mg, 50 μmol) and potassium acetate (0.29 g, 3.0 mmol) were mixed in a sealed microwave flask under inert atmosphere. Dry dioxane (6.0 mL) was added and the mixture was degassed. It was then irradiated in a microwave reactor at 170 °C for 30 min with stirring at 600 rpm. Solvent was removed under vacuum before a purification by column chromatography on silica gel (20:1 n-hexane/AcOEt) to give the expected compound as a white solid (0.16 mg, 65% yield). Spectral data were in agreement with those previously reported.[11] ^{1}H NMR (500 MHz, $CDCl_3$) δ 7.70 (d, *J* = 8.3 Hz, 2H, H_3), 7.22 (d, *J* = 8.3 Hz, 2H, H_2), 2.49 (s, 3H, H_5), 1.34 (s, 12H, H_7). ^{13}C NMR (126 MHz, $CDCl_3$) δ 142.7 (C_1), 135.2 (C_3), 125.2 (C_2, C_4), 83.9 (C_6), 25.0 (C_7), 15.2 (C_5). HRMS (ESI-TOF): *m/z* = 251.1280 $[M+H]^+$ calculated 251.1274 for $C_{13}H_{20}BO_2S$).

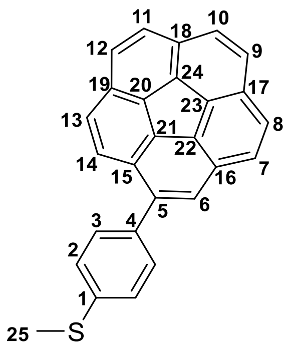

Compound 4

Compound **3** (29 mg, 0.11 mmol), 1-bromocorannulene (41 mg, 0.12 mmol), [$PdCl_2$(dppf)] (11 mg, 15 μmol), and ^{t}BuONa (35 mg, 0.36 mmol) were mixed in a microwave flask under inert atmosphere. Dry and degassed toluene (3.0 mL) was then added. The solution was irradiated in a sealed microwave reactor at 130 °C for 30 minutes with stirring at 600 rpm. The solvent was removed under vacuum before subjecting the resulting crude to a purification by column chromatography on silica gel (20:1 n-hexane/AcOEt) to give the pure compound as a pale-yellow solid (21 mg, 50% yield). Spectral data were in agreement with those reported in the literature.[3] ^{1}H NMR (500 MHz, $CDCl_3$) δ 7.86 (s, 1H, H_6), 7.85 (m, 2H, H_7,H_8), 7.84 – 7.79 (m, 5H, H_{10},H_{11},H_9,H14,H_{12}), 7.78 (d, *J* = 8.8 Hz, 1H, H_{13}), 7.72 (d, *J* = 8.4 Hz, 2H, H_3), 7.44 (d, *J* = 8.4 Hz, 2H, H_2), 2.59 (s, 3H, H_{25}).^{13}C NMR (126 MHz, $CDCl_3$) δ 141.3 (C_5), 138.4 (C_1), 136.6 (C_4), 136.5 (C_{21}), 136.4 (C_{24}), 136.0 (C_{20}), 135.6 (C_{23}), 135.5 (C_{22}), 131.1 (C_q), 131.0 (C_q),131.0 (C_q), 130.9 (C_q), 130.5 (C_3), 129.8 (C_{15}), 127.6 (C_{13}), 127.5 (C_8), 127.4 (C_{11}), 127.3 (C_9), 127.2 (C_{12}), 127.1 (C_{10}), 127.1 (C_7), 127.1 (C_{14}), 126.8 (C_2), 125.7 (C_6), 16.0 (C_{25}). ^{1}H NMR (500 MHz, Toluene-d_8) δ 7.71 (d, J = 8.8 Hz, 1H, H_{14}), 7.63 (s, 1H, H_6), 7.55 – 7.53 (m, 3H, H_7, H_{11}, H_{12}), 7.53 – 7.50 (m, 2H, H_8, H_{10} or H_9), 7.50 – 7.47 (m, 4H, H_3, H_{13} ,H_9 or H_{10}), 7.21 (d, J = 8.4 Hz, 2H, H_2), 2.10 (s, 3H, H_{25}). ^{13}C NMR (101 MHz, toluene) δ 141.6 (C_5), 139.0 (C_1), 136.9 (C_{21}), 136.8 (C_4), 136.7 (C_q), 136.3 (C_q), 135.9 (C_q), 135.8 (C_{22}), 131.3 (C_q), 131.3 (C_q,C_{19}), 131.1 (C_q), 130.7 (C_3), 130.1 (C_q), 127.6 (C_{10} or C_9), 127.5 (C_{11} or C_{12}), 127.4 (C_8), 127.4 (C_{14}), 127.3 (C_{HCora}), 127.3

(C_{HCora}), 127.2 (C_7), 127.1 (C_{13},C_{HCora}), 126.9 (C_2), 125.8 (C_6), 15.2 (C_{25}). HRMS (MALDI-TOF): *m/z* = 372.0952 $[M]^+$ calculated 372.0967 for $C_{27}H_{16}S$).

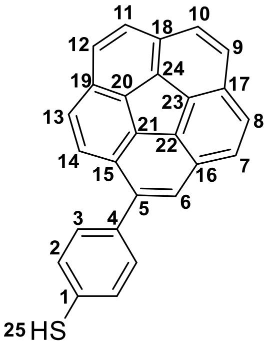

Compound 5-SH

A mixture of **4** (45 mg, 0.12 mmol), ^{t}BuSNa[4] (0.11 g, 0.97 mmol) in dry DMF (0.50 mL) was heated in an oil bath under inert atmosphere at 160 °C for 1 hour. The reaction color turned red and another portion of ^{t}BuSNa (0.11 g, 0.98 mmol) was added to the mixture. After 1 h, the reaction was allowed to cool to room temperature. HCl (35%) (2.0 mL) was added to the mixture to quench the reaction and a pale-yellow solid appeared. The solid was separated by centrifugation. Subsequently, it was dissolved in CH_2Cl_2 and extracted with H_2O (3 X 10 mL). It was dried with $MgSO_4$, filtered, and concentrated at reduced pressure until 1.0 mL of CH_2Cl_2 was left. Addition of 3.0 mL of n-hexane was needed to afford precipitation. Such a resulting solid was then centrifuged to obtain the pure expected compound as a pale-yellow solid (32 mg, 74% yield). ^{1}H NMR (500 MHz, $CDCl_3$) δ 7.84 (s, 1H, H_6), 7.86 – 7.75 (m, 8H, H_7, H_8, H_9, H_{10}, H_{11}, H_{12}, H_{13}, H_{14}), 7.67 (d, J = 8.5 Hz, 2H, H_3), 7.46 (d, J = 8.5 Hz, 2H, H_2), 3.58 (s, 1H, H_{25}). ^{13}C NMR (126 MHz, $CDCl_3$) δ 140.9 (C_5), 137.2 (C_4), 136.3 (C_q), 136.2 (C_q), 135.8 (C_q), 135.4 (C_q), 135.3 (C_q), 131.0 (C_q), 130.9 (C_q), 130.8 (C_q), 130.8 (C_q), 130.6 (C_3), 130.5 (C_1), 129.6 (C_2), 129.5 (C_{15}), 127.5 (C_{HCora}), 127.4 (C_{HCora}), 127.3 (C_{HCora}), 127.1 (C_{HCora}), 127.0 (C_{HCora}), 126.9 (C_{HCora}), 126.9 (C_{HCora}), 126.8 (C_{HCora}), 125.6 (C_6). HRMS (ESI-TOF): *m/z* = 359.0890 $[M+H]^+$ calculated 359.0889 for $C_{26}H_{15}S$).

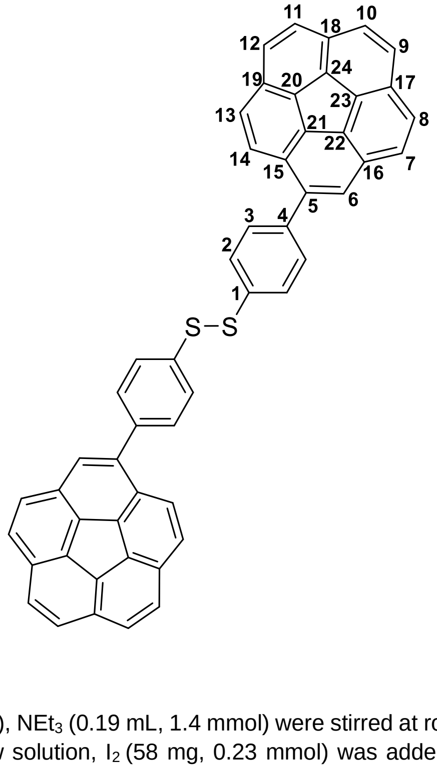

Compound 5_2-SS

A mixture of **5-SH** (25 mg, 70 μmol), NEt_3 (0.19 mL, 1.4 mmol) were stirred at room temperature in CH_2Cl_2 (10 mL) for 5 minutes. To the bright-yellow solution, I_2 (58 mg, 0.23 mmol) was added, and the solution color turned to orange. It was stirred until there was no presence of starting material in the mixture, according to the ^{1}H-NMR spectrum of an aliquot, and the reaction was quenched by addition of a solution of $Na_2S_2O_3$ (sat) (3mL). The crude was extracted with H_2O (3 x 10 mL), dried with $MgSO_4$, filtered, and concentrated under pressure. Final purification was carried out by column chromatography on silica gel (15:1 n-hexane/AcOEt) to give the pure compound as a yellow solid (22 mg, 90% yield). ^{1}H NMR (500 MHz, $CDCl_3$) δ 7.88 (s, 2H, H_6), 7.86 – 7.75 (m, 24H, H_2, H_3, H_7, H_8, H_9, H_{10}, H_{11}, H_{12}, H_{13}, H_{14}). ^{13}C NMR (126 MHz, $CDCl_3$) δ 140.9 (C_q), 139.0 (C_q), 136.8 (C_q), 136.5 (C_q), 136.4 (C_q), 136.0 (C_q), 135.6 (C_q), 135.6 (C_q), 131.2 (C_q), 131.1 (C_q), 131.0 (C_q), 130.9 (C_q), 130.8 (C_{2or3}), 129.6 (C_q), 128.0

(C_{2or3}), 127.7 (C_{HCora}), 127.6 (C_{HCora}), 127.5 (C_{HCora}), 127.3 (C_{HCora}), 127.2 (C_{HCora}), 127.1 (C_{HCora}), 127.1 (C_{HCora}), 127.0 (C_{HCora}), 126.1 (C_6). HRMS (MALDI-TOF) *m/z* = 714.1481 $[M]^+$ calculated 714.1470 for $C_{52}H_{26}S_2$).

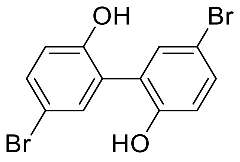

Compound 7

4,4'-Dibromo-2,2'-biphenol was synthesized by following the reported literature procedure from compound **6**.[12] The spectral data were in agreement with those previously reported as well[13] with 80% yield.

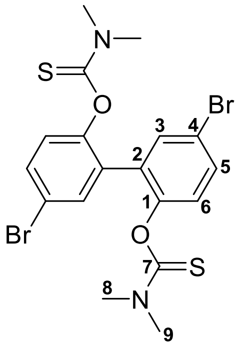

Compound 8

Compound **7** (4.0 g, 12 mmol) in DMF (15 mL) was kept in a Schlenk under inert atmosphere. NaH (1.1 g, 47 mmol) was added portion-wise for 15 min. After completing the addition, the mixture was left to stir at room temperature. After 30 min, dimethylcarbamoyl chloride (4.3 g, 35 mmol) was added and the mixture was stirred overnight at 105 °C in an oil bath under inert atmosphere. Then the reaction was allowed to cool down to room temperature and water (20 mL) was added in order to quench the reaction. The reaction was extracted with CH_2Cl_2 (3 x 20 mL), dried with $MgSO_4$, filtered and evaporated to give a brownish oil. Compound **8** was purified by column chromatography on silica gel (n-hexane/ CH_2Cl_2 1:1) to give the expected molecule as a white solid. (3.3 g, 56% yield). ^{1}H NMR (500 MHz, $CDCl_3$) δ 7.57 (d, J = 2.4 Hz, 2H, H_3), 7.51 (dd, J = 8.7, 2.4 Hz, 2H, H_5), 7.09 (d, J = 8.7 Hz, 2H, H_6), 3.31 (s, 6H, H_9), 3.08 (s, 6H, H_8).^{13}C NMR (126 MHz, $CDCl_3$) δ 186.7 (C_7), 150.5 (C_2), 133.8 (C_3), 132.0. (C_5), 131.4 (C_1), 126.4 (C_6), 118.6 (C_4), 43.4 (C_9), 38.7 (C_8). HRMS (ESI-TOF): *m/z* = 538.9078 $[M+Na]^+$ calculated 538.9069 for $C_{18}H_{18}Br_2N_2NaO_2S_2$).

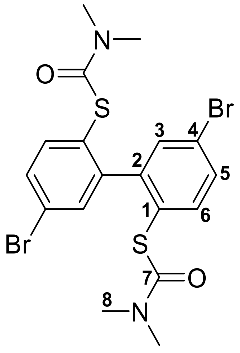

Compound 9

Compound **8** (0.52g, 1.0 mmol), NMP (2.0 mL) were mixed in a microwave flask giving a light-yellow solution. It was irradiated in a sealed microwave reactor at 280 °C for 15 min with stirring at 600 rpm. After that time, water (10 mL) was added to the red solution and a white solid precipitated. It was separated by centrifugation and washed with water until the filtrate became colorless. The white solid was then dissolved in CH_2Cl_2 (20 mL) and extracted with water (3 x 20 mL), dried with $MgSO_4$, filtered and concentrated under vacuum to dryness. A mixture of CH_2Cl_2 (2.0 mL) and n-hexane (8.0 mL) was needed to afford precipitation of a pure white solid. (0.41g, 79% yield). ^{1}H NMR (500 MHz, $CDCl_3$) δ 7.52 (dd, J = 8.3, 2.2 Hz, 2H, H_5), 7.46 (d, J = 8.3 Hz, 2H, H_6), 7.43 (d, J = 2.2 Hz, 2H, H_3), 2.90 (s, 12H, H_8). ^{13}C NMR (101 MHz, $CDCl_3$) δ 165.7 (C_7), 146.0 (C_2), 138.5 (C_6), 133.4 (C_3), 131.7 (C_5), 128.0 (C_1), 123.4 (C_4), 37.1 (C_8). HRMS (ESI-TOF): *m/z* = 538.9086 $[M+Na]^+$ calculated 538.9069 for $C_{18}H_{18}Br_2N_2NaO_2S_2$).

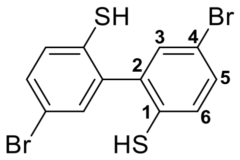

Compound 10-SH

Compound **9** (1.0 g, 2.0 mmol) was dissolved in dry THF (12 mL) under inert atmosphere. $LiAlH_4$ in THF (13 mL, 1.0 M) was added dropwise. The reaction was refluxed using an oil bath under inert atmosphere. After 1 h, it was quenched with slow addition of water followed by addition of 2.0 mL of HCl (35%) and extracted with diethyl ether (3 x 20 mL), dried with $MgSO_4$, filtered and the solvent was evaporated under vacuum to obtain a yellow oil of the pure compound which eventually solidified (0.70 g, 94% yield). ^1H NMR (500 MHz, $CDCl_3$) δ 7.41 (dd, J = 8.4, 2.1 Hz, 2H, H_5), 7.31 (d, J = 2.1 Hz, 2H, H_3), 7.28 (d, J = 8.4 Hz, 172H, H_6), 3.28 (s, 2H, SH). ^{13}C NMR (126 MHz, $CDCl_3$) δ 139.7 (C_2), 133.0 (C_3), 132.2 (C_5), 131.2 (C_6), 131.1 (C_4), 119.4 (C_1). HRMS (ESI-TOF): *m/z* = 372.8357 $[M-H]^-$ calculated 372.8361 for $C_{12}H_7Br_2S_2$).

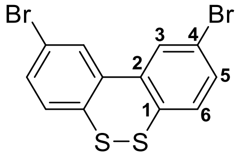

Compound 10-SS

Compound **10-SS** was synthesized following the same procedure as compound **5₂-SS**. It was isolated as a yellow solid. (90% yield). ^1H NMR (500 MHz, $CDCl_3$) δ 7.79 (d, J = 2.1 Hz, 2H, H_3), 7.44 (dd, J = 8.2, 2.1 Hz, 2H, H_5), 7.39 (d, J = 8.2 Hz, 2H, H_6). ^{13}C NMR (101 MHz, $CDCl_3$) δ 138.8 (C_2), 135.4 (C_1), 131.4 (C_5), 130.9 (C_3), 130.4 (C_6), 122.6 (C_4). HRMS (ESI-TOF): *m/z* = 370.8197 $[M-H]^-$ calculated 370.8205 for $C_{12}H_5Br_2S_2$).

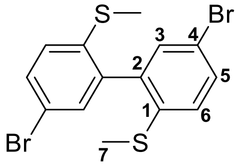

Compound 11a

Compound **10-SH** (0.70 g, 1.8 mmol) was dissolved in dry THF (18 mL) and NaH (0.18g, 7.3 mmol) was added. After stirring for 10 min, MeI (0.45 mL, 7.3 mmol) and CH_2Cl_2 (5.0 mL) were added to the mixture and it was allowed to stir at room temperature for 30 min. Water (10 mL) was added to quench the reaction and it was extracted with CH_2Cl_2 (3 x 20 mL), dried with $MgSO_4$, filtered and the solvent was evaporated under vacuum to obtain the pure compound as a yellowish solid (0.67 g, 92% yield). ^1H NMR (500 MHz, $CDCl_3$) δ 7.49 (dd, J = 8.5, 2.2 Hz, 2H, H_5), 7.27 (d, J = 2.2 Hz, 2H, H_3), 7.14 (d, J = 8.5 Hz, 2H, H_6), 2.38 (s, 6H, H_7). ^{13}C NMR (101 MHz, $CDCl_3$) δ 139.2 (C_2), 137.7 (C_1), 132.7 (C_3), 131.9 (C_5), 126.9 (C_6), 118.3 (C_4), 16.0 (C_7). HRMS (ESI-TOF): *m/z* = 400.8661 $[M-H]^-$ calculated 400.8674 for $C_{14}H_{11}Br_2S_2$).

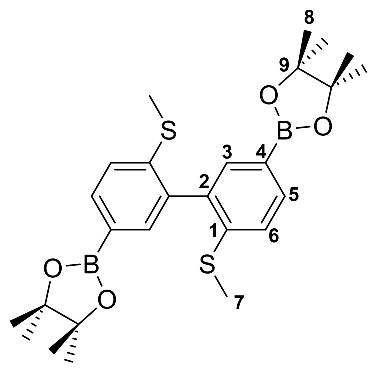

Compound 12a

Compound **11a** (0.10 g, 0.25 mmol), bis(pinacolato)diboron (0.19 g, 0.75 mmol), [PdCl$_2$(dppf)] (18 mg, 25 μmol) and potassium acetate (0.15g, 1.5 mmol) were mixed in a sealed microwave flask under inert atmosphere. Dry dioxane (2.0 mL) was added and the mixture was degassed. It was then irradiated in a microwave reactor at 170 °C for 30 min with stirring at 600 rpm. Solvent was removed under vacuum before a purification by column chromatography on silica gel (10:1 n-hexane/AcOEt) to give the expected compound as a white solid (75 mg, 60% yield). ^{1}H NMR (500 MHz, CDCl$_3$) δ 7.78 (dd, *J* = 8.0, 1.4 Hz, 2H, H$_5$), 7.54 (d, *J* = 1.4 Hz, 2H, H$_3$), 7.24 (d, *J* = 8.0 Hz, 2H, H$_6$), 2.40 (s, 6H, H$_7$), 1.32 (s, 24H, H$_8$). ^{13}C NMR (101 MHz, CDCl$_3$) δ 142.5 (C$_1$), 137.9 (C$_2$), 136.4 (C$_3$), 134.9 (C$_5$), 124.5 (C$_4$), 123.4 (C$_6$), 83.8 (C$_9$), 25.1 (C$_8$), 25.0 (C$_8$), 15.5 (C$_7$). HRMS (ESI-TOF): *m/z* = 521.2155 [M+Na]$^+$ calculated 521.2143 for $C_{26}H_{36}Br_2NaO_4S_2$).

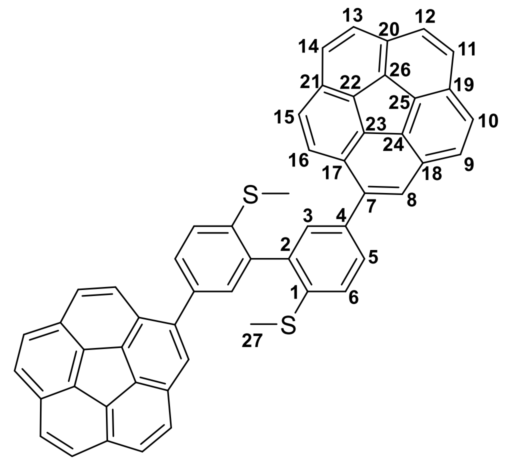

Compound 13-Me

Compound **12a** (40 mg, 80 μmol), 1-bromocorannulene (54 mg, 0.16 mmol), [PdCl$_2$(dppf)] (24 mg, 33 μmol), and ^{t}BuONa (47 mg, 0.49 mmol) were mixed in a microwave flask under inert atmosphere. Dry and degassed toluene (6.0 mL) was then added. The solution was irradiated in a sealed microwave reactor at 130 °C for 30 minutes with stirring at 600 rpm. The solvent was removed under vacuum before subjecting the resulting material to a purification by column chromatography on silica gel (5:1 n-hexane/AcOEt) to give the pure compound as a pale-yellow solid (30 mg, 50% yield). ^{1}H NMR (500 MHz, CDCl$_3$) δ 7.98 (d, *J* = 8.8 Hz, 2H, H$_{16}$), 7.95 (s, 2H, H$_8$), 7.85 – 7.81 (m, 14H, H$_5$, H$_9$, H$_{10}$, H$_{11}$, H$_{12}$, H$_{13}$, H$_{14}$), 7.81 – 7.78 (m, 4H, H$_3$, H$_{15}$), 7.52 (d, *J* = 8.2 Hz, 2H, H$_6$), 2.55 (s, 6H, H$_{27}$). ^{13}C NMR (126 MHz, CDCl$_3$) δ 141.1 (C$_7$), 138.8 (C$_2$), 138.2 (C$_1$), 136.4 (C$_{23}$), 136.2 (C$_q$), 136.0 (C$_4$), 135.6 (C$_{22}$), 135.5 (C$_q$), 135.4 (C$_{24}$) 132.0 (C$_3$), 131.1 (C$_q$), 131.0 (C$_q$), 130.9 (C$_q$), 130.1 (C$_5$), 129.8 (C$_{17}$), 127.6 (C$_{15}$), 127.5 (C$_{HCora}$), 127.4 (C$_{16}$), 127.4 (C$_{HCora}$), 127.2 (C$_{HCora}$), 127.1 (C$_{HCora}$, C$_9$), 127.1 (C$_{14}$), 125.7 (C$_8$), 125.4 (C$_6$), 15.9 (C$_{27}$). HRMS (ESI-TOF): *m/z* = 743.1847 [M+H]$^+$ calculated 743.1862 for $C_{54}H_{31}S_2$).

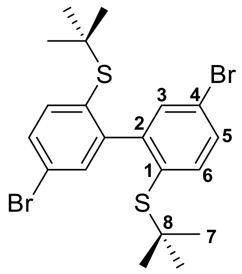

Compound 11b

Compound **10-SH** (0.15 g, 0.40 mmol), tert-butyl chloride (1.0 mL) and $AlBr_3$ (catalytic amount, 11 mg, 40 µmol) were mixed under inert atmosphere. Dry CH_2Cl_2 (1.0 mL) was added, and it was allowed to stir at room temperature for 30 min. the mixture was quenched with H_2O (2.0 mL) and after the addition of CH_2Cl_2 (10 mL), the mixture was extracted with water (3 x 10 mL), dried with $MgSO_4$, filtered and concentrated under vacuum giving a yellow oil. Final purification was carried out by column chromatography on silica gel (pure n-hexane to 10:1 n-hexane/AcOEt) to give the pure compound as a yellowish oil. (0.15 g, 77% yield). ^1H NMR (500 MHz, $CDCl_3$) δ 7.51 (d, *J* = 8.2 Hz, 2H, H_6), 7.47 (dd, *J* = 8.2, 2.2 Hz, 2H, H_5), 7.43 (d, *J* = 2.2 Hz, 1H, H_3), 1.06 (s, 18H, H_7). ^{13}C NMR (101 MHz, $CDCl_3$) δ 148.3 (C_2), 139.2 (C_6), 133.5 (C_3), 133.1 (C_1), 130.7 (C_5), 122.2 (C_4), 47.7 (C_8), 31.3 (C_7). HRMS (ESI-TOF): *m/z* = 508.9590 $[M+Na]^+$ calculated 508.9578 for $C_{20}H_{24}Br_2NaS_2$).

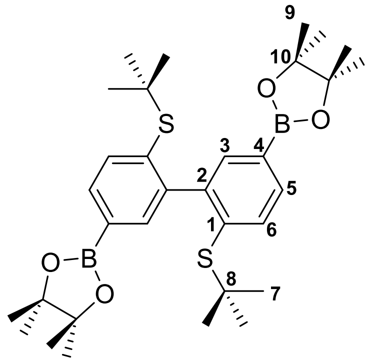

Compound 12b

Compound **11b** (0.34 g, 0.70 mmol), bis(pinacolato)diboron (0.53 g, 2.1 mmol), [$PdCl_2$(dppf)] (51 mg, 70 µmol) and potassium acetate (0.41g, 4.1 mmol) were mixed in a sealed microwave flask under inert atmosphere. Dry dioxane (3.5 mL) was added and the mixture was degassed. It was then irradiated in a microwave reactor at 170 °C for 30 min with stirring at 600 rpm. Solvent was removed under vacuum before a purification by column chromatography on silica gel (10:1 n-hexane/AcOEt) to give the expected compound as a white solid (0.21 g, 65% yield). ^1H NMR (500 MHz, $CDCl_3$) δ 7.74 (d, *J* = 1.4 Hz, 2H, H_3), 7.72 (dd, *J* = 7.7, 1.4 Hz, 2H, H_5), 7.64 (d, *J* = 7.7 Hz, 2H, H_6), 1.36 (s, 12H, H_9), 1.33 (s, 12H, H_9), 1.04 (s, 18H, H_7). ^{13}C NMR (126 MHz, $CDCl_3$) δ 146.8 (C_2), 137.4 (C_1), 137.2 (C_3), 136.6 (C_6), 133.3 (C_5), 84.0 (C_{10}), 47.5 (C_8), 31.4 (C_7), 25.2 (C_9), 24.8 (C_9). HRMS (MALDI-TOF): *m/z* = 605.3066 $[M+Na]^+$ calculated 605.3084 for $C_{32}H_{48}B_2NaO_4S_2$).

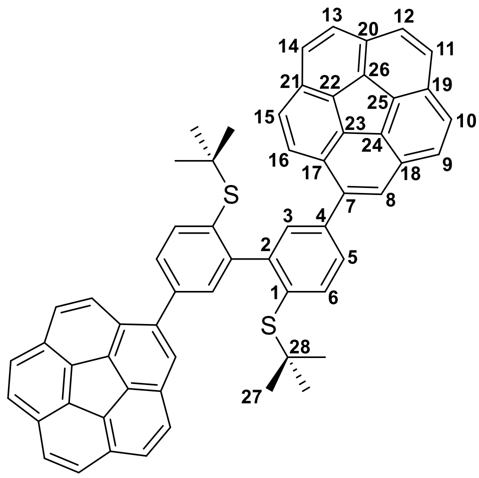

Compound 13-S^{t}Bu

Compound **12b** (90 mg, 0.15 mmol), 1-bromocorannulene (0.11g, 0.33 mmol), [PdCl$_2$(dppf)] (23 mg, 30 μmol), and ^{t}BuONa (90 mg, 0.93 mmol) were mixed in a sealed microwave flask under inert atmosphere. Dry and degassed toluene (7.5 mL) was then added. The solution was irradiated in a microwave reactor at 130 °C for 30 minutes with stirring at 600 rpm. The solvent was removed under vacuum before subjecting the resulting crude to a purification by column chromatography on silica gel (15:1 n-hexane/AcOEt) to give the pure compound as a pale-yellow solid (60 mg, 47% yield). ^{1}H NMR (500 MHz, CDCl$_3$) δ 7.91 (s, 2H, H$_8$), 7.95 (d, J = 7.9 Hz, 2H, H$_6$), 7.89 (d, J = 8.8 Hz, 2H, H$_{16}$), 7.85 – 7.78 (m, 16H, H$_3$, H$_9$, H$_{10}$, H$_{11}$, H$_{12}$, H$_{13}$, H$_{14}$, H$_5$), 7.78 (d, J = 8.8 Hz, 2H, H$_{15}$), 1.23 (s, 18H, H$_{27}$). ^{13}C NMR (101 MHz, CDCl$_3$) δ 147.8 (C$_2$), 140.9 (C$_7$), 139.3 (C$_4$), 137.9 (C$_6$), 136.3 (C$_{23}$), 136.2 (C$_{25or26}$), 135.8 (C$_{22}$), 135.4 (C$_{24}$), 135.4 (C$_{25or26}$), 133.4, (C$_1$) 132.4 (C$_3$), 131.0 (C$_q$), 130.8 (C$_{21}$), 130.8 (C$_q$), 130.7 (C$_q$), 129.5 (C$_{17}$), 128.7 (C$_5$), 127.5 (C$_{15}$), 127.4 (C$_{HCora}$), 127.3 (C$_{HCora}$), 127.1 (C$_{HCora}$), 127.0 (C$_{HCora}$), 126.9 (C$_9$), 126.9 (C$_{16}$), 126.9 (C$_{14}$), 126.0 (C$_8$), 47.5 (C$_{28}$), 31.5 (C$_{27}$). ^{1}H NMR (500 MHz, Toluene-d_8) δ 8.08 (d, J = 2.1 Hz, 2H, H$_3$), 7.96 (d, J = 8.8 Hz, 2H, H$_{16}$), 7.92 (d, J = 7.9 Hz, 1H, H$_6$), 7.78 (s, 2H, H$_8$), 7.62 (dd, J = 7.9, 2.1 Hz, 2H, H$_5$), 7.52 – 7.44 (m, 14H, H$_9$,H$_{10}$,H$_{11}$,H$_{12}$,H$_{13}$,H$_{14}$,H$_{15}$), 1.24 (s, 18H, H$_{27}$). ^{13}C NMR (126 MHz, Toluene-d_8) δ 148.4 (C$_2$), 141.3 (C$_7$), 139.9 (C$_4$), 138.4 (C$_6$), 136.8 (C$_q$), 136.7 (C$_q$), 136.3 (C$_q$), 135.9 (C$_{24}$), 135.9 (C$_q$), 134.3 (C$_1$), 133.2 (C$_3$), 131.3 (C$_q$), 131.3 (C$_q$), 131.2 (C$_q$), 131.2 (C$_q$), 129.9 (C$_{17}$), 129.0 (C$_5$), 127.7 (C$_{HCora}$), 127.5 (C$_{15}$), 127.4 (C$_{HCora}$), 127.3 (C$_{HCora}$), 127.2 (C$_9$,C$_{16}$) 127.1 (C$_{HCora}$), 126.4 (C$_8$), 47.2 (C$_{28}$), 31.6 (C$_{27}$). HRMS (ESI-TOF): m/z = 849.2618 [M+Na]$^+$ calculated 849.2620 for $C_{60}H_{42}NaS_2$).

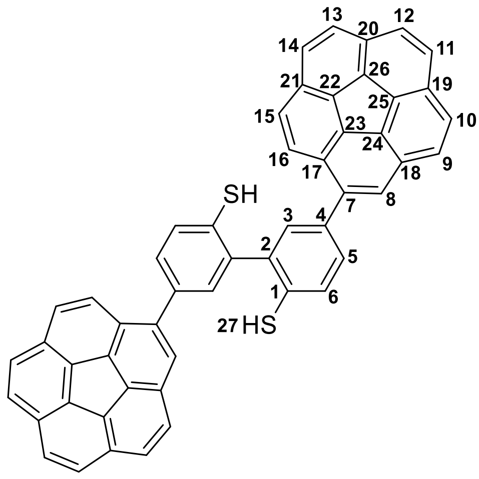

Compound 13-SH

Compound **13-S^{t}Bu** (60 mg, 70 μmol) was dissolved under inert atmosphere in a 1:1 mixture of THF/AcOH (4.0 mL). 2-Nitrobenzenesulfonyl chloride (39 mg, 0.17 mmol) was added and the mixture was allowed to stir at room temperature. After 15 hours, it was quenched with water and extracted with CH_2Cl_2 (3 x 10mL). It was dried with $MgSO_4$, filtered, concentrated under vacuum, and precipitated with hexane. A yellow powder appeared which was isolated by centrifugation. Subsequently, it was dissolved under inert atmosphere in a mixture of THF/MeOH (5mL, 3:2) giving a light yellow color solution. $NaBH_4$ (30 mg, 0.80 mmol) was added and the solution turned brownish in color. After 30 min the reaction was quenched with HCl (35%) (1.0 mL) and the solution turned yellow again. It was extracted with CH_2Cl_2/H_2O (3 x 10mL), dried with $MgSO_4$, filtered, concentrated under vacuum and precipitated with hexane. A light yellow powder appeared which was isolated by centrifugation. Final purification was carried

out by column chromatography on silica gel (3:1 to 1:1 n-hexane/AcOEt) to give the expected compound as a light yellowish solid. (25 mg, 50% yield). ^{1}H NMR (500 MHz, $CDCl_3$) δ 7.92 (s, 2H, H_8), 7.91 (d, J = 8.9 Hz, 2H, H_{16}), 7.84 – 7.81 (m, 10H, H_9, H_{10}, H_{11}, H_{12}, H_{13}, H_{14}), 7.80 (d, J = 2.1 Hz, 2H, H_3), 7.80 (m, 2H, H_{15}, H_{Cora}), 7.75 (dd, J = 8.1, 2.1 Hz, 2H, H_5), 7.66 – 7.62 (d, J = 8.1 Hz, 2H, H_6), 3.56 (s, 2H, H_{27}). ^{13}C NMR (126 MHz, $CDCl_3$) δ 140.6 (C_7), 139.3 (C_2), 137.6 (C_4), 136.6 (C_{23}), 136.4 (C_q), 136.0 (C_{22}), 135.6 (C_{24}), 135.5 (C_q), 132.0 (C_3), 131.8 (C_q), 131.2 (C_q), 131.1 (C_{21}), 131.0 (C_q), 130.9 (C_q), 130.4 (C_5), 130.1 (C_6), 129.6 (C_{17}), 127.8 (C_{15}), 127.6 (C_{HCora}), 127.5 (C_{HCora}), 127.3 (C_{HCora}), 127.2 (C_{HCora}), 127.1(C_{HCora}, C_9, C_{16}), 125.9 (C_8). HRMS (ESI-TOF): *m/z* = 715.1535 $[M+H]^+$ calculated 71715.1549 for $C_{52}H_{27}S_2$).

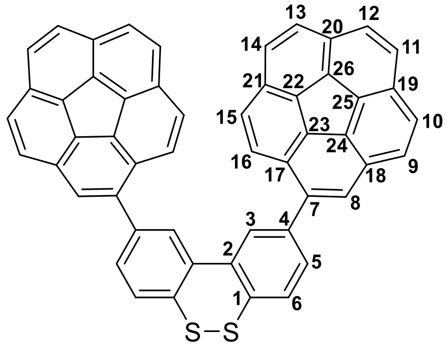

Compound 13-SS

A mixture of **13-SH** (20 mg, 20 μmol), NEt_3 (56 μL, 0.40 mmol) were stirred at room temperature in CH_2Cl_2 (10 mL) for 5 minutes. To the bright-yellow solution, I_2 (15 mg, 6.0 μmol) was added, and the solution color turned to orange. It was stirred until there was no presence of starting product in the mixture, according to the ^{1}H-NMR spectrum of an aliquot, and the reaction was quenched by addition of a solution of $Na_2S_2O_3$ (sat) (3.0 mL). The crude was extracted with H_2O (3 x 10 mL), dried with $MgSO_4$, filtered, and concentrated under pressure giving the pure compound as a light-yellow solid. (18 mg, 95% yield). ^{1}H NMR (500 MHz, $CDCl_3$) δ 8.15 (d, J = 1.4 Hz, 2H, H_3), 7.89 (s, 2H, H_8), 7.82 (d, J = 8.8 Hz, 2H, H_{16}), 7.80 – 7.78 (m, 10H, H_9,H_{10},H_{11},H_{12},H_{13}), 7.78 – 7.76 (m, 6H, H_{14},H_5,H_6), 7.74 (d, J = 8.8 Hz, 2H, H_{15}). ^{13}C NMR (126 MHz, $CDCl_3$) δ 140.6 (C_1), 140.5 (C_4), 138.5 (C_2), 136.5 (C_{23}), 136.7 (C_q), 136.0 (C_{22}), 135.9 (C_7), 135.6 (C_q), 135.5 (C_q), 131.15 (C_q), 131.07 (C_{21}), 131.0 (C_q), 130.8 (C_q), 129.7 (C_5), 129.6 (C_3), 129.55 (C_6), 129.4 (C_{17}), 127.8 (C_{15}), 127.6 (C_{HCora}), 127.5 (C_{10}), 127.3 (C_{HCora}), 127.3 (C_{HCora}), 127.1 (C_{HCora}), 127.1 (C_{HCora}), 126.8 (C_9), 126.2 (C_{14}). HRMS (MALDI): *m/z* = 712.1350 $[M]^+$ calculated 712.1314 for $C_{52}H_{24}S_2$).

NMR and HR-MS spectra

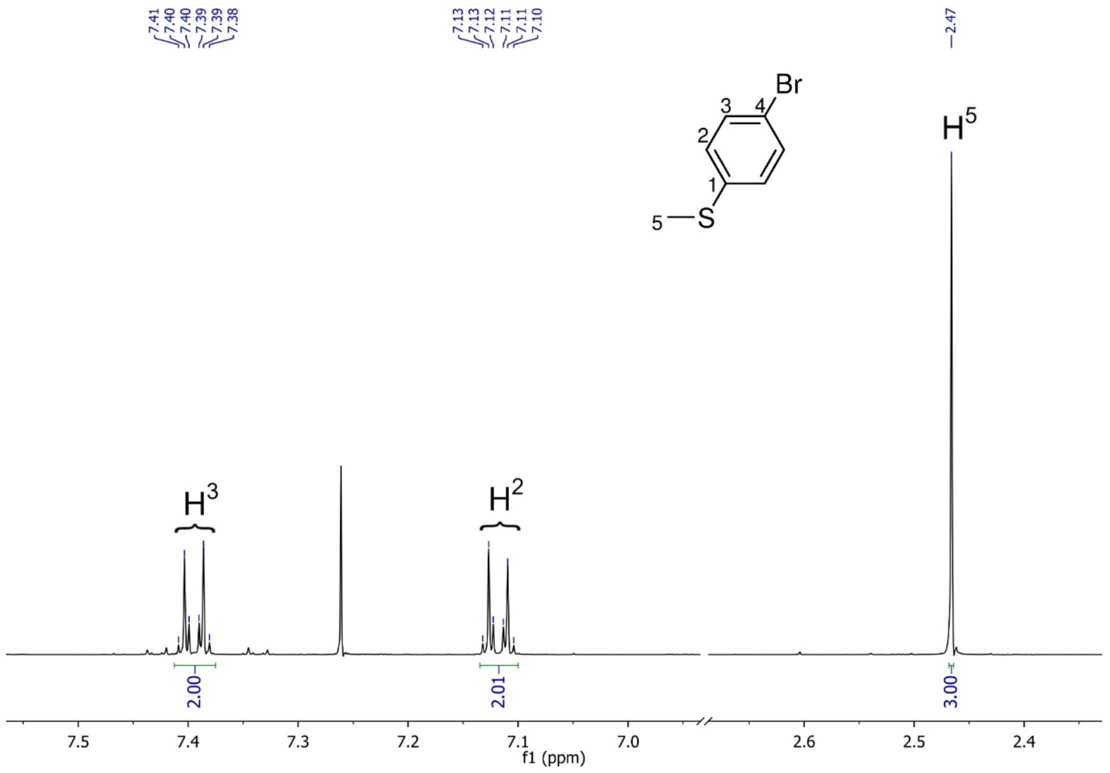

Figure S 1. ^{1}H-NMR (500 MHz, $CDCl_3$) spectrum of compound **2**.

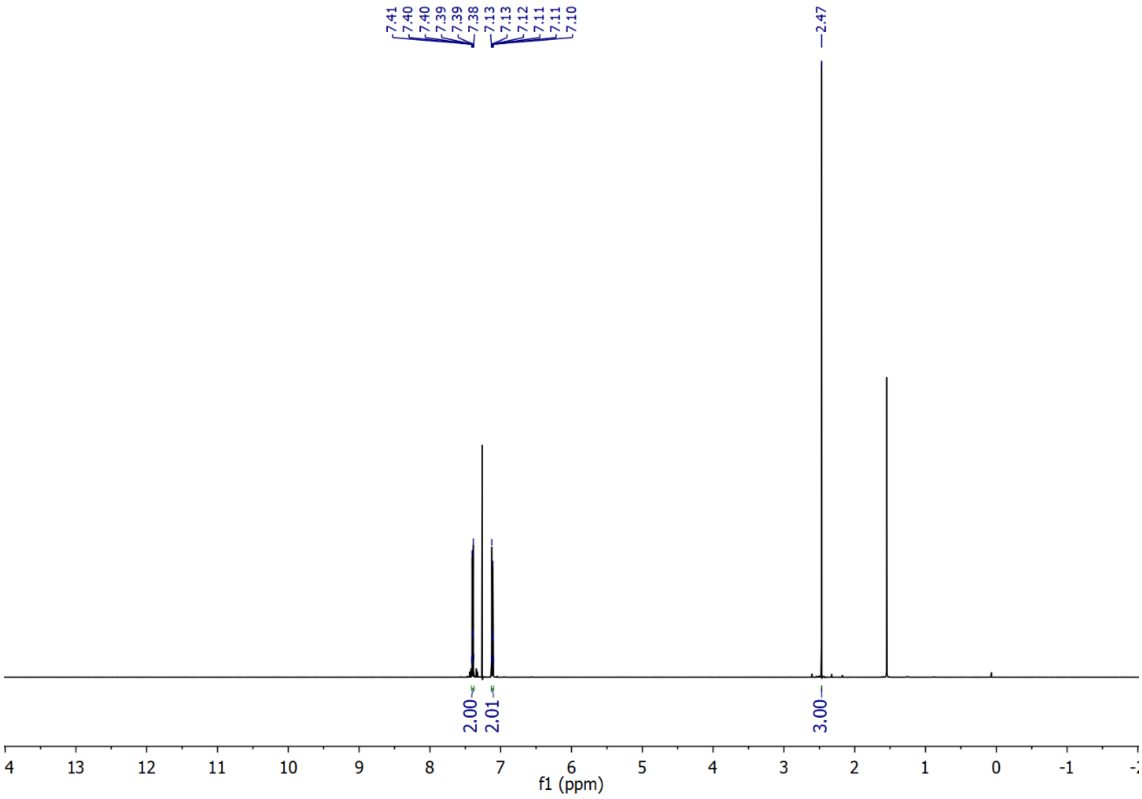

Figure S 2. Full ^{1}H-NMR (500 MHz, $CDCl_3$) spectrum of compound **2**.

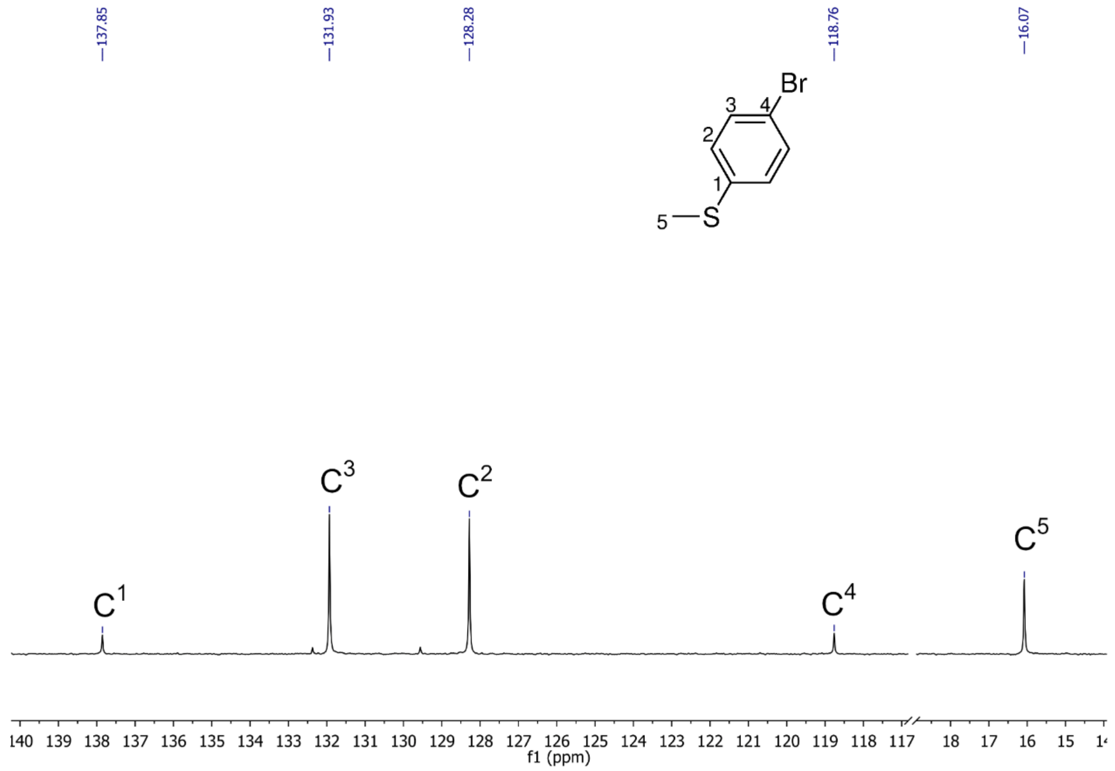

Figure S 3. $^{13}C\{^1H\}$-NMR (101 MHz, $CDCl_3$) spectrum of compound **2**.

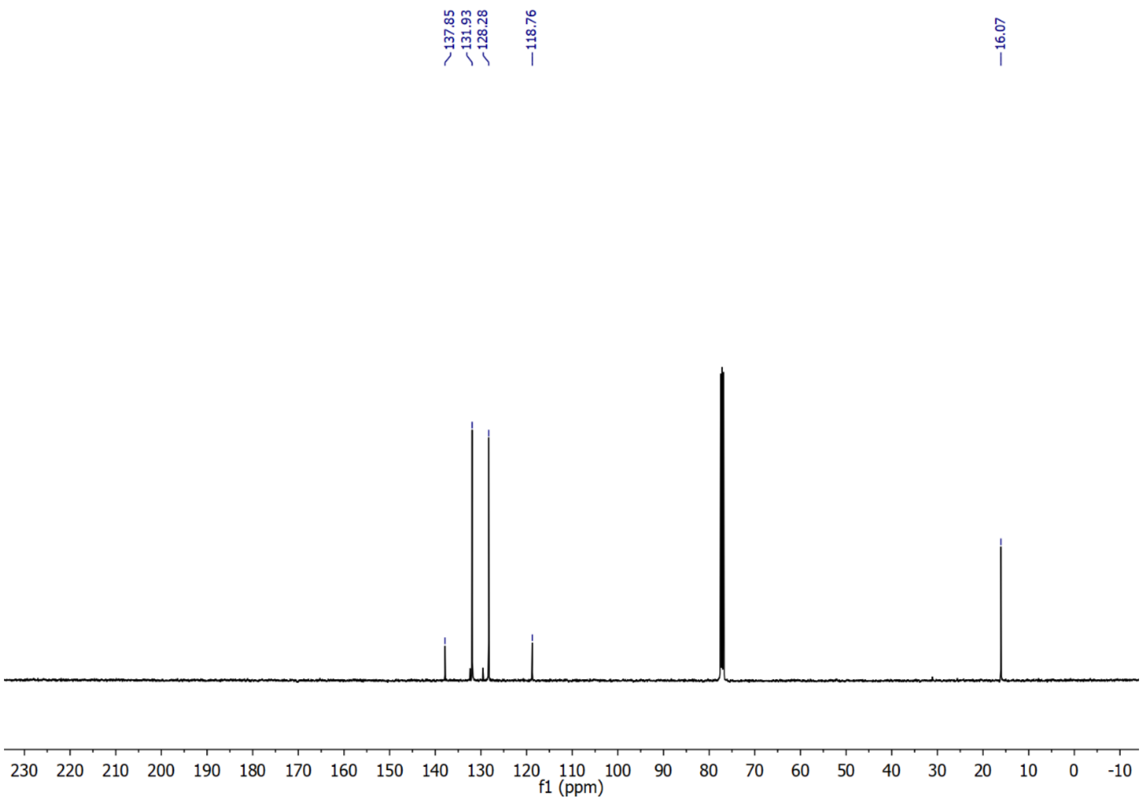

Figure S 4. Full $^{13}C\{^1H\}$-NMR (101 MHz, $CDCl_3$) spectrum of compound **2**.

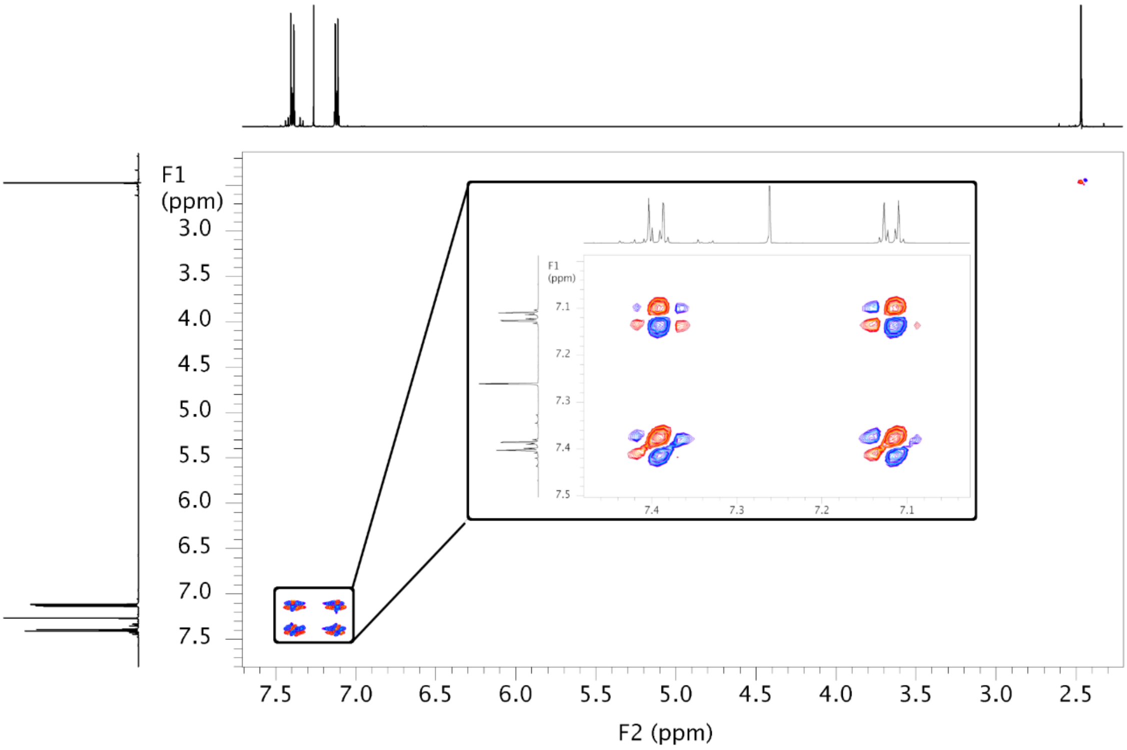

Figure S 5. ^{1}H-^{1}H gDQFCOSY (400 MHz, $CDCl_3$) spectrum of compound **2**.

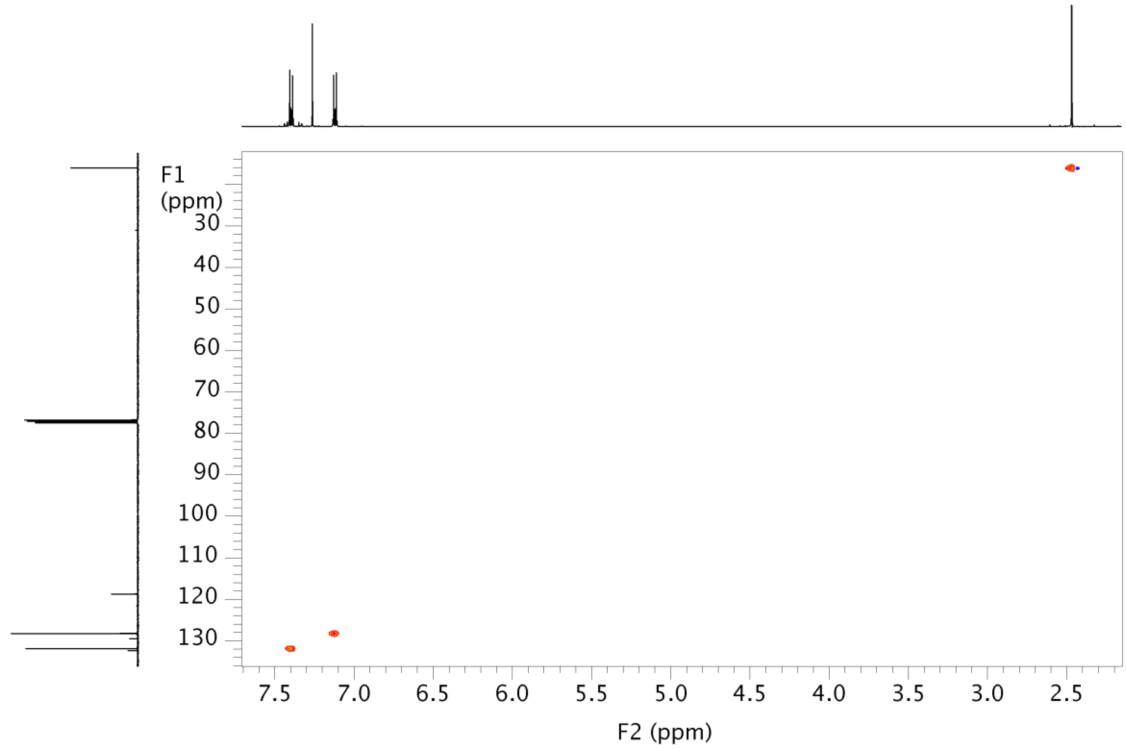

Figure S 6. ^{1}H-^{13}C gc2HSQC (400 MHz, $CDCl_3$) spectrum of compound **2**.

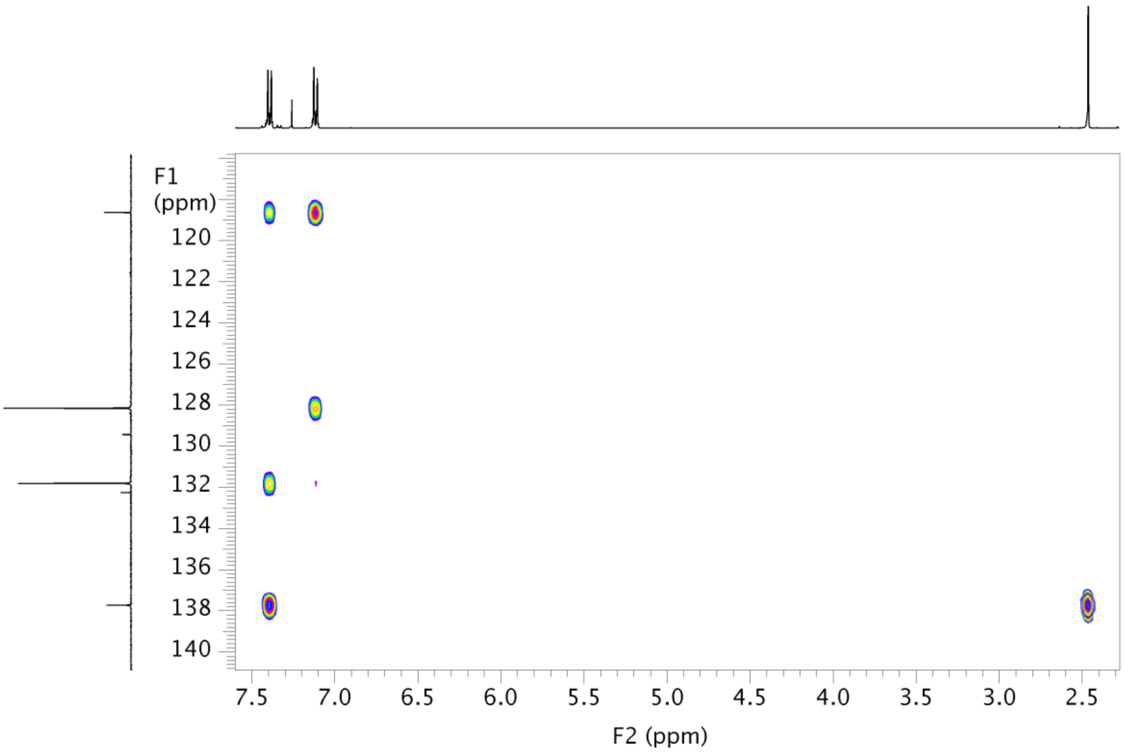

Figure S 7. ^{1}H-^{13}C gc2HMBC (400 MHz, $CDCl_3$) spectrum of compound **2**.

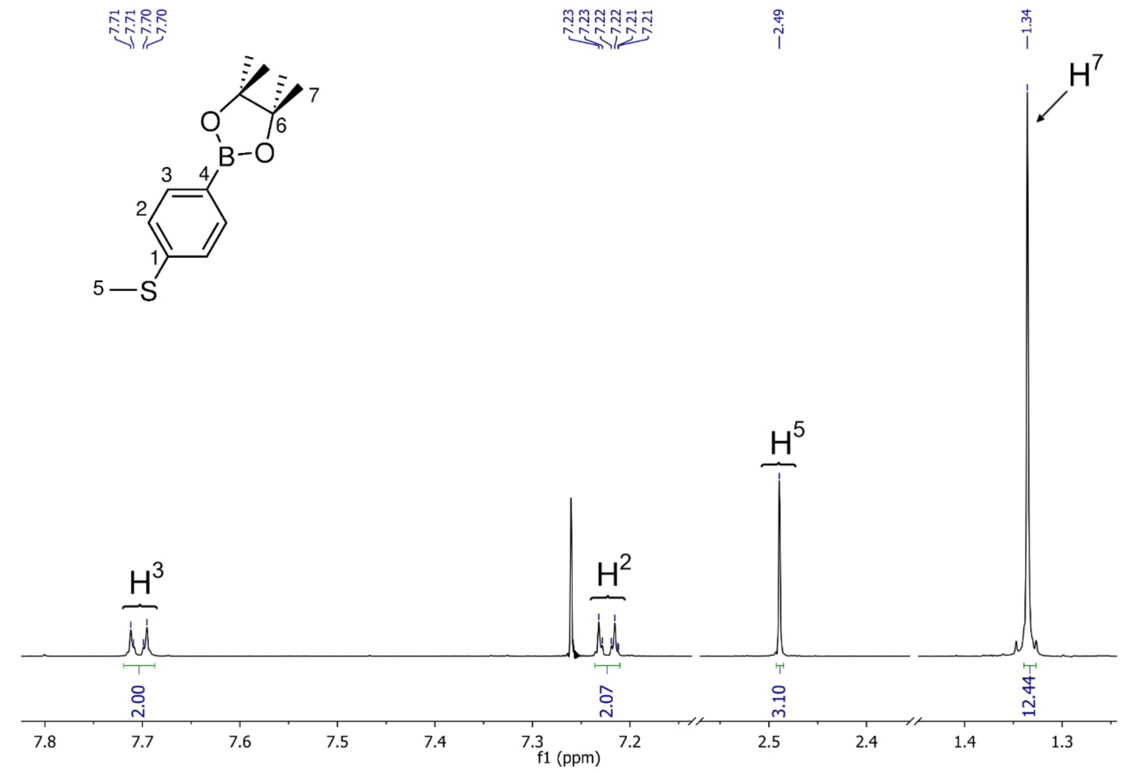

Figure S 8. ^{1}H-NMR (500 MHz, $CDCl_3$) spectrum of compound **3**.

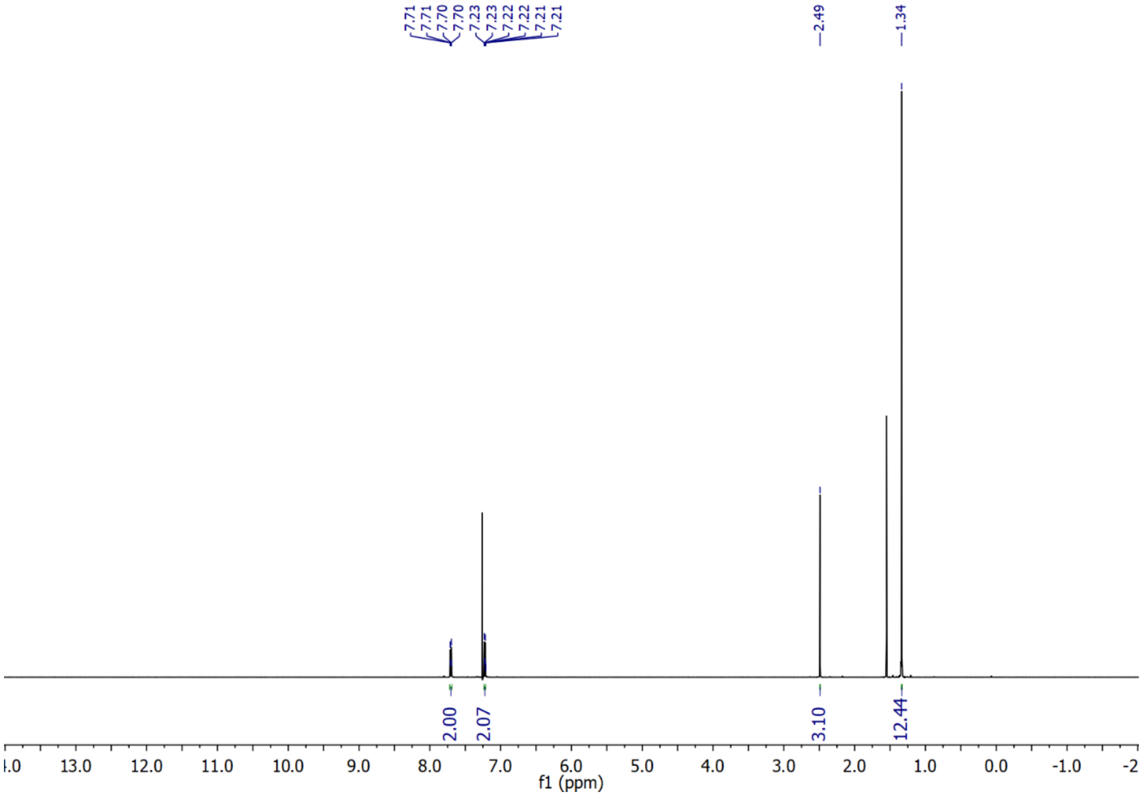

Figure S 9. Full ^{1}H-NMR (500 MHz, $CDCl_3$) spectrum of compound **3**.

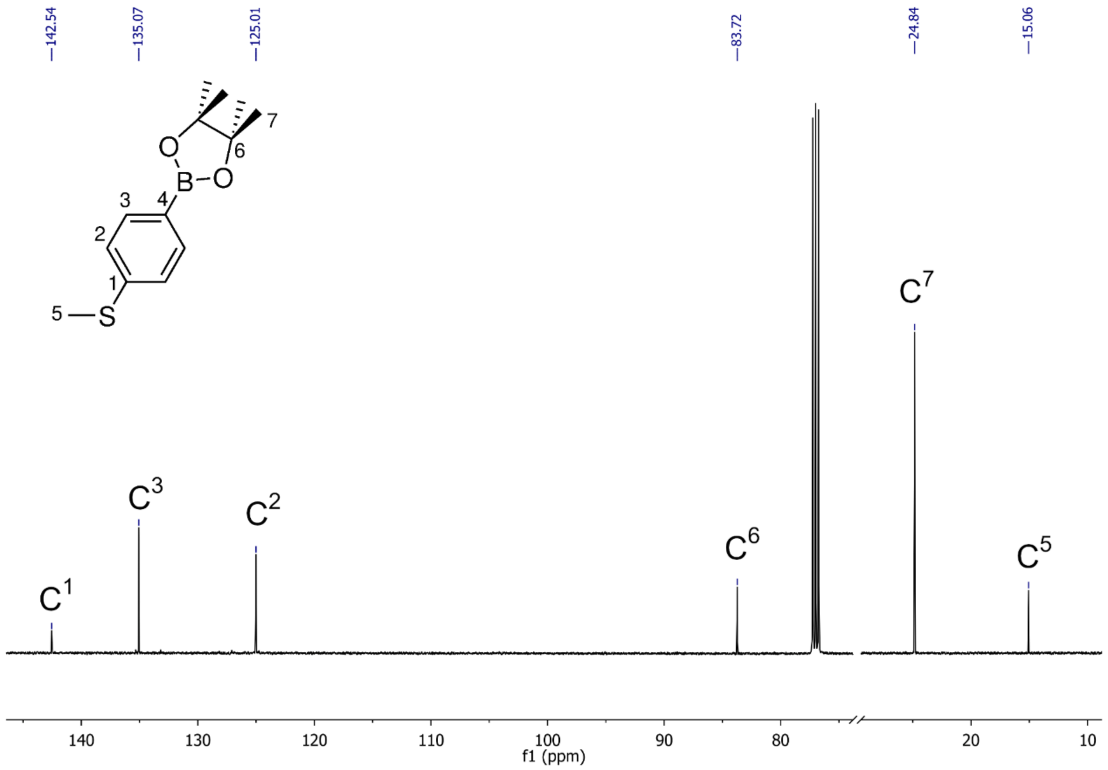

Figure S 10. $^{13}C\{^{1}H\}$-NMR (126 MHz, $CDCl_3$) spectrum of compound **3**.

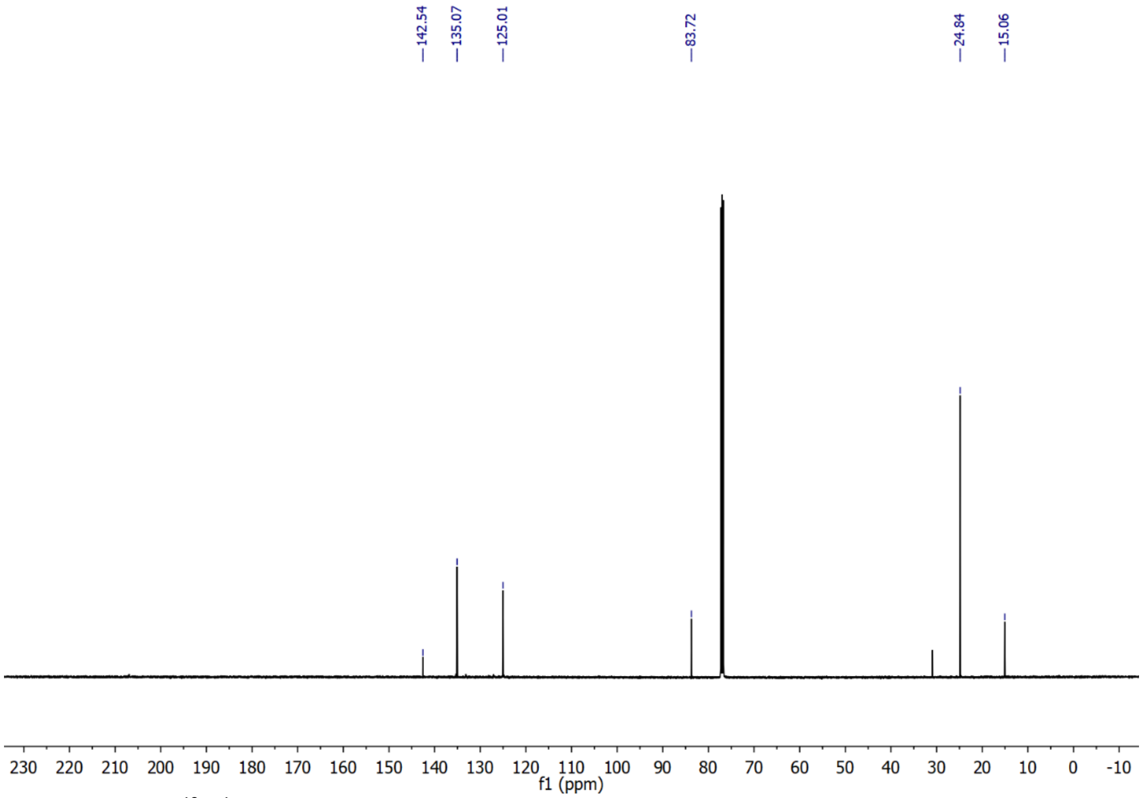

Figure S 11. Full $^{13}C\{^{1}H\}$-NMR (126 MHz, $CDCl_3$) spectrum of compound **3**.

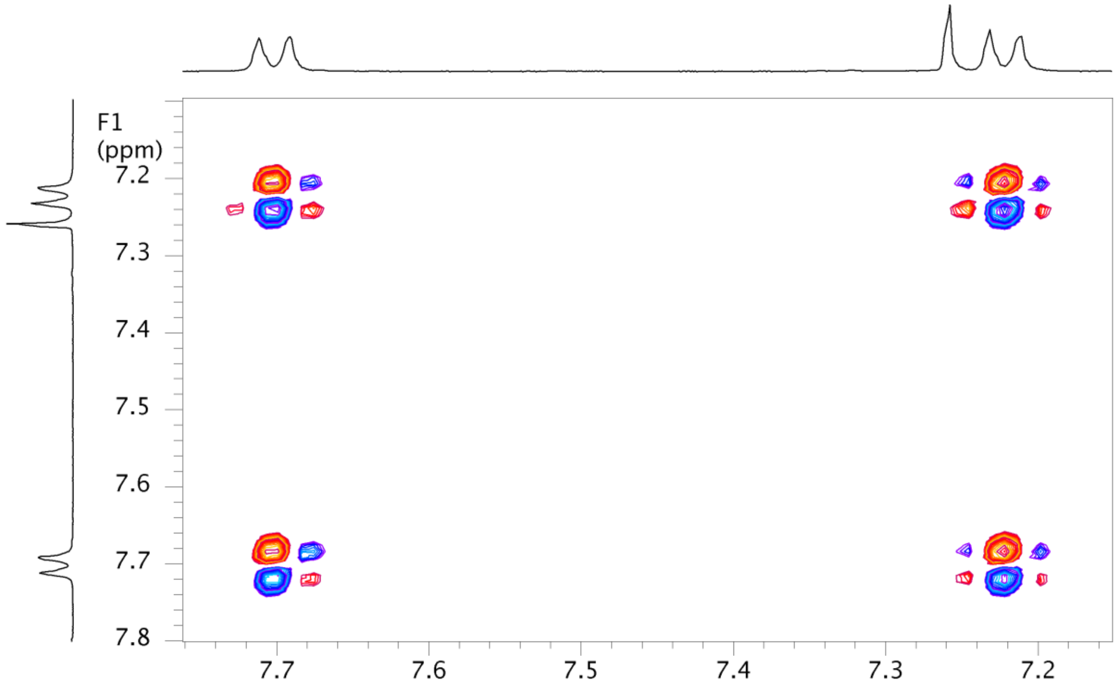

Figure S 12. ^{1}H-^{1}H gDQFCOSY (400 MHz, $CDCl_3$) spectrum of compound **3**.

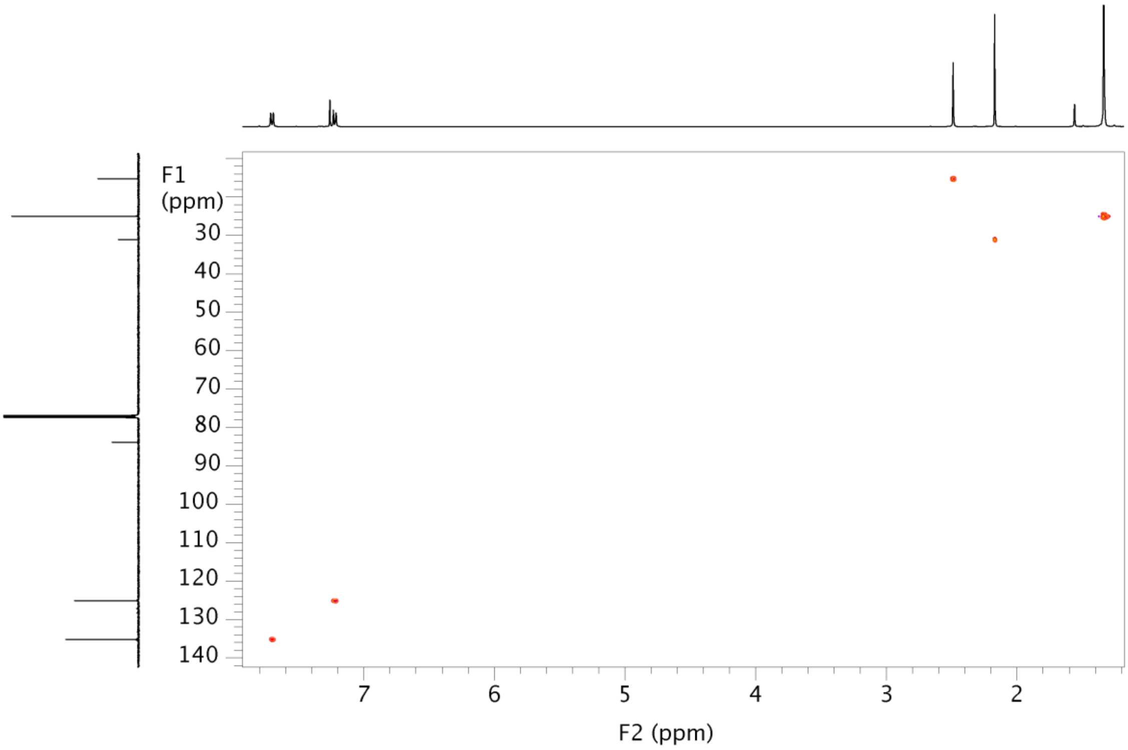

Figure S 13. ^{1}H-^{13}C gc2HSQC (400 MHz, $CDCl_3$) spectrum of compound **3**.

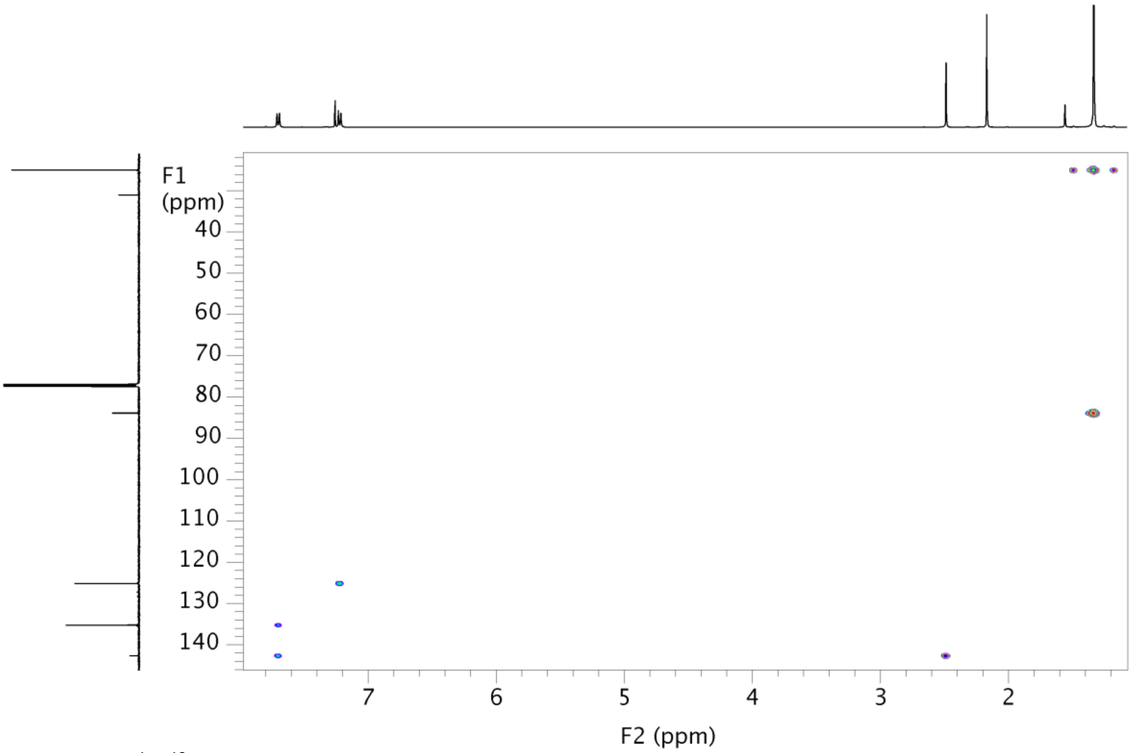

Figure S 14. ^{1}H-^{13}C gc2HMBC (400 MHz, $CDCl_3$) spectrum of compound **3**.

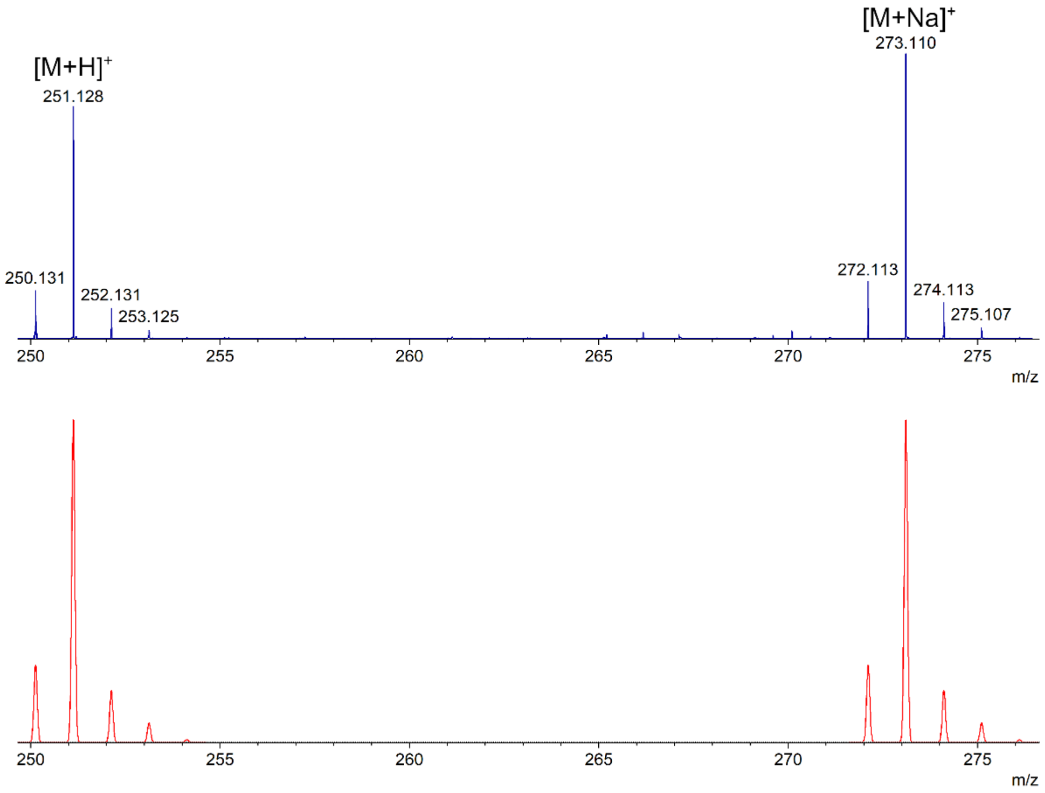

Figure S 15. HRMS (ESI-TOF), of compound **3**, $[M+H]^+$ and $[M+Na]^+$. Calculated (red), measured (blue).

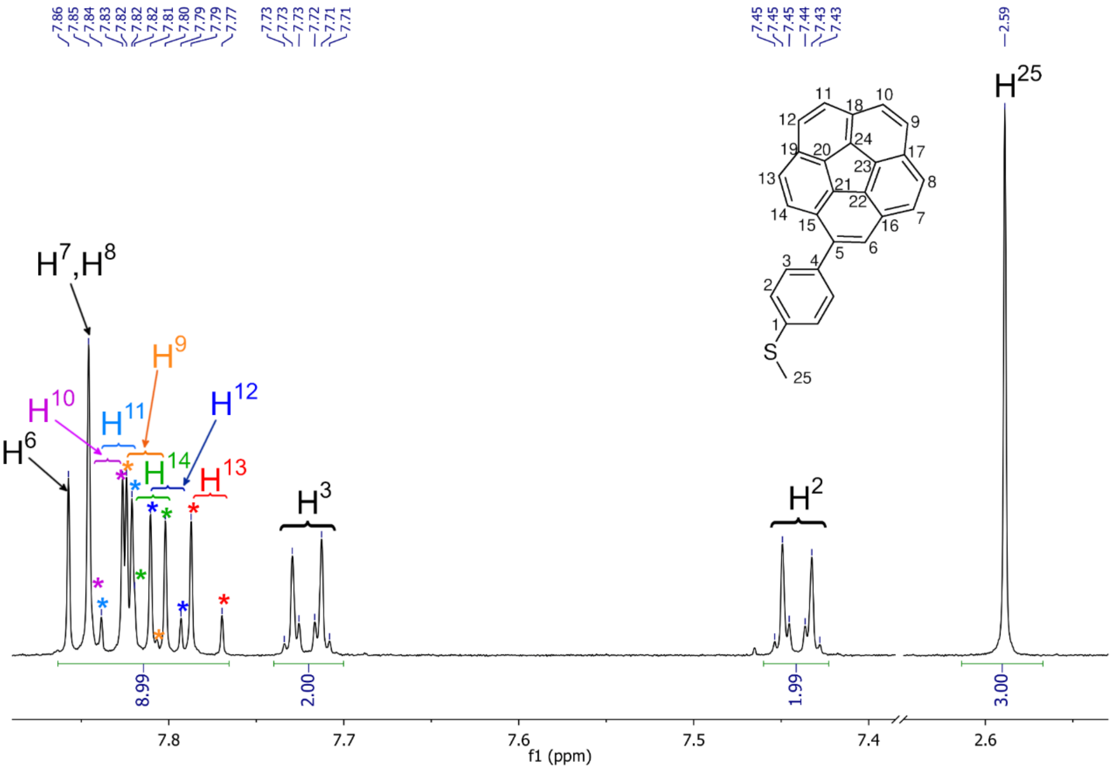

Figure S 16. ^{1}H-NMR (500 MHz, $CDCl_3$) spectrum of compound **4**.

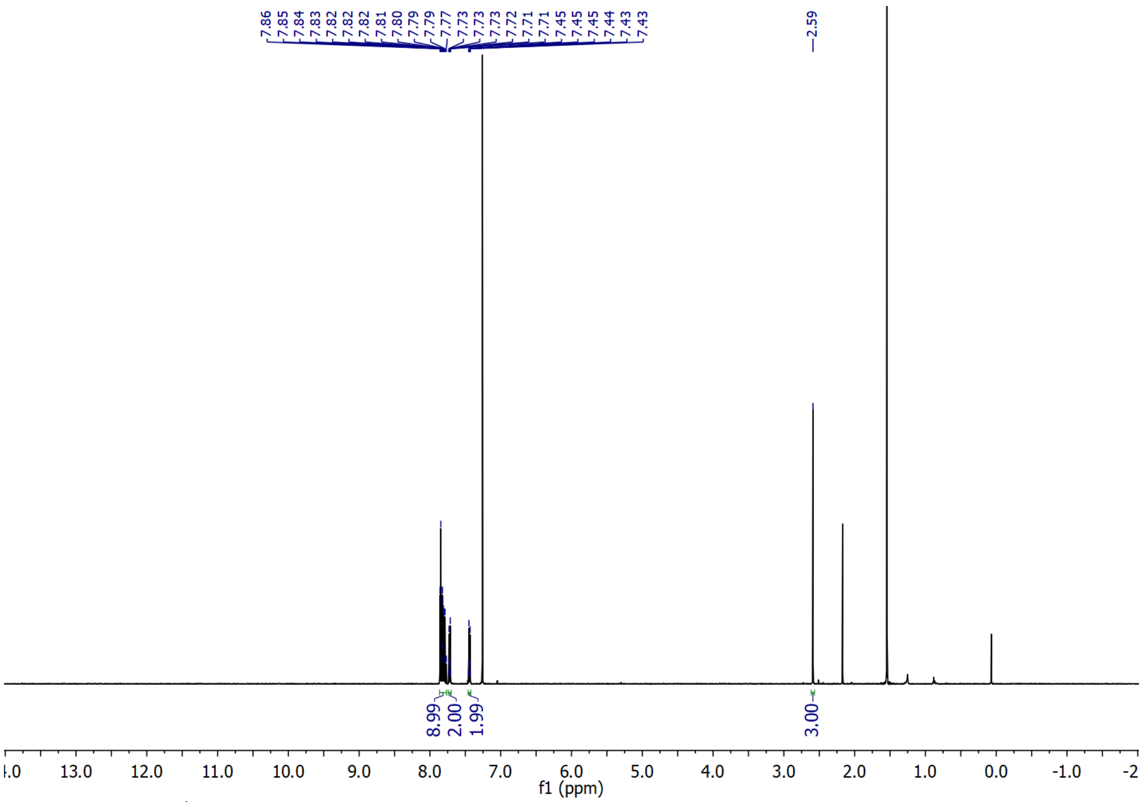

Figure S 17. Full ^{1}H-NMR (500 MHz, $CDCl_3$) spectrum of compound **4**.

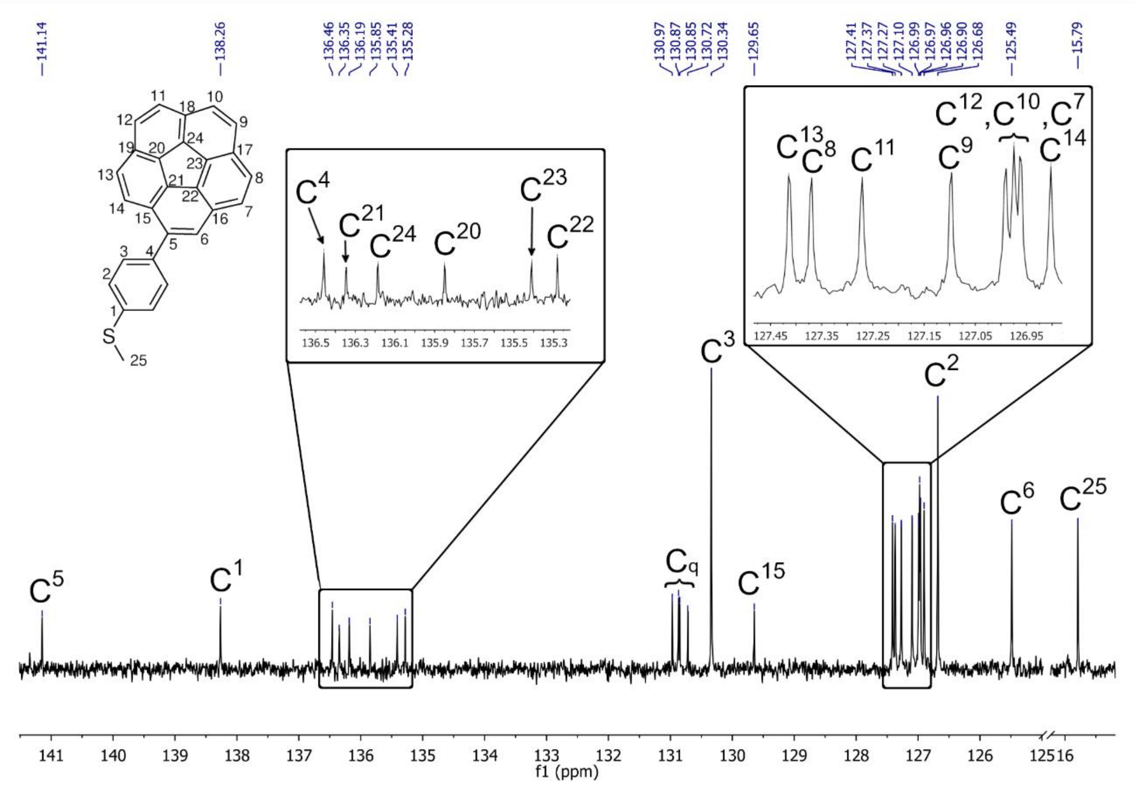

Figure S 18. $^{13}C\{^1H\}$-NMR (126 MHz, $CDCl_3$) spectrum of compound **4**.

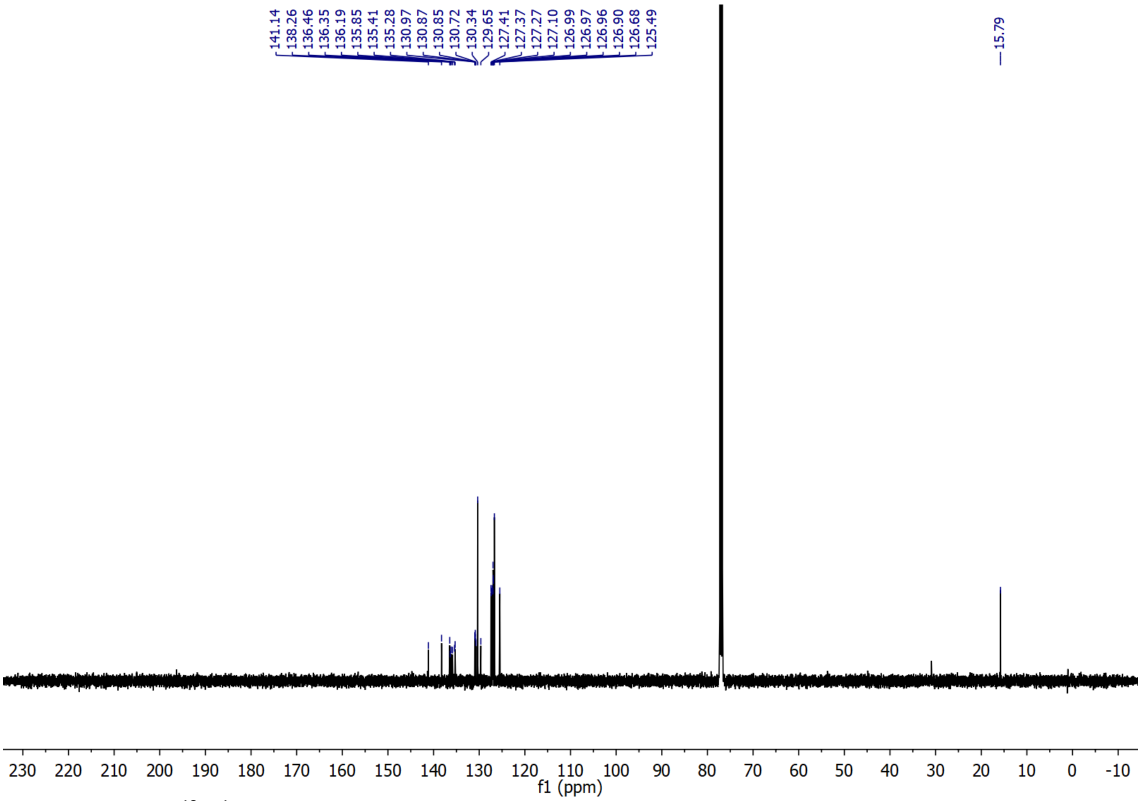

Figure S 19. Full $^{13}C\{^1H\}$-NMR (126 MHz, $CDCl_3$) spectrum of compound **4**.

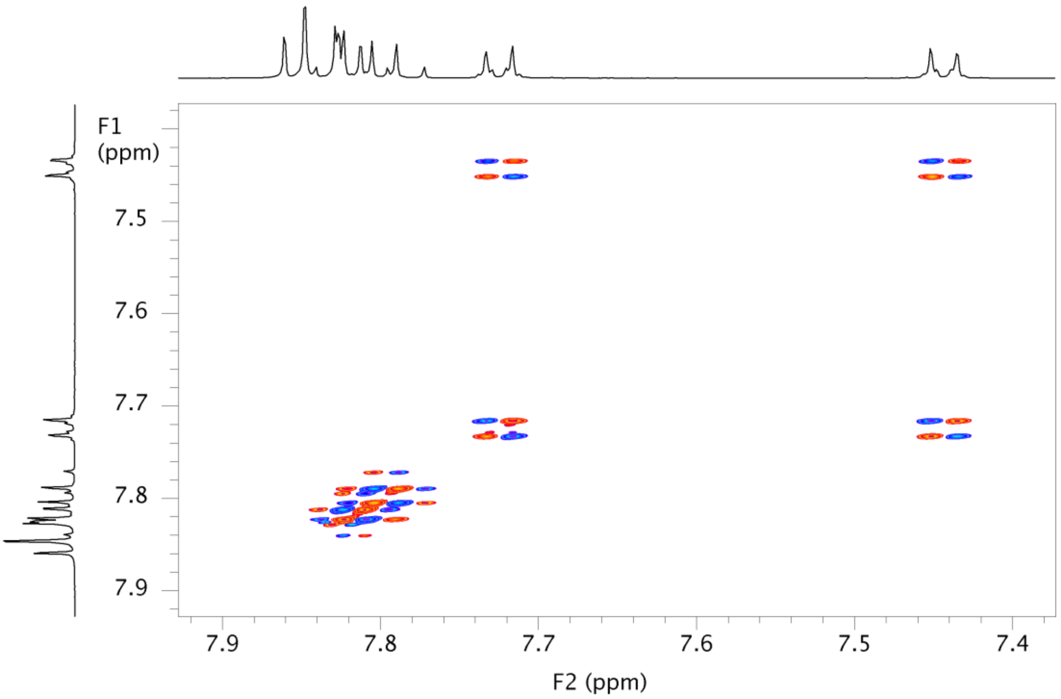

Figure S 20. ^{1}H-^{1}H gDQFCOSY (500 MHz, $CDCl_3$) spectrum of compound **4**.

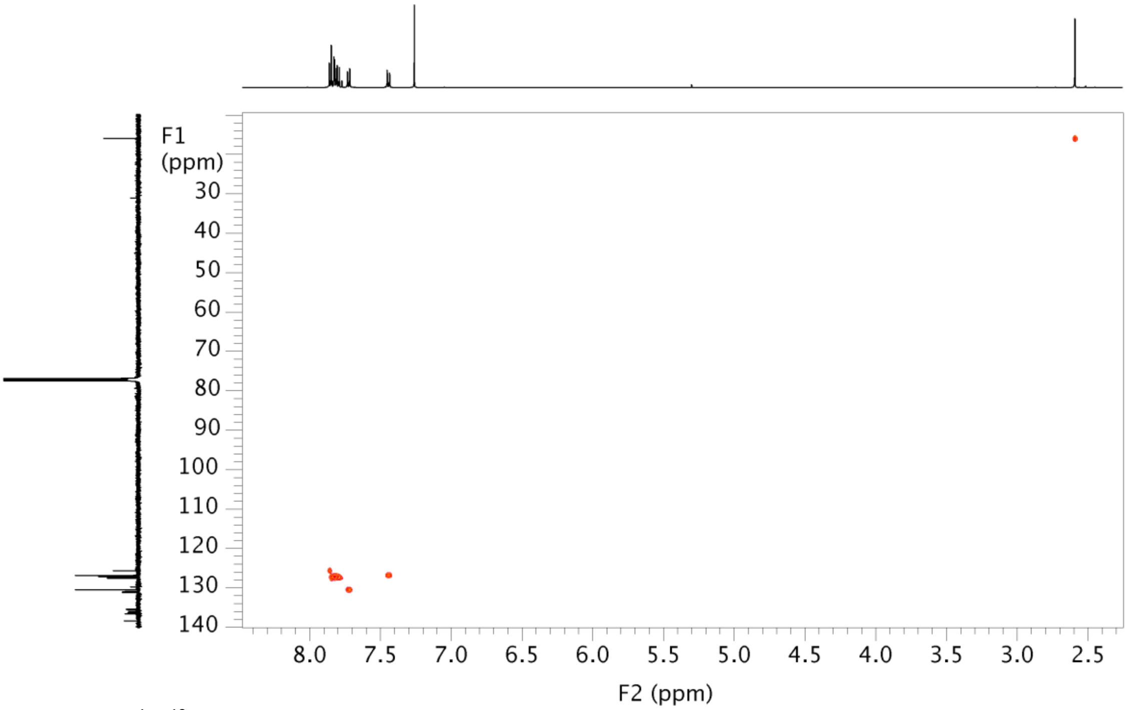

Figure S 21. ^{1}H-^{13}C gc2HSQC (500 MHz, $CDCl_3$) spectrum of compound **4**.

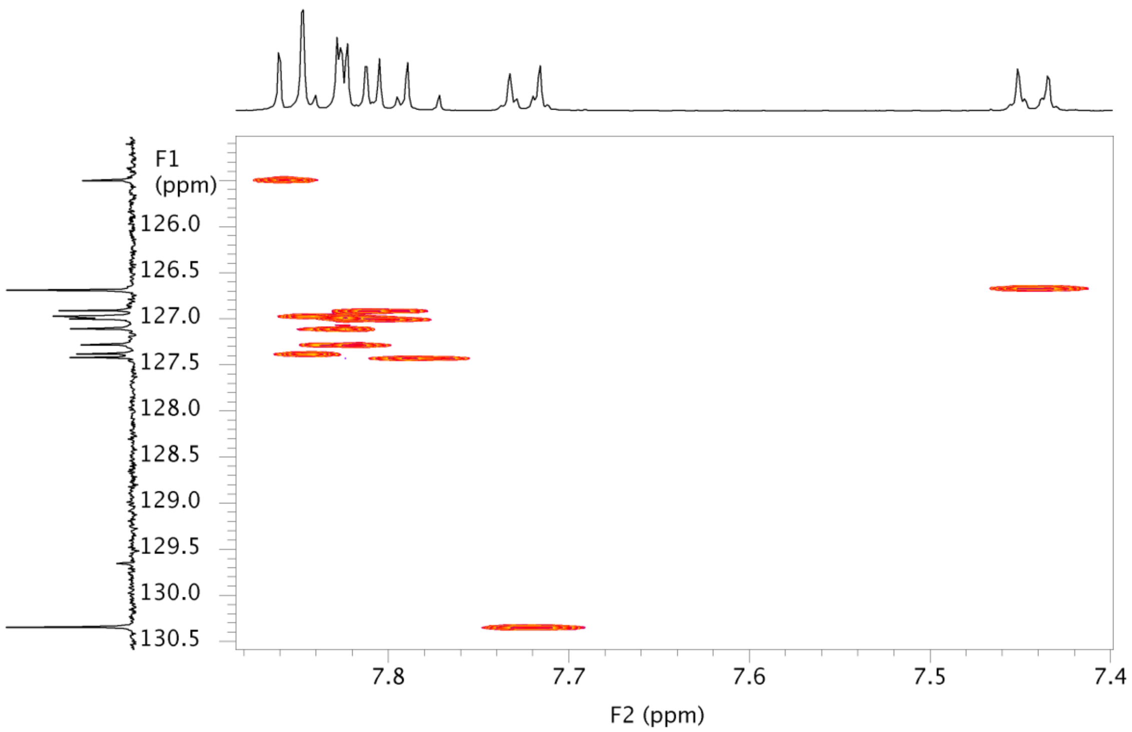

Figure S 22. ^{1}H-^{13}C bsgHSQCAD (500 MHz, $CDCl_3$) spectrum of compound **4**.

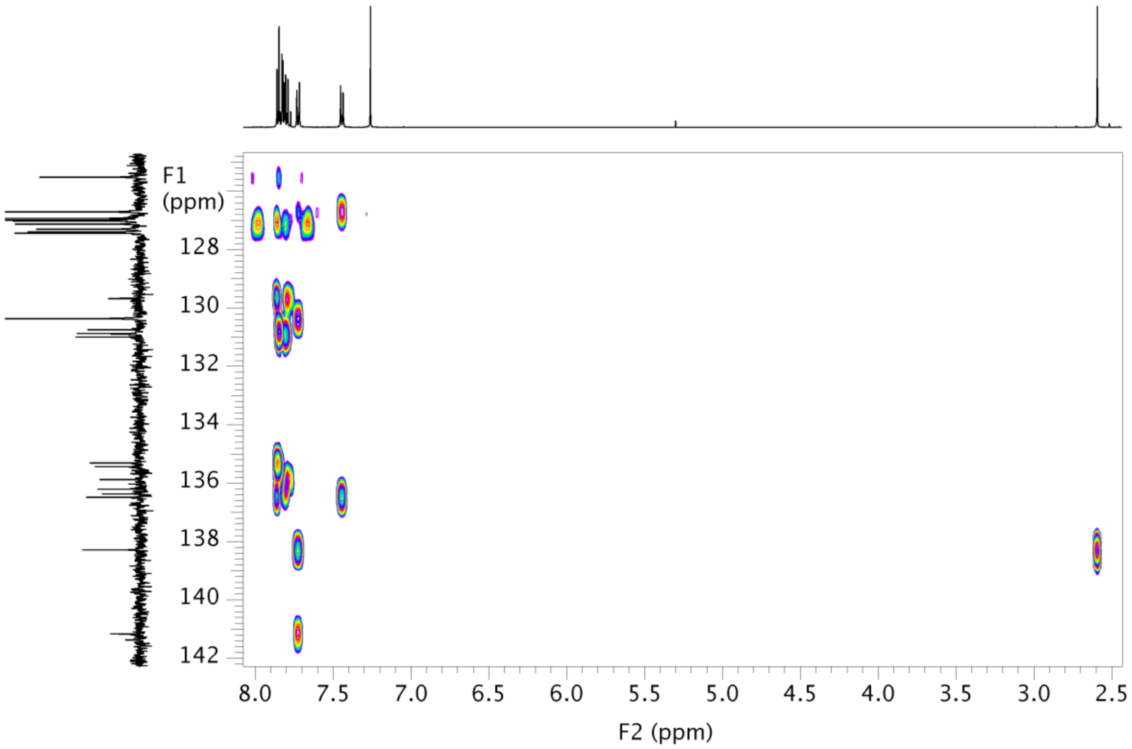

Figure S 23. ^{1}H-^{13}C gc2HMBC (500 MHz, $CDCl_3$) spectrum of compound **4**.

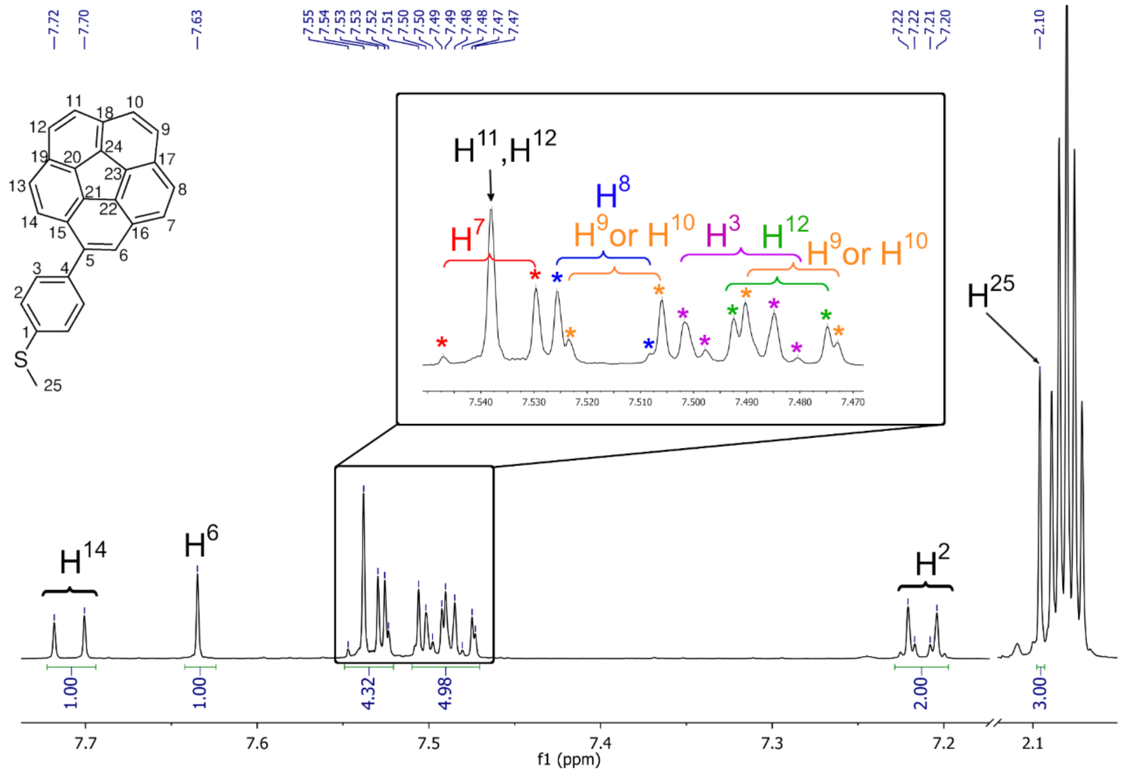

Figure S 24. ^{1}H-NMR (500 MHz, Toluene-d_8) spectrum of compound **4**.

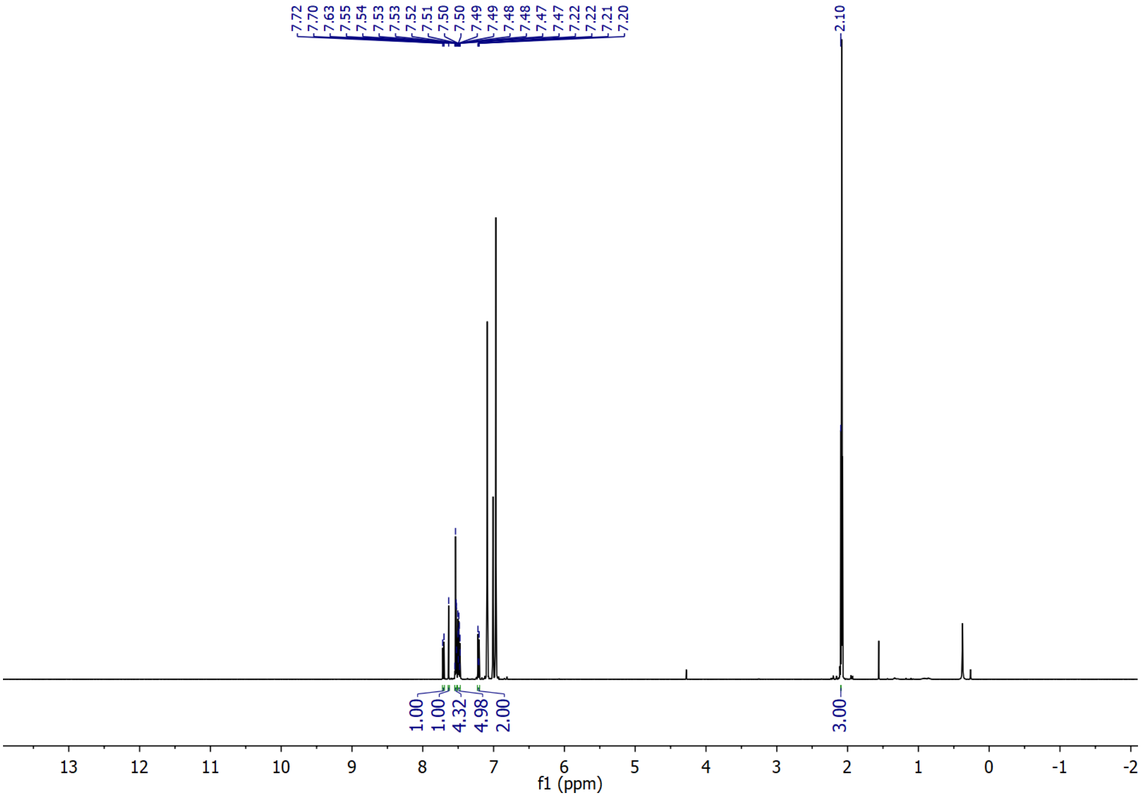

Figure S 25. Full ^{1}H-NMR (500 MHz, Toluene-d_8) spectrum of compound **4**.

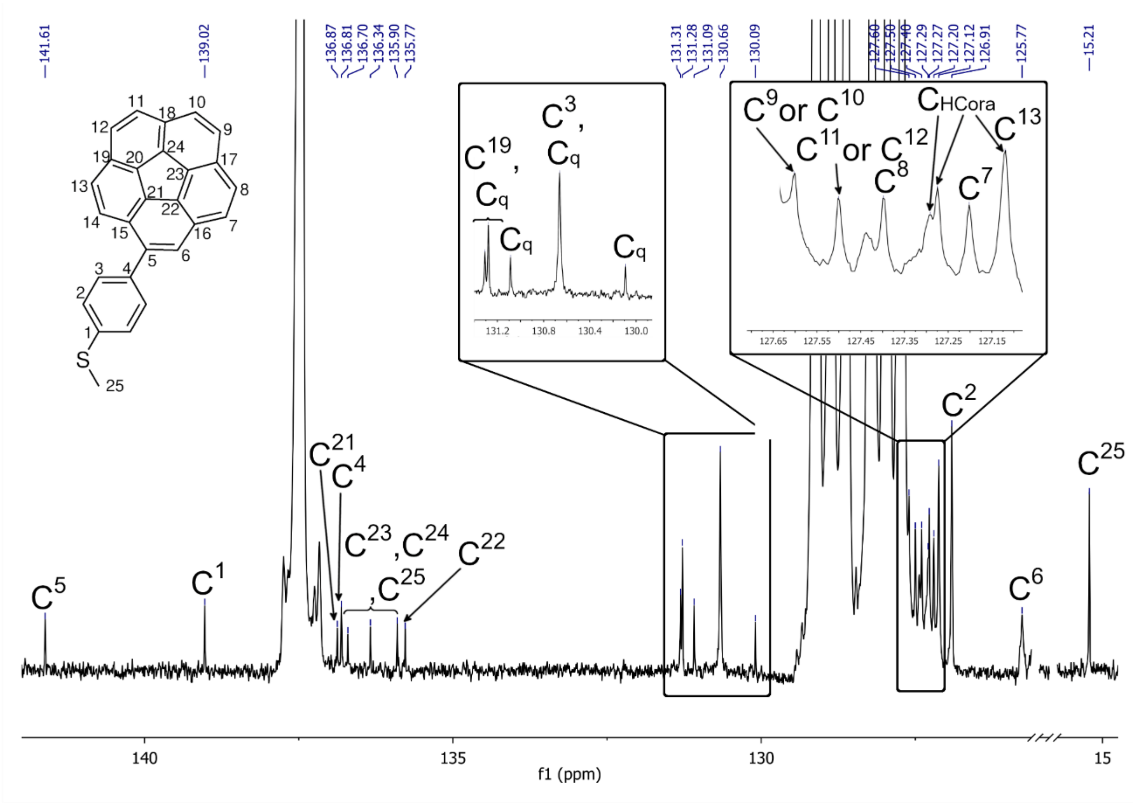

Figure S 26. $^{13}C\{^1H\}$-NMR (101 MHz, Toluene-d_8) spectrum of compound **4**.

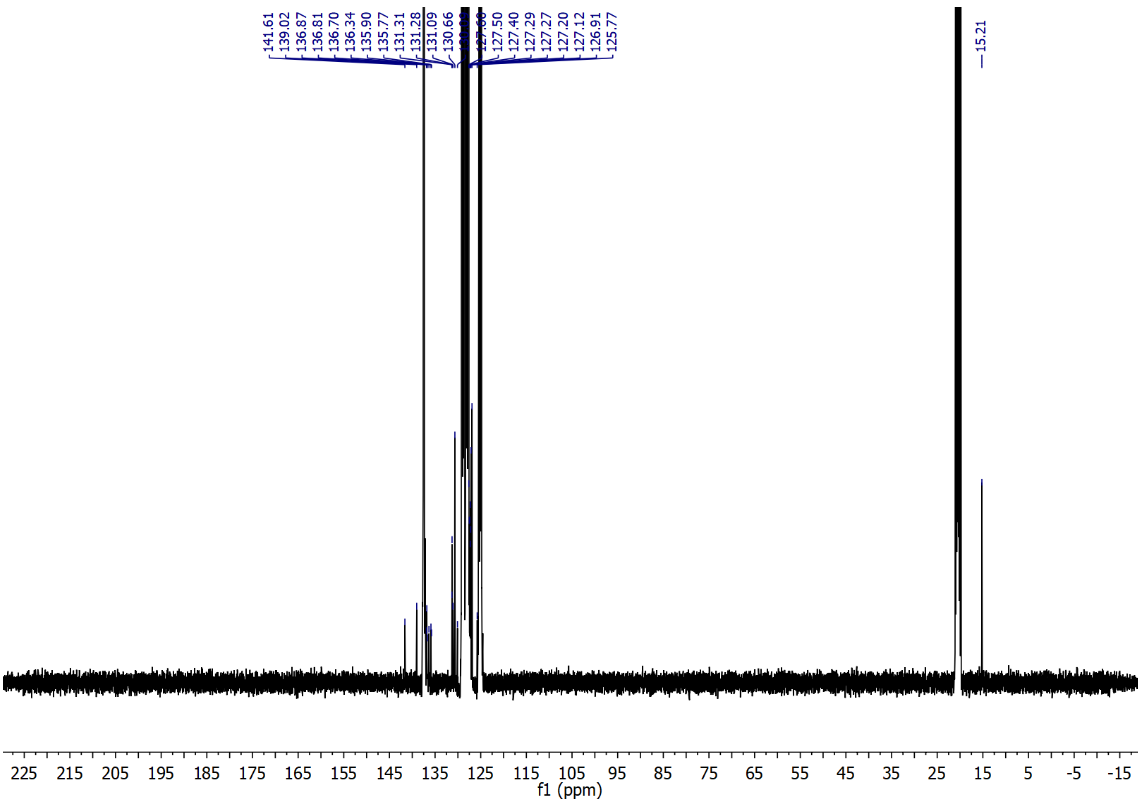

Figure S 27. Full $^{13}C\{^1H\}$-NMR (101 MHz, Toluene-d_8) spectrum of compound **4**.

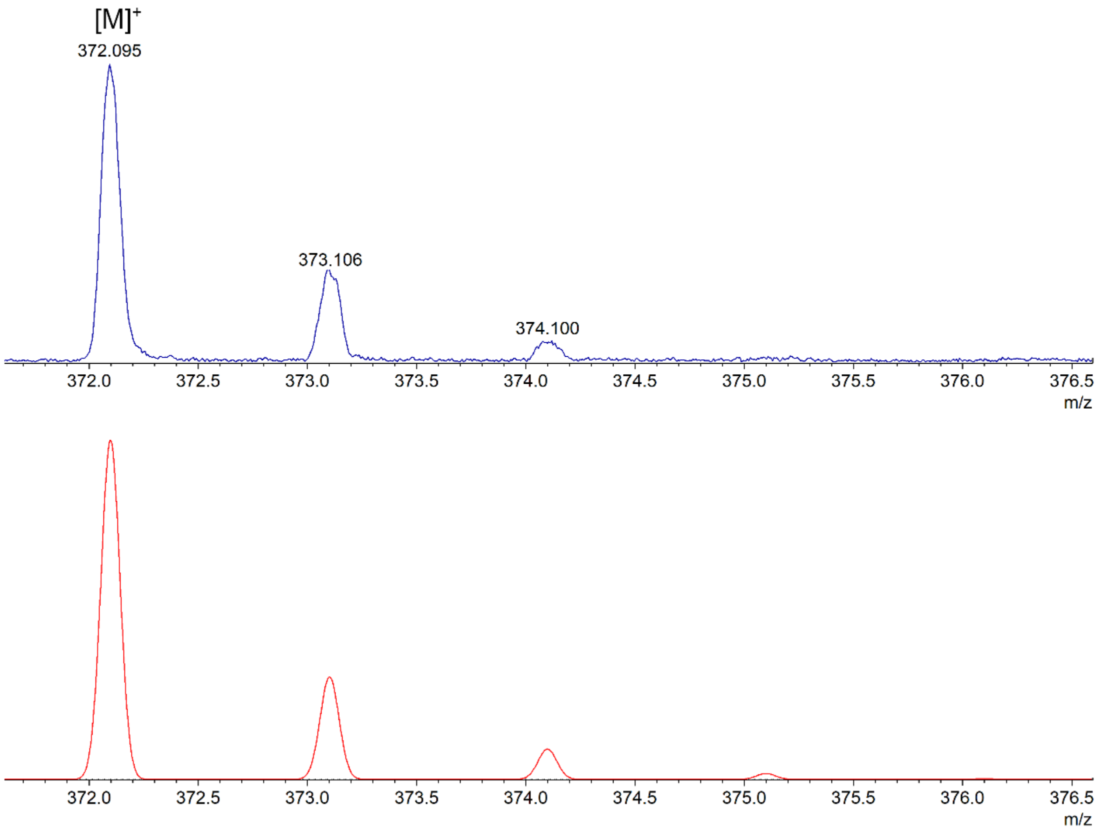

Figure S 28. HRMS (MALDI-TOF), of compound **4** $[M]^{+}$. Calculated (red), measured (blue).

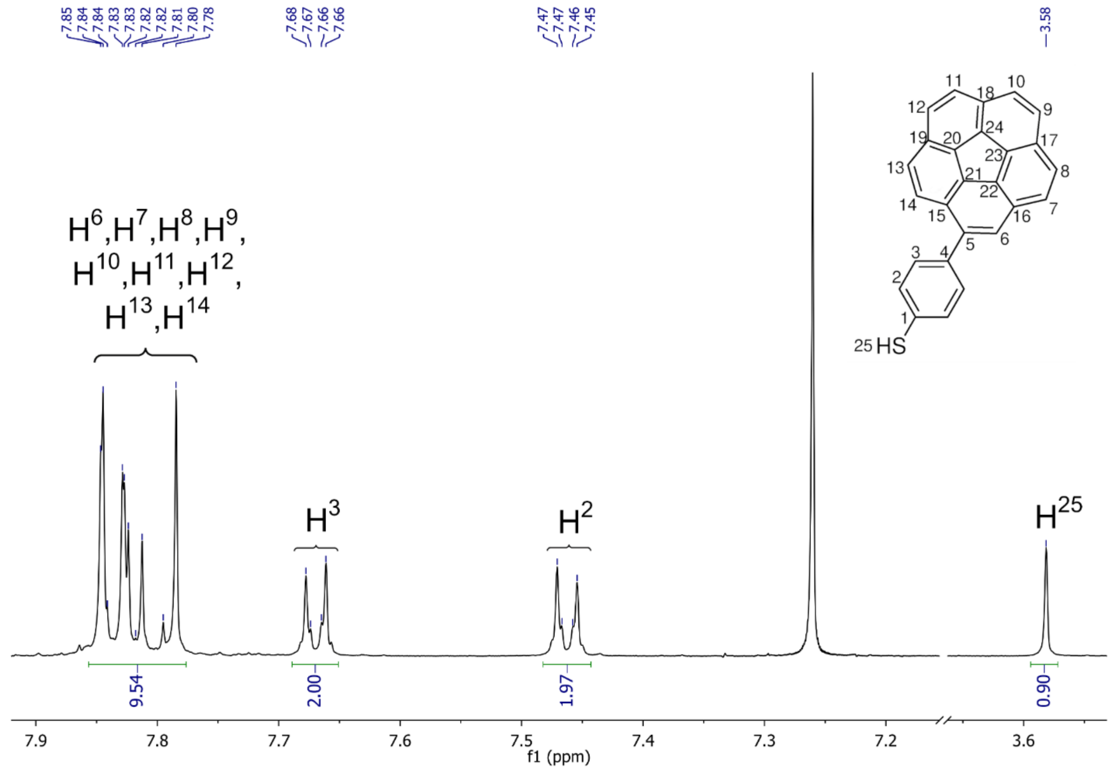

Figure S 29. ^1H-NMR (500 MHz, $CDCl_3$) spectrum of compound **5-SH**.

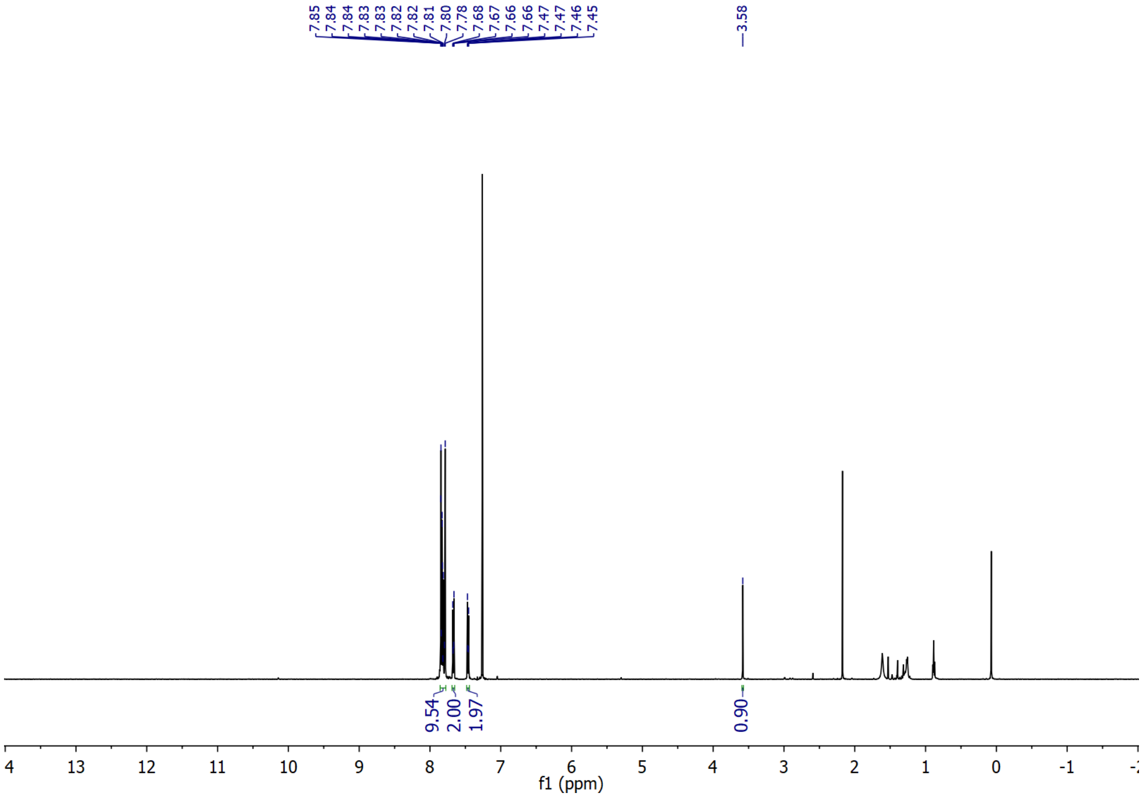

Figure S 30. Full ^1H-NMR (500 MHz, $CDCl_3$) spectrum of compound **5-SH**.

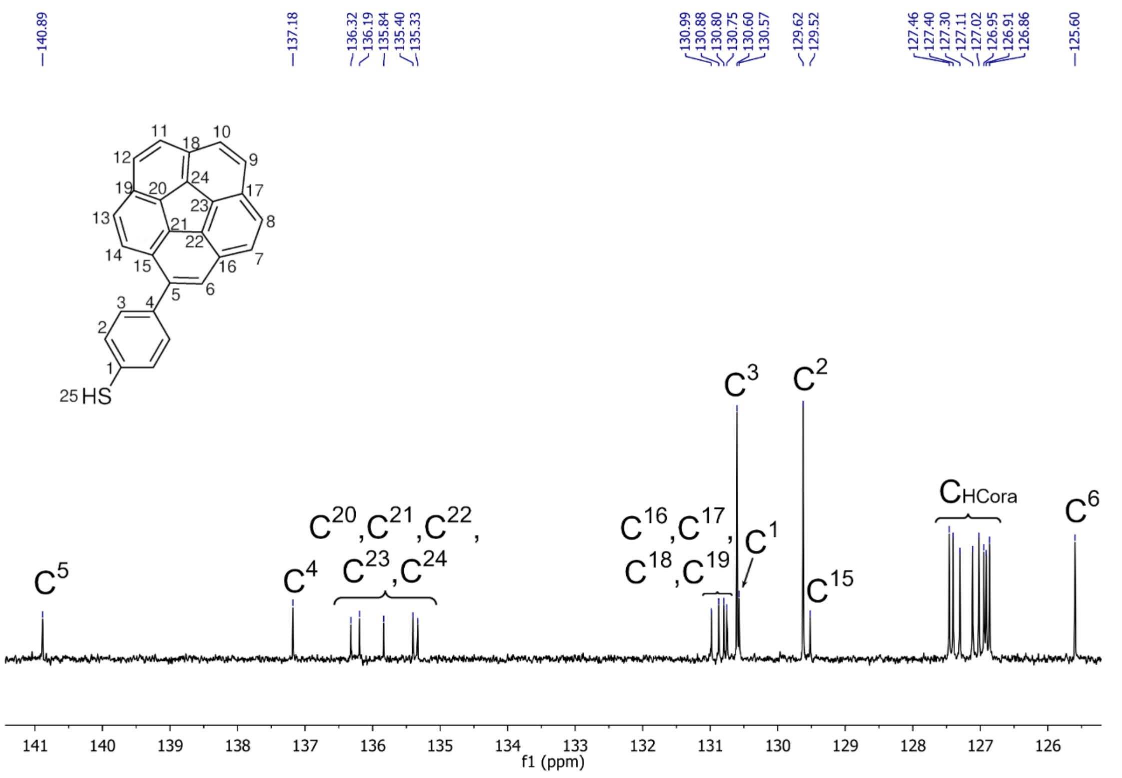

Figure S 31. $^{13}C\{^1H\}$-NMR (126 MHz, $CDCl_3$) spectrum of compound **5-SH**.

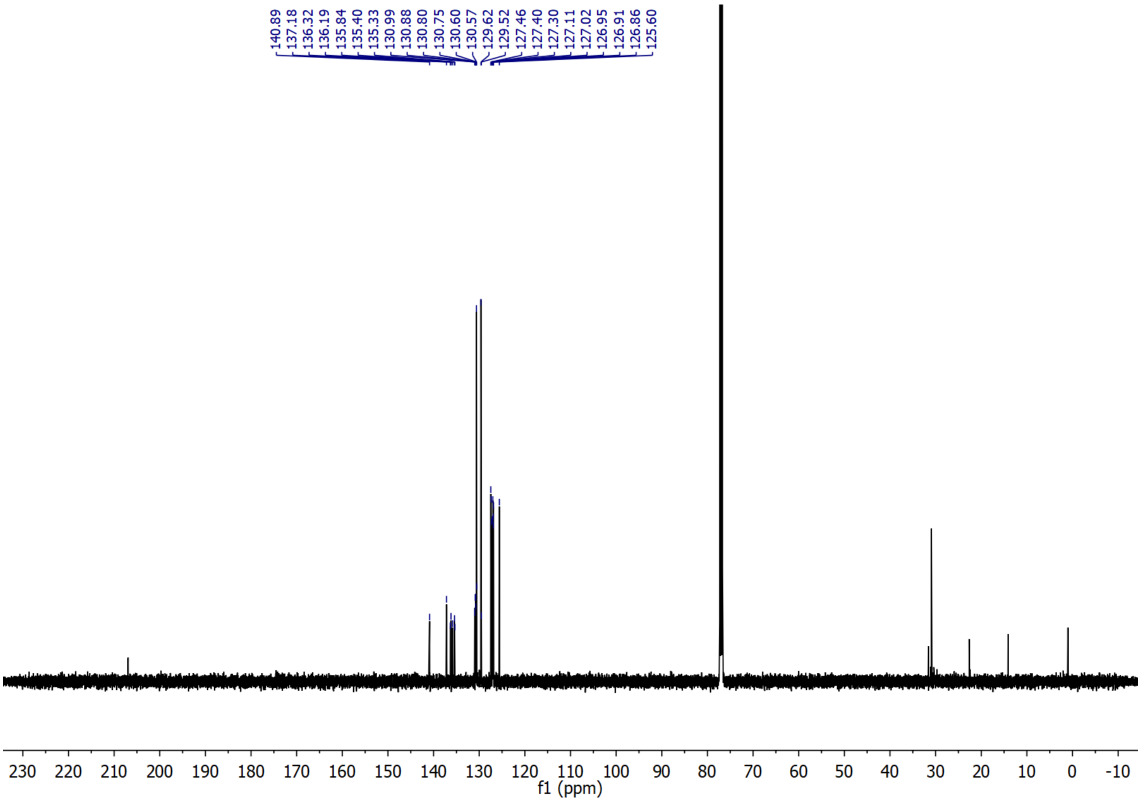

Figure S 32. Full $^{13}C\{^1H\}$-NMR (126 MHz, $CDCl_3$) spectrum of compound **5-SH**.

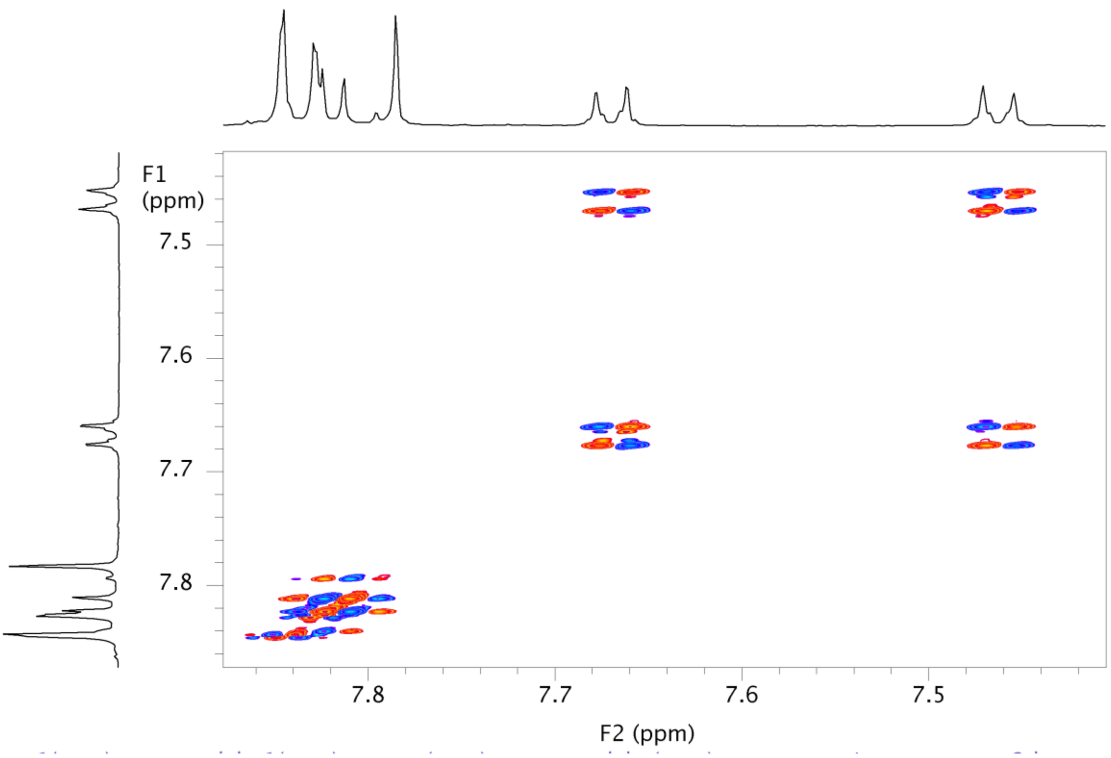

Figure S 33. ^{1}H-^{1}H gDQFCOSY (500 MHz, $CDCl_3$) spectrum of compound **5-SH**.

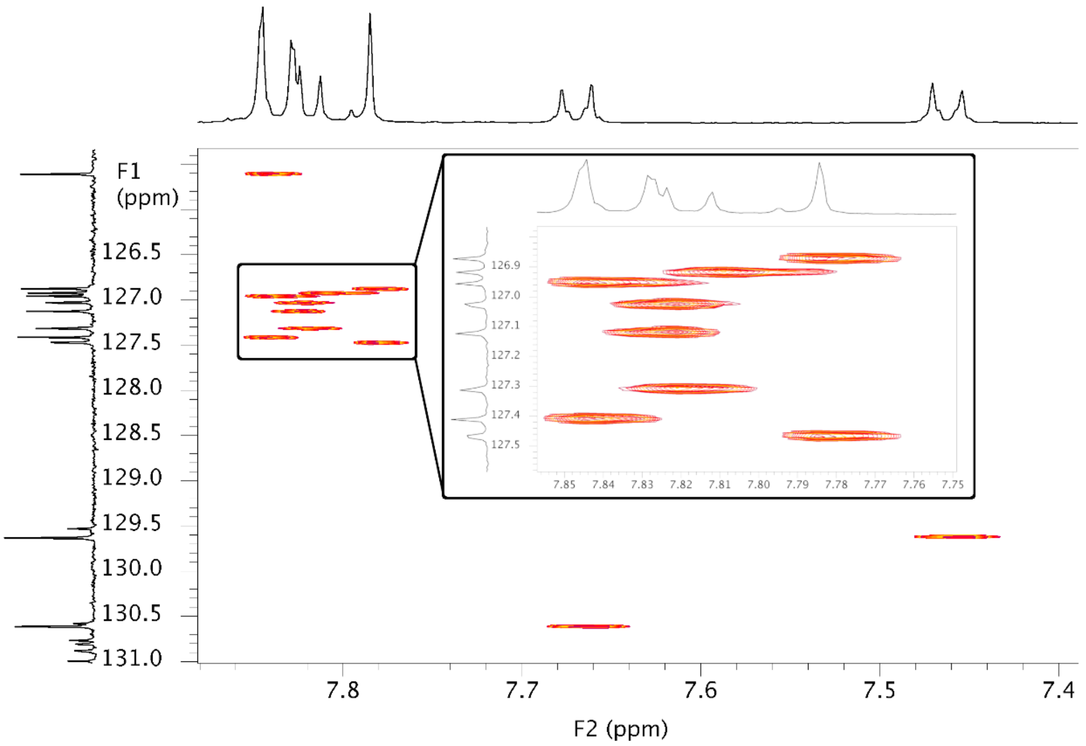

Figure S 34. ^{1}H-^{13}C bsgHSQCAD (500 MHz, $CDCl_3$) spectrum of compound **5-SH**.

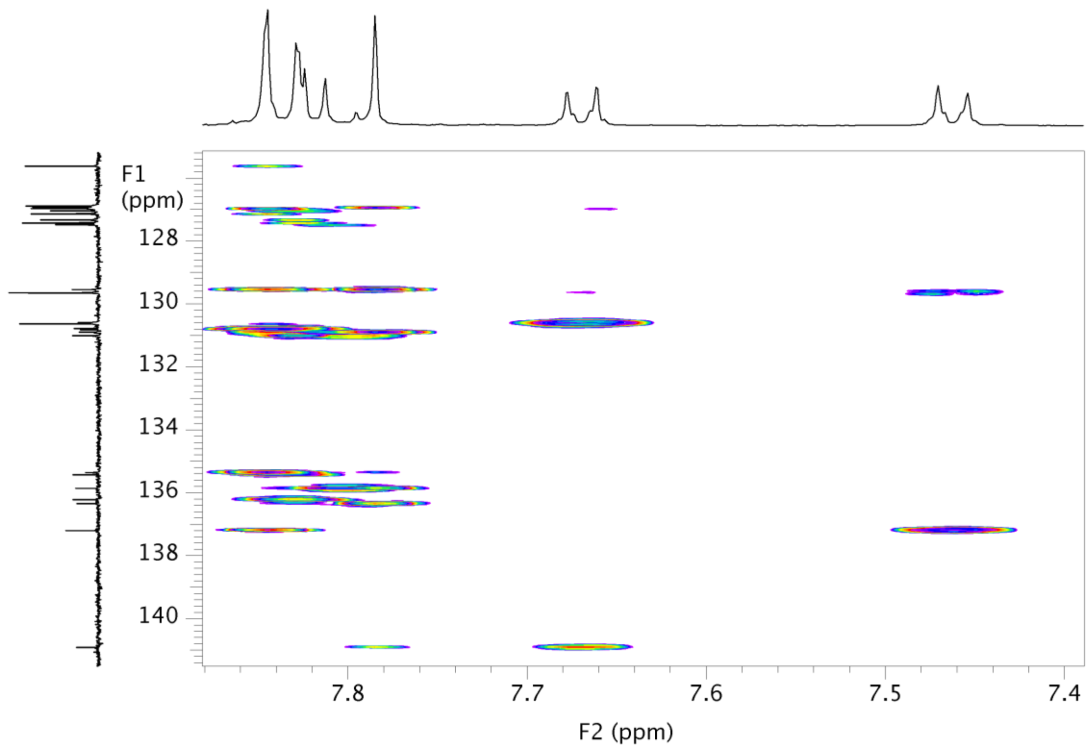

Figure S 35. ^{1}H-^{13}C bsgHMBC (500 MHz, $CDCl_3$) spectrum of compound **5-SH**.

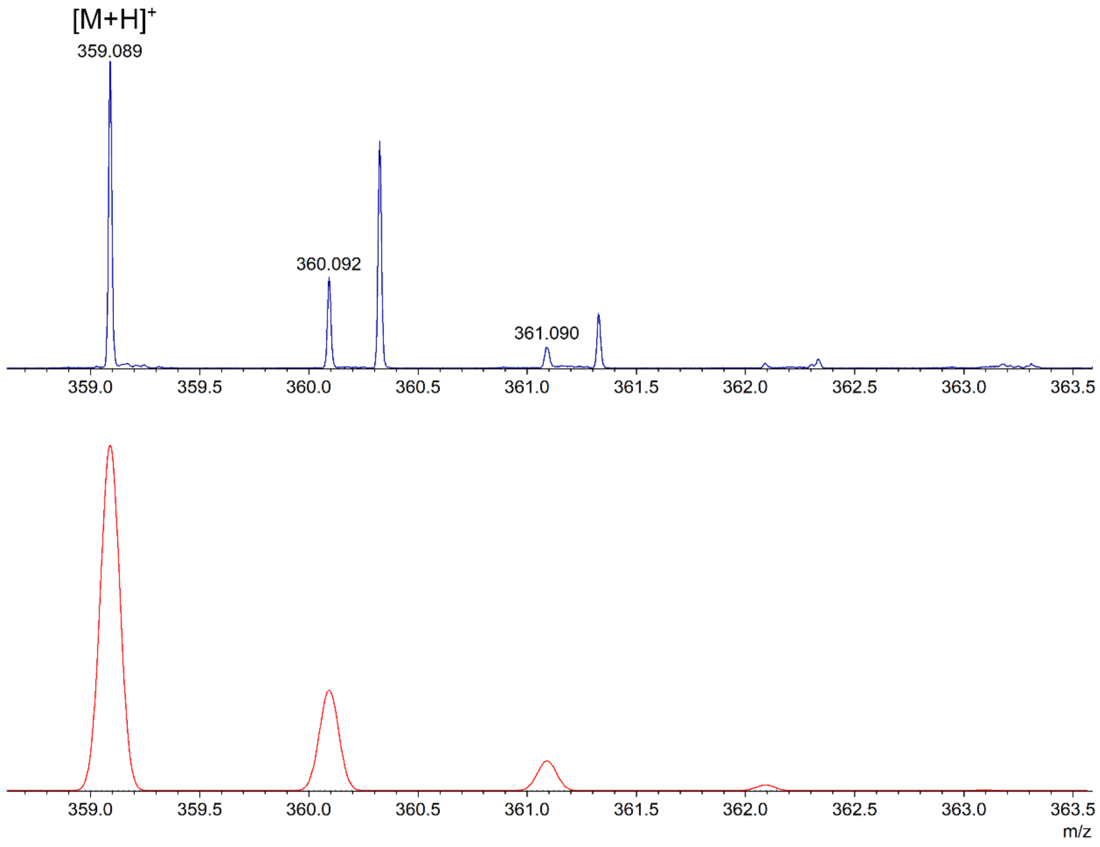

Figure S 36. HRMS (ESI-TOF), of compound **5-SH** $[M+H]^+$. Calculated (red), measured (blue).

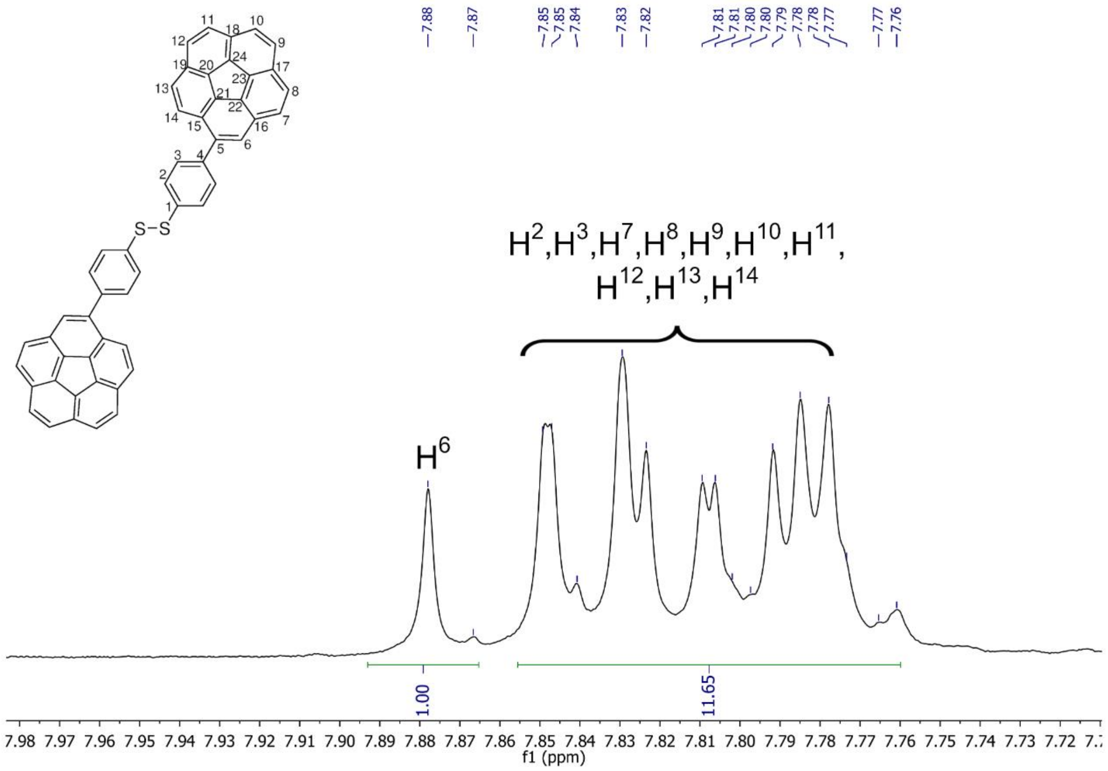

Figure S 37. ^{1}H-NMR (500 MHz, $CDCl_3$) spectrum of compound **5_2-SS**.

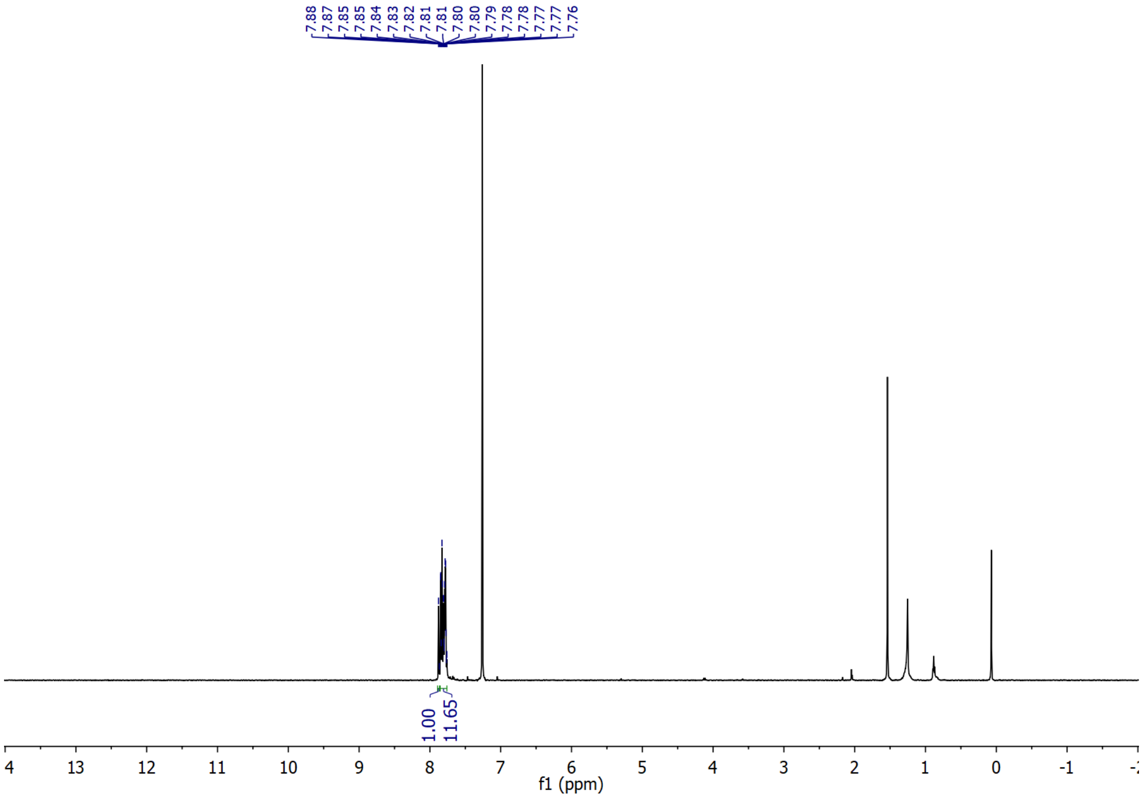

Figure S 38. Full ^{1}H-NMR (500 MHz, $CDCl_3$) spectrum of compound **5_2-SS**.

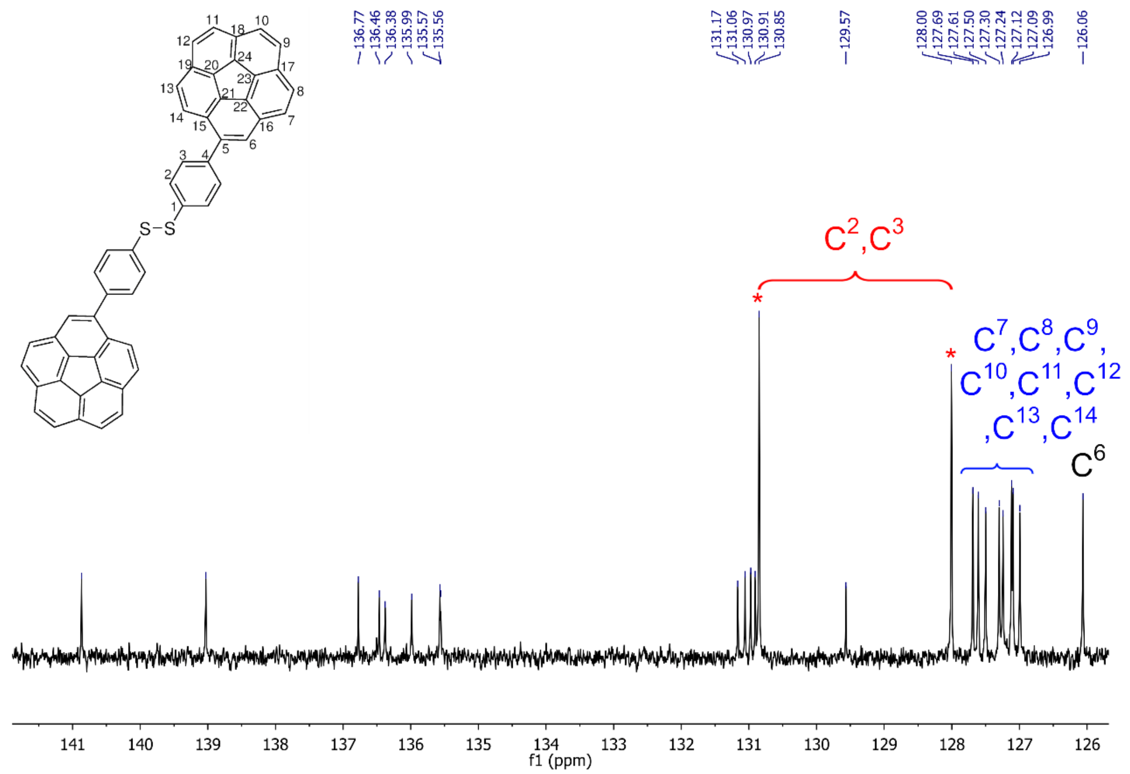

Figure S 39. $^{13}C\{^1H\}$-NMR (126 MHz, $CDCl_3$) spectrum of compound **5_2-SS**.

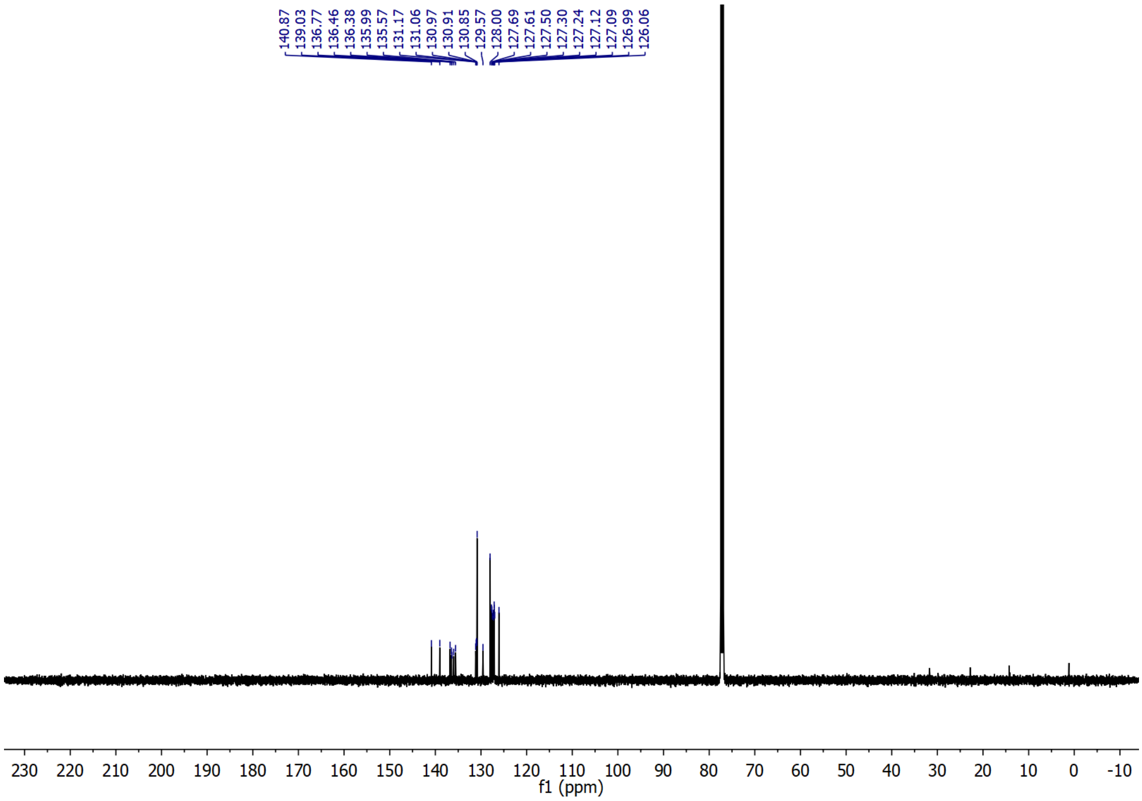

Figure S 40. Full $^{13}C\{^1H\}$-NMR (126 MHz, $CDCl_3$) spectrum of compound **5_2-SS**.

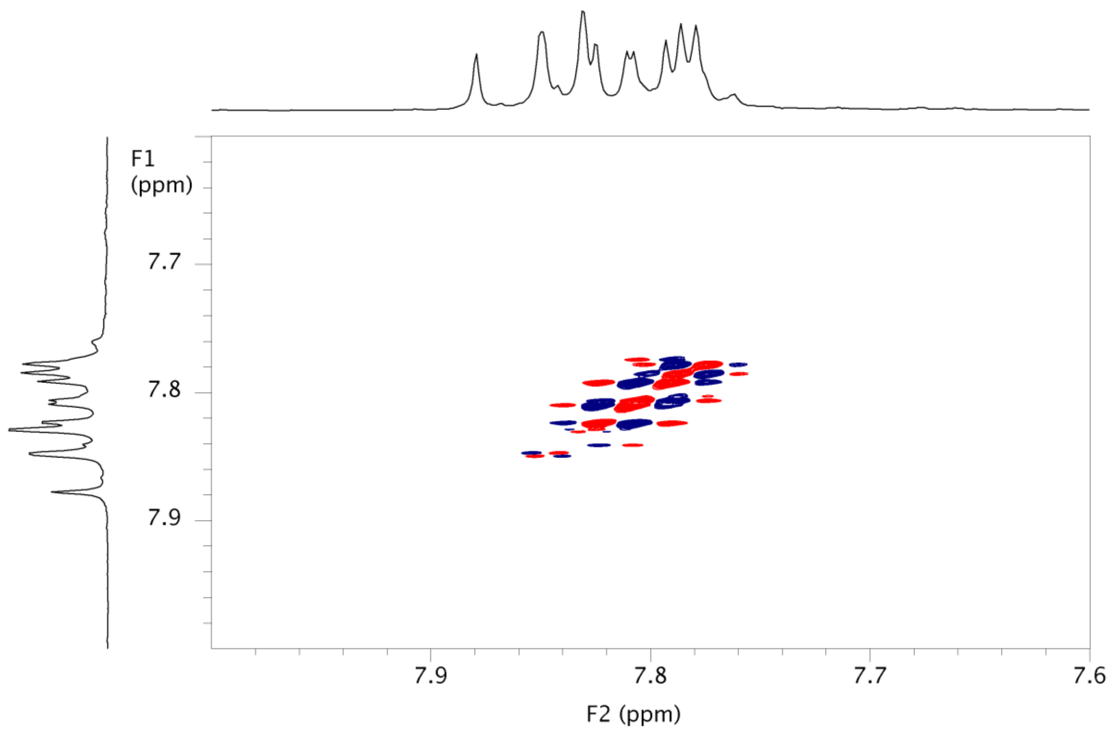

Figure S 41. ^{1}H-^{1}H gDQFCOSY (500 MHz, $CDCl_3$) spectrum of compound **5_2-SS**.

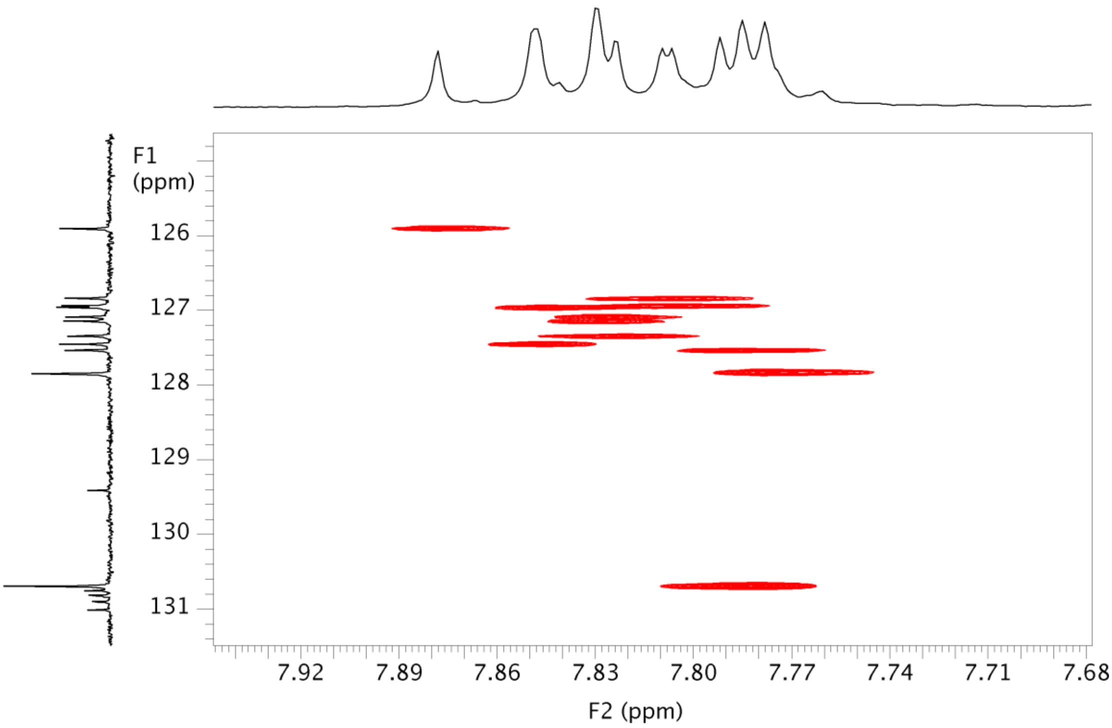

Figure S 42. ^{1}H-^{13}C bsgHSQCAD (500 MHz, $CDCl_3$) spectrum of compound **5_2-SS**.

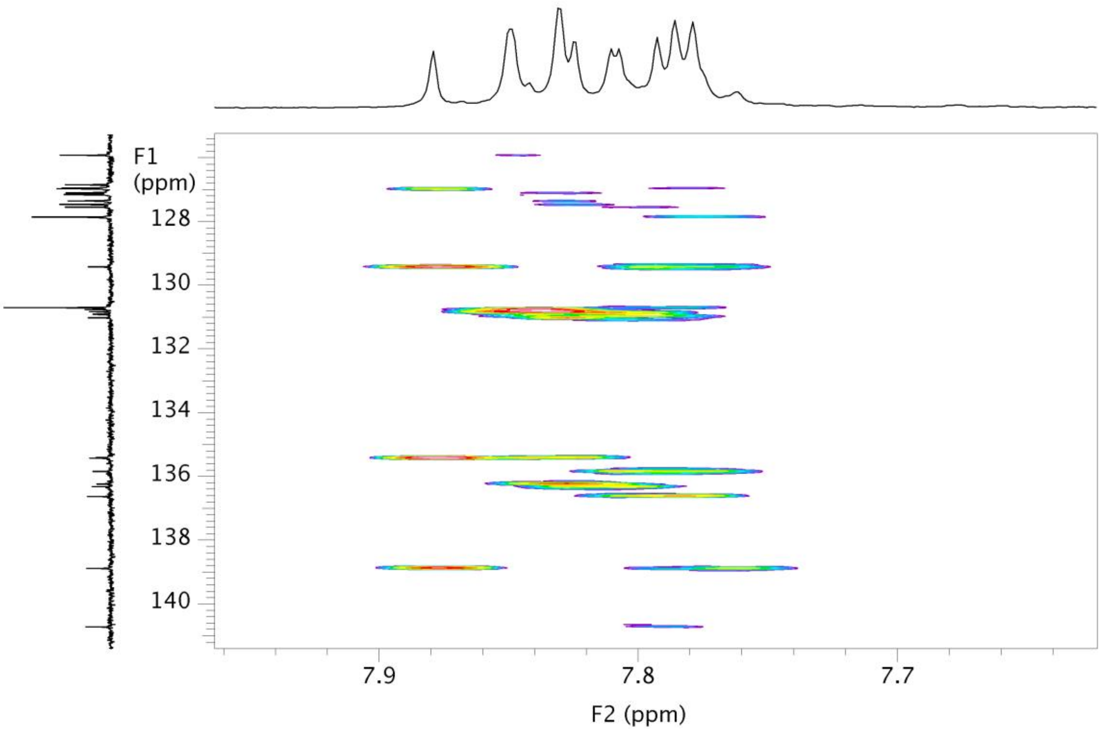

Figure S 43. ^{1}H-^{13}C bsgHMBC (500 MHz, $CDCl_3$) spectrum of compound **5_2-SS**.

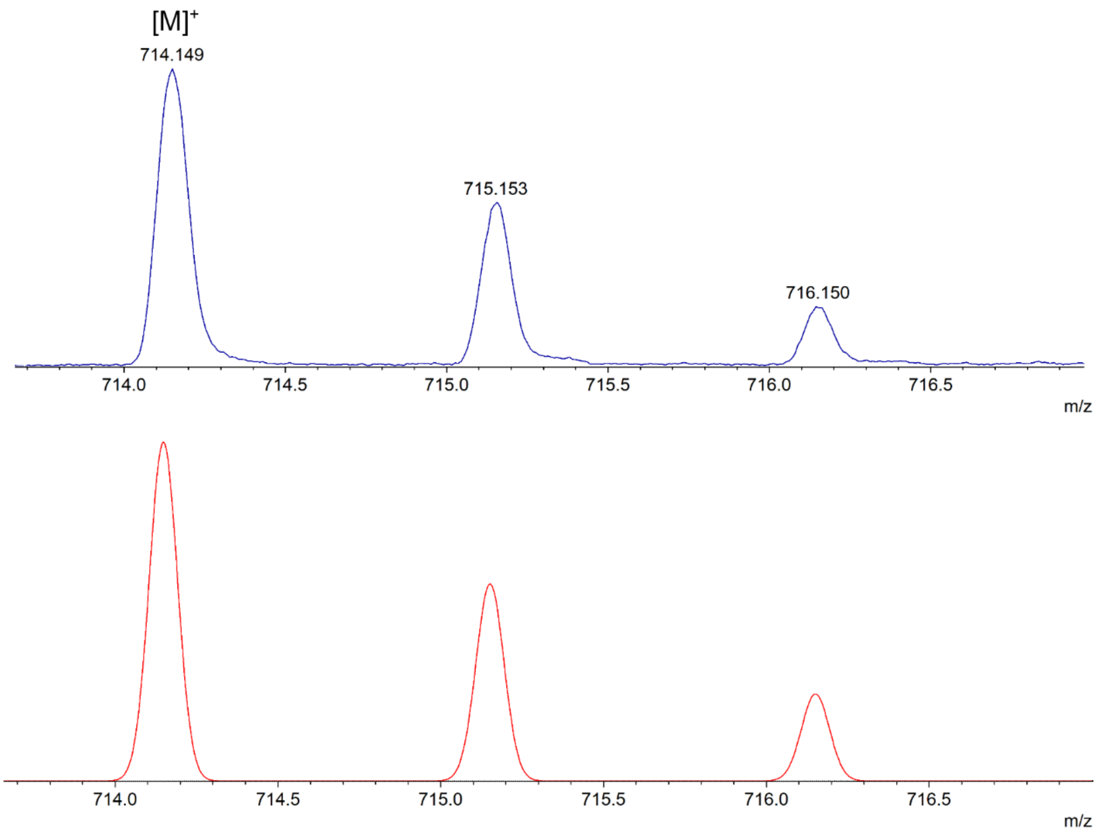

Figure S 44. HRMS (MALDI-TOF), of compound **5_2-SS** $[M]^+$. Calculated (red), measured (blue).

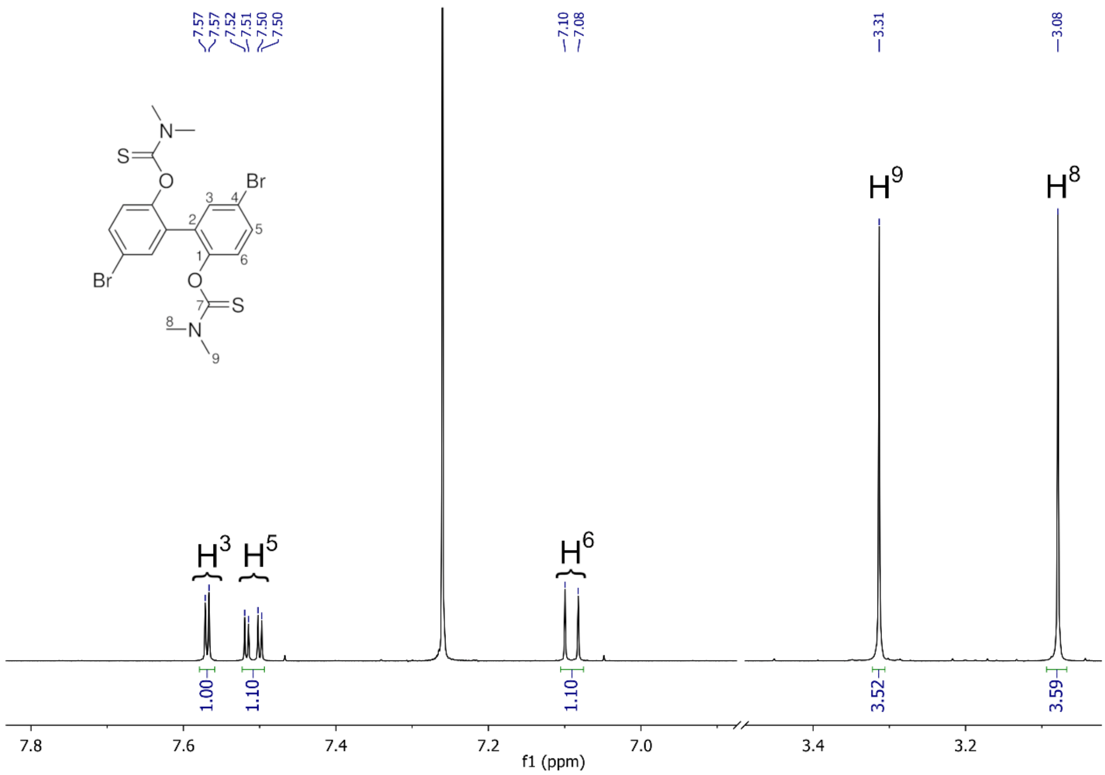

Figure S 45. ^{1}H-NMR (500 MHz, $CDCl_3$) spectrum of compound **8**.

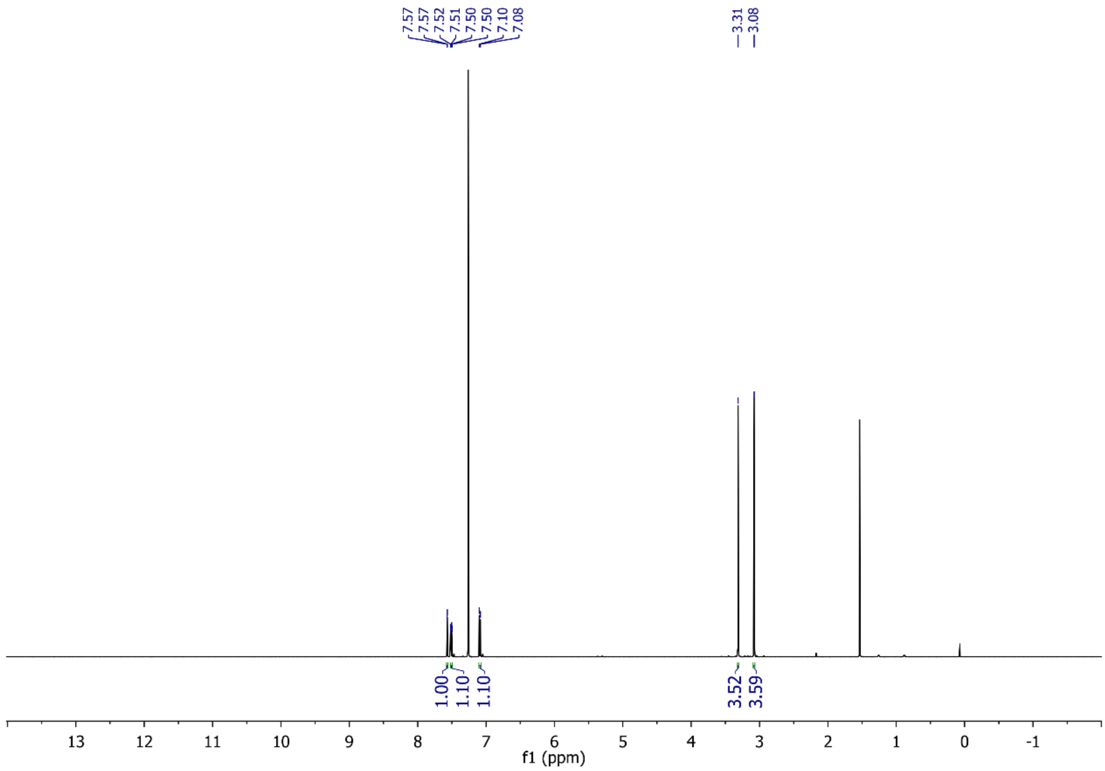

Figure S 46. Full ^{1}H-NMR (500 MHz, $CDCl_3$) spectrum of compound **8**.

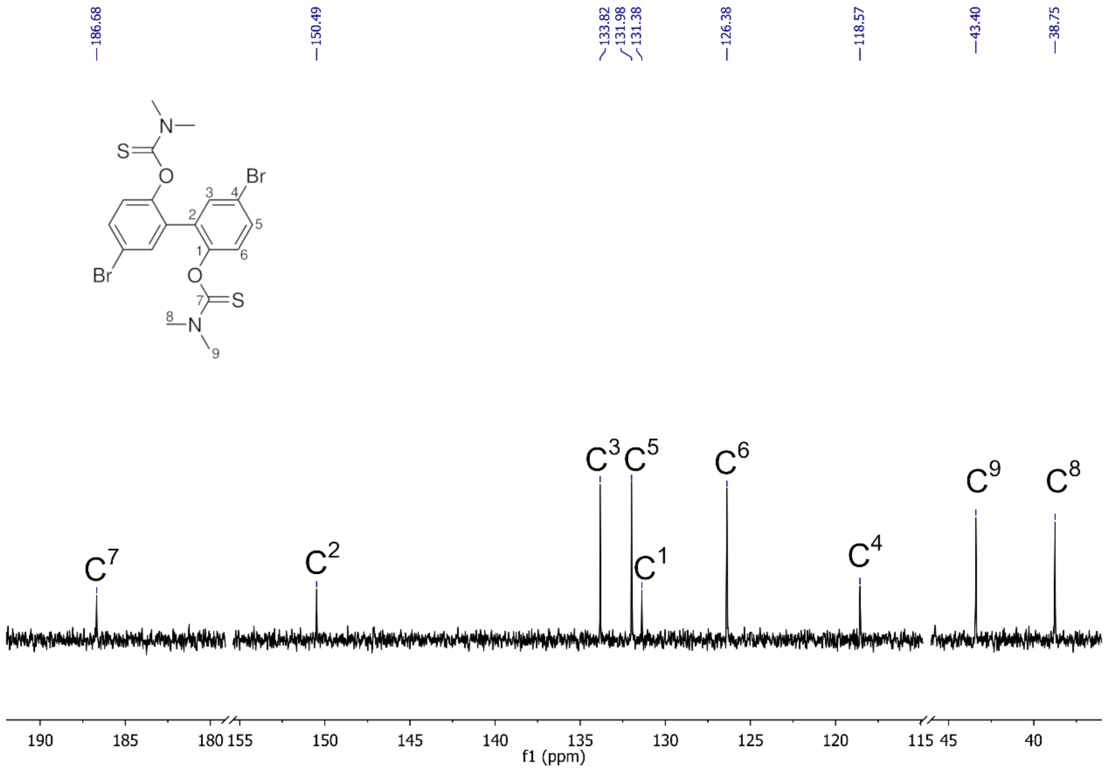

Figure S 47. $^{13}C\{^1H\}$-NMR (126 MHz, $CDCl_3$) spectrum of compound **8**.

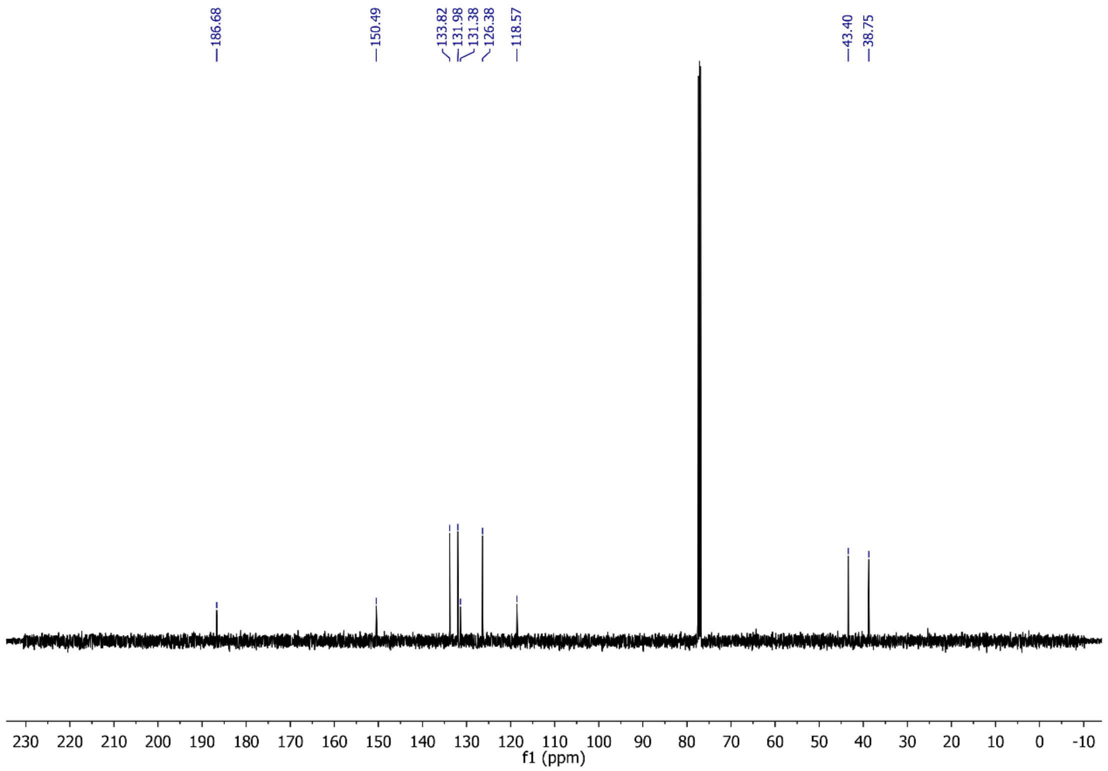

Figure S 48. Full $^{13}C\{^1H\}$-NMR (126 MHz, $CDCl_3$) spectrum of compound **8**.

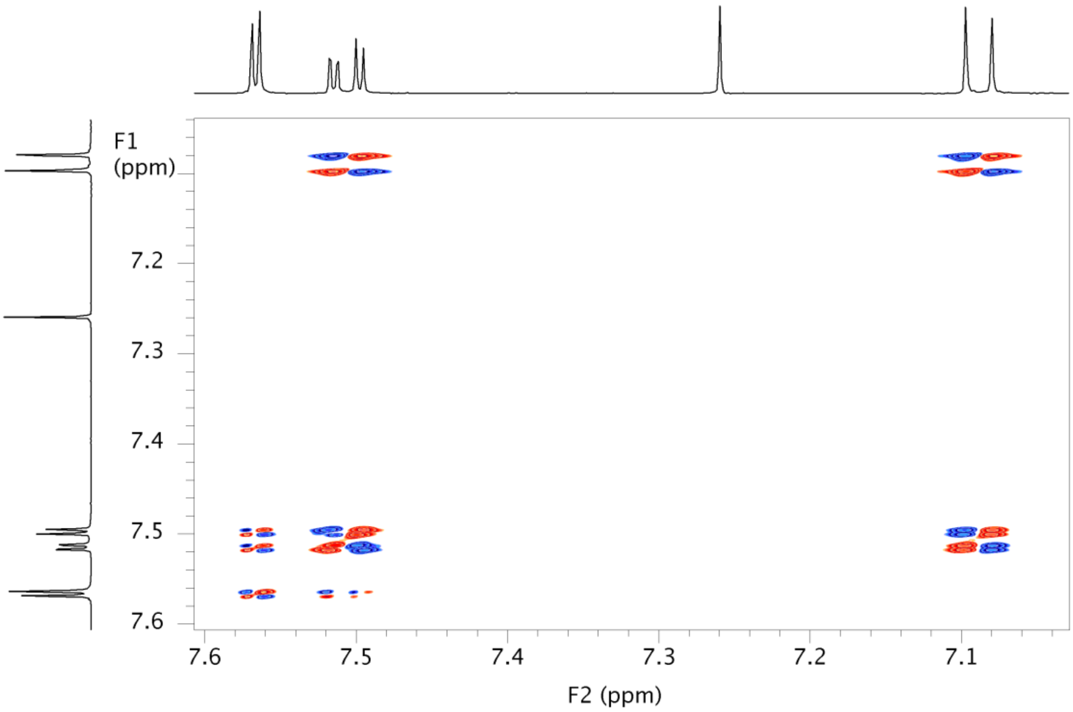

Figure S 49. ^{1}H-^{1}H gDQFCOSY (500 MHz, $CDCl_3$) spectrum of compound **8**.

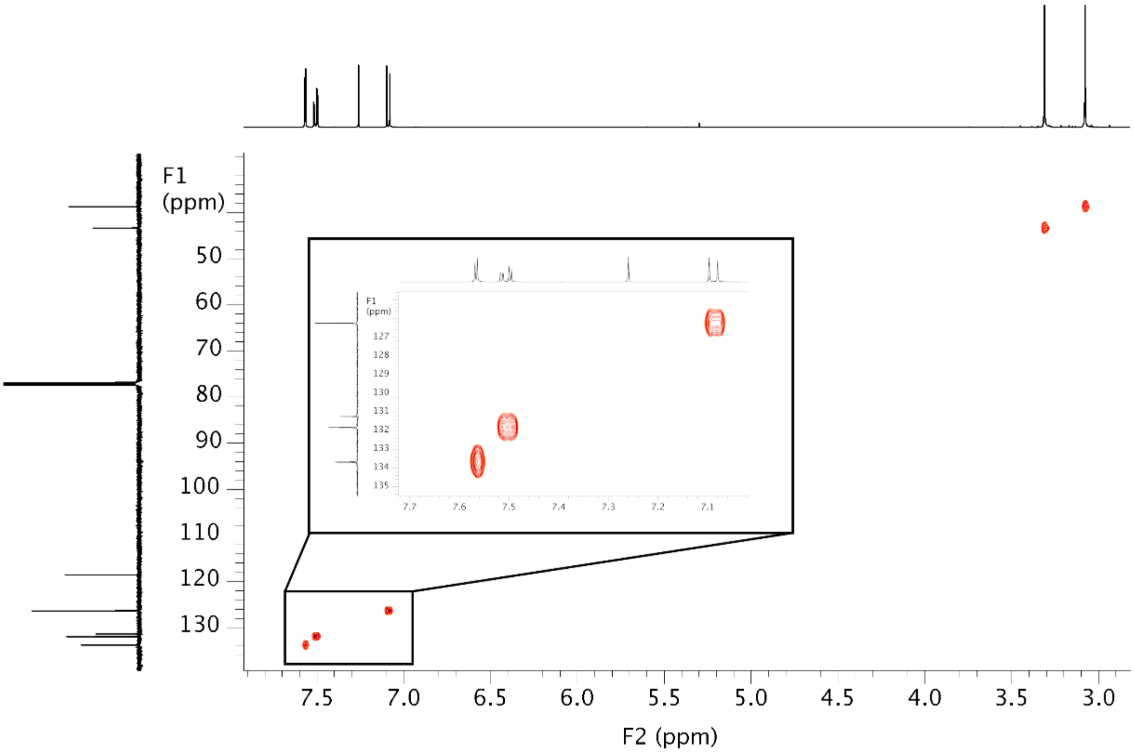

Figure S 50. ^{1}H-^{13}C gc2HSQC (500 MHz, $CDCl_3$) spectrum of compound **8**.

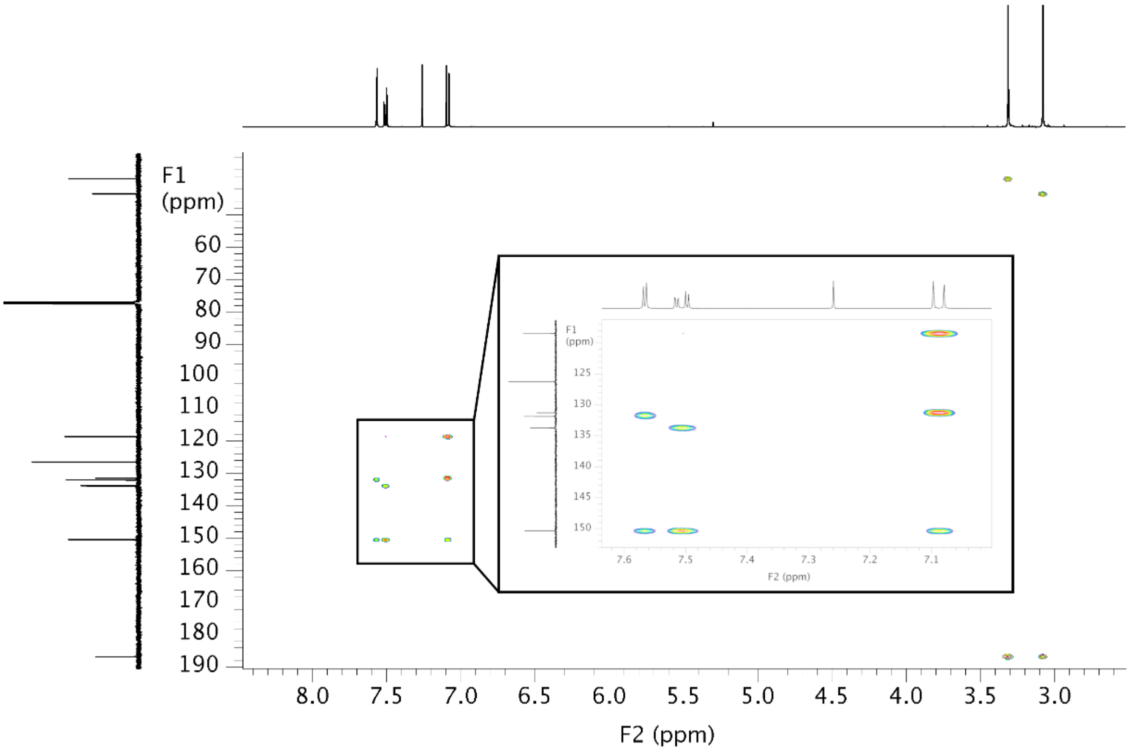

Figure S 51. ^{1}H-^{13}C gc2HMBC (500 MHz, $CDCl_3$) spectrum of compound **8**.

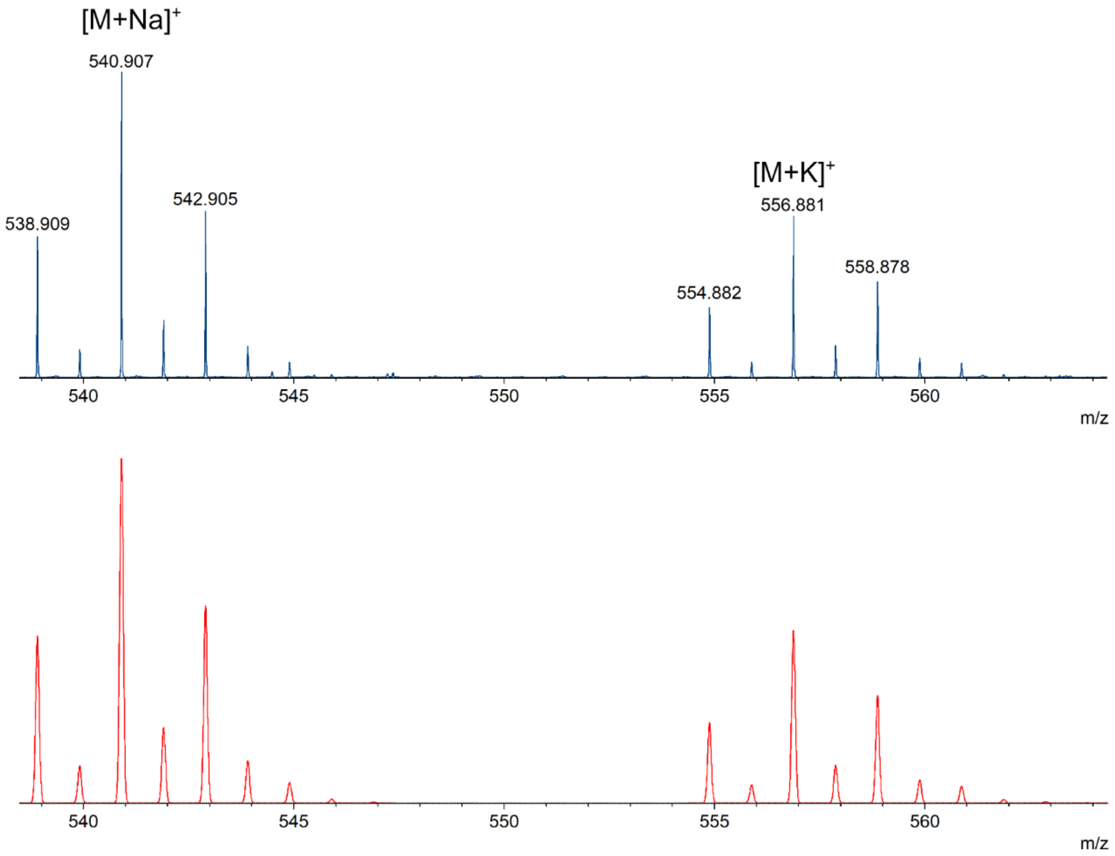

Figure S 52. HRMS (ESI-TOF), of compound **8**, $[M+Na]^+$ and $[M+K]^+$. Calculated (red), measured (blue).

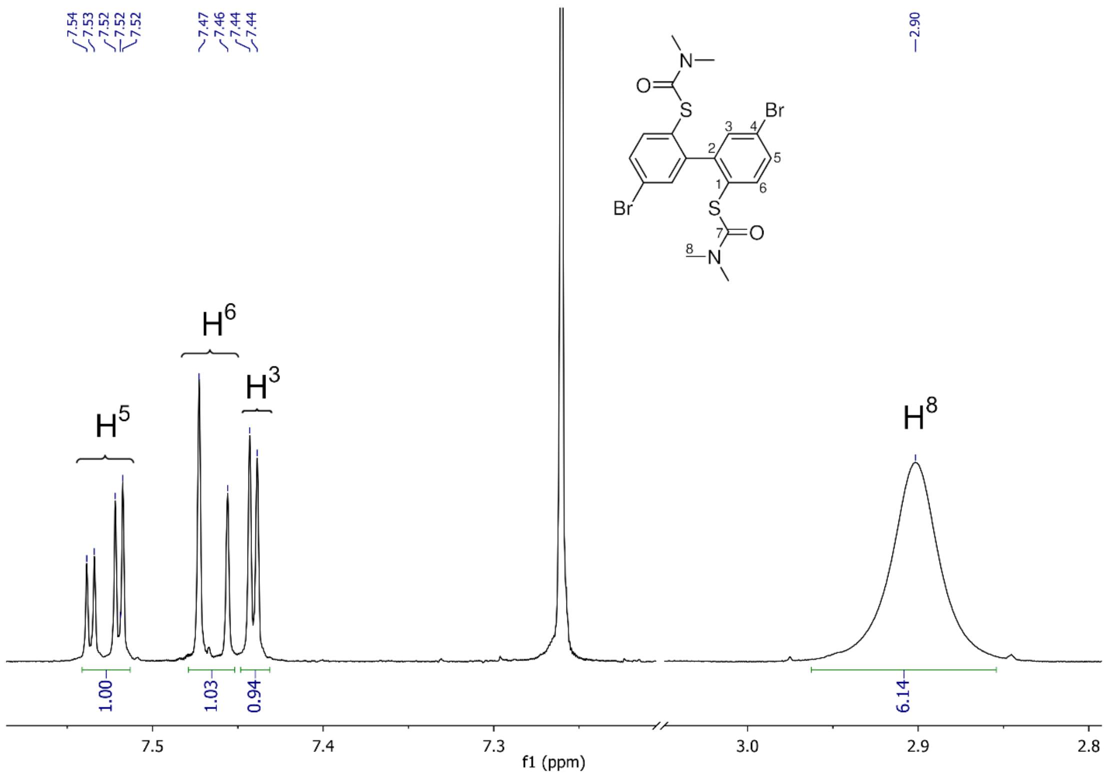

Figure S 53. ^{1}H-NMR (500 MHz, $CDCl_3$) spectrum of compound **9**.

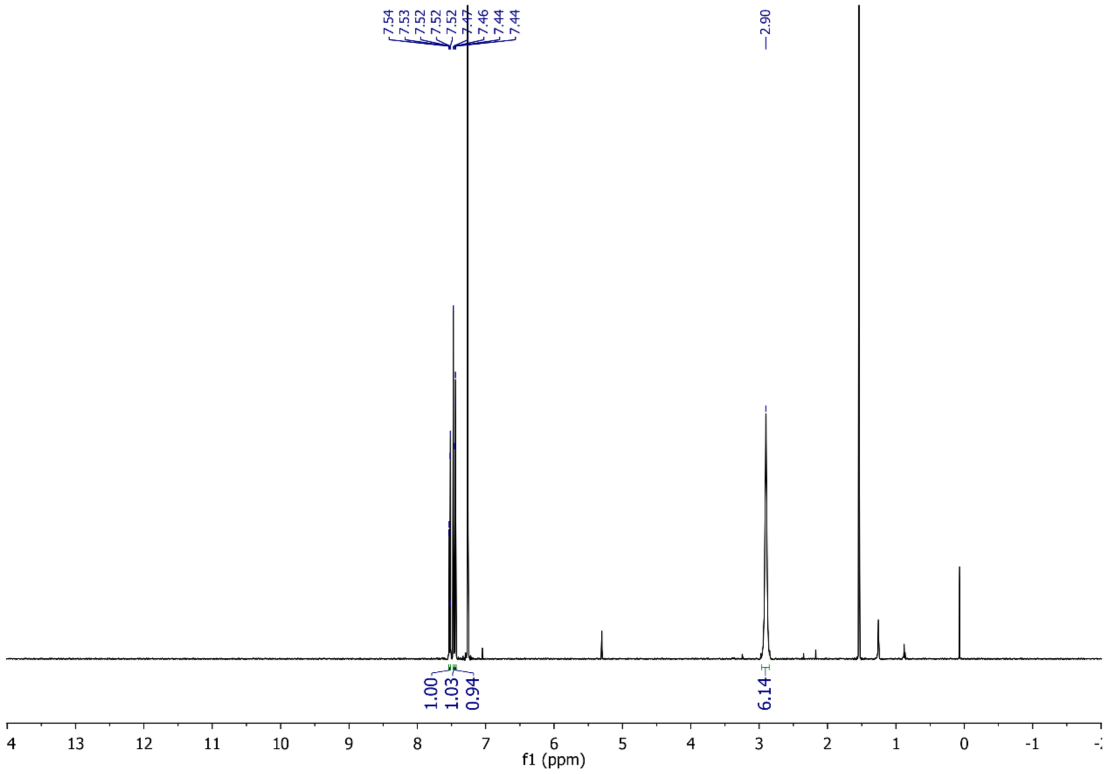

Figure S 54. Full ^{1}H-NMR (500 MHz, $CDCl_3$) spectrum of compound **9**.

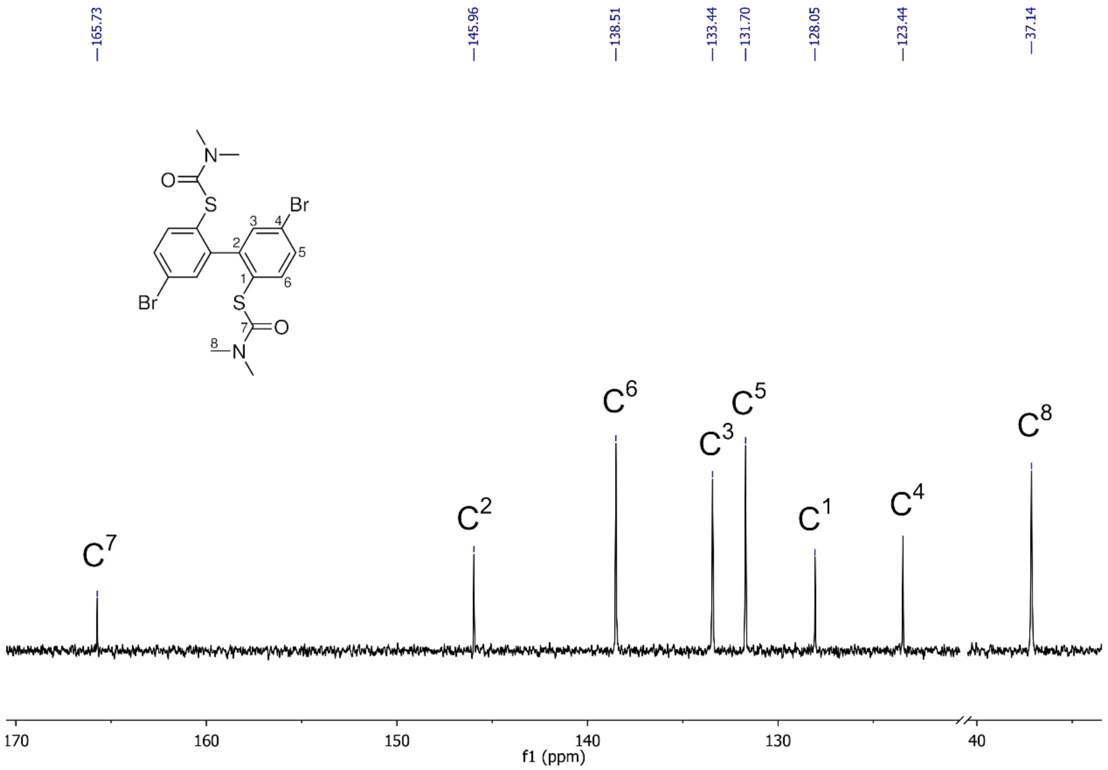

Figure S 55. $^{13}C\{^{1}H\}$-NMR (101 MHz, $CDCl_3$) spectrum of compound **9**.

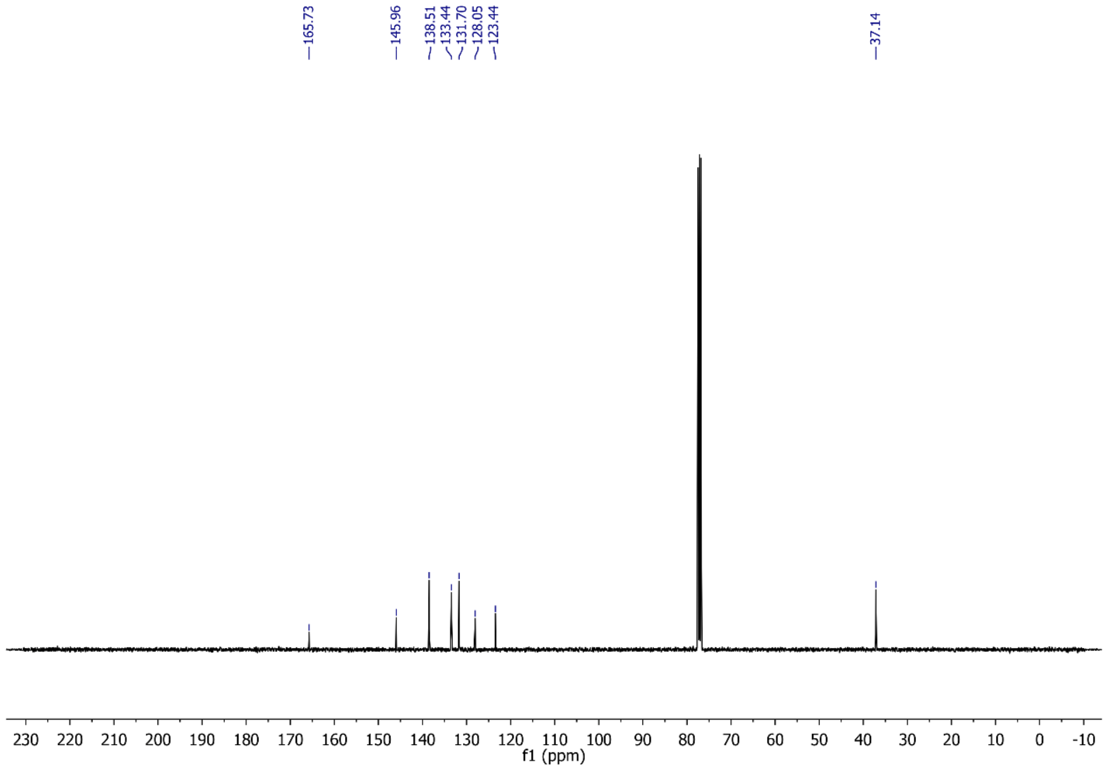

Figure S 56. Full $^{13}C\{^{1}H\}$-NMR (101 MHz, $CDCl_3$) spectrum of compound **9**.

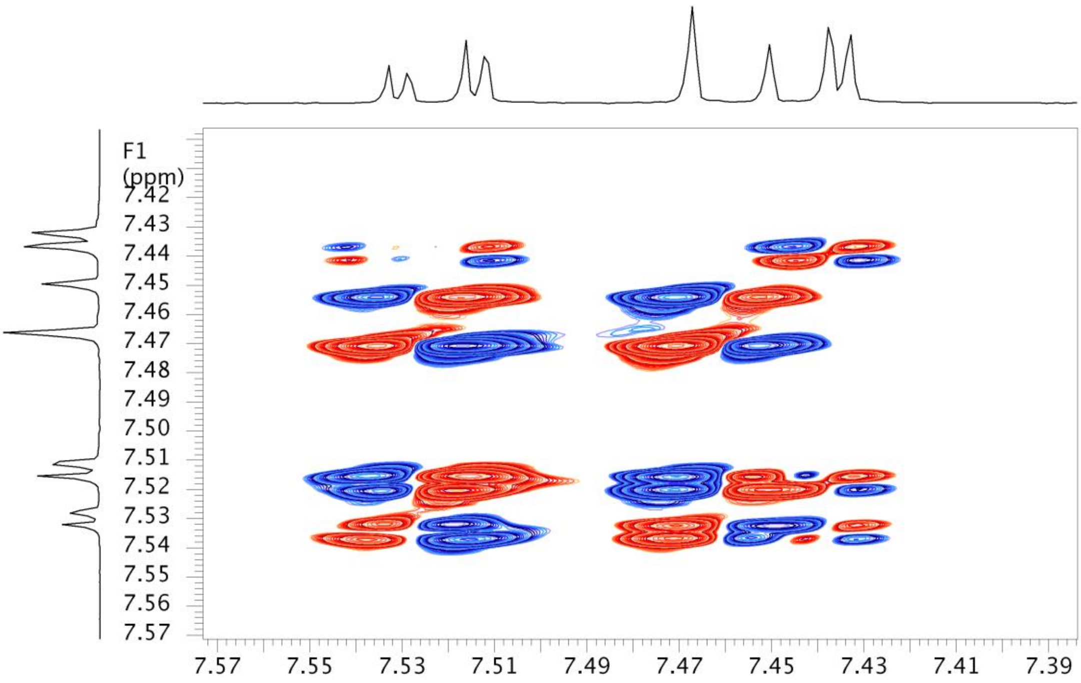

Figure S 57. ^{1}H-^{1}H gDQFCOSY (500 MHz, $CDCl_3$) spectrum of compound **9**.

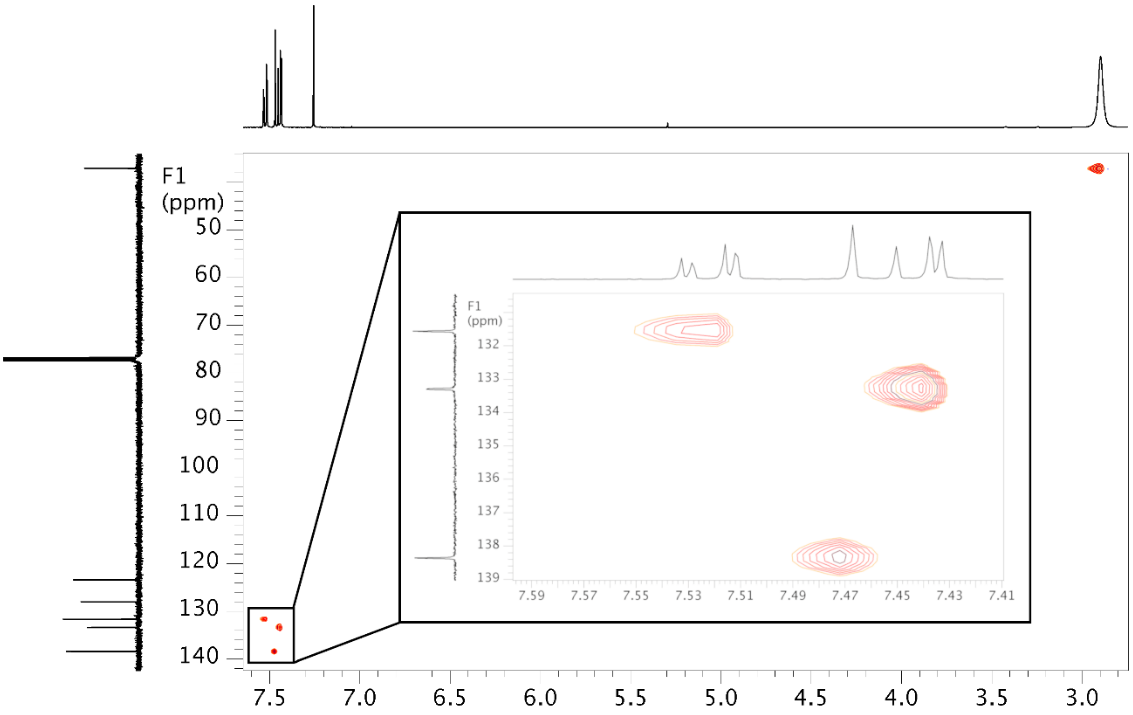

Figure S 58. ^{1}H-^{13}C gc2HSQC (500 MHz, $CDCl_3$) spectrum of compound **9**.

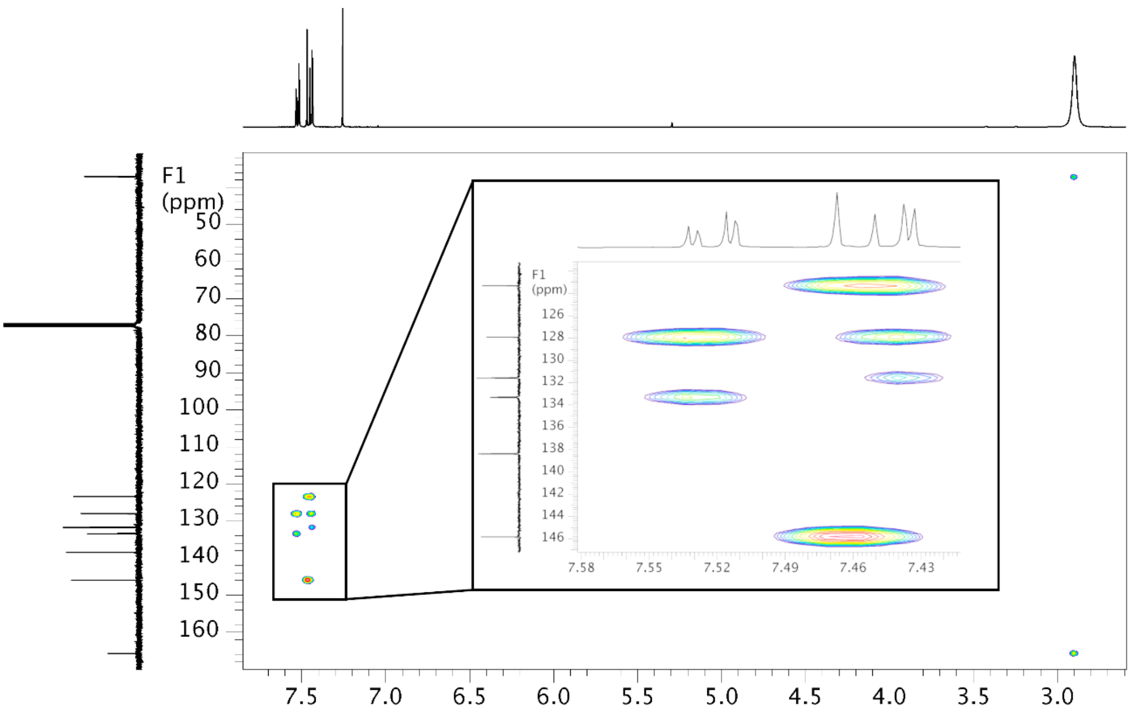

Figure S 59. ^{1}H-^{13}C gc2HMBC (500 MHz, $CDCl_3$) spectrum of compound **9**.

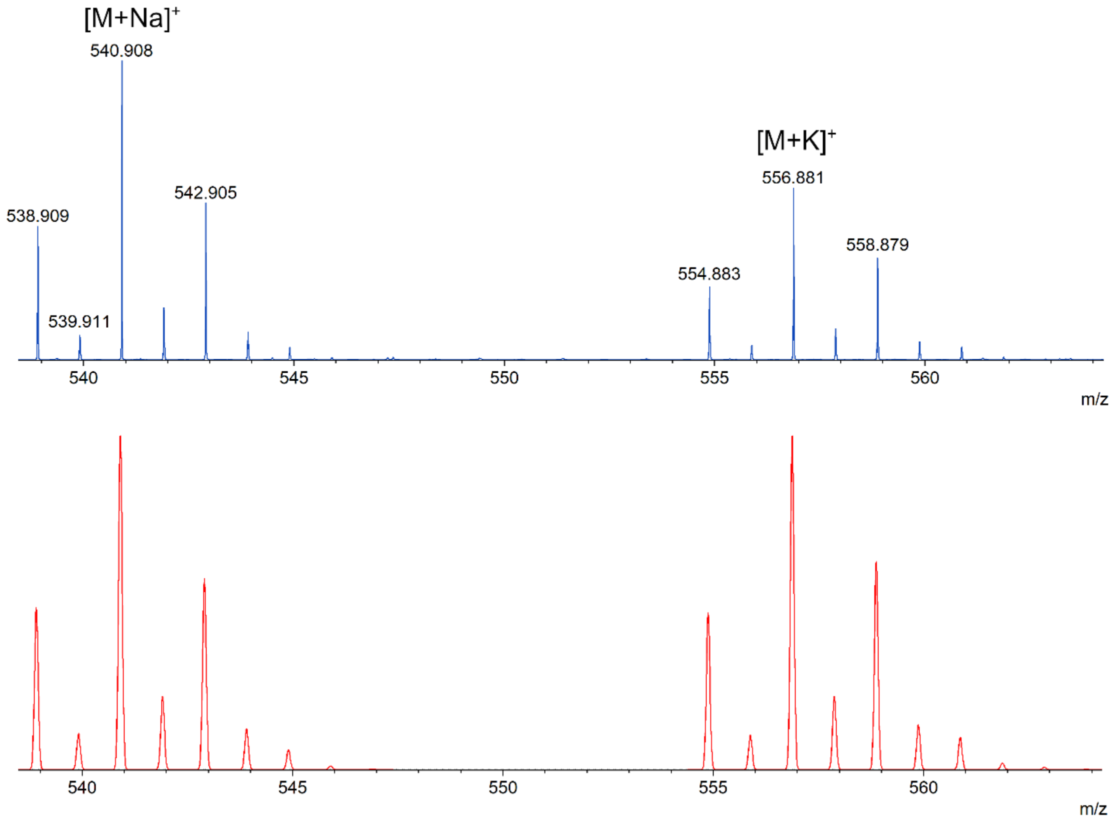

Figure S 60. HRMS (ESI-TOF), of compound **9**, $[M+Na]^+$ and $[M+K]^+$. Calculated (red), measured (blue).

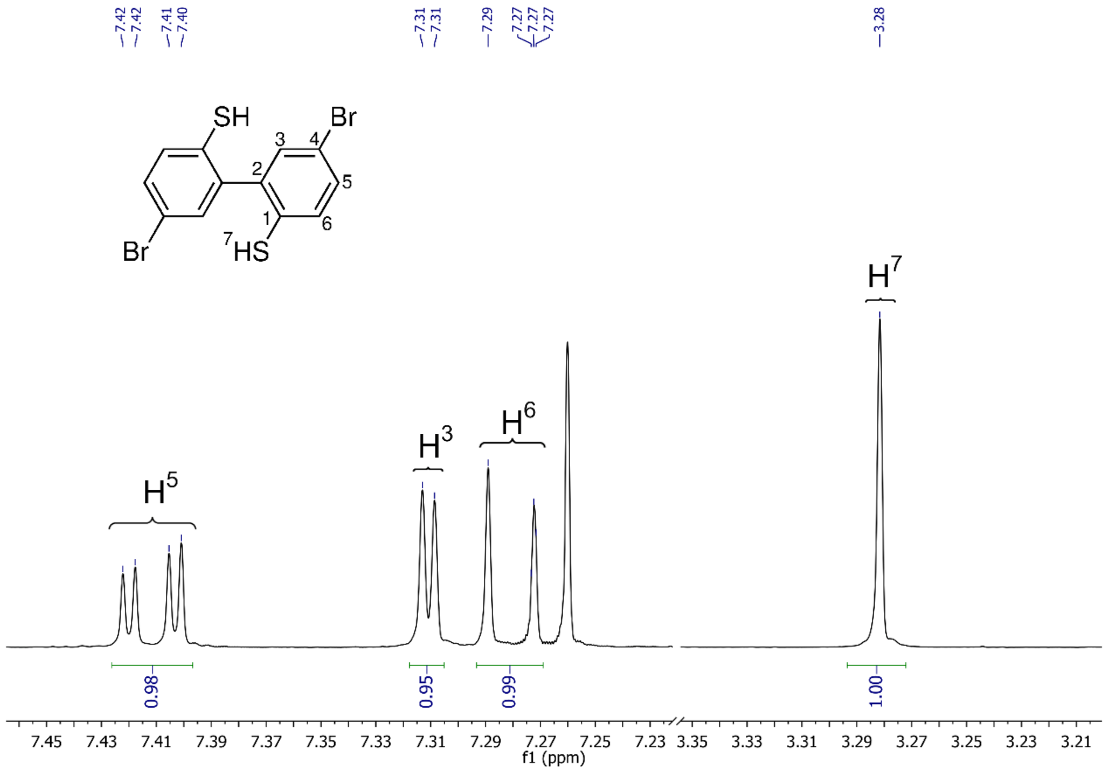

Figure S 61. ^{1}H-NMR (500 MHz, $CDCl_3$) spectrum of compound **10-SH**.

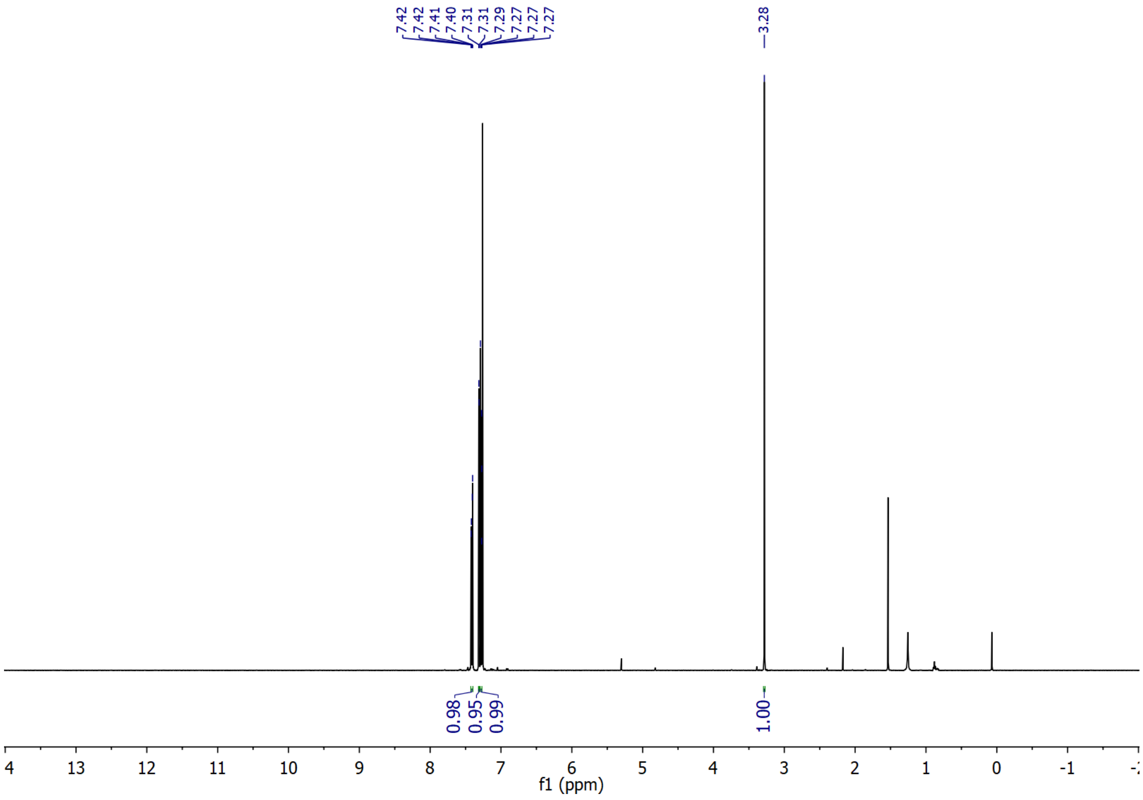

Figure S 62. Full ^{1}H-NMR (500 MHz, $CDCl_3$) spectrum of compound **10-SH**.

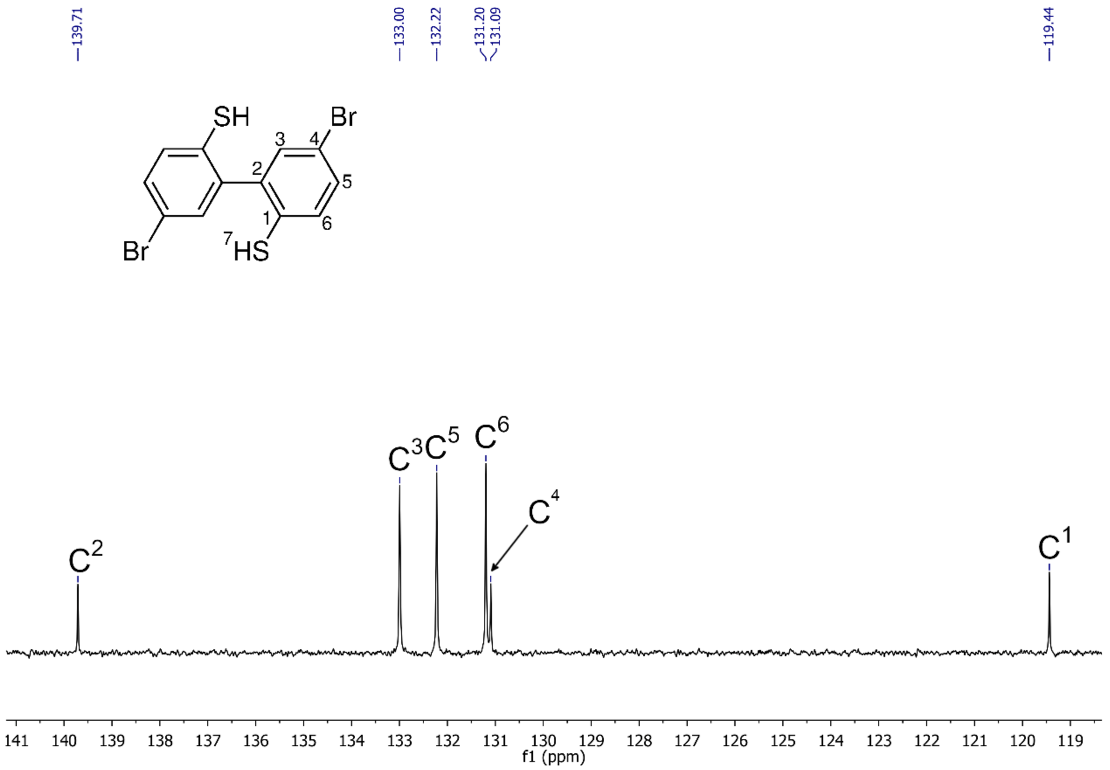

Figure S 63. $^{13}C\{^1H\}$-NMR (126 MHz, $CDCl_3$) spectrum of compound **10-SH**.

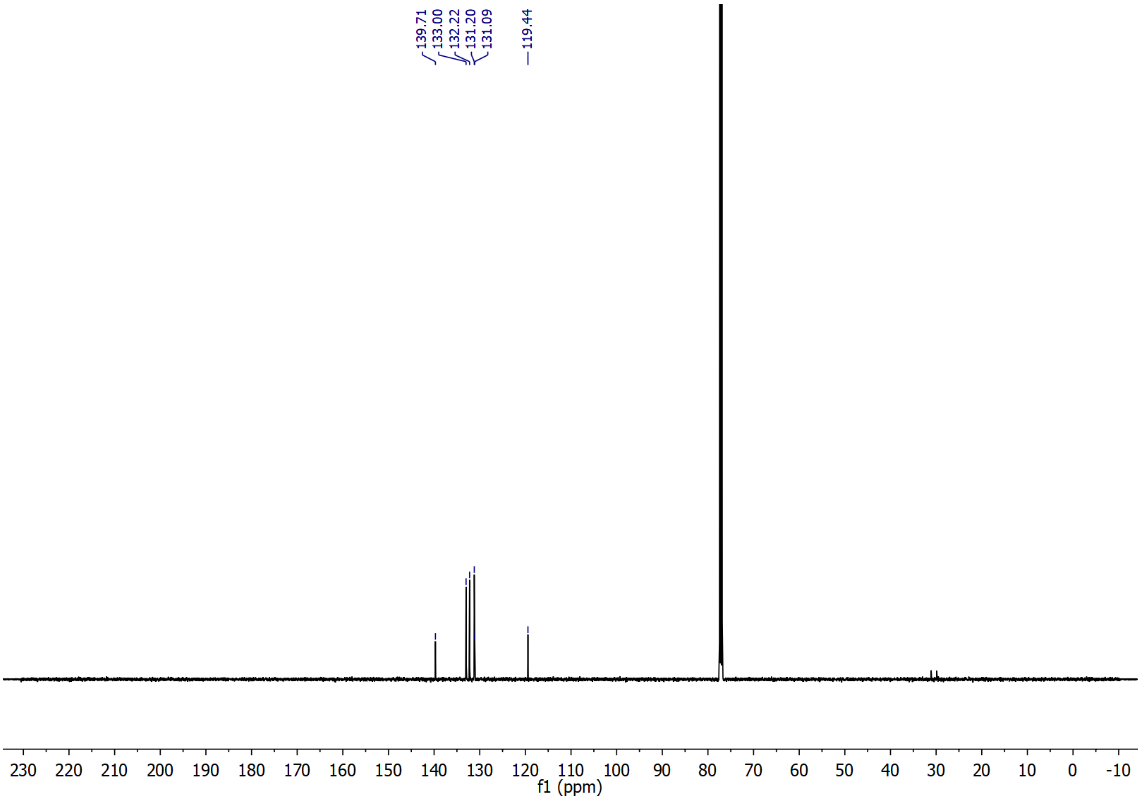

Figure S 64. Full $^{13}C\{^1H\}$-NMR (126 MHz, $CDCl_3$) spectrum of compound **10-SH**.

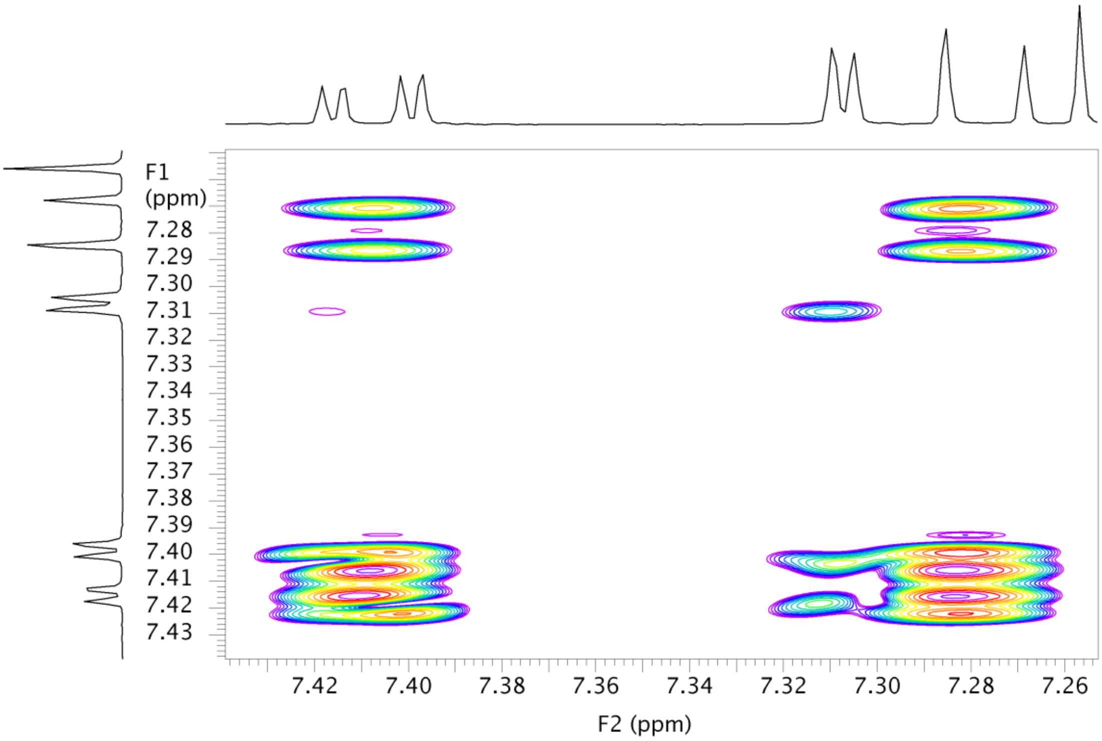

Figure S 65. ^{1}H-^{1}H gCOSY (500 MHz, $CDCl_3$) spectrum of compound **10-SH**.

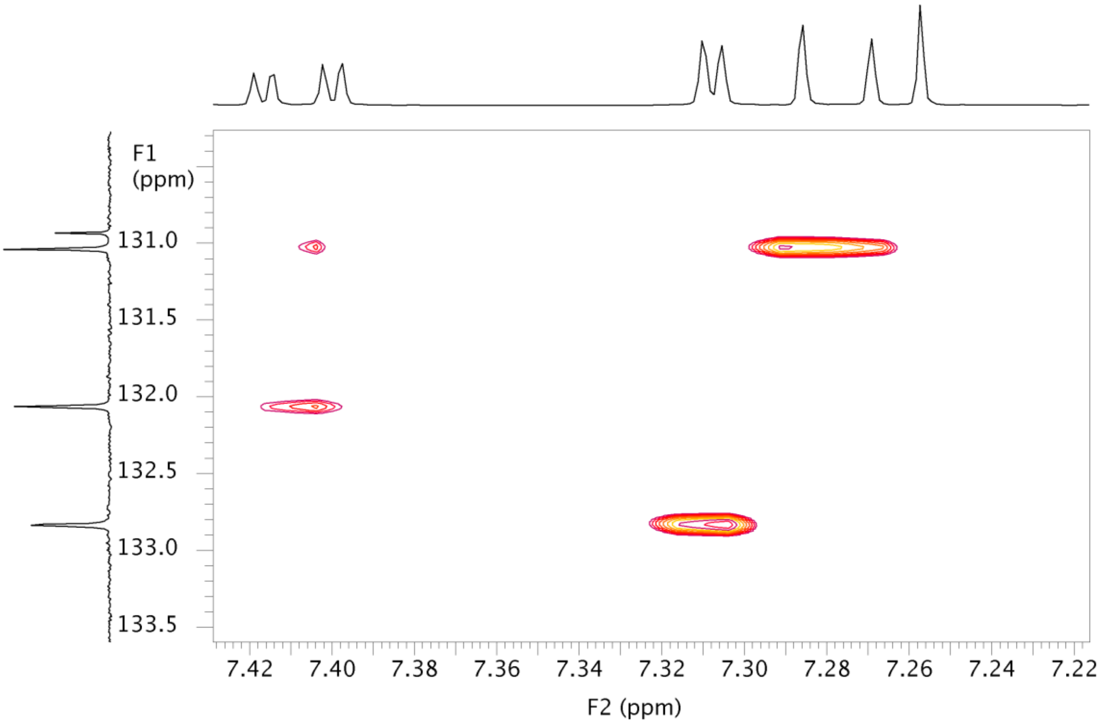

Figure S 66. ^{1}H-^{13}C bsgHSQCAD (500 MHz, $CDCl_3$) spectrum of compound **10-SH**.

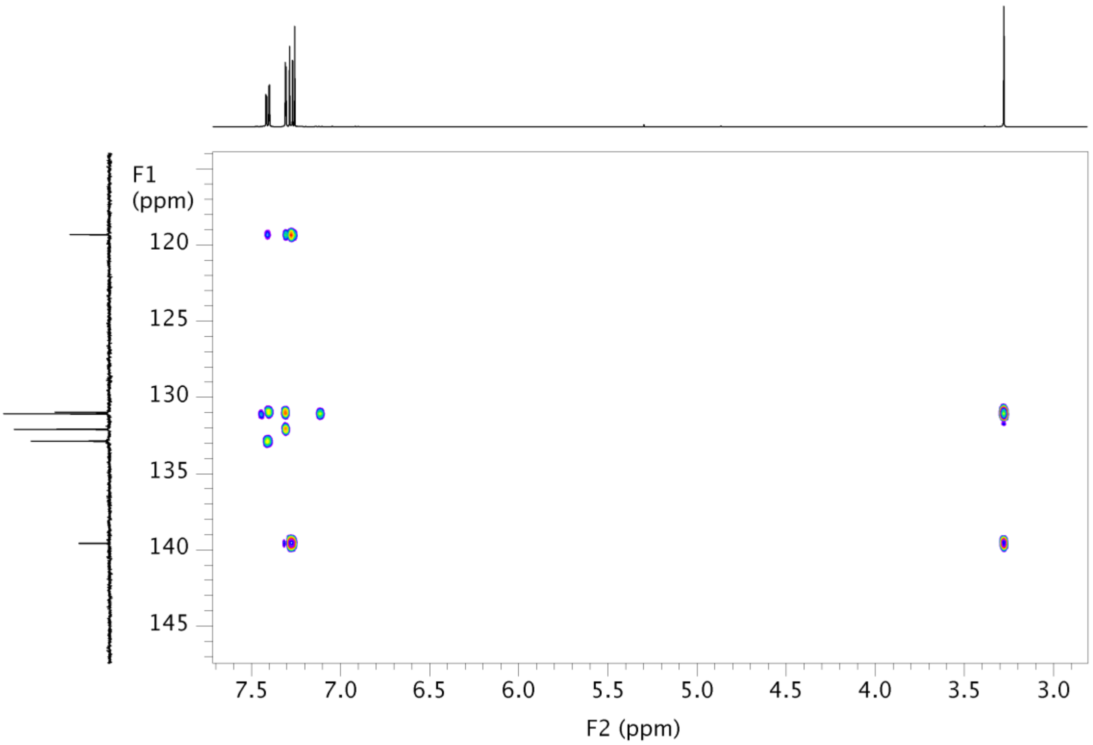

Figure S 67. ^{1}H-^{13}C gc2HMBC (500 MHz, $CDCl_3$) spectrum of compound **10-SH**.

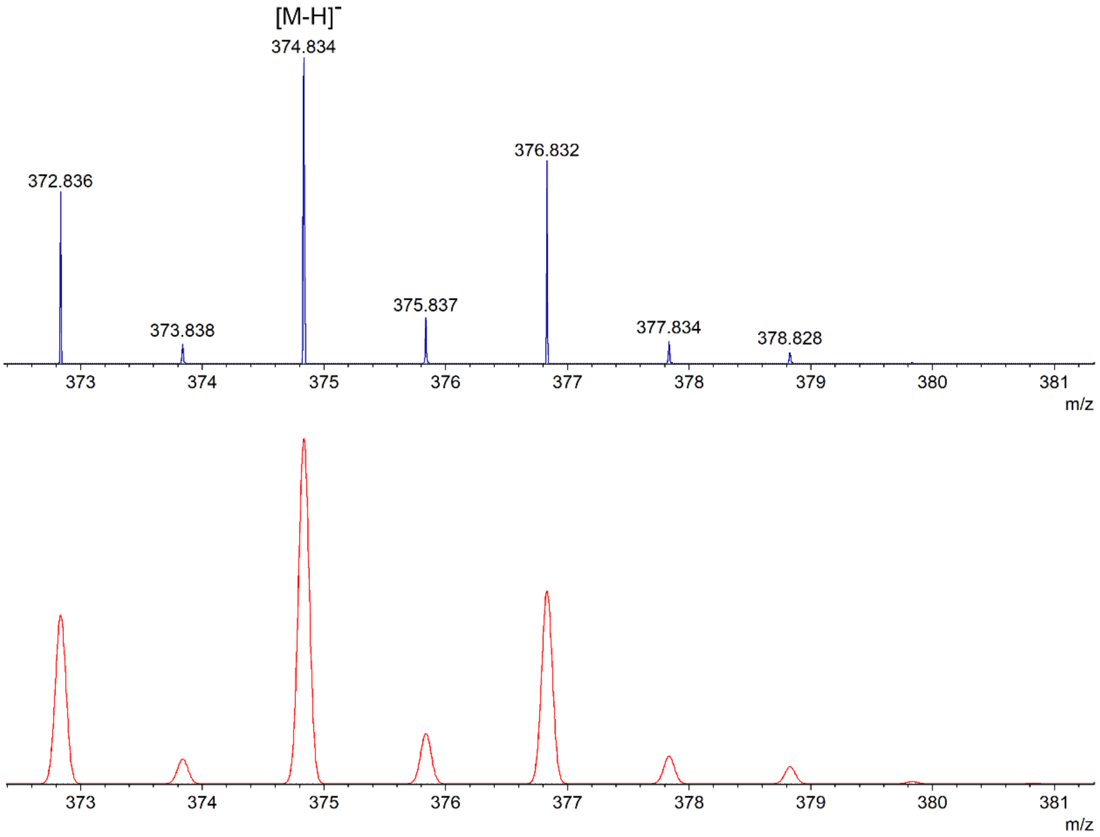

Figure S 68. HRMS (ESI-TOF), of compound **10-SH** $[M-H]^-$. Calculated (red), measured (blue).

.

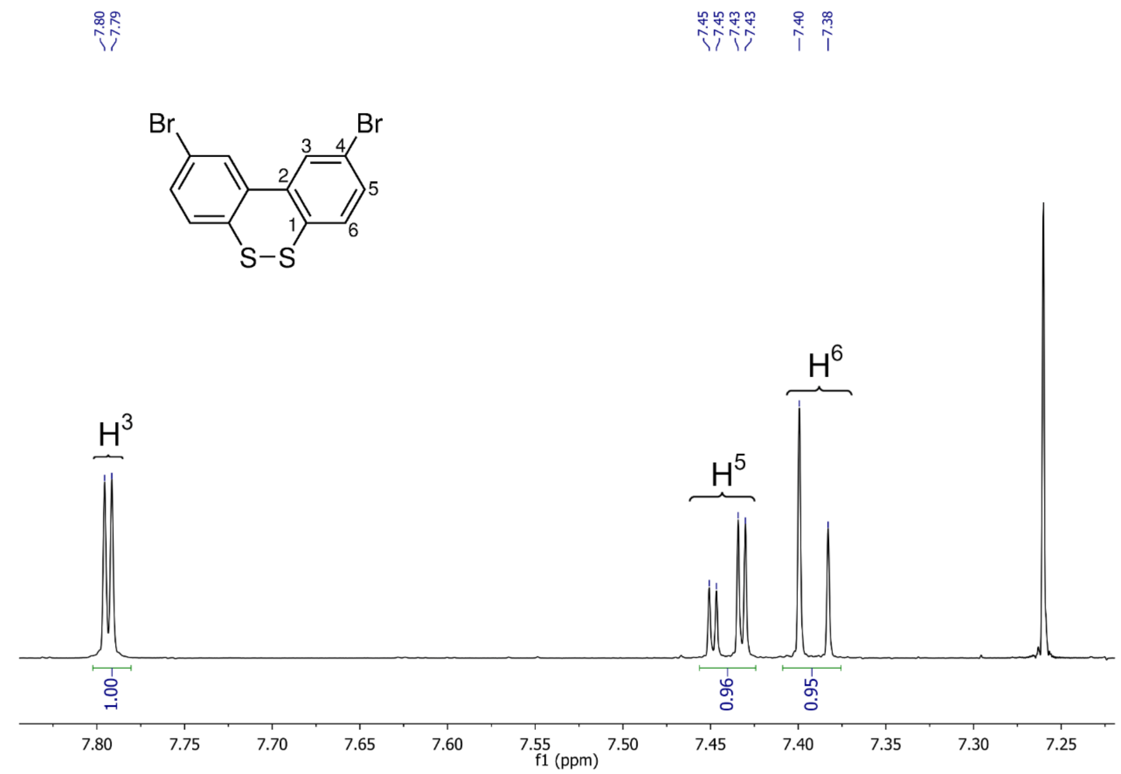

Figure S 69. ^{1}H-NMR (500 MHz, $CDCl_3$) spectrum of compound **10-SS**.

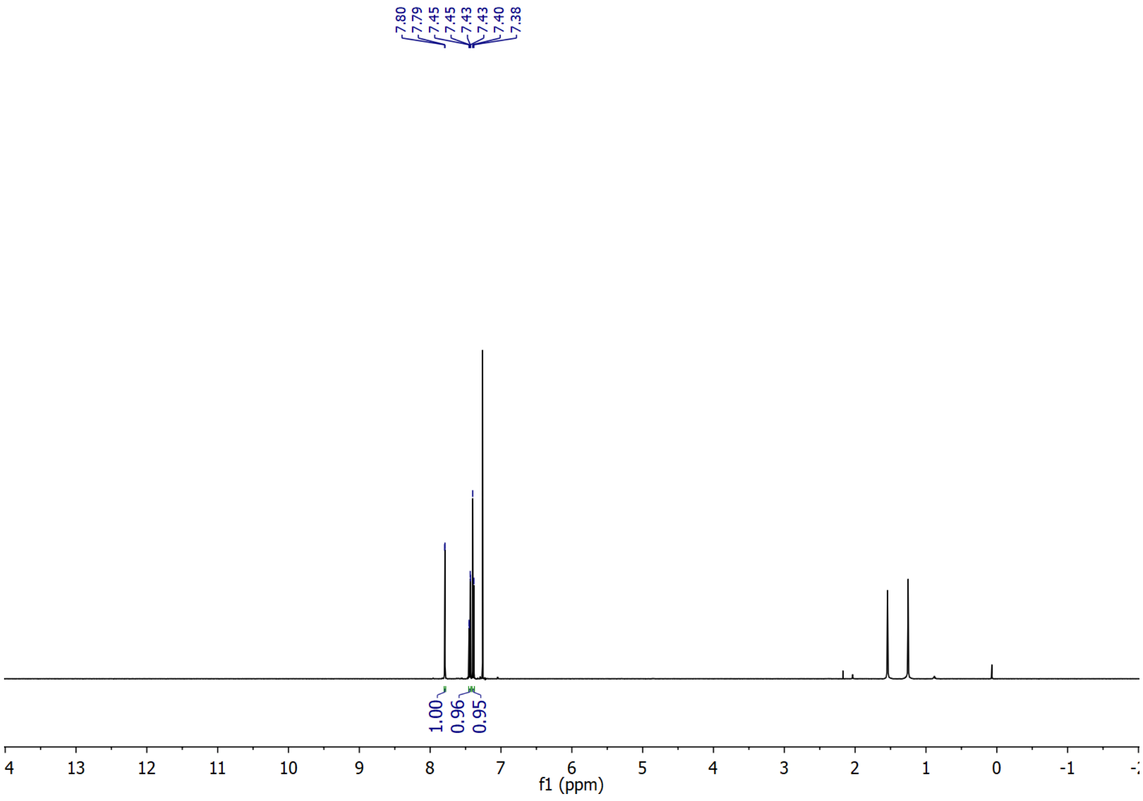

Figure S 70. Full ^{1}H-NMR (500 MHz, $CDCl_3$) spectrum of compound **10-SS**.

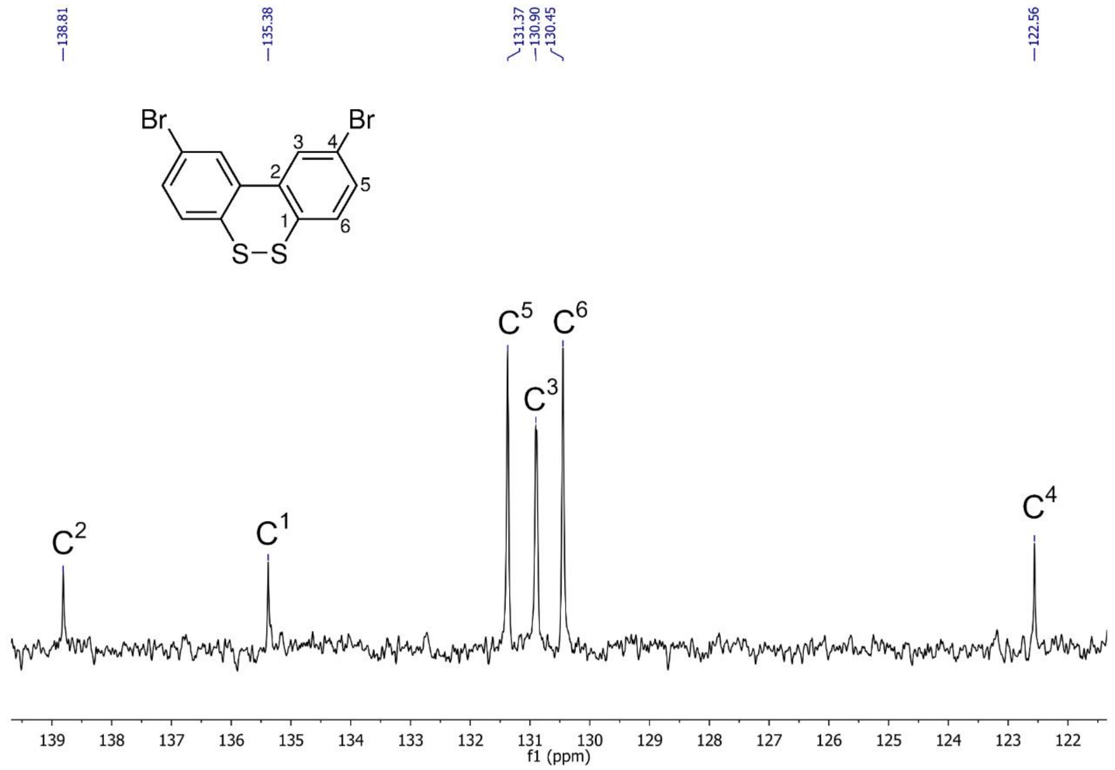

Figure S 71. $^{13}C\{^{1}H\}$-NMR (101 MHz, $CDCl_3$) spectrum of compound **10-SS**.

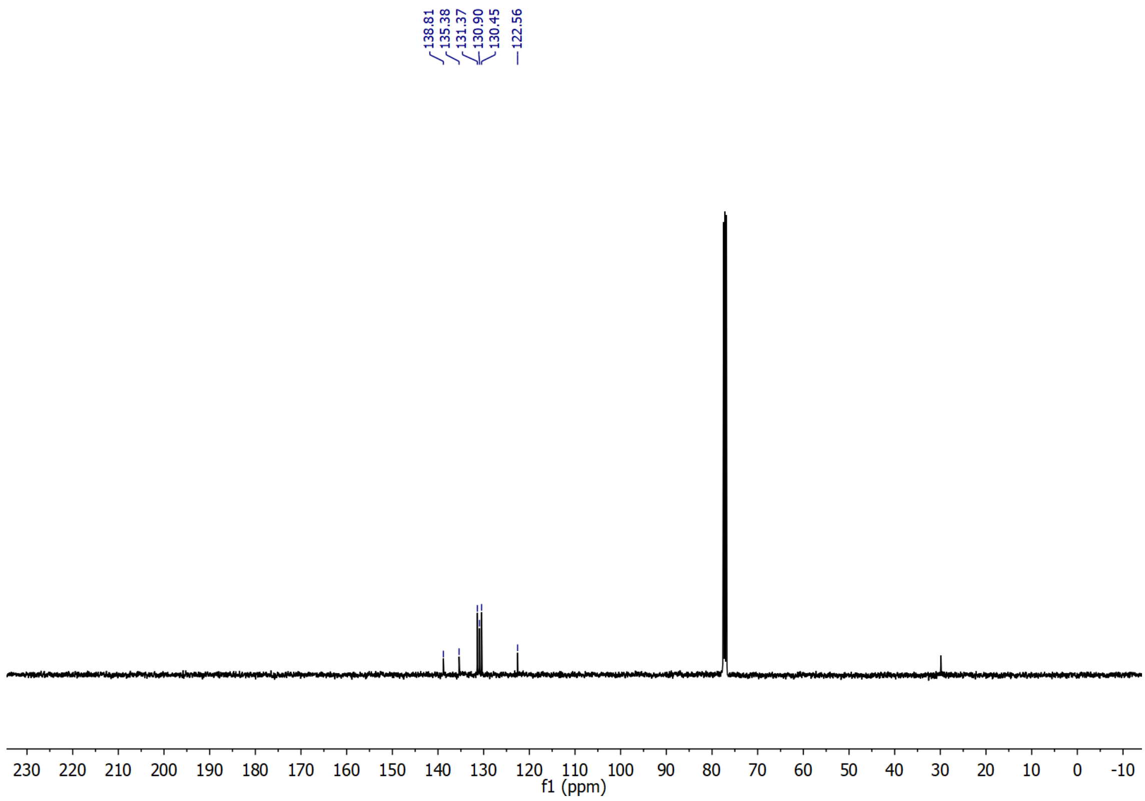

Figure S 72. Full $^{13}C\{^{1}H\}$-NMR (101 MHz, $CDCl_3$) spectrum of compound **10-SS**.

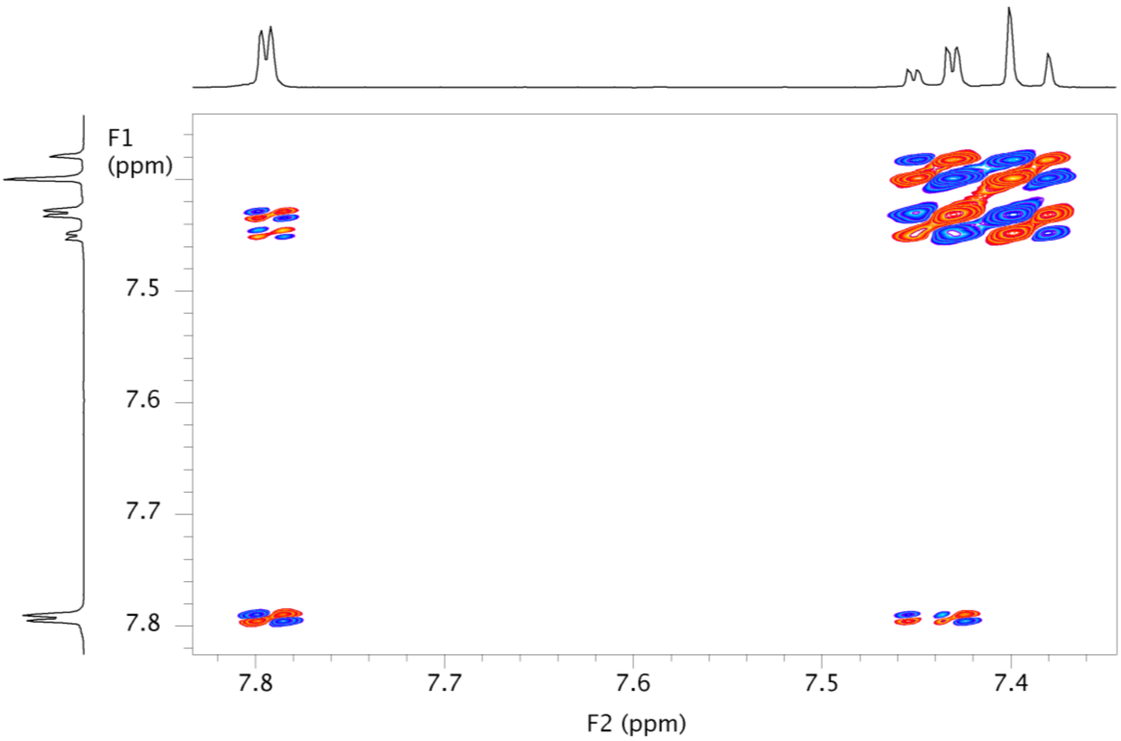

Figure S 73. ^{1}H-^{1}H gDQFCOSY (500 MHz, $CDCl_3$) spectrum of compound **10-SS**.

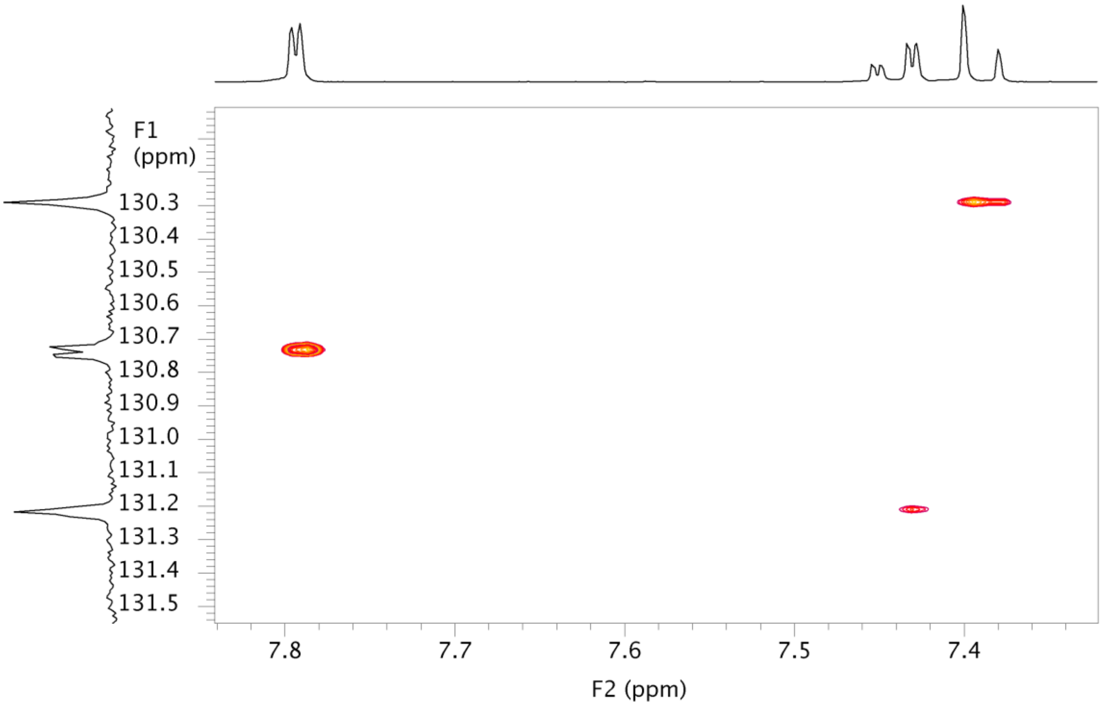

Figure S 74. ^{1}H-^{13}C bsgHSQC (500 MHz, $CDCl_3$) spectrum of compound **10-SS**.

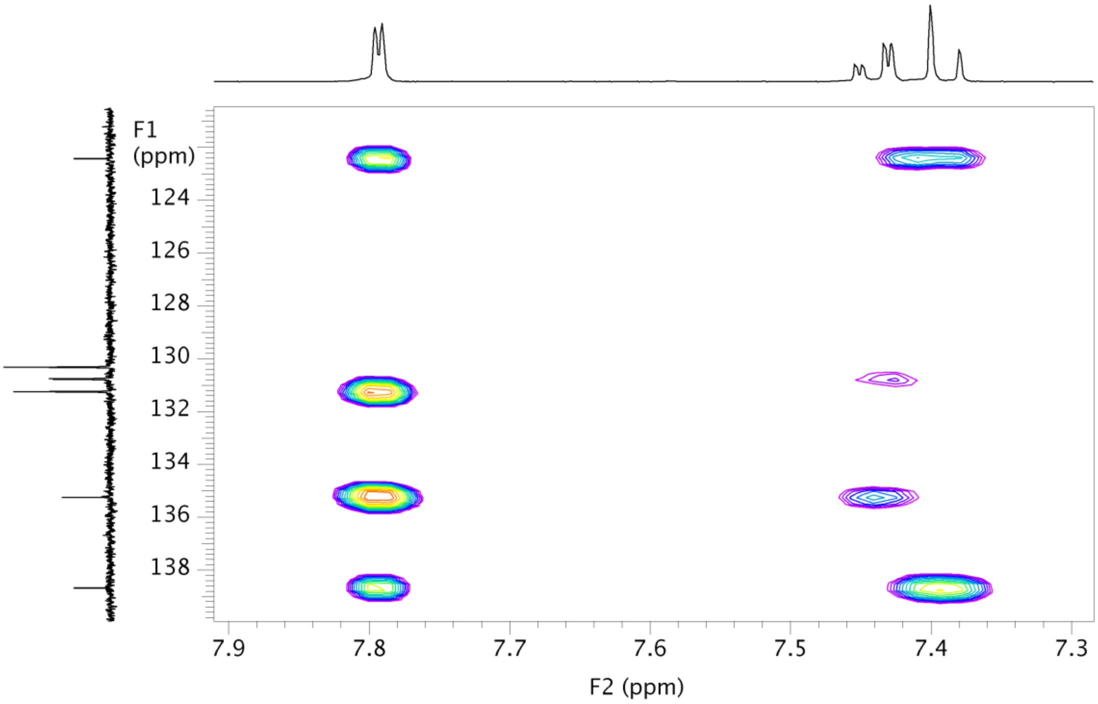

Figure S 75. ^{1}H-^{13}C gc2HSMBC (400 MHz, $CDCl_3$) spectrum of compound **10-SS**.

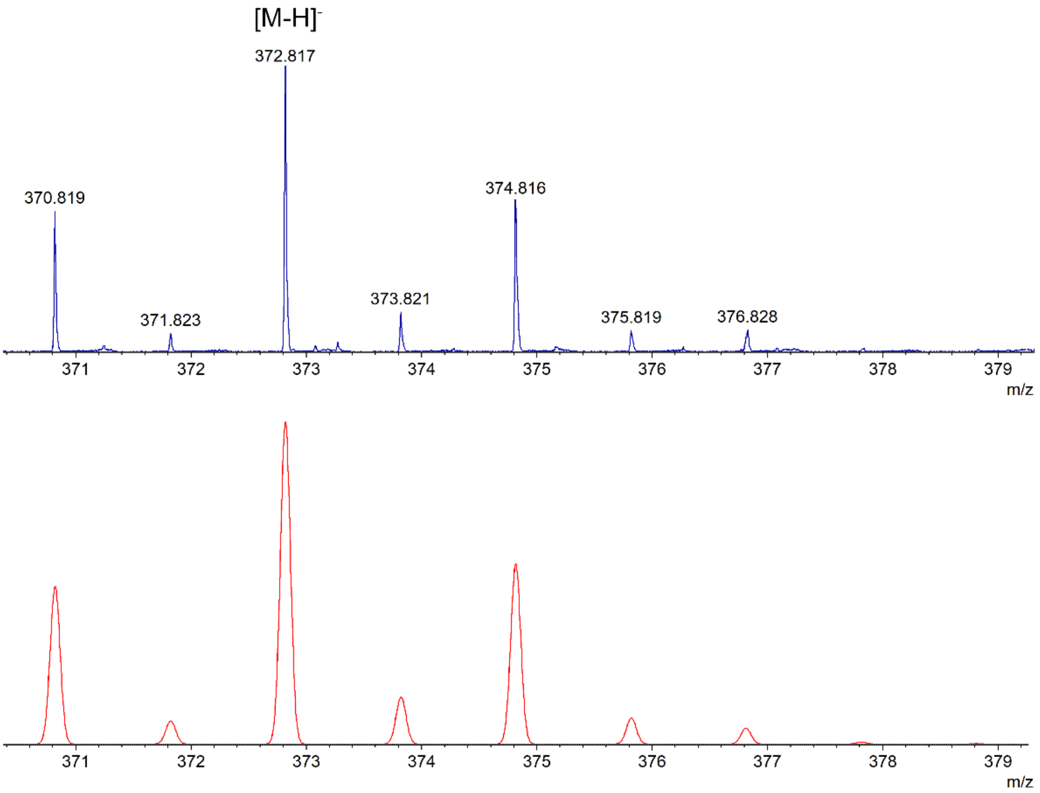

Figure S 76. HRMS (ESI-TOF), of compound **10-SS** [M-H]$^{-}$. Calculated (red), measured (blue).

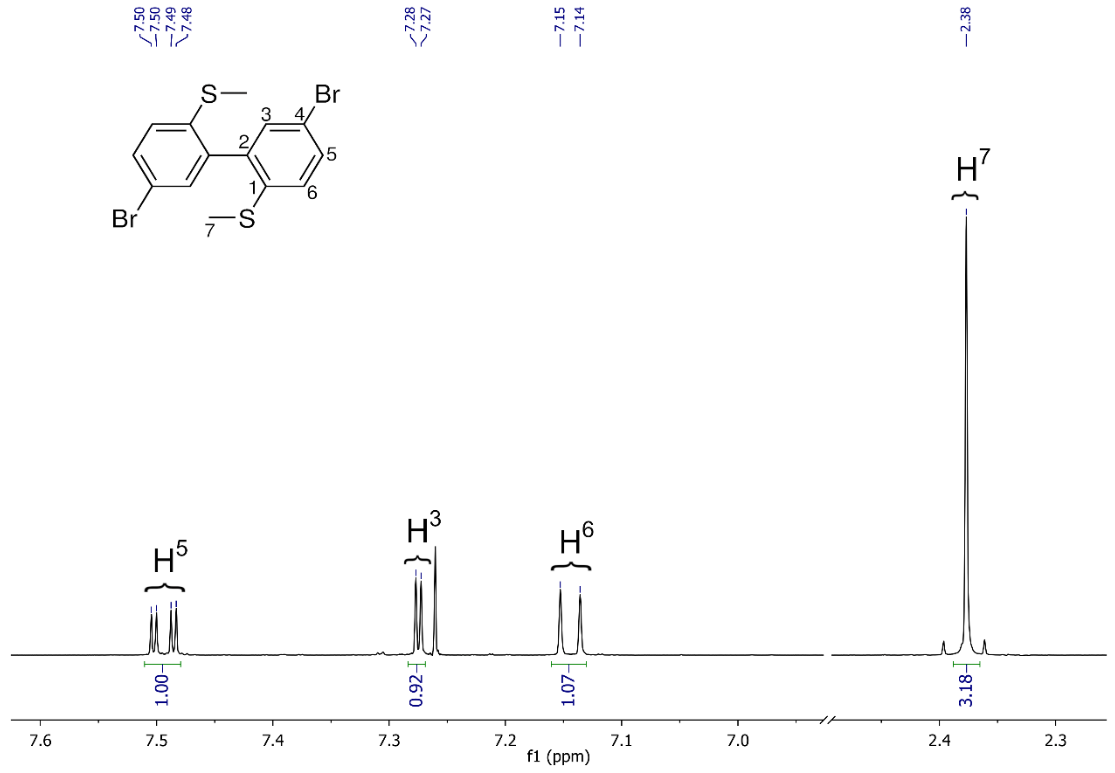

Figure S 77. ^{1}H-NMR (500 MHz, $CDCl_3$) spectrum of compound **11a**.

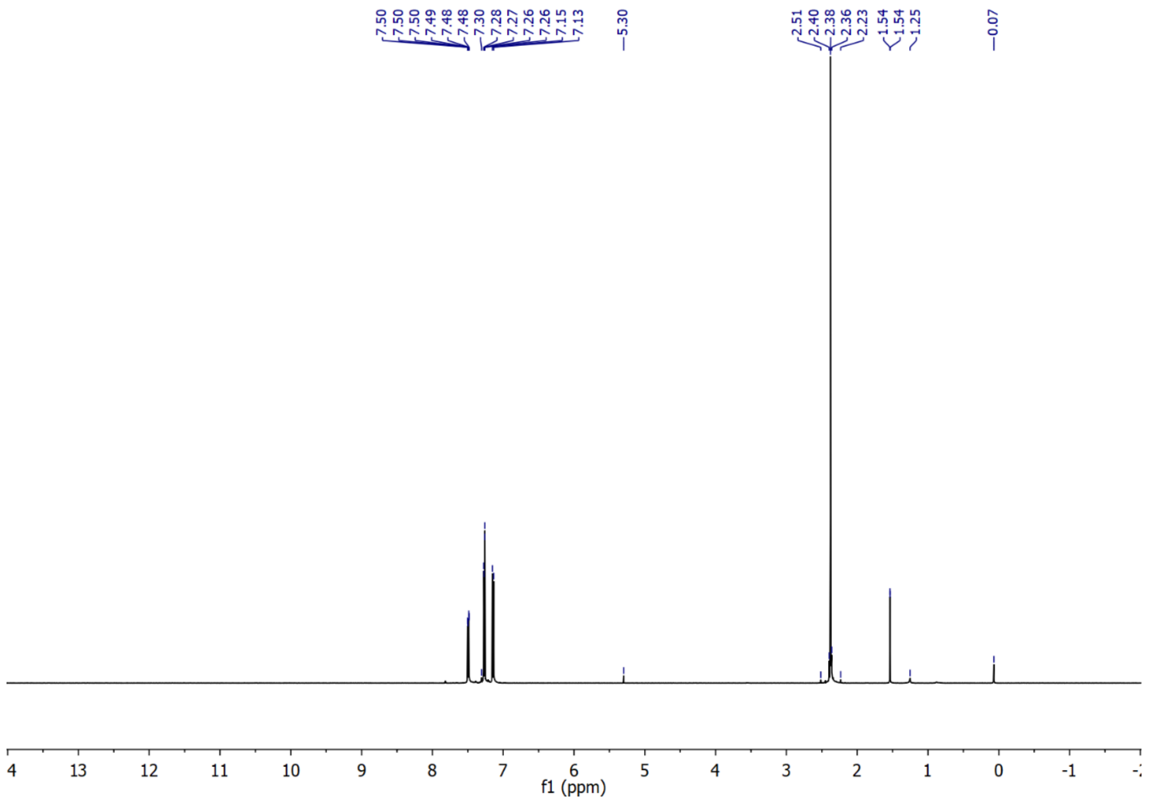

Figure S 78. Full ^{1}H-NMR (500 MHz, $CDCl_3$) spectrum of compound **11a**.

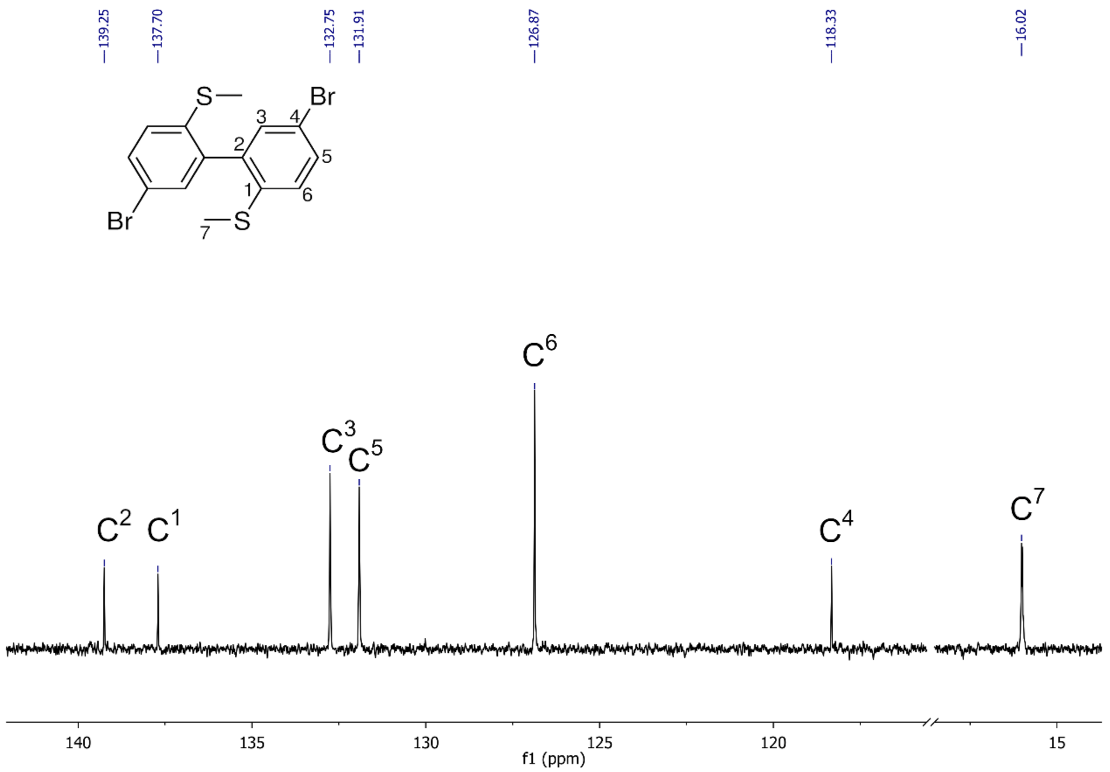

Figure S 79. $^{13}C\{^1H\}$-NMR (101 MHz, $CDCl_3$) spectrum of compound **11a**.

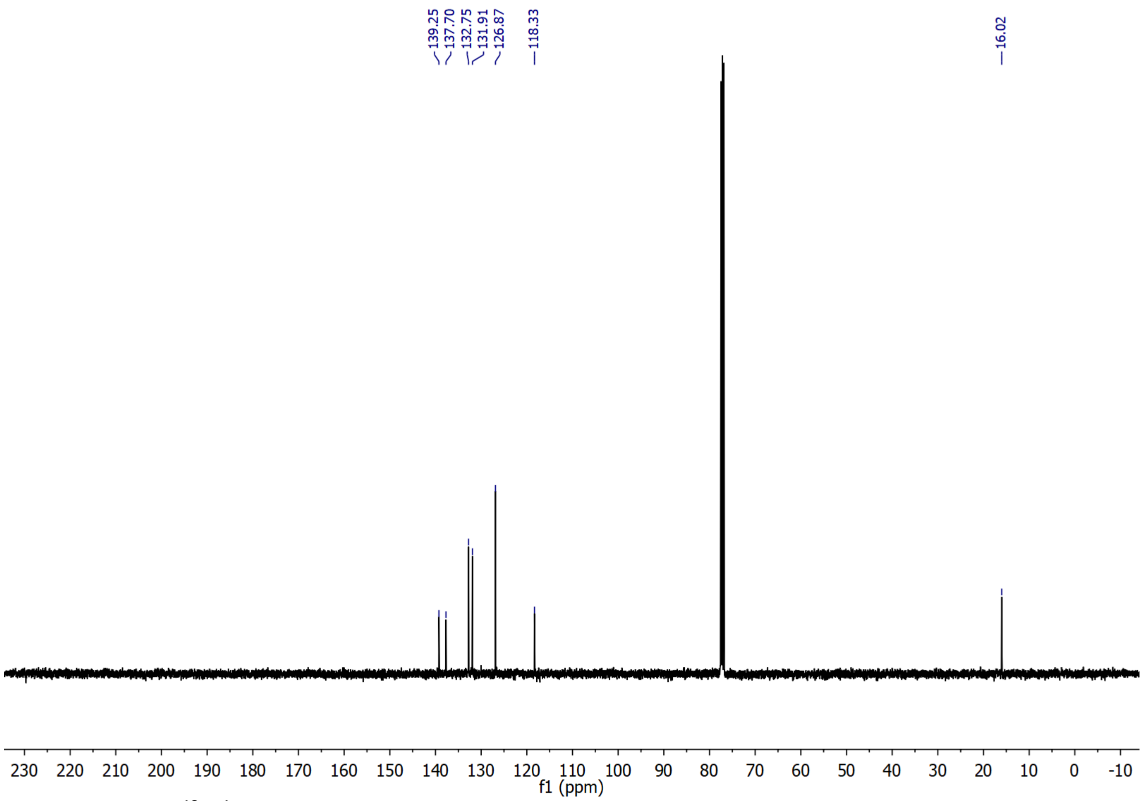

Figure S 80. Full $^{13}C\{^1H\}$-NMR (101 MHz, $CDCl_3$) spectrum of compound **11a**.

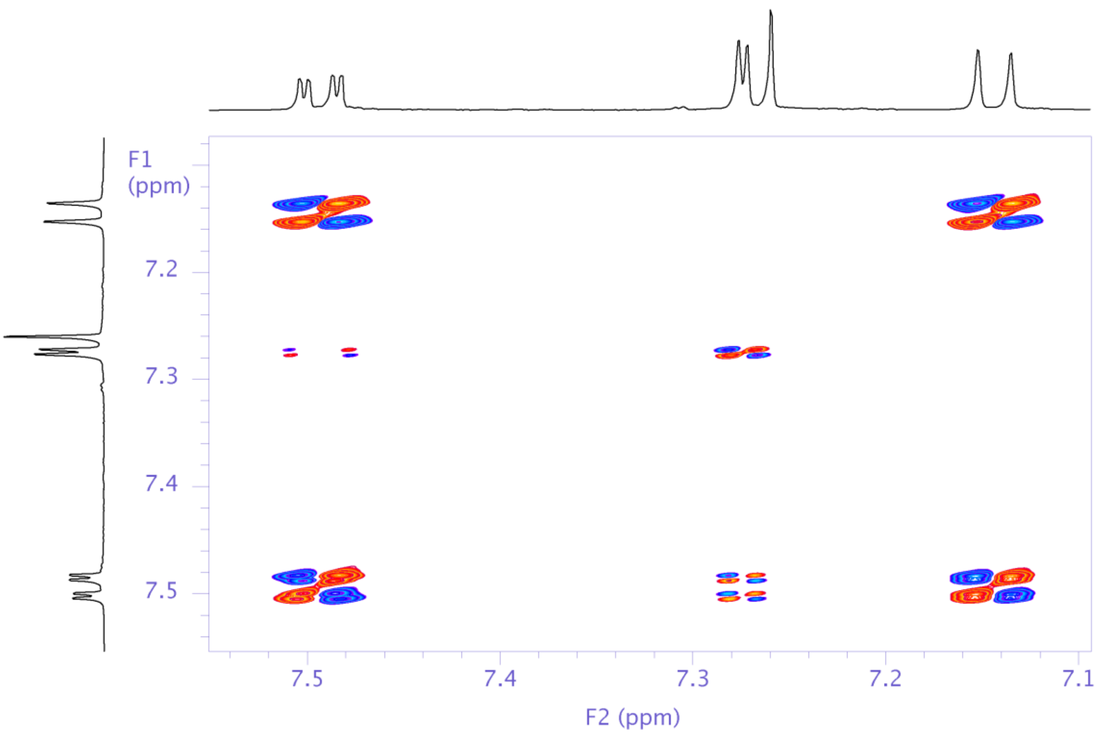

Figure S 81. ^{1}H-^{1}H gDQFCOSY (500 MHz, $CDCl_3$) spectrum of compound **11a**.

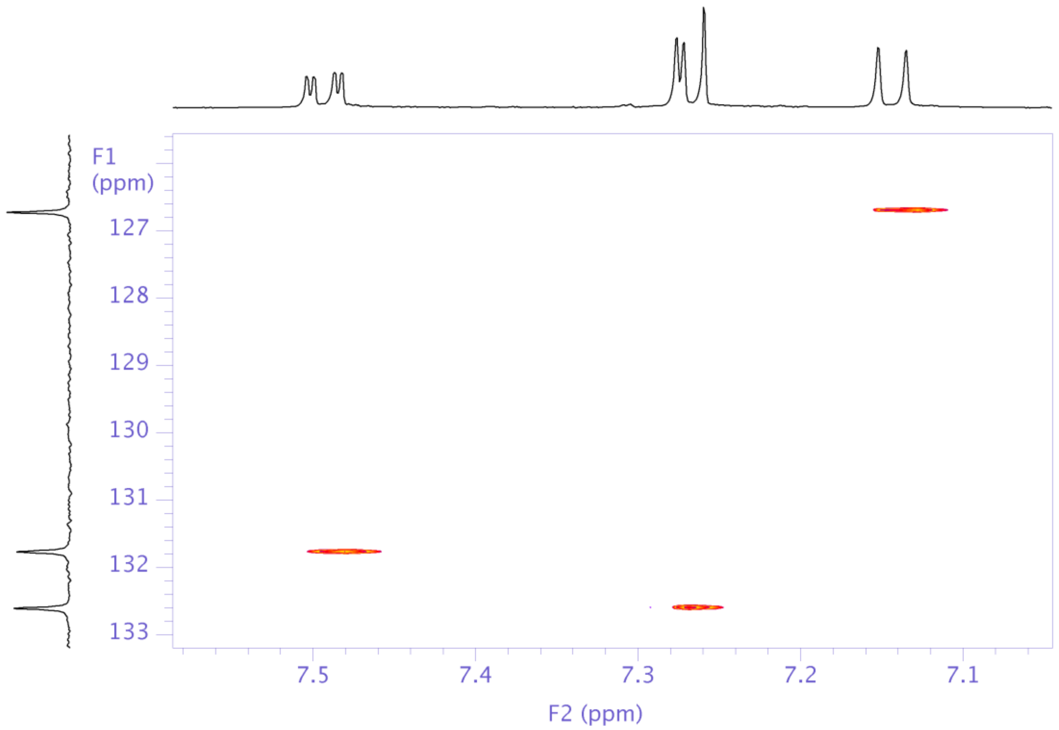

Figure S 82. ^{1}H-^{13}C bsgHSQCAD (500 MHz, $CDCl_3$) spectrum of compound **11a**.

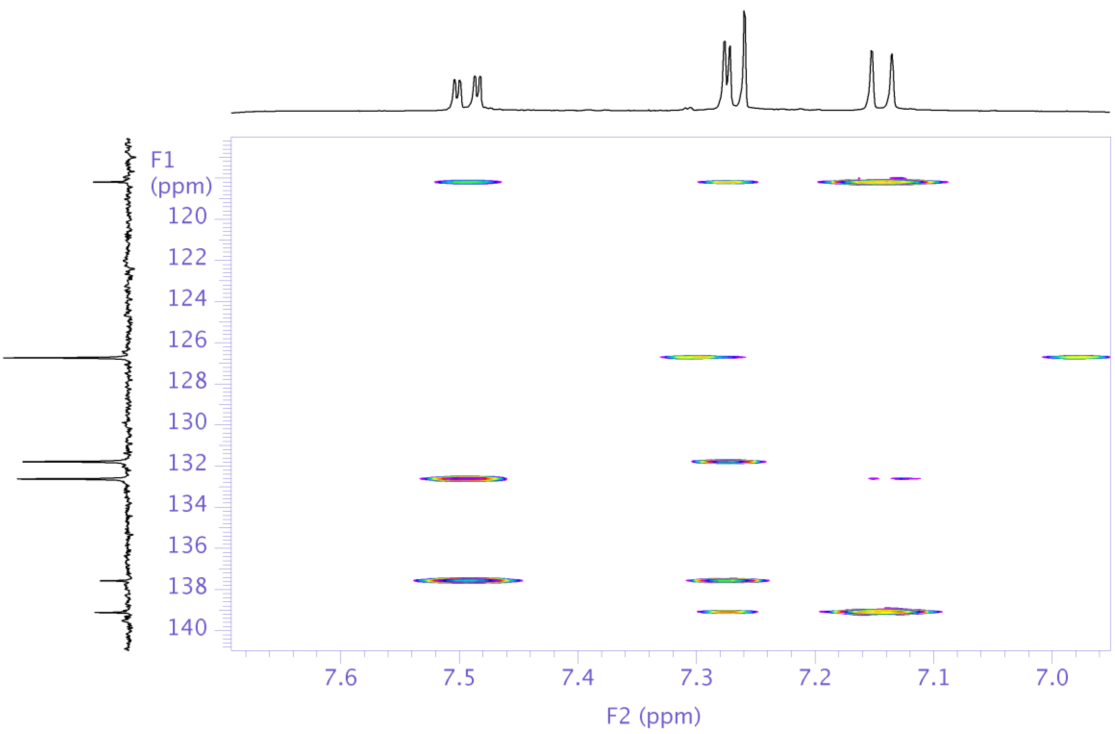

Figure S 83. ^{1}H-^{13}C bsgHSMBC (500 MHz, $CDCl_3$) spectrum of compound **11a**.

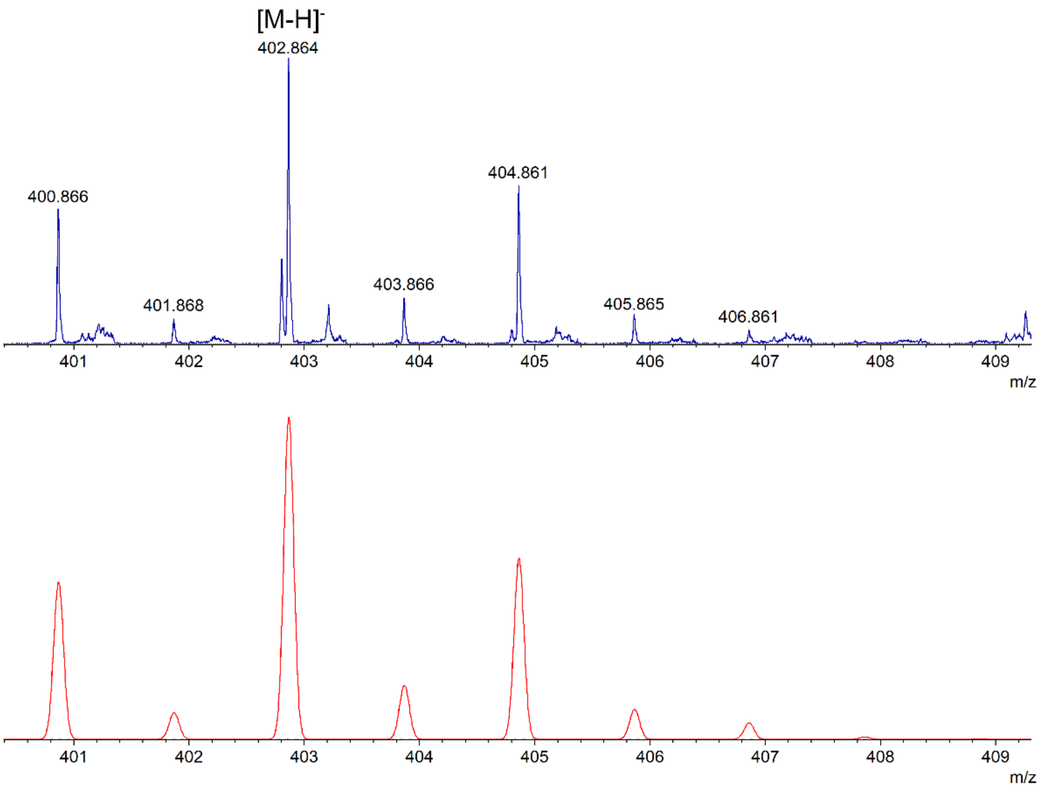

Figure S 84. HRMS (ESI-TOF), of compound **11a** $[M-H]^-$. Calculated (red), measured (blue).

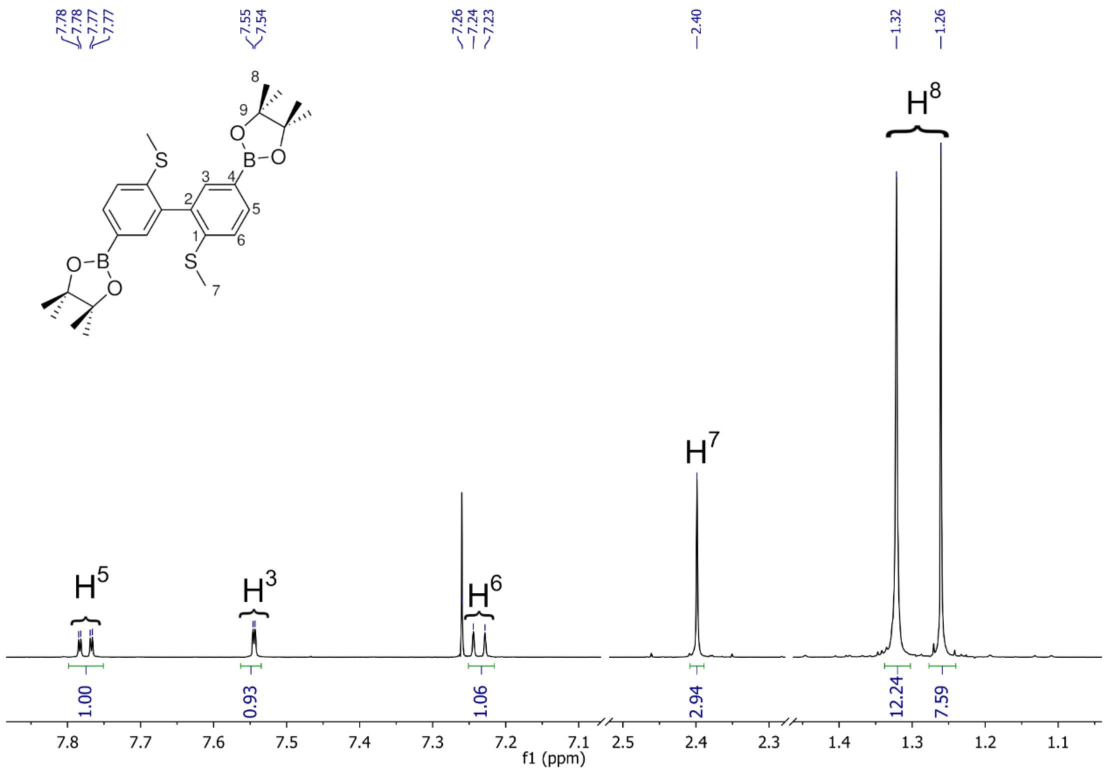

Figure S 85. ^{1}H-NMR (500 MHz, $CDCl_3$) spectrum of compound **12a**.

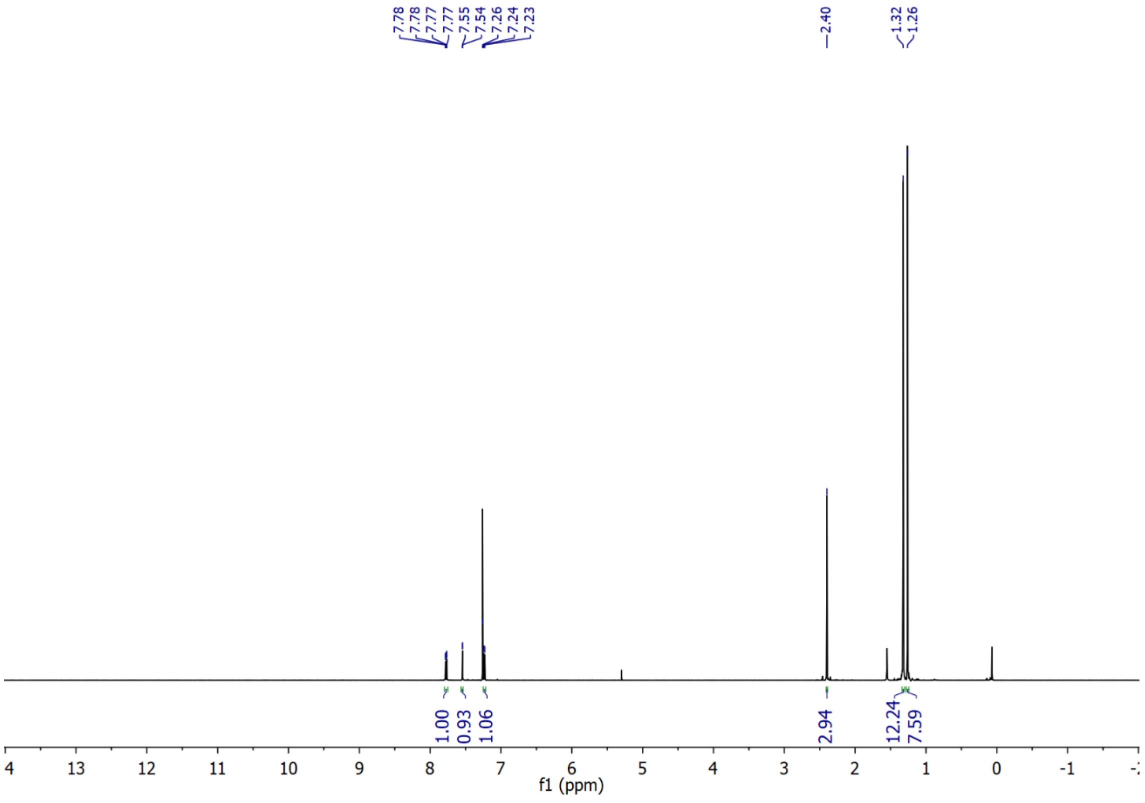

Figure S 86. Full ^{1}H-NMR (500 MHz, $CDCl_3$) spectrum of compound **12a**.

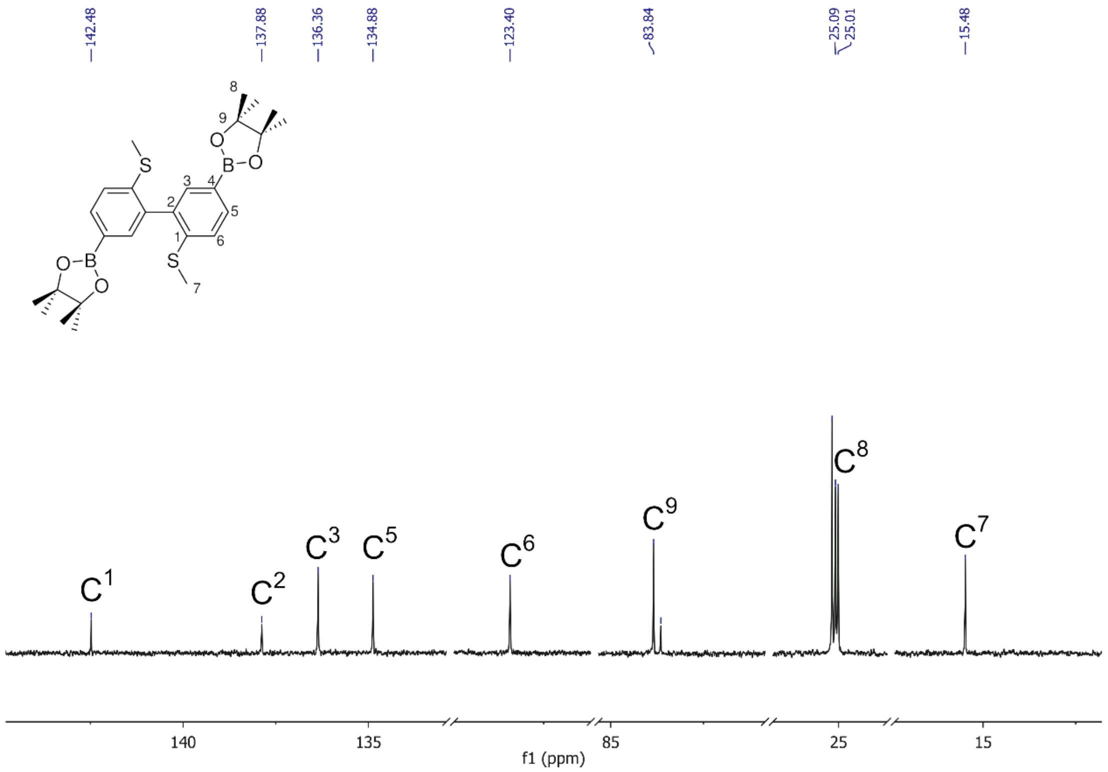

Figure S 87. $^{13}C\{^1H\}$-NMR (101 MHz, $CDCl_3$) spectrum of compound **12a**.

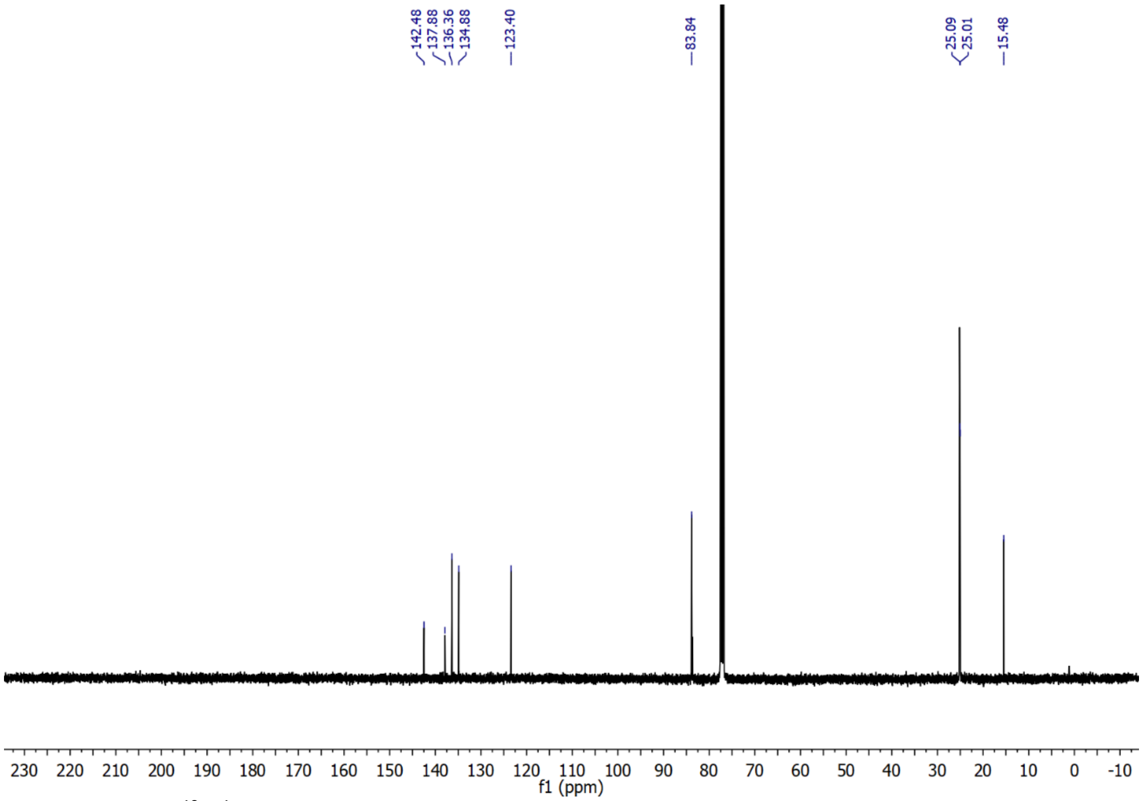

Figure S 88. Full $^{13}C\{^1H\}$-NMR (101 MHz, $CDCl_3$) spectrum of compound **12a**.

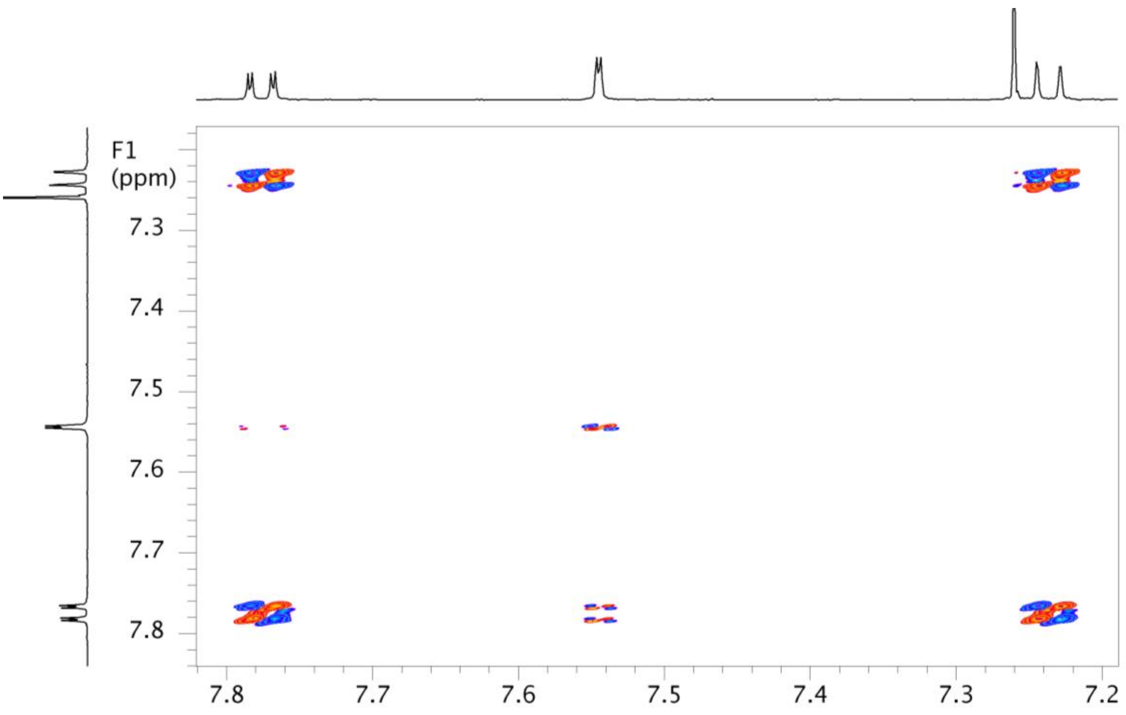

Figure S 89. ^{1}H-^{1}H gDQFCOSY (500 MHz, $CDCl_3$) spectrum of compound **12a**.

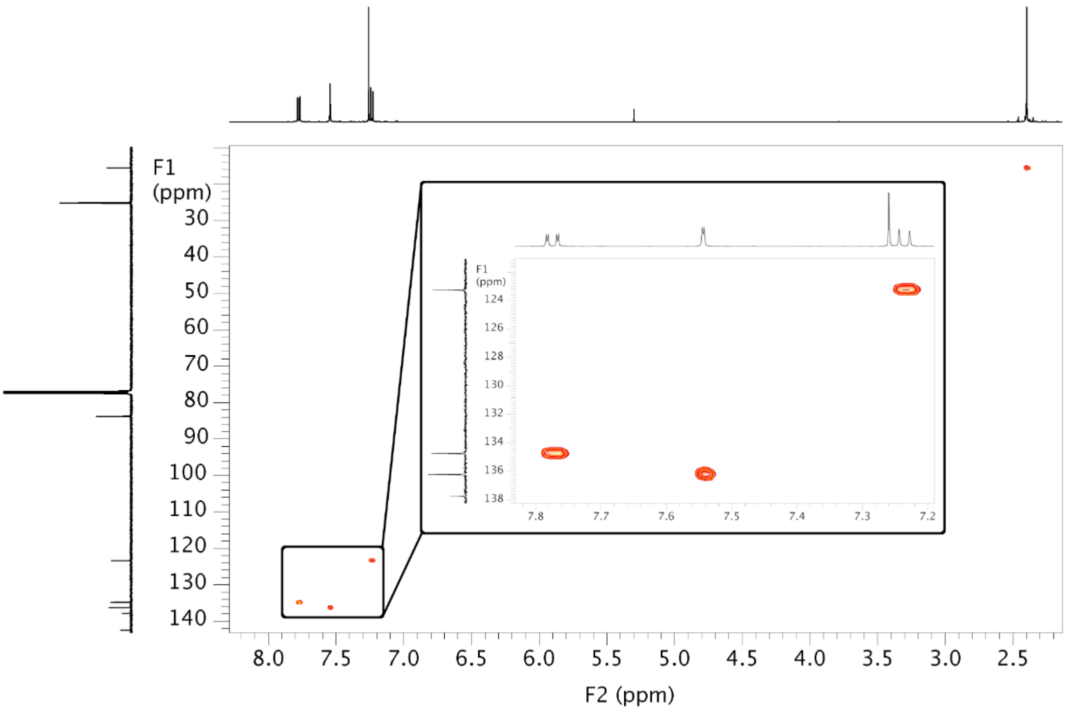

Figure S 90. ^{1}H-^{13}C gc2HSQC (500 MHz, $CDCl_3$) spectrum of compound **12a**.

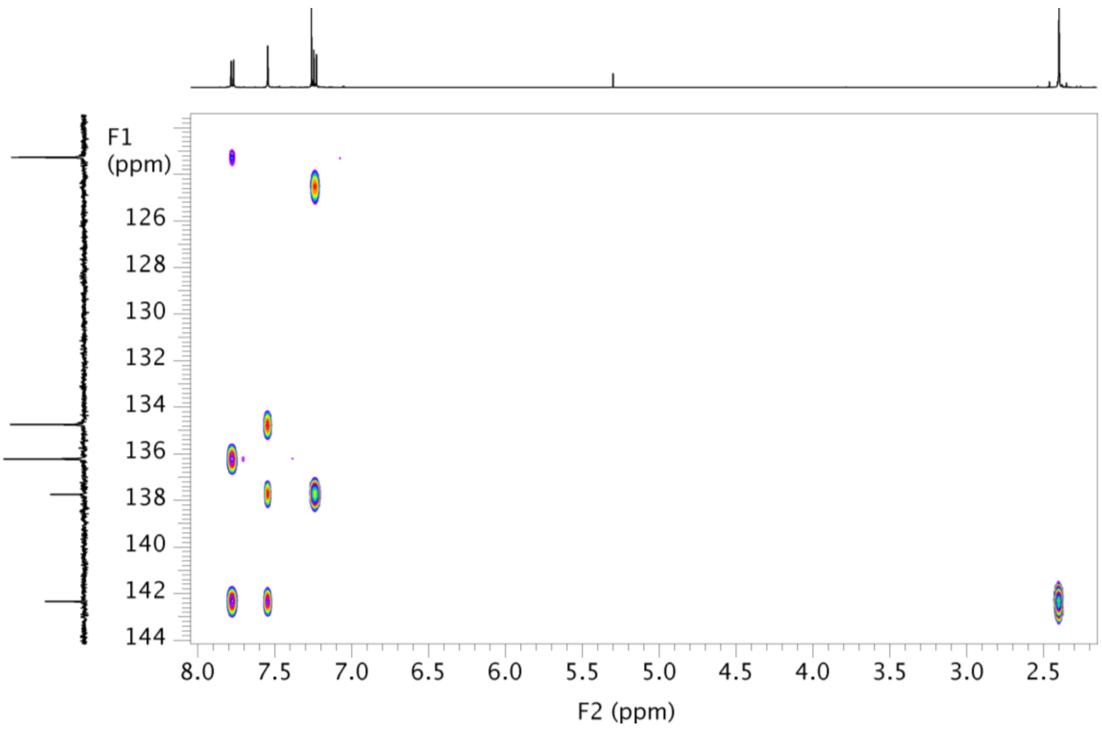

Figure S 91. ^{1}H-^{13}C gc2HMBC (500 MHz, $CDCl_3$) spectrum of compound **12a**.

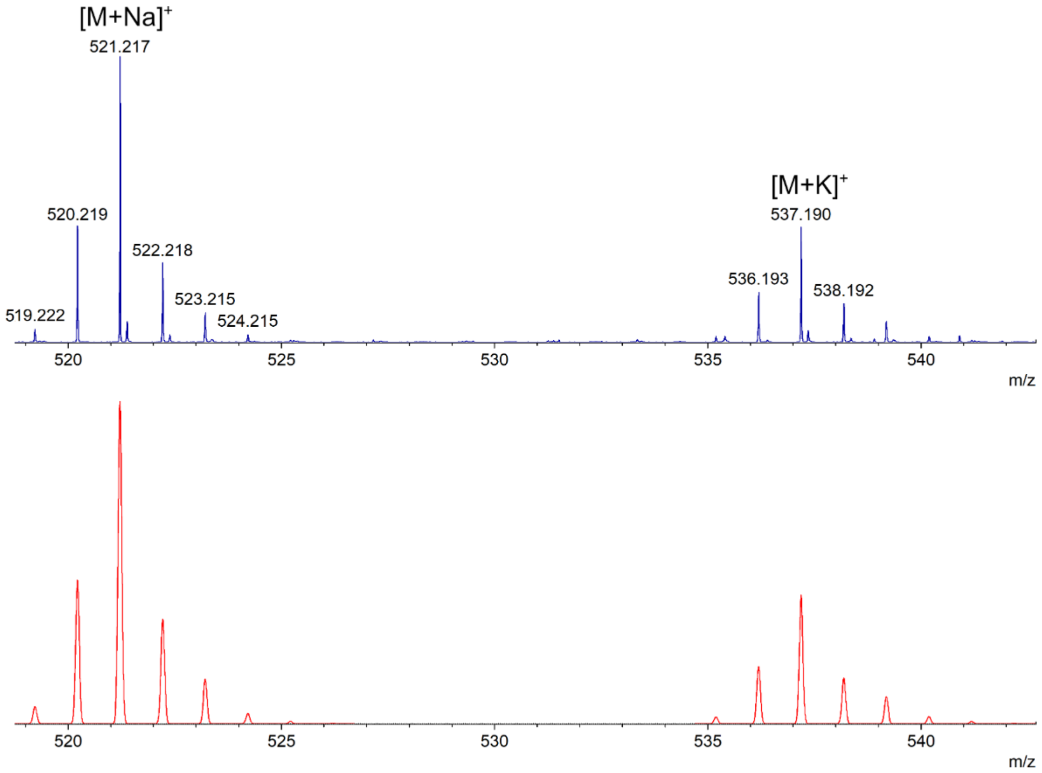

Figure S 92. HRMS (ESI-TOF), of compound **12a**, $[M+Na]^+$ and $[M+K]^+$. Calculated (red), measured (blue).

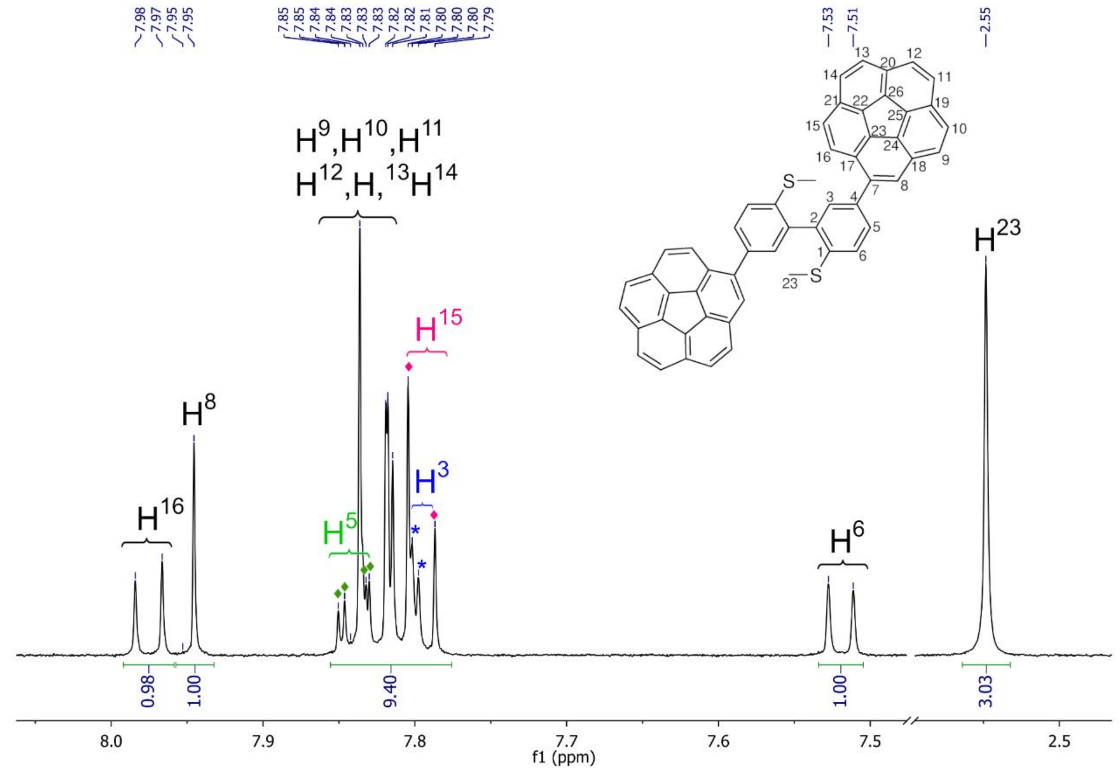

Figure S 93. ^{1}H-NMR (500 MHz, $CDCl_3$) spectrum of compound **13-SMe**.

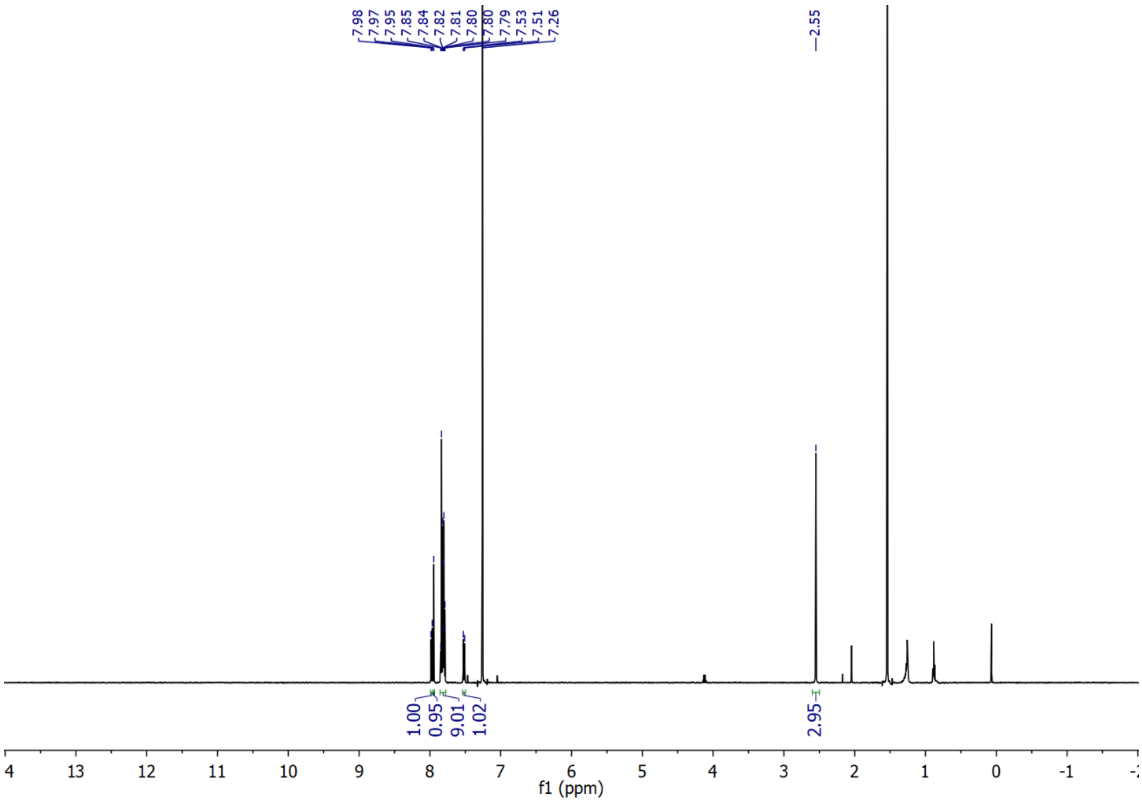

Figure S 94. Full ^{1}H-NMR (500 MHz, $CDCl_3$) spectrum of compound **13-SMe**.

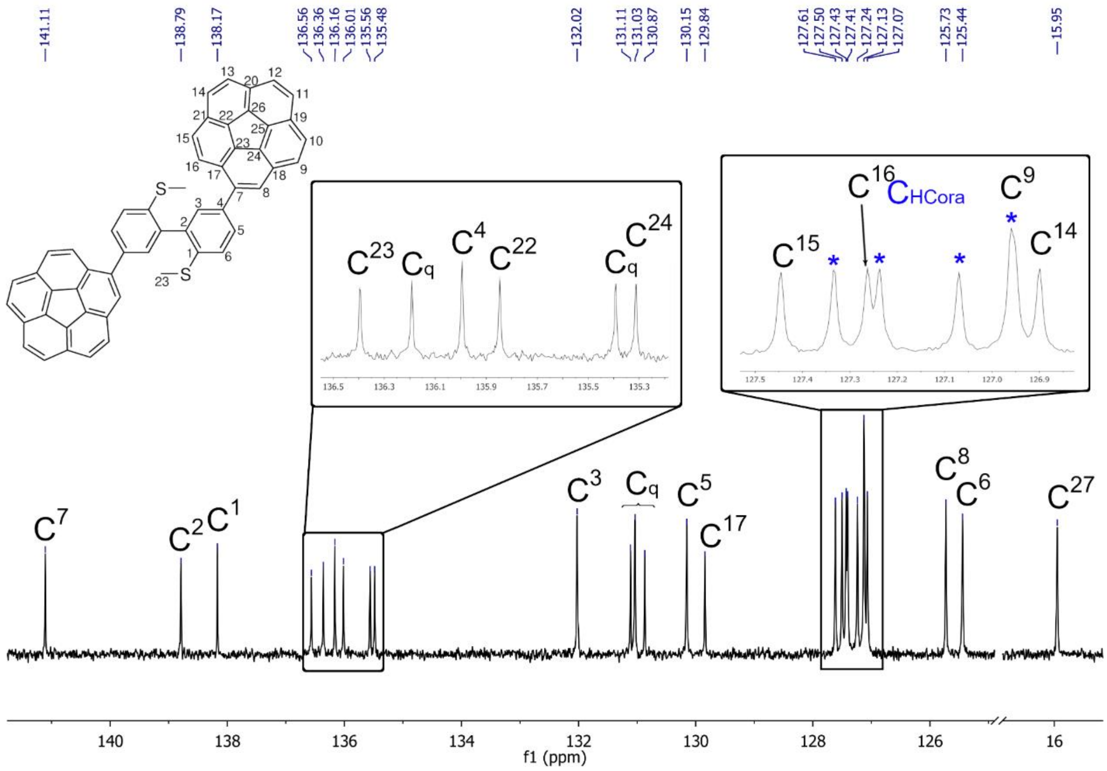

Figure S 95. $^{13}C\{^{1}H\}$-NMR (126 MHz, $CDCl_3$) spectrum of compound **13-SMe**.

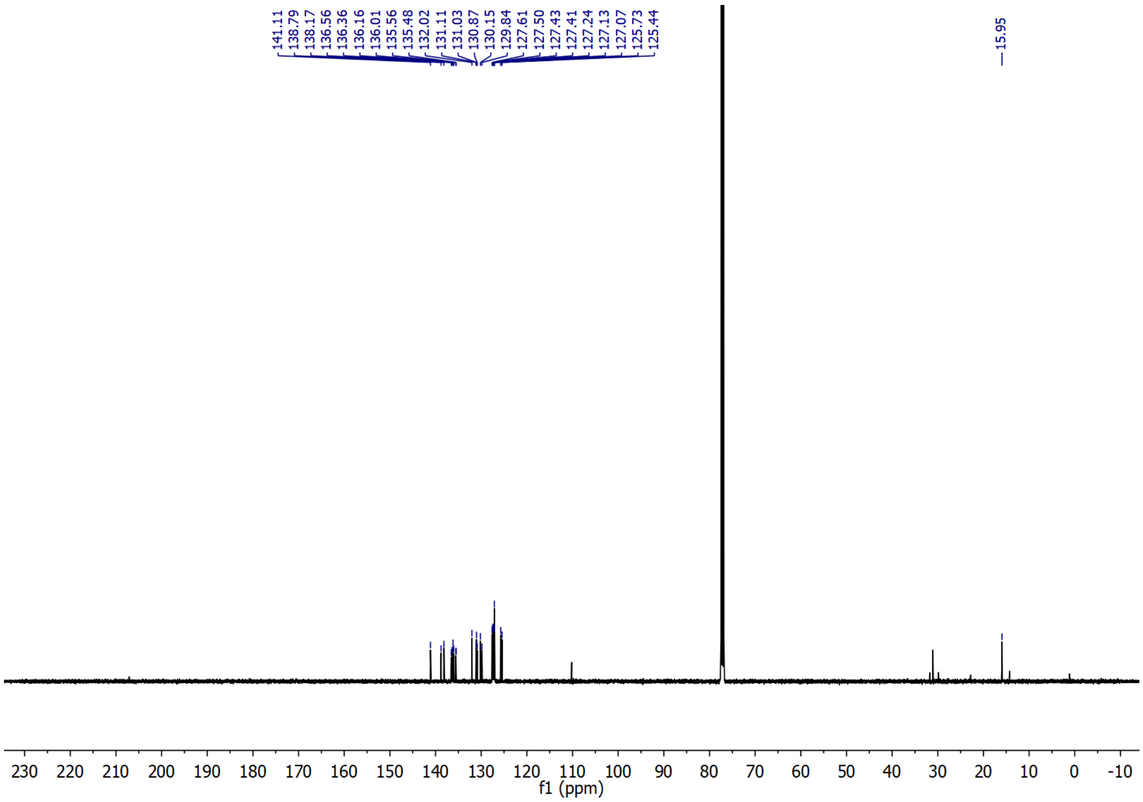

Figure S 96. Full $^{13}C\{^{1}H\}$-NMR (126 MHz, $CDCl_3$) spectrum of compound **13-SMe**.

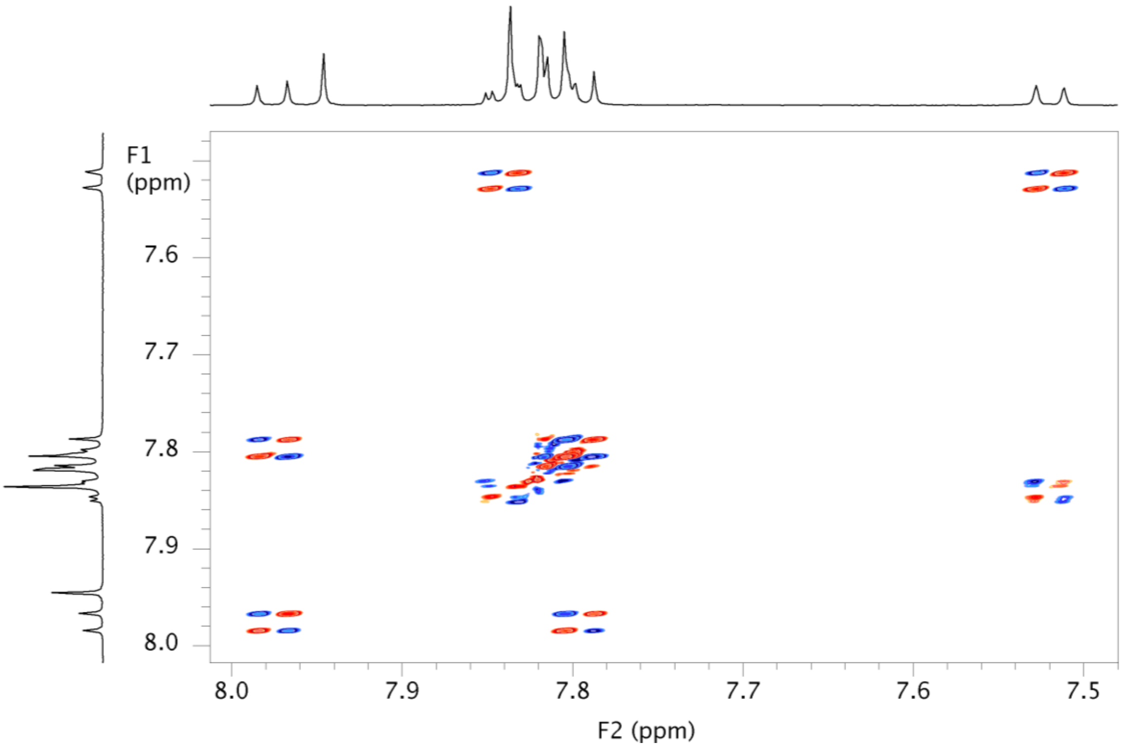

Figure S 97. ^{1}H-^{1}H gDQFCOSY (500 MHz, $CDCl_3$) spectrum of compound **13-SMe**.

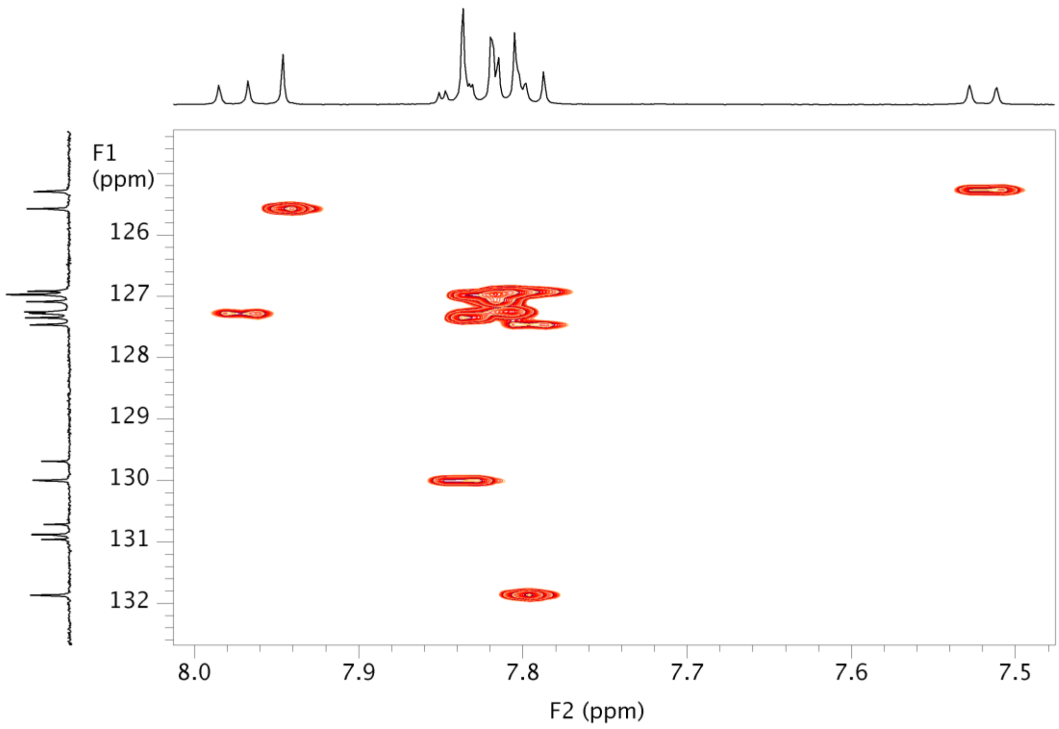

Figure S 98. ^{1}H-^{13}C bsgHSQCAD (500 MHz, $CDCl_3$) spectrum of compound **13-SMe**.

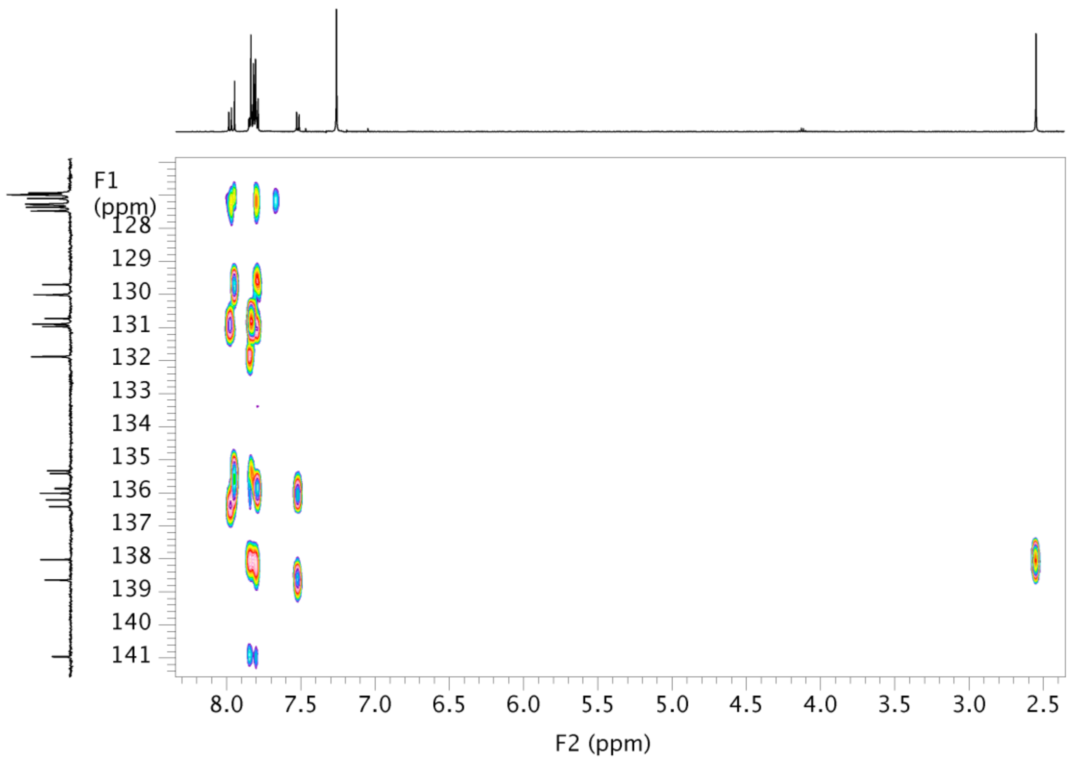

Figure S 99. ^{1}H-^{13}C gc2HMBC (500 MHz, $CDCl_3$) spectrum of compound **13-SMe**. (Crosspeak between H_{25} and C_1 is observed)

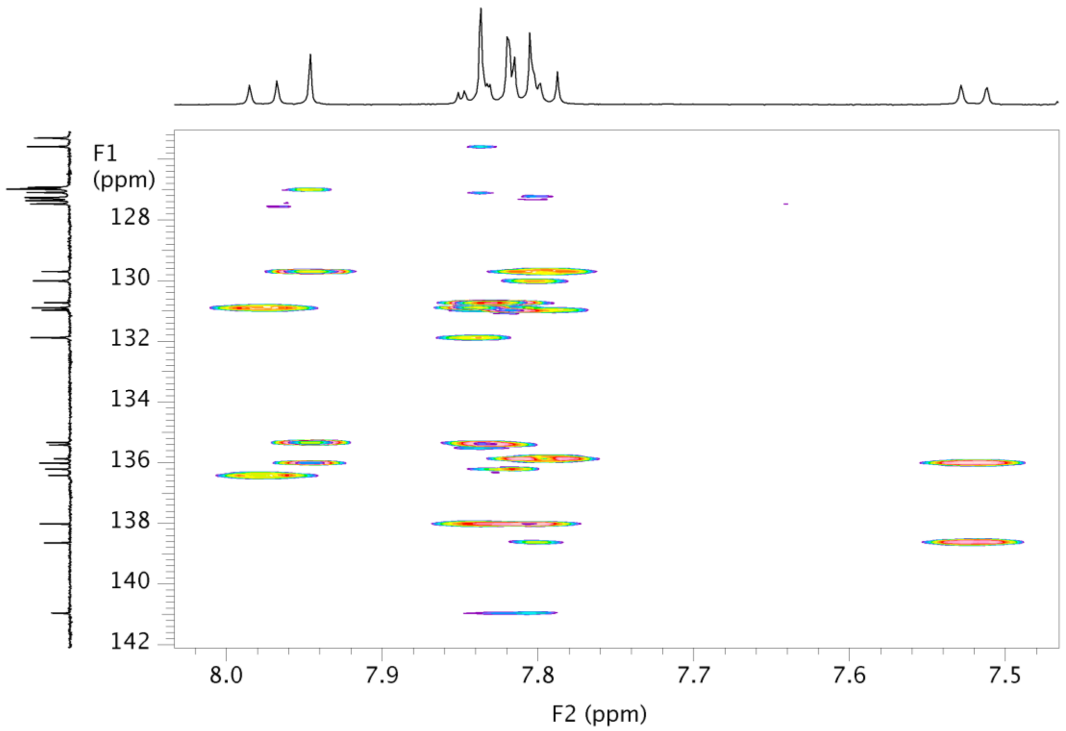

Figure S 100. ^{1}H-^{13}C bsgHMBC (500 MHz, $CDCl_3$) spectrum of compound **13-SMe**.

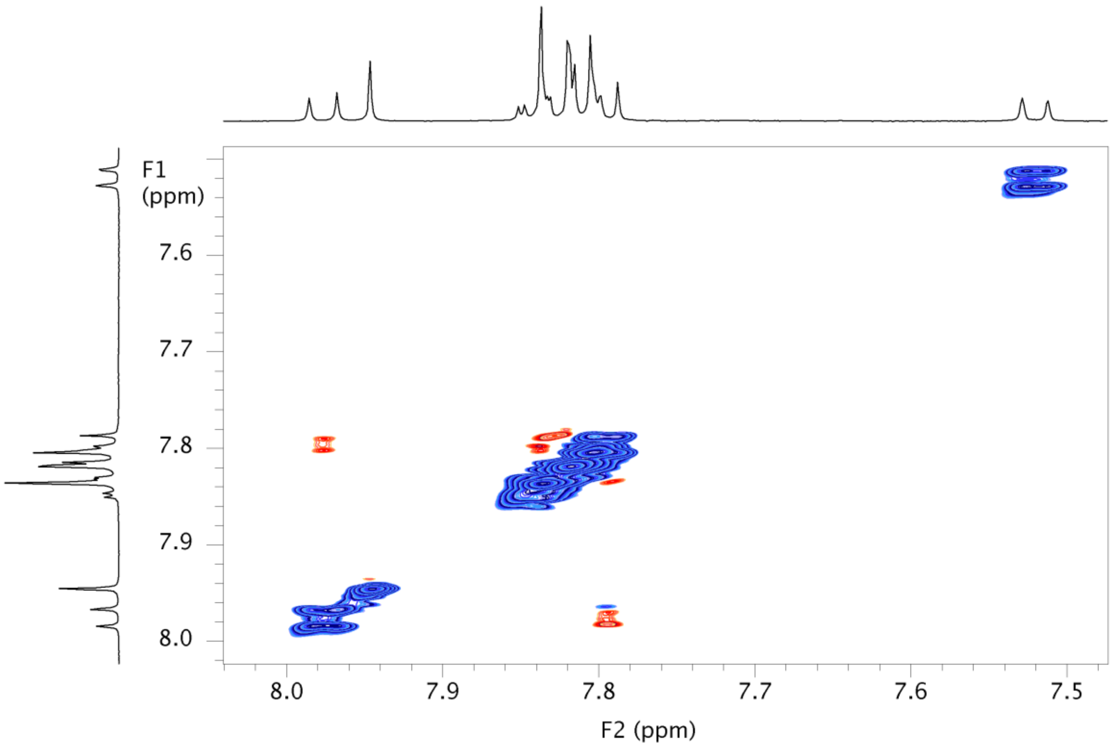

Figure S 101. ^{1}H-^{1}H ROESYAD (500 MHz, $CDCl_3$) spectrum of compound **13-SMe**. Spinlock time: 500 ms.

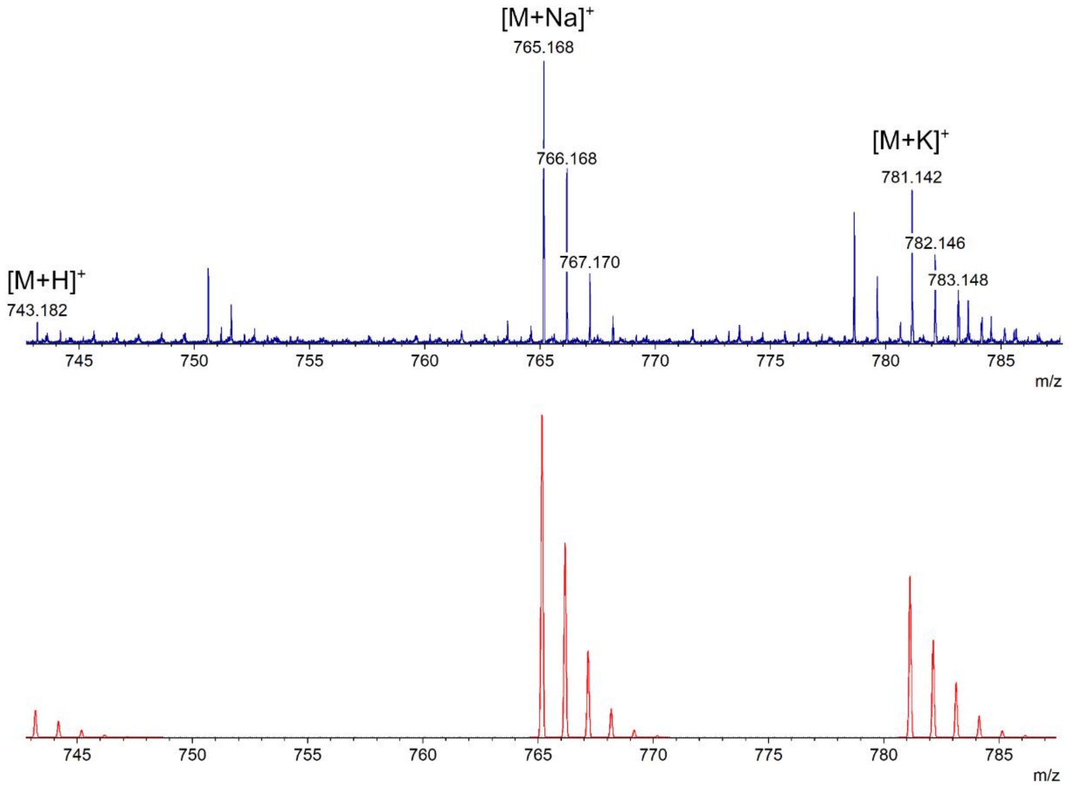

Figure S 102. HRMS (ESI-TOF), of compound **13-SMe**, $[M+H]^+$, $[M+Na]^+$ and $[M+K]^+$. Calculated (red), measured (blue).

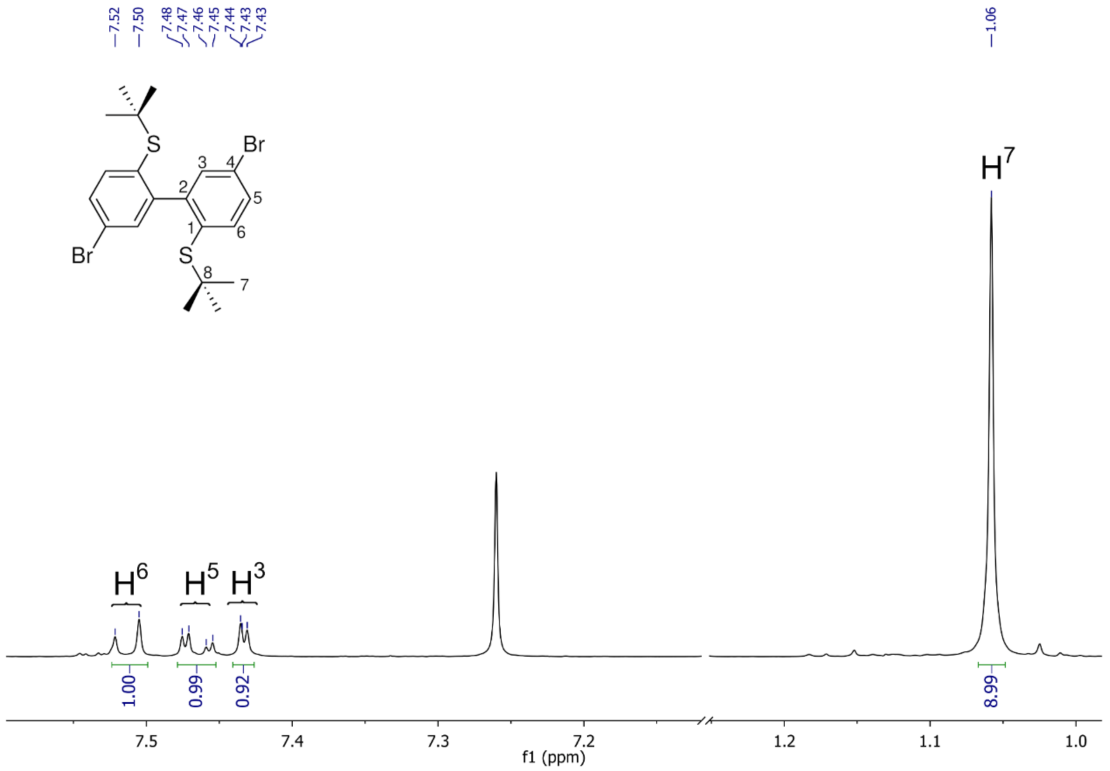

Figure S 103. ^{1}H-NMR (500 MHz, $CDCl_3$) spectrum of compound **11b**.

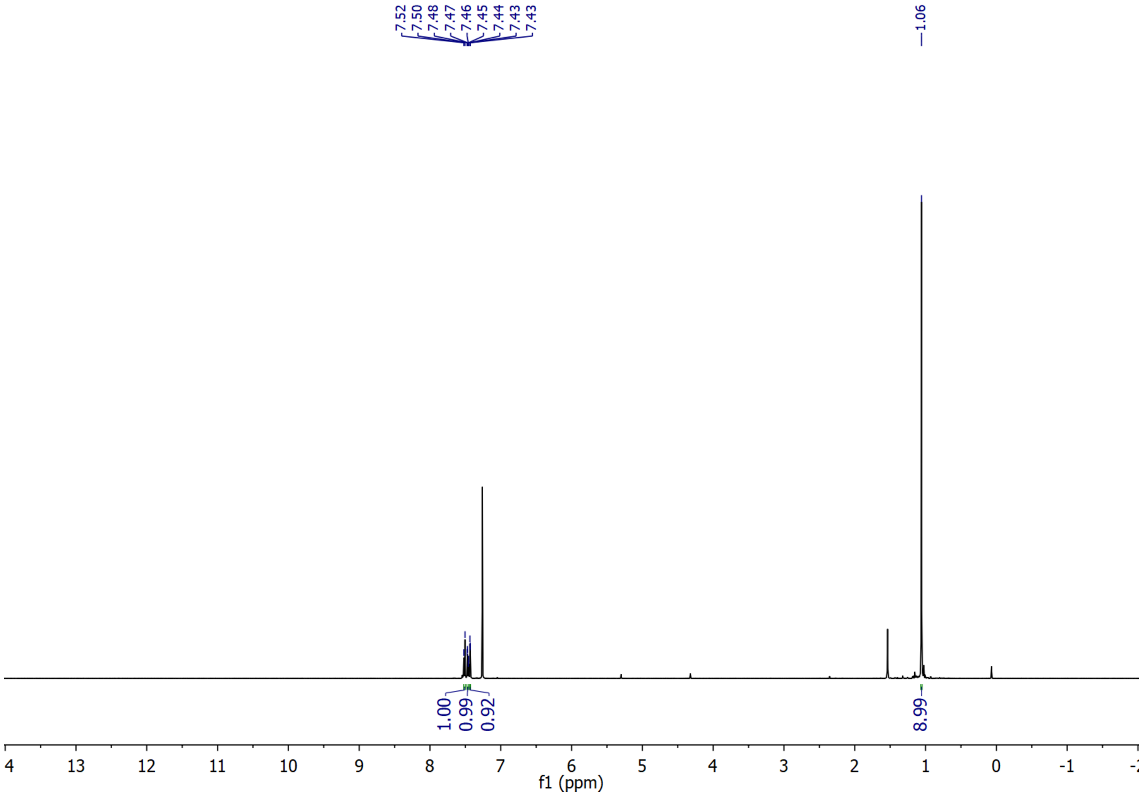

Figure S 104. Full ^{1}H-NMR (500 MHz, $CDCl_3$) spectrum of compound **11b**.

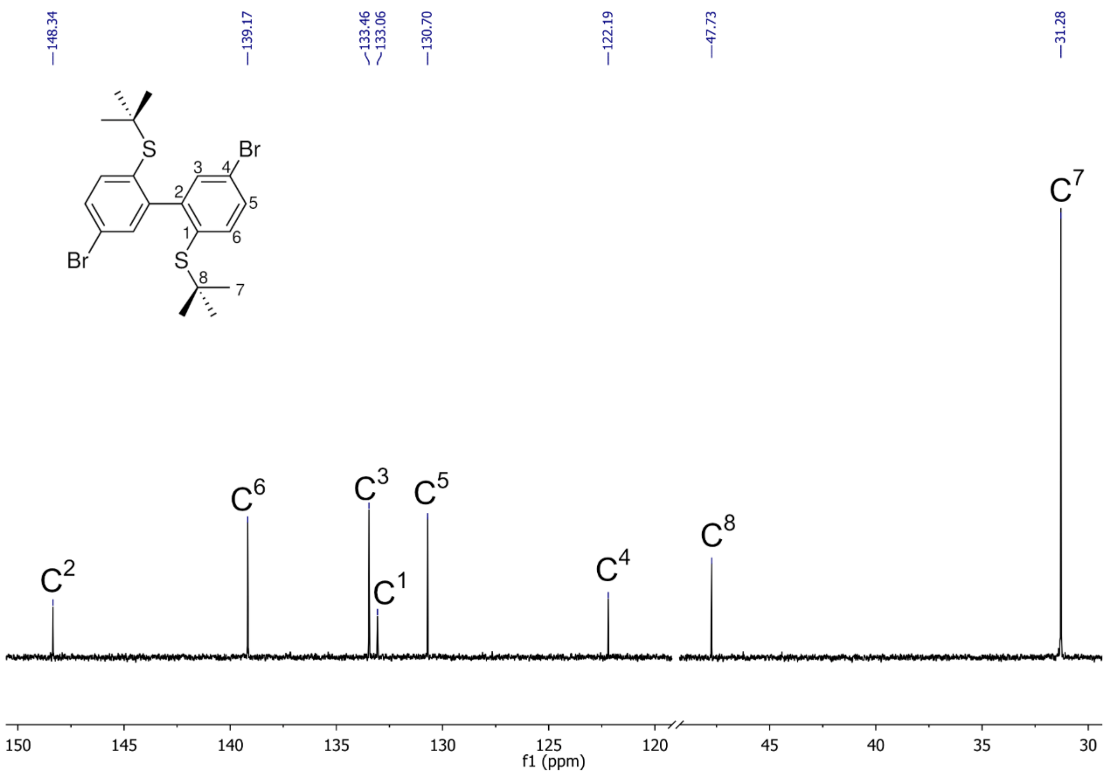

Figure S 105. $^{13}C\{^1H\}$-NMR (101 MHz, $CDCl_3$) spectrum of compound **11b**.

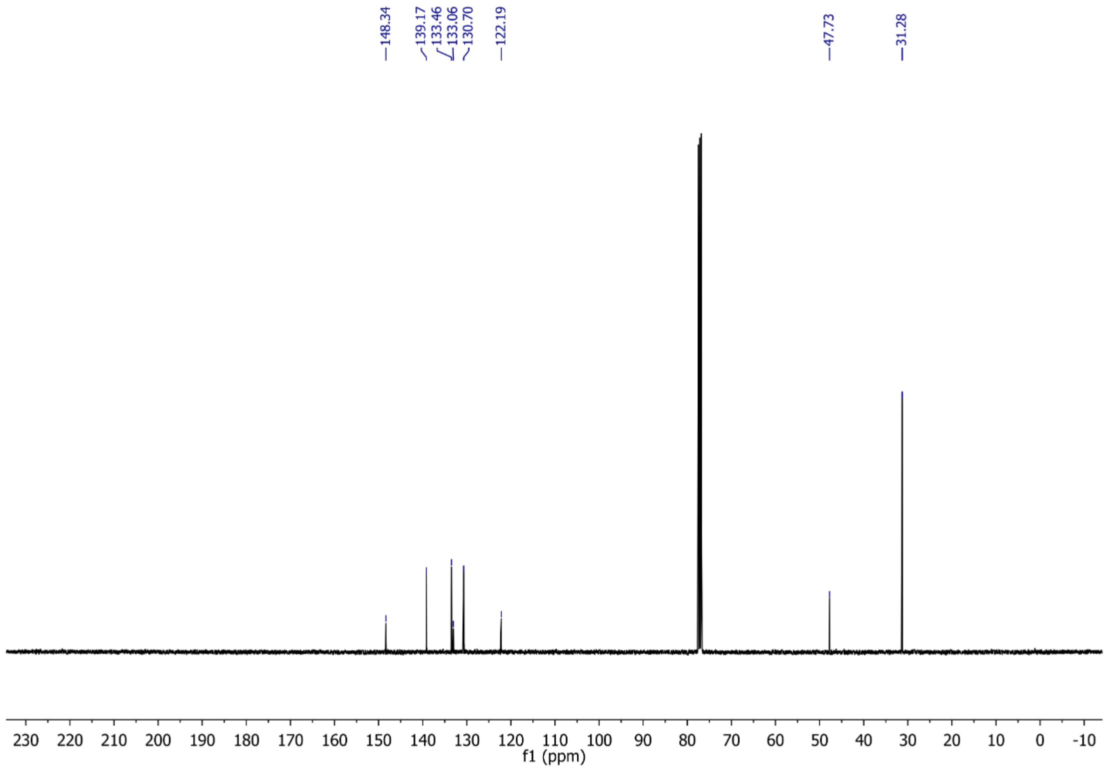

Figure S 106. Full $^{13}C\{^1H\}$-NMR (101 MHz, $CDCl_3$) spectrum of compound **11b**.

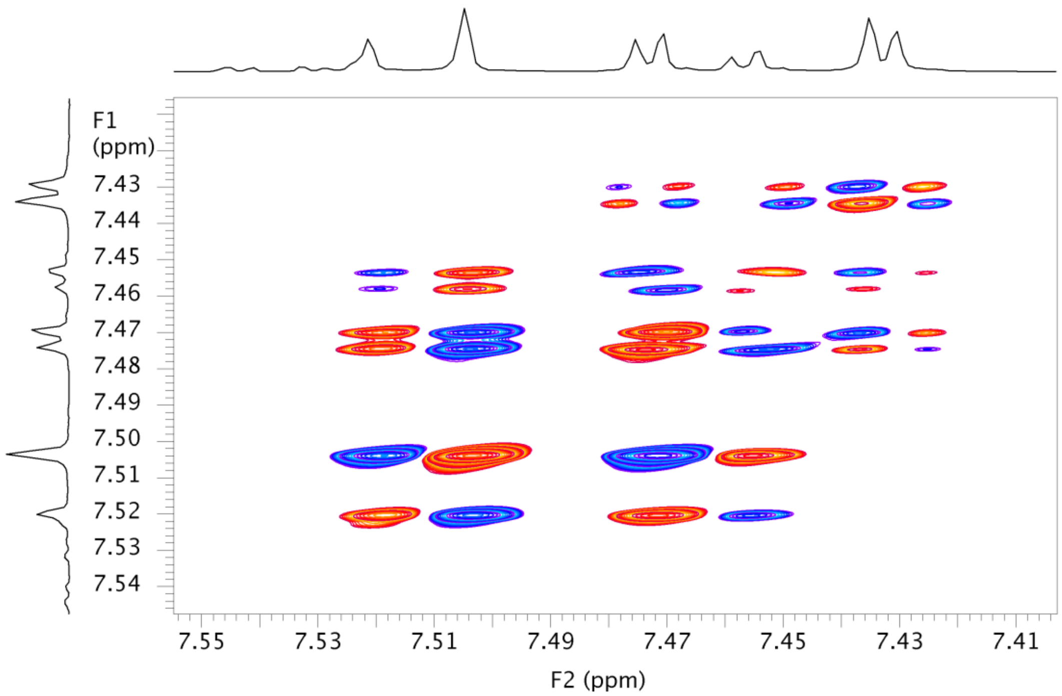

Figure S 107. ^{1}H-^{1}H gDQFCOSY (500 MHz, $CDCl_3$) spectrum of compound 11b.

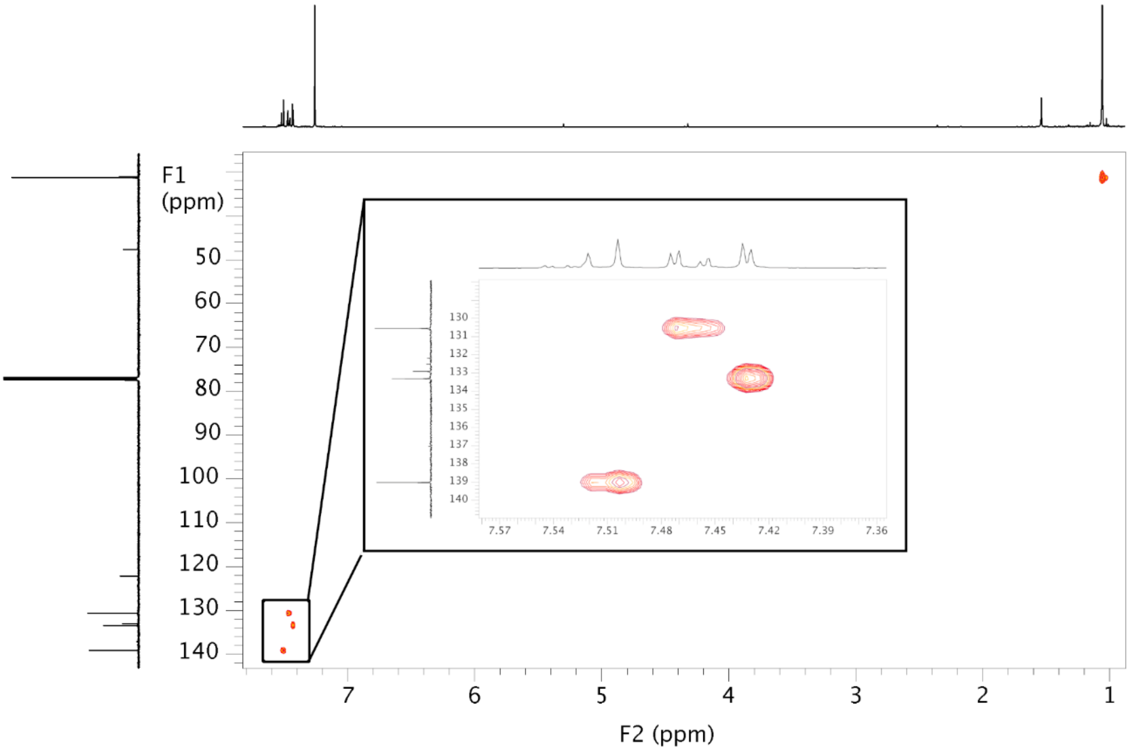

Figure S 108. ^{1}H-^{13}C gc2HSQC (500 MHz, $CDCl_3$) spectrum of compound **11b**.

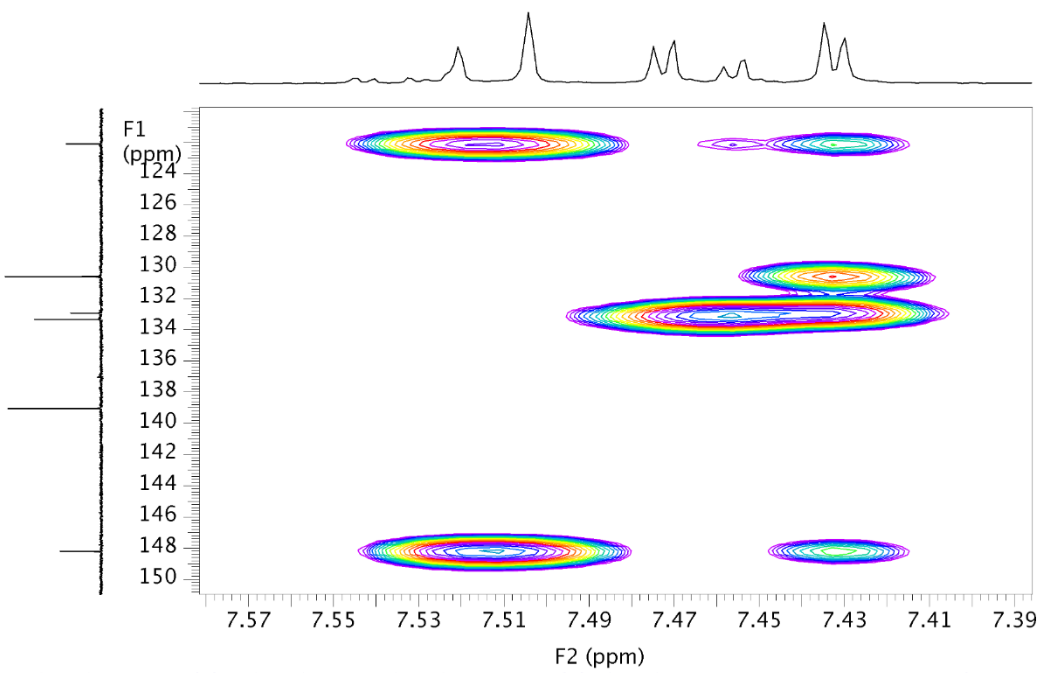

Figure S 109. ^{1}H-^{13}C gc2HMBC (500 MHz, $CDCl_3$) spectrum of compound **11b**.

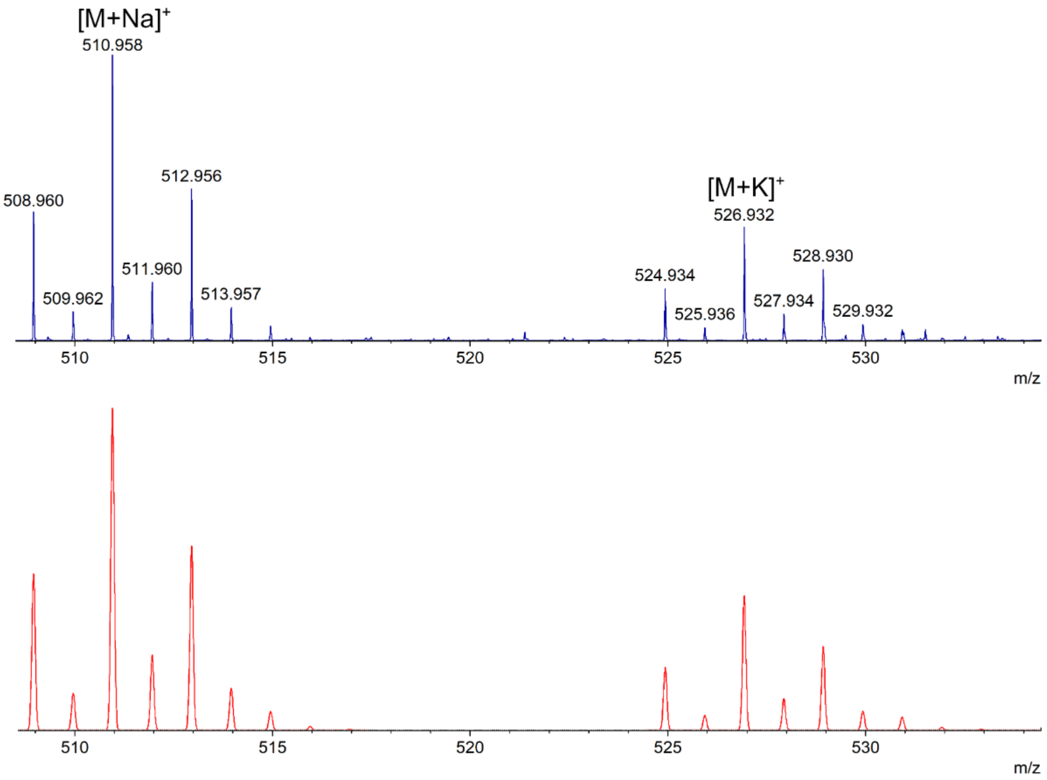

Figure S 110. HRMS (ESI-TOF), of compound **11b**, $[M-Na]^+$ and $[M-K]^+$. Calculated (red), measured (blue).

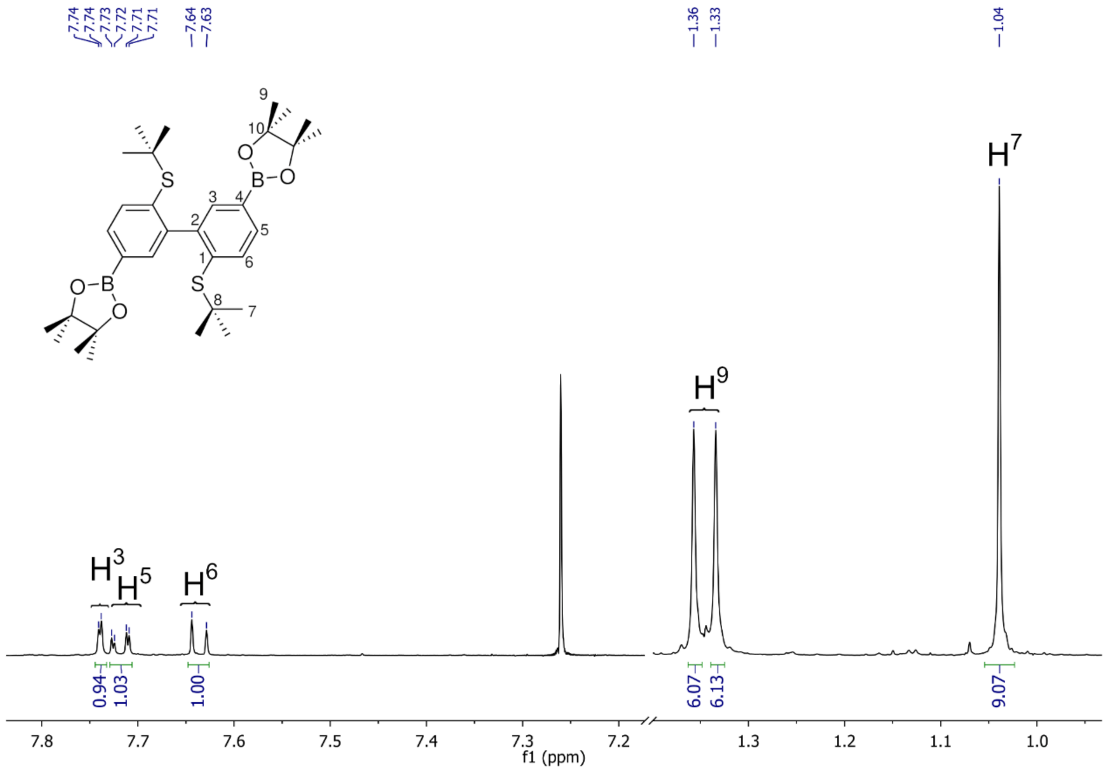

Figure S 111. ^{1}H-NMR (500 MHz, $CDCl_3$) spectrum of compound **12b**.

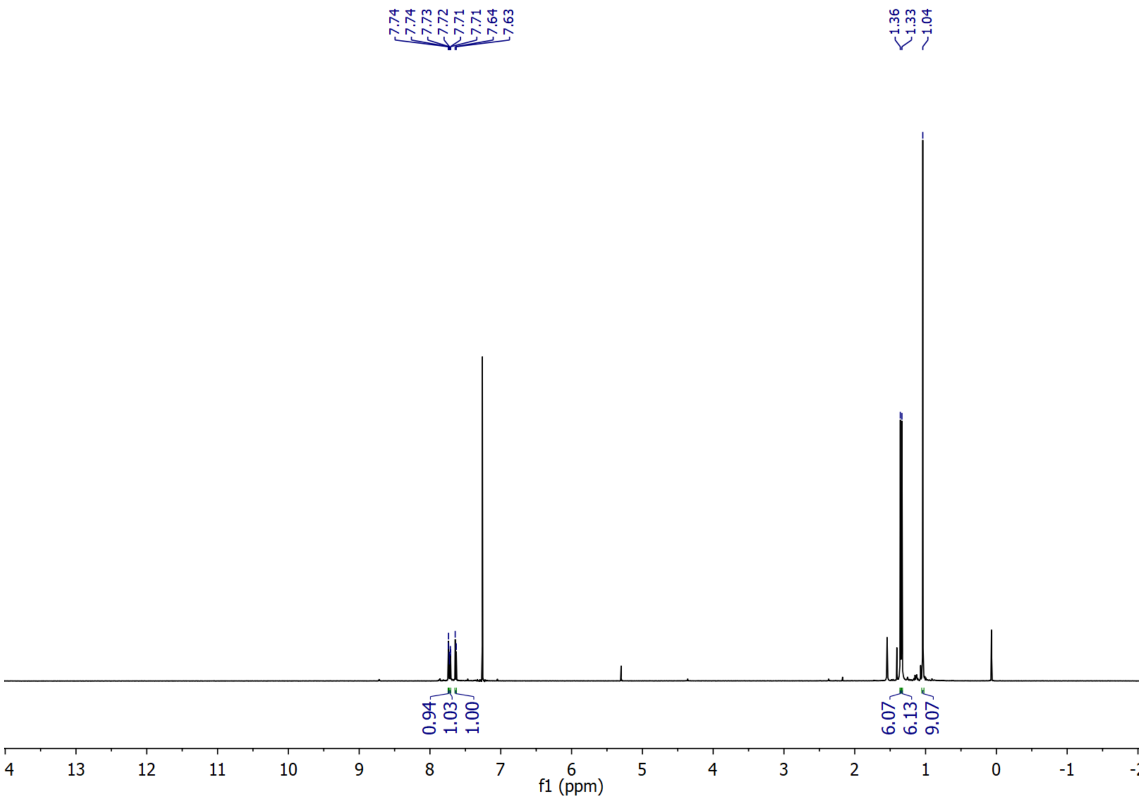

Figure S 112. Full ^{1}H-NMR (500 MHz, $CDCl_3$) spectrum of compound **12b**.

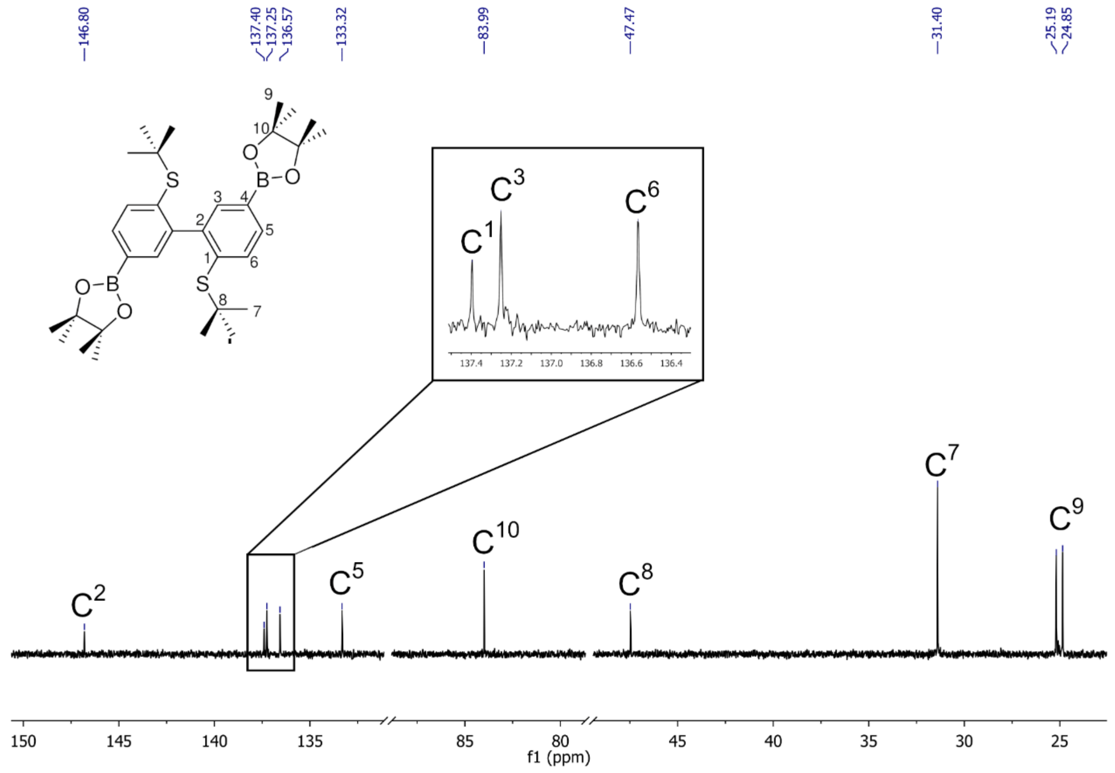

Figure S 113. $^{13}C\{^{1}H\}$-NMR (126 MHz, $CDCl_3$) spectrum of compound **12b**.

.

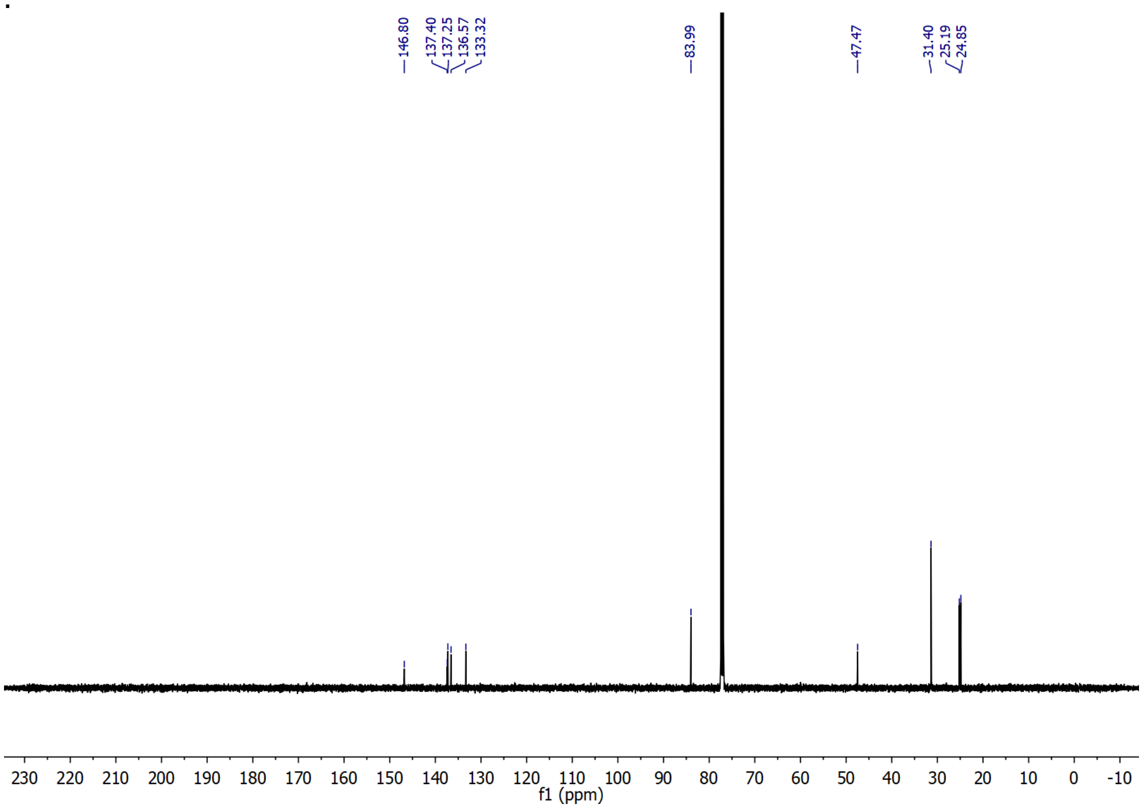

Figure S 114. Full $^{13}C\{^{1}H\}$-NMR (126 MHz, $CDCl_3$) spectrum of compound **12b**.

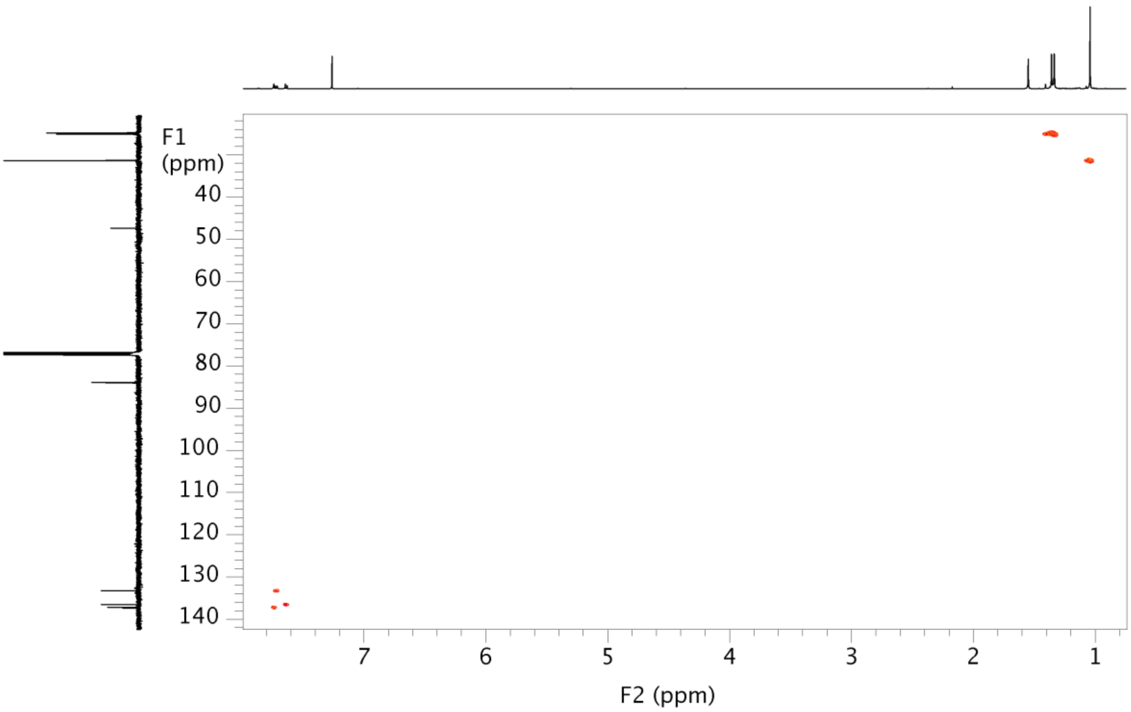

Figure S 115. ^{1}H-^{13}C gc2HSQC (400 MHz, $CDCl_3$) spectrum of compound **12b**.

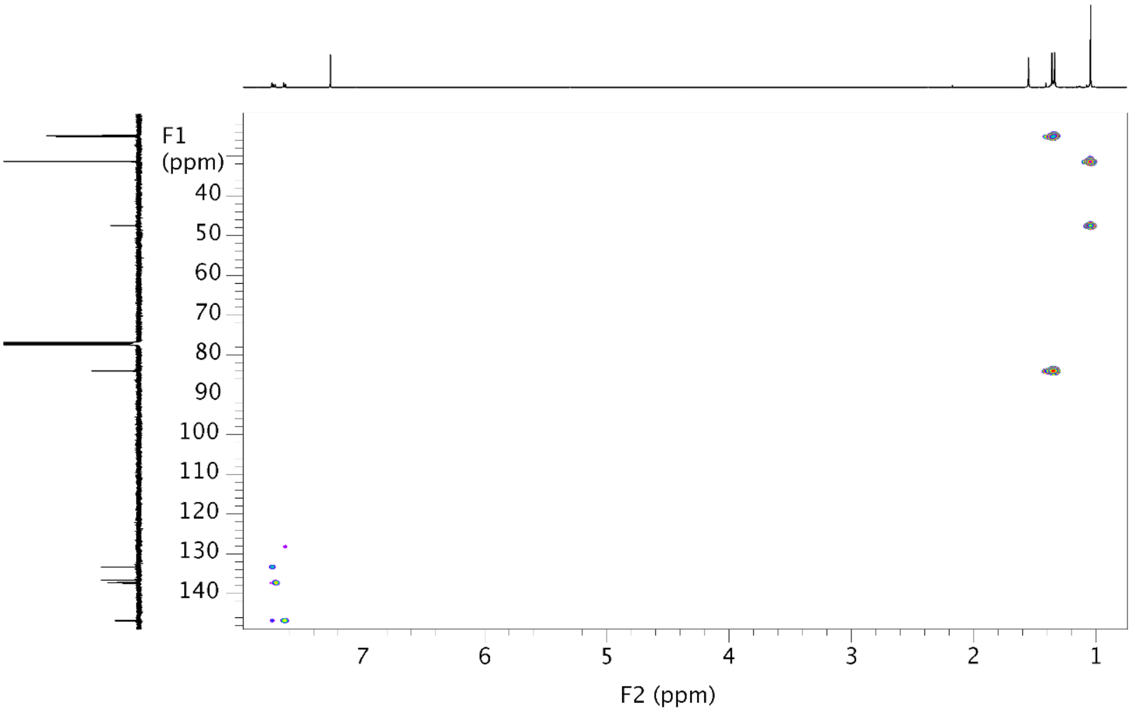

Figure S 116. ^{1}H-^{13}C gc2HMBC (400 MHz, $CDCl_3$) spectrum of compound **12b**.

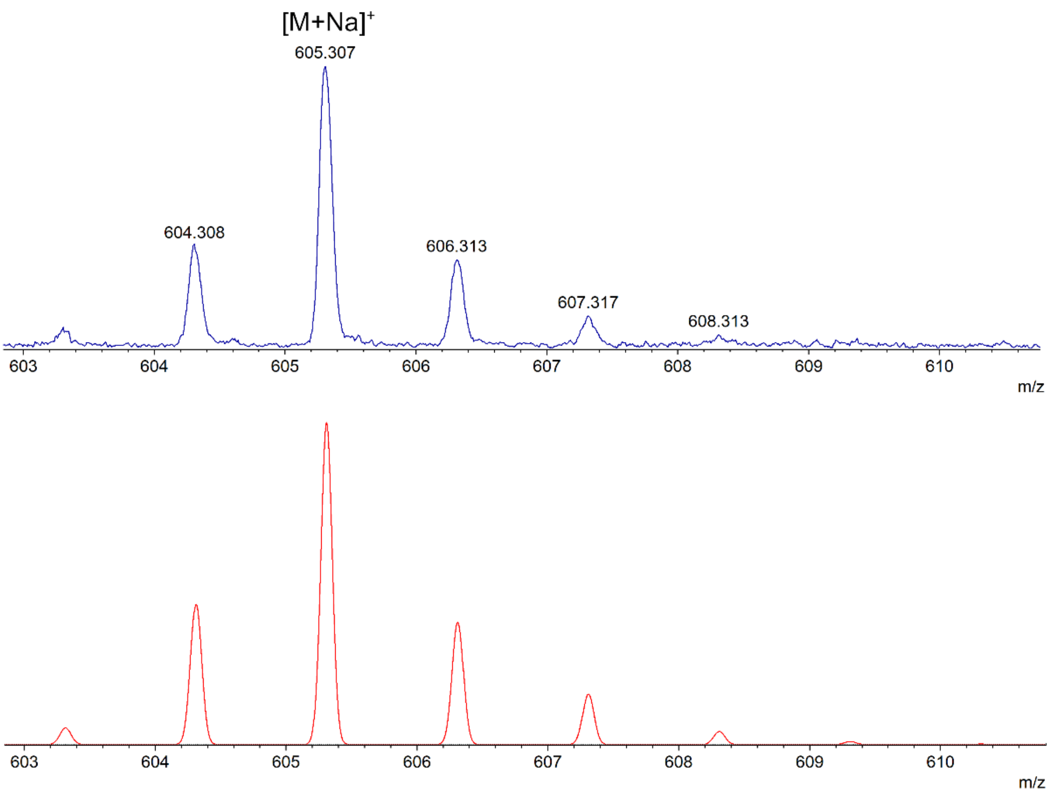

Figure S 117. HRMS (MALDI-TOF), of compound 12b $[M+Na]^+$. Calculated (red), measured (blue).

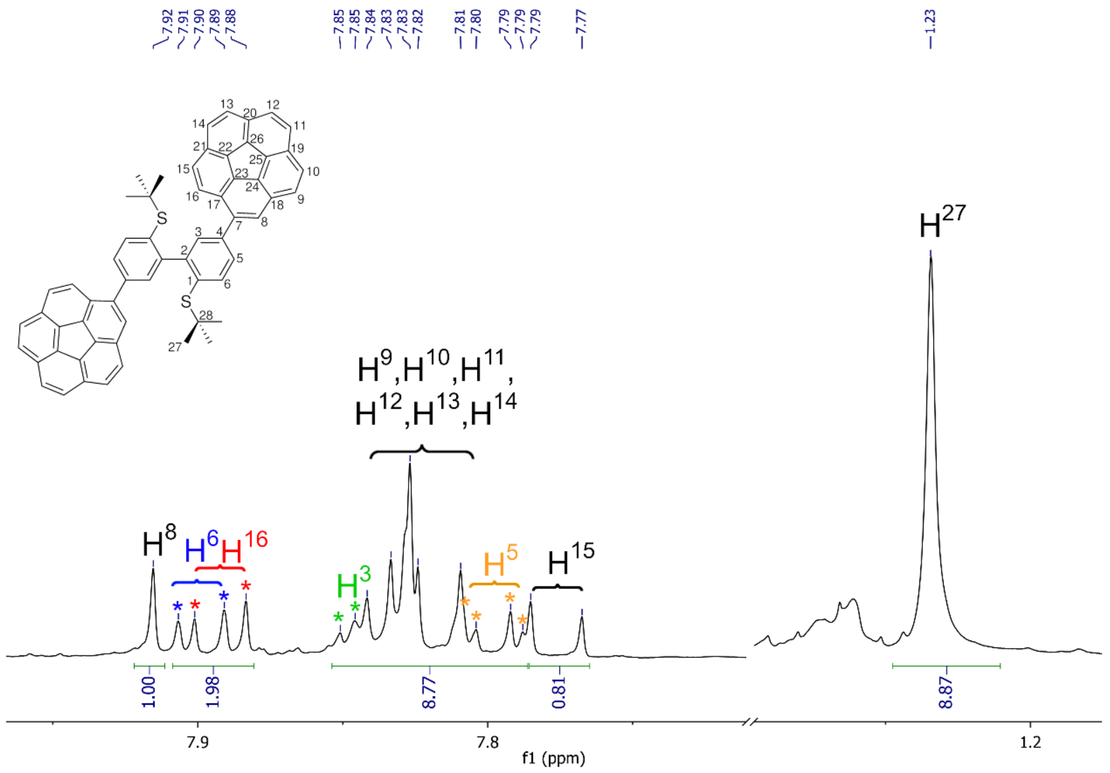

Figure S 118. ^{1}H-NMR (500 MHz, $CDCl_3$) spectrum of compound **13-S^{t}Bu**.

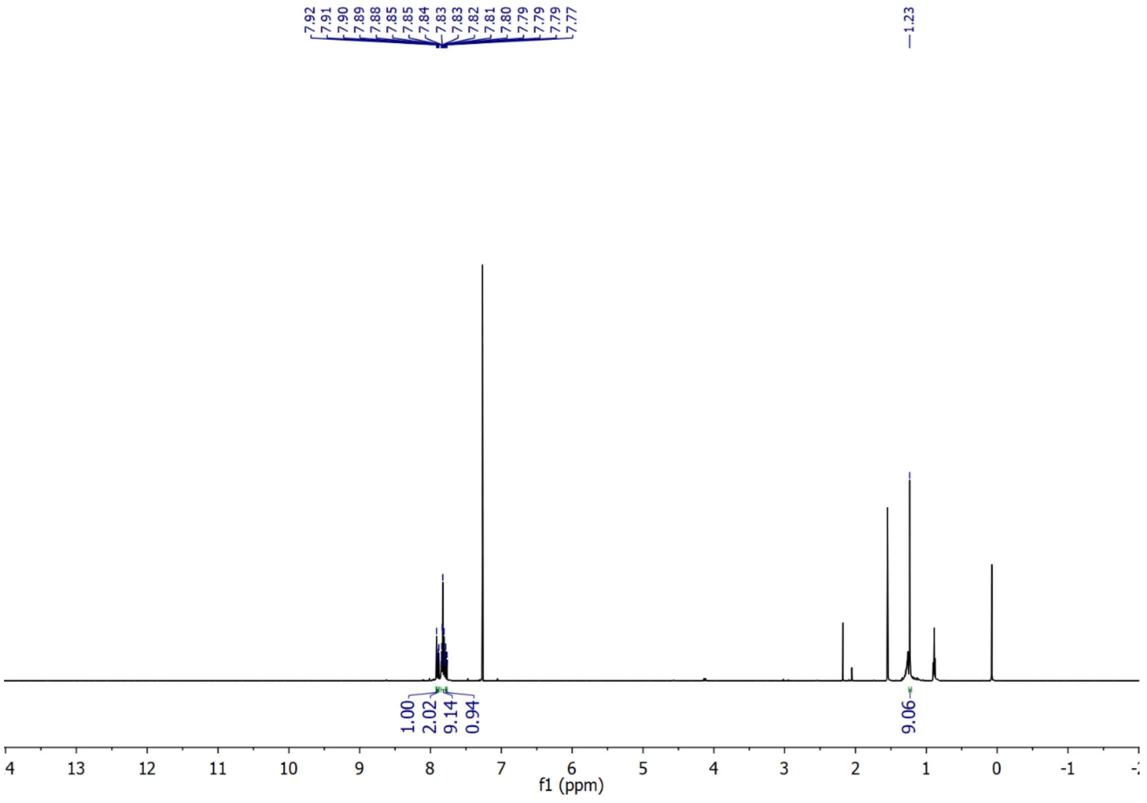

Figure S 119. Full ^{1}H-NMR (500 MHz, $CDCl_3$) spectrum of compound **13-S^{t}Bu**.

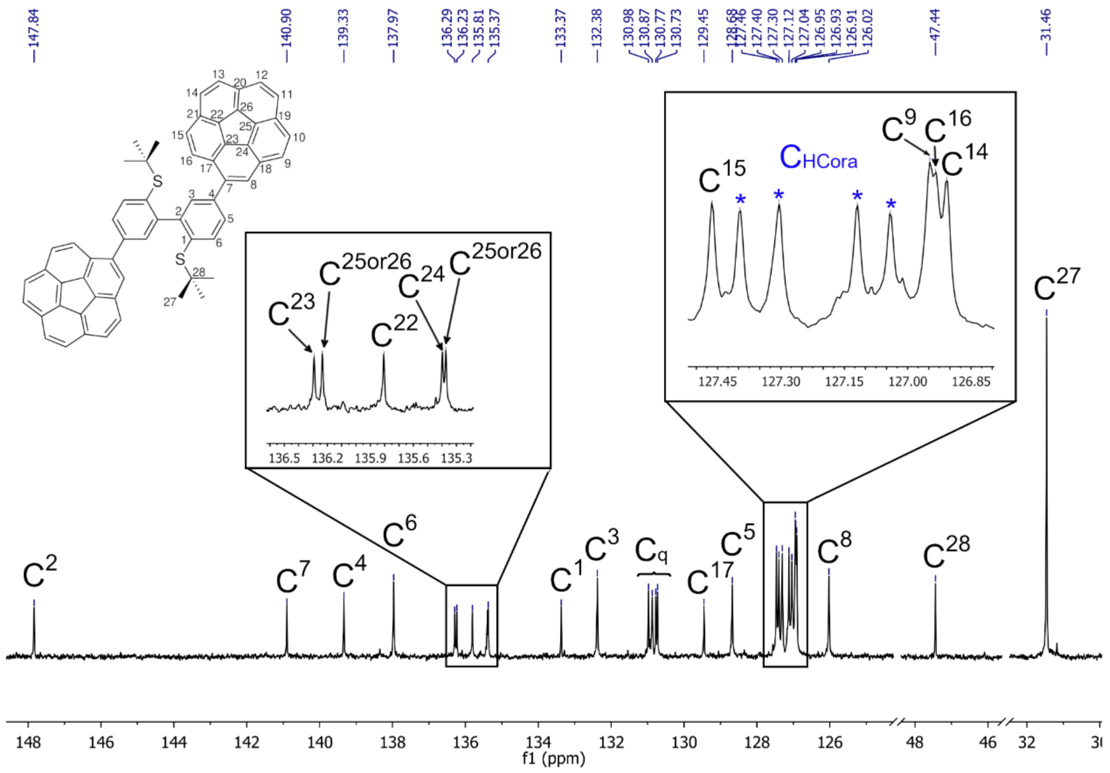

Figure S 120. $^{13}C\{^1H\}$-NMR (101 MHz, $CDCl_3$) spectrum of compound **13-S^{t}Bu**.

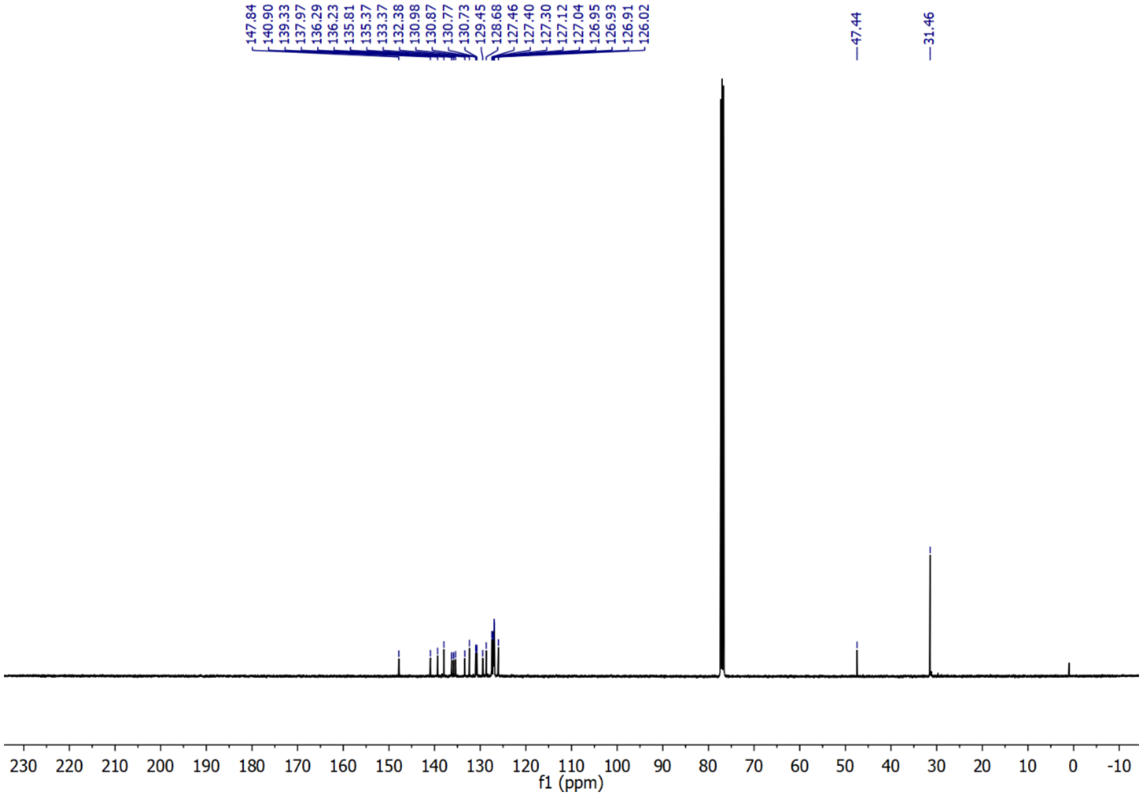

Figure S 121. Full $^{13}C\{^1H\}$-NMR (101 MHz, $CDCl_3$) spectrum of compound **13-S^{t}Bu**.

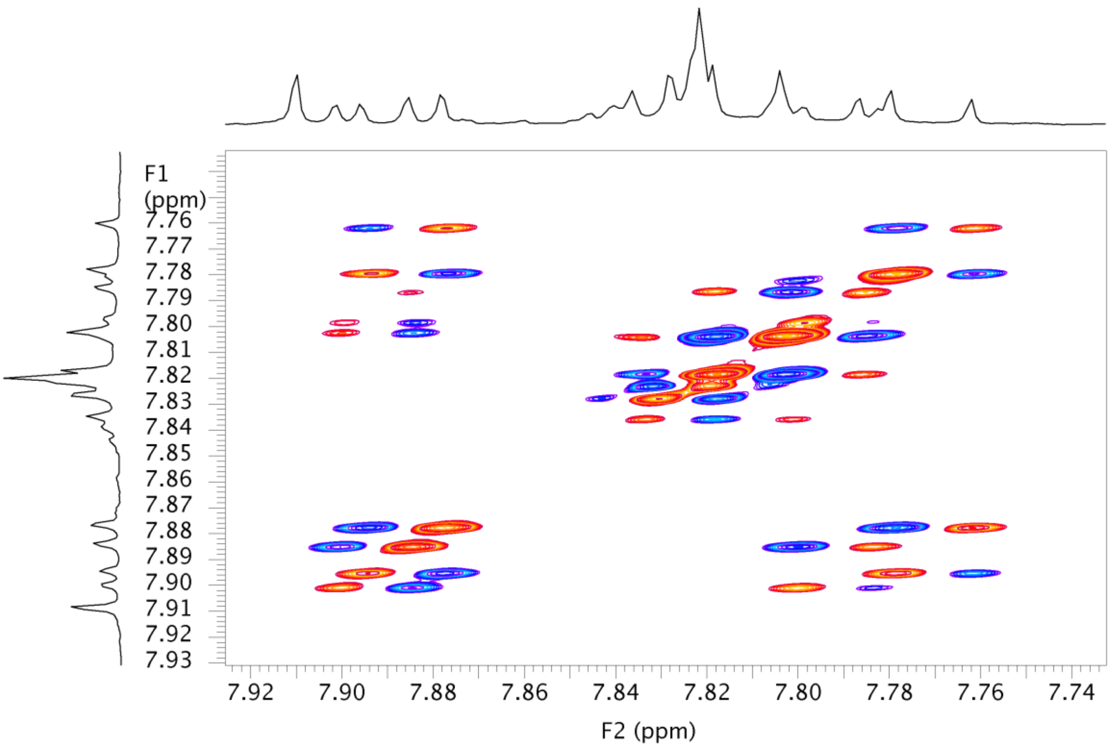

Figure S 122. ^{1}H-^{1}H gDQFCOSY (500 MHz, $CDCl_3$) spectrum of compound **13-S^{t}Bu**.

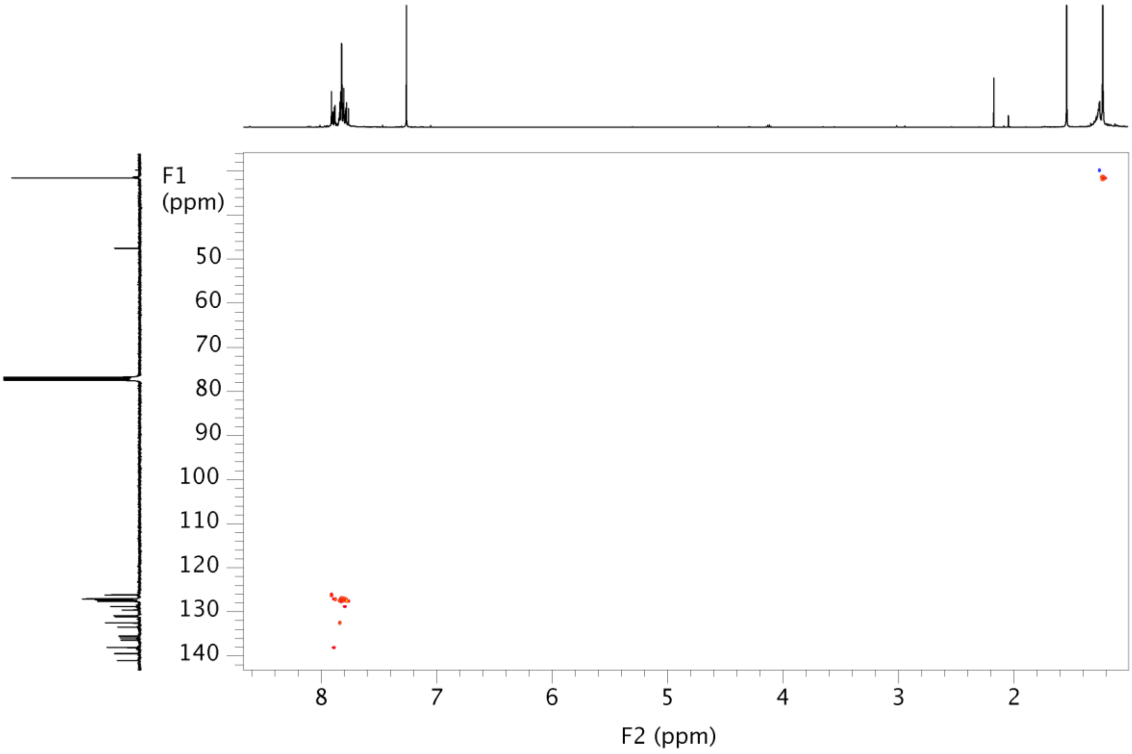

Figure S 123. ^{1}H-^{13}C gc2HSQC (500 MHz, $CDCl_3$) spectrum of compound **13-S^{t}Bu**.

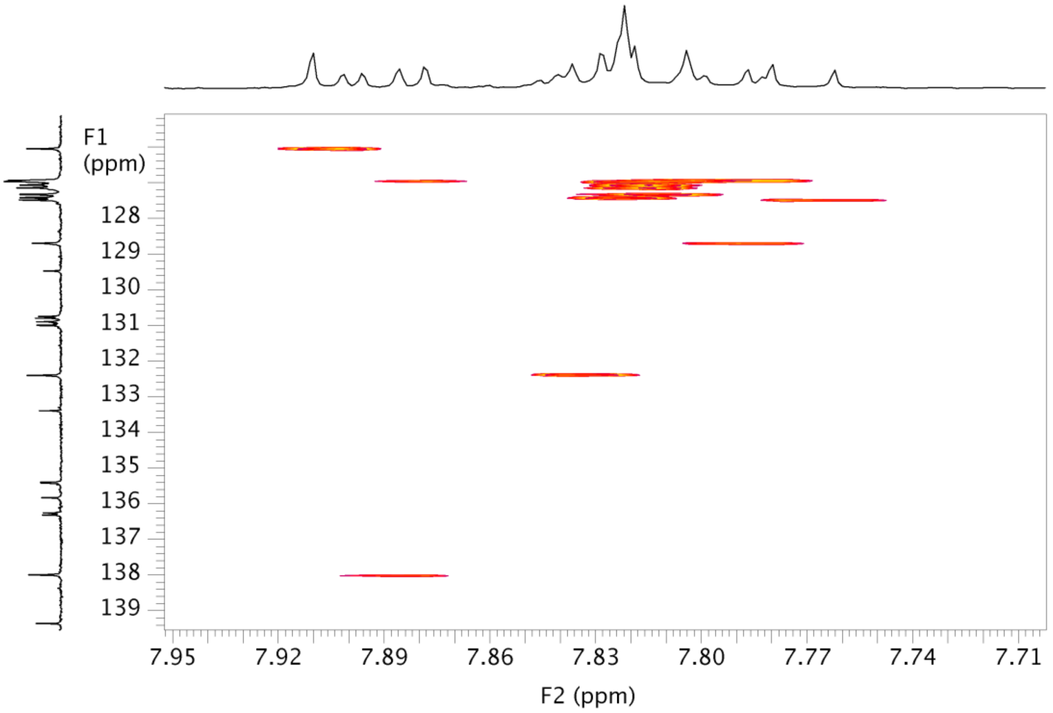

Figure S 124. ^{1}H-^{13}C bsgHSQC (500 MHz, $CDCl_3$) spectrum of compound **13-S^{t}Bu**.

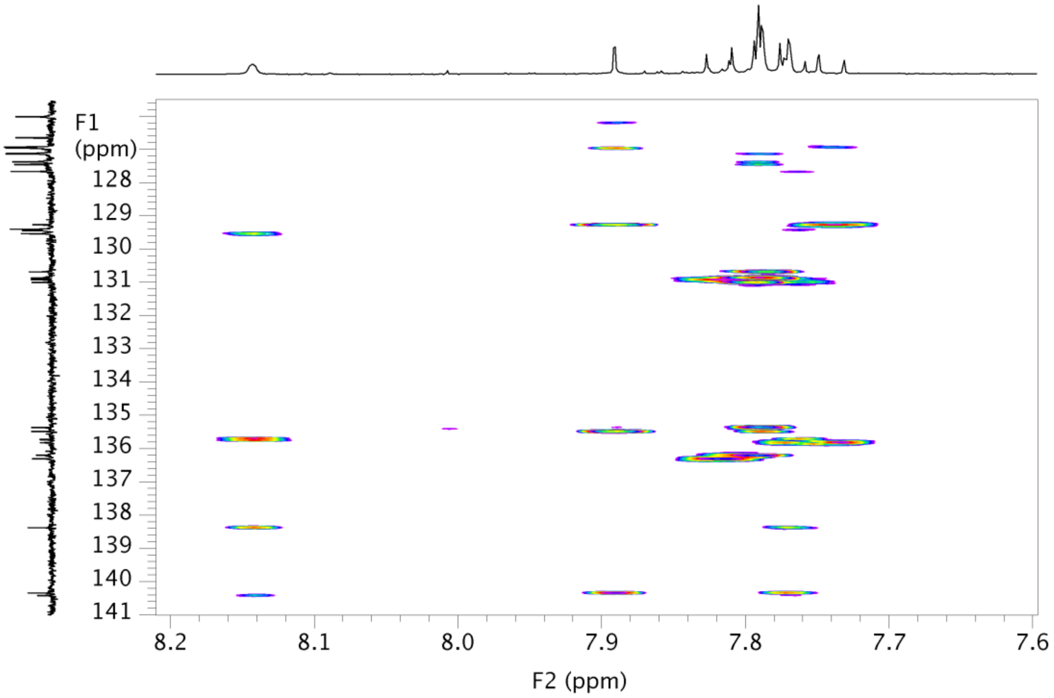

Figure S 125. ^{1}H-^{13}C bsgHMBC (500 MHz, $CDCl_3$) spectrum of compound **13-S^{t}Bu**.

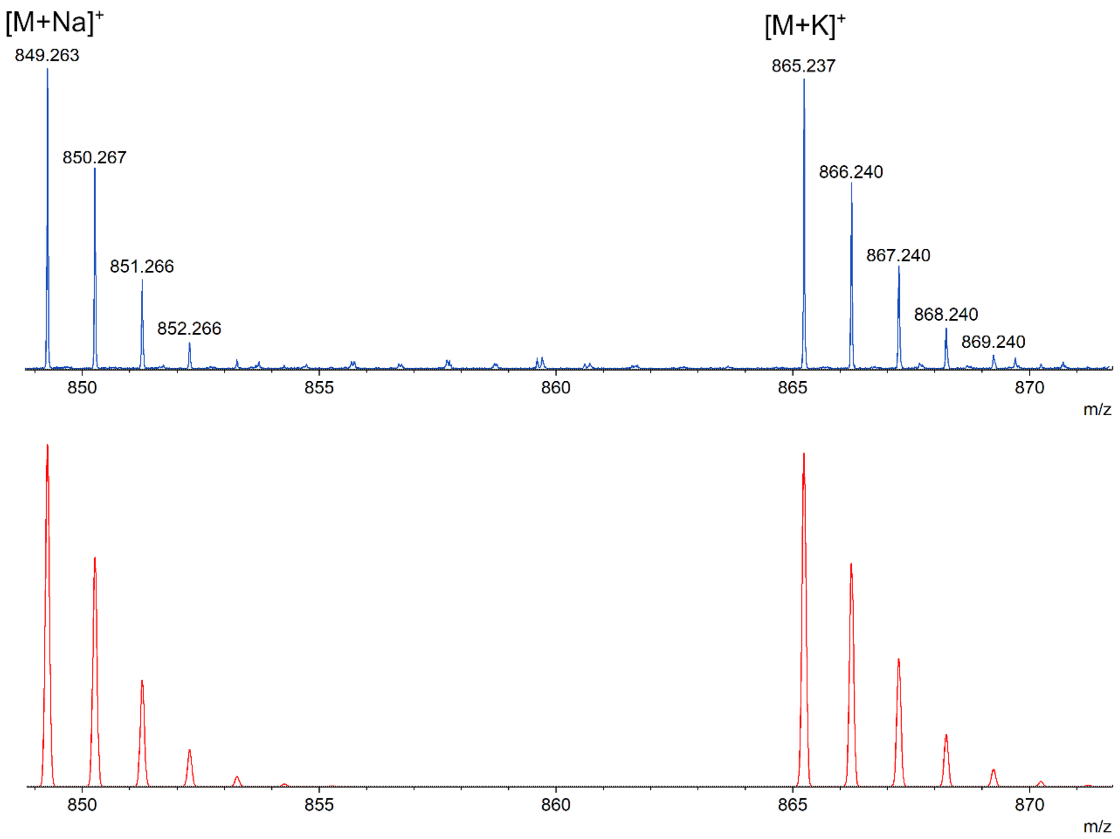

Figure S 126. HRMS (ESI-TOF), of compound **13-S^{t}Bu**, $[M+Na]^+$ and $[M+K]^+$. Calculated (red), measured (blue).

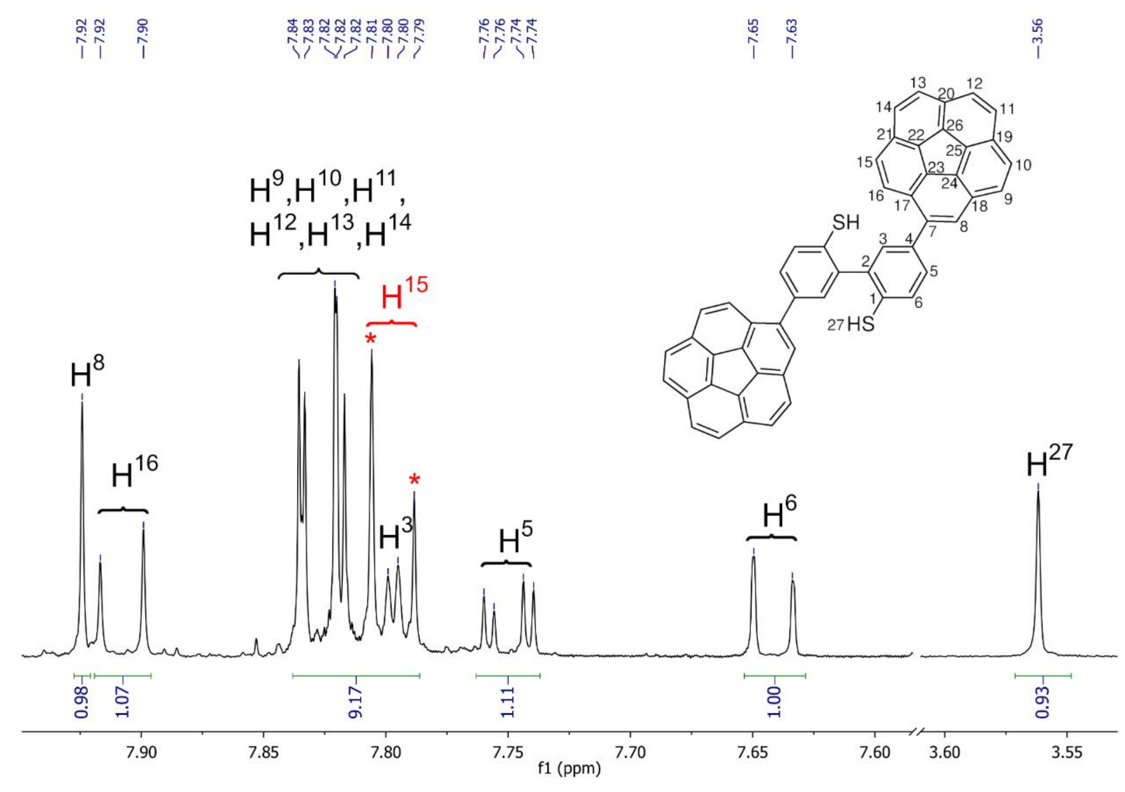

Figure S 127. ^{1}H-NMR (500 MHz, $CDCl_3$) spectrum of compound **13-SH**.

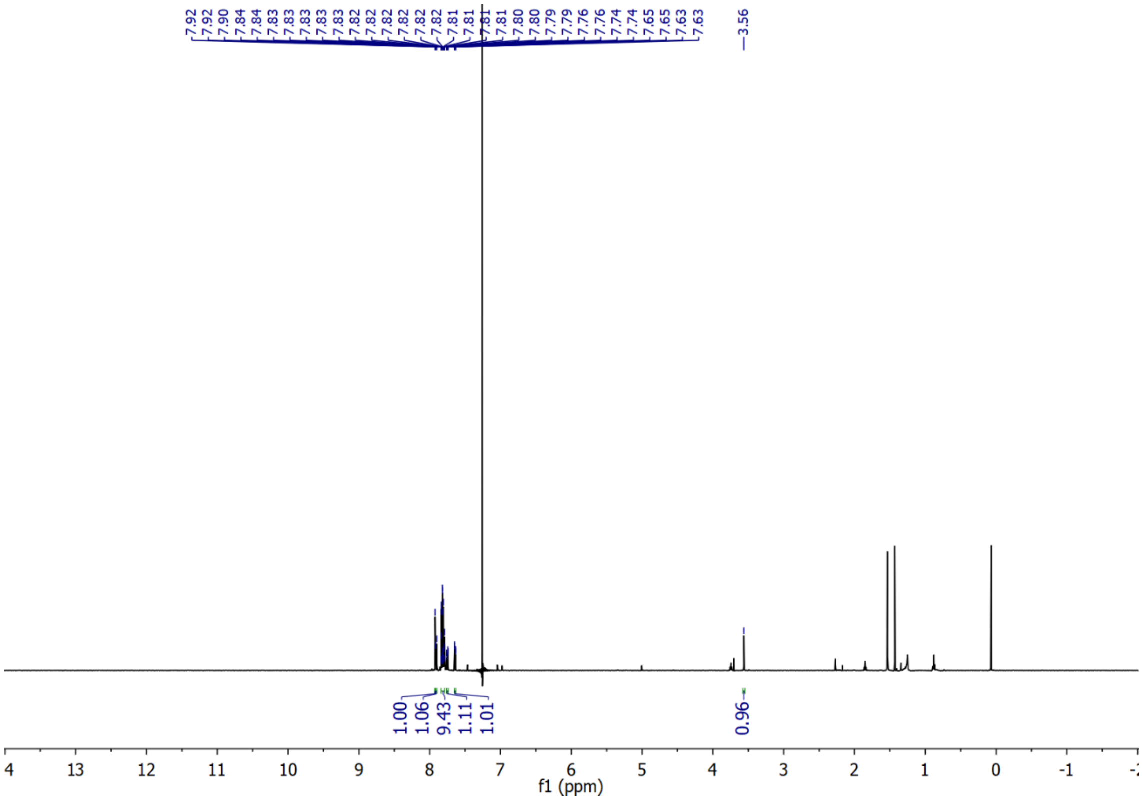

Figure S 128. Full ^{1}H-NMR (500 MHz, $CDCl_3$) spectrum of compound **13-SH**.

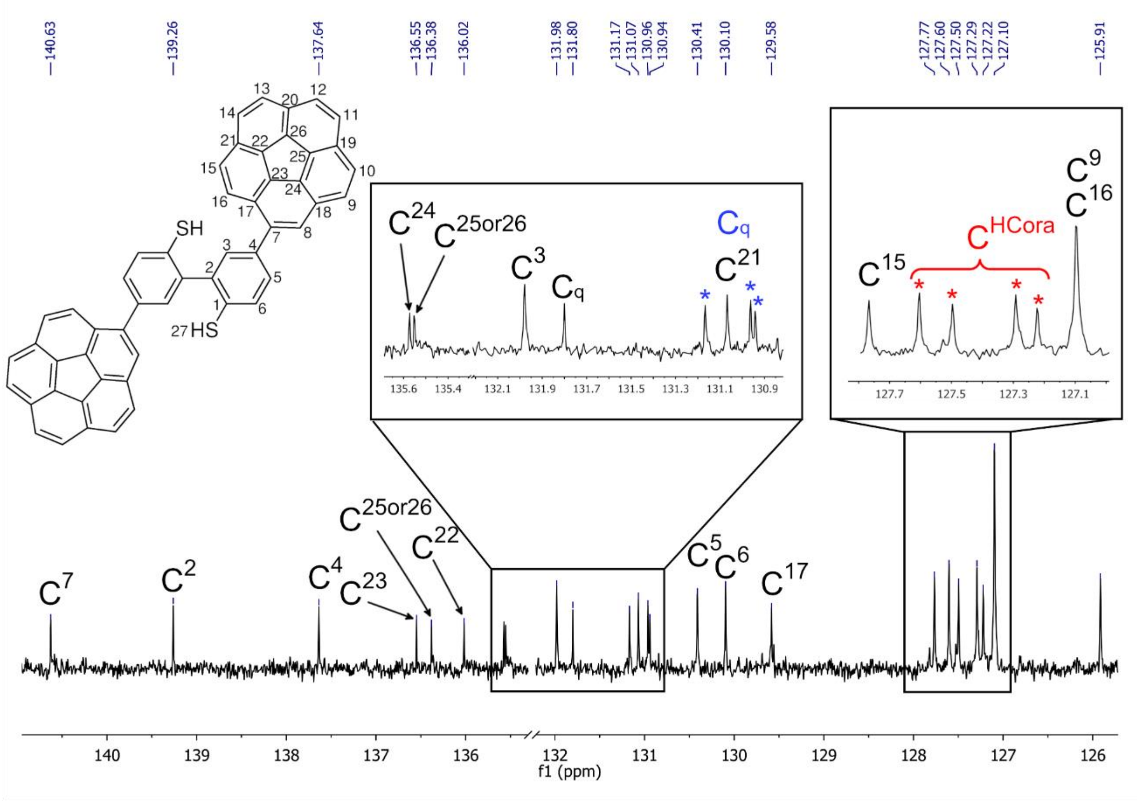

Figure S 129. $^{13}C\{^1H\}$-NMR (126 MHz, $CDCl_3$) spectrum of compound **13-SH**.

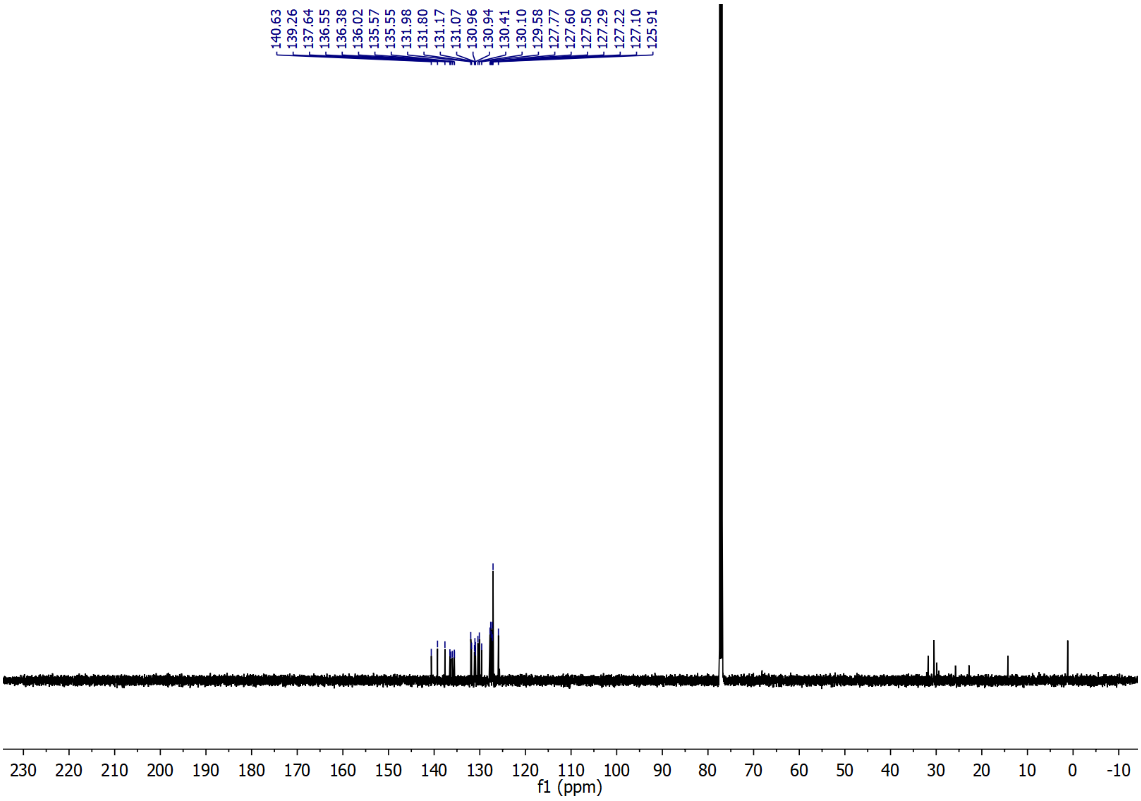

Figure S 130. Full $^{13}C\{^1H\}$-NMR (126 MHz, $CDCl_3$) spectrum of compound **13-SH**.

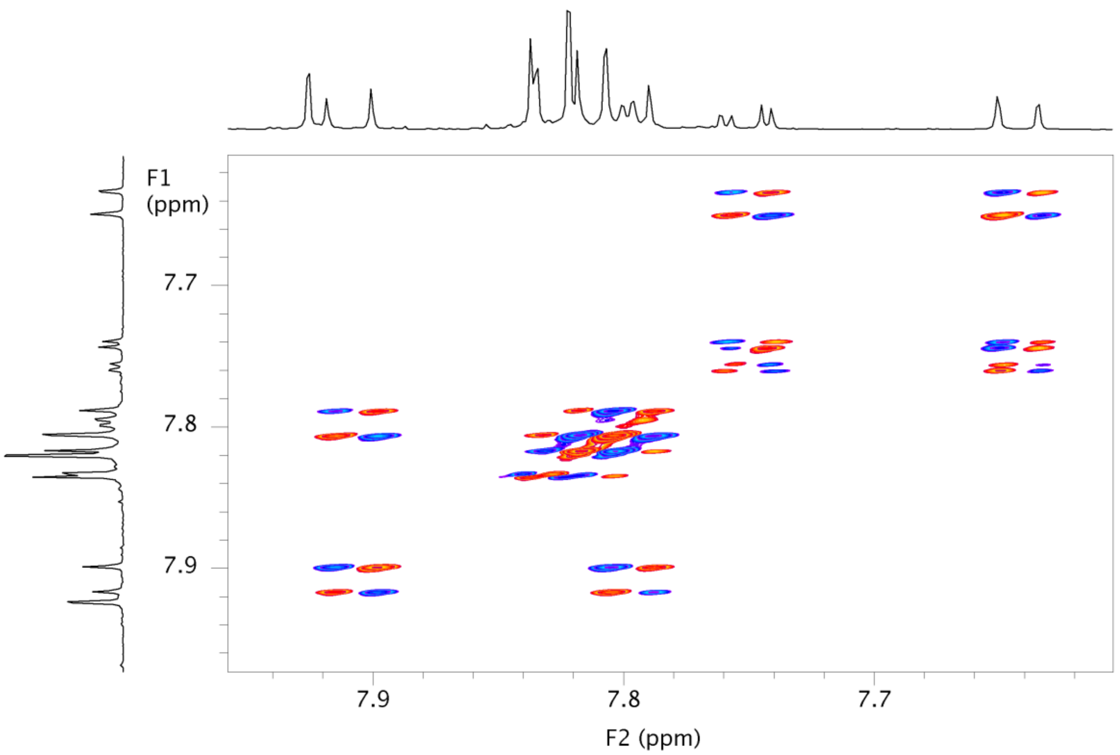

Figure S 131. ^{1}H-^{1}H gDQFCOSY (500 MHz, $CDCl_3$) spectrum of compound **13-SH**.

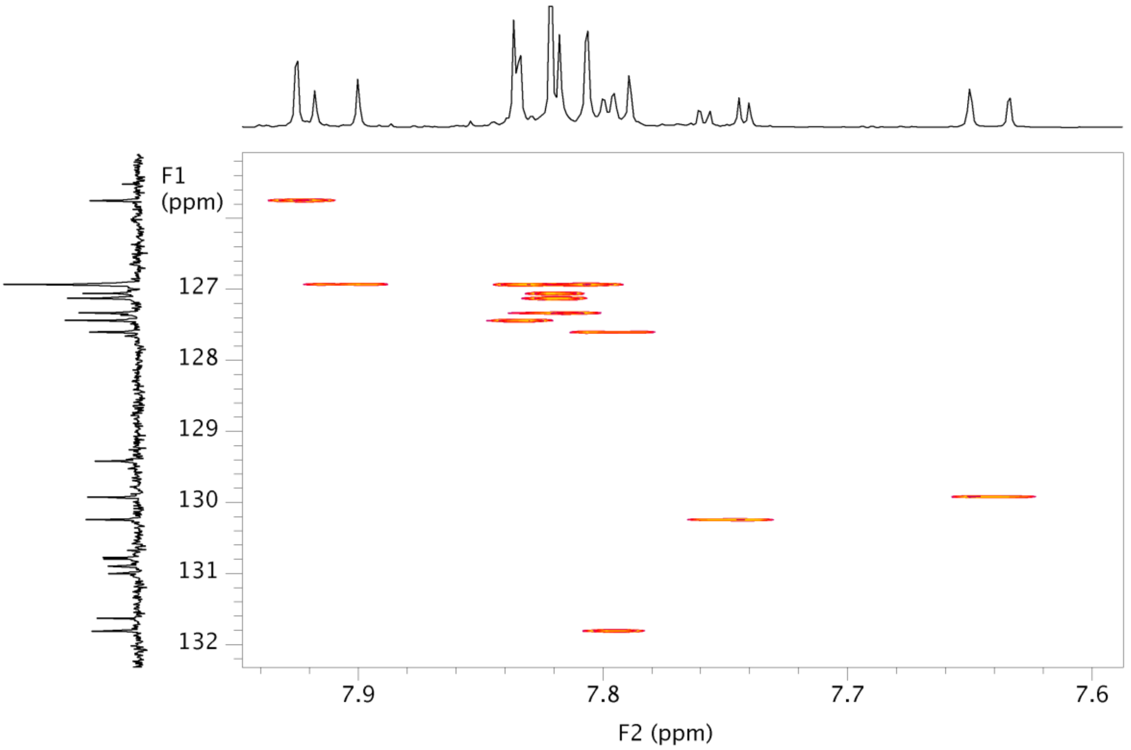

Figure S 132. ^{1}H-^{13}C bsgHSQC (500 MHz, $CDCl_3$) spectrum of compound **13-SH**.

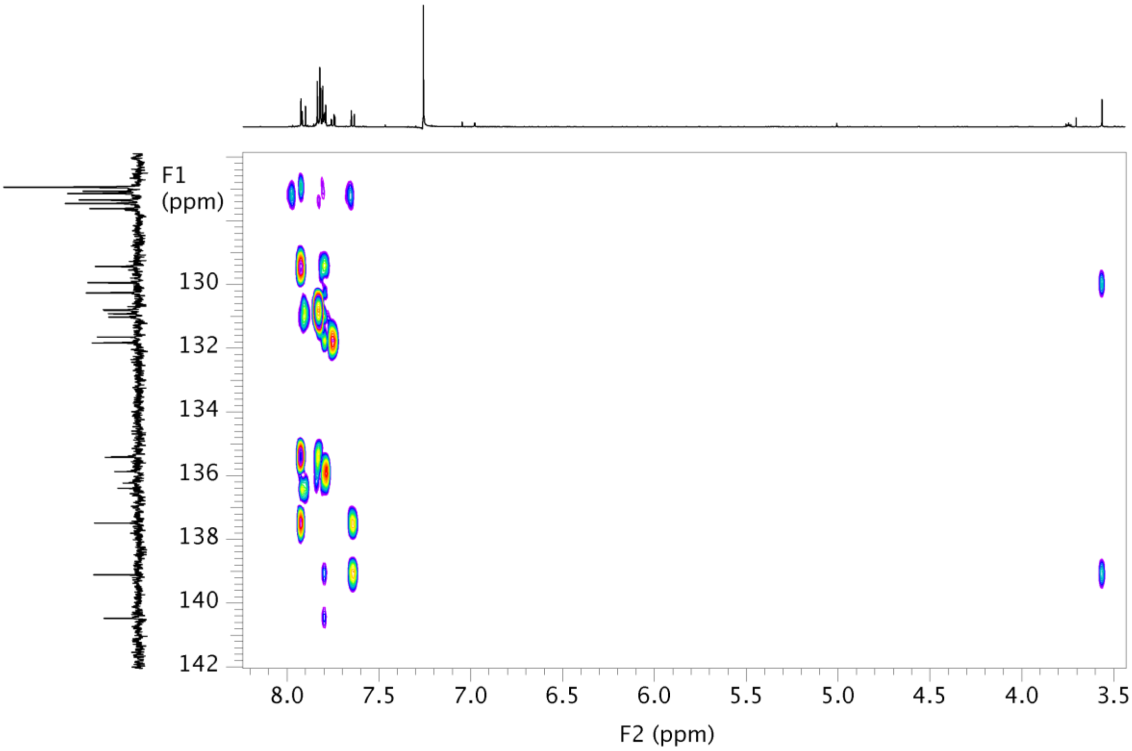

Figure S 133. ^{1}H-^{13}C gc2HMBC (500 MHz, $CDCl_3$) spectrum of compound **13-SH**.

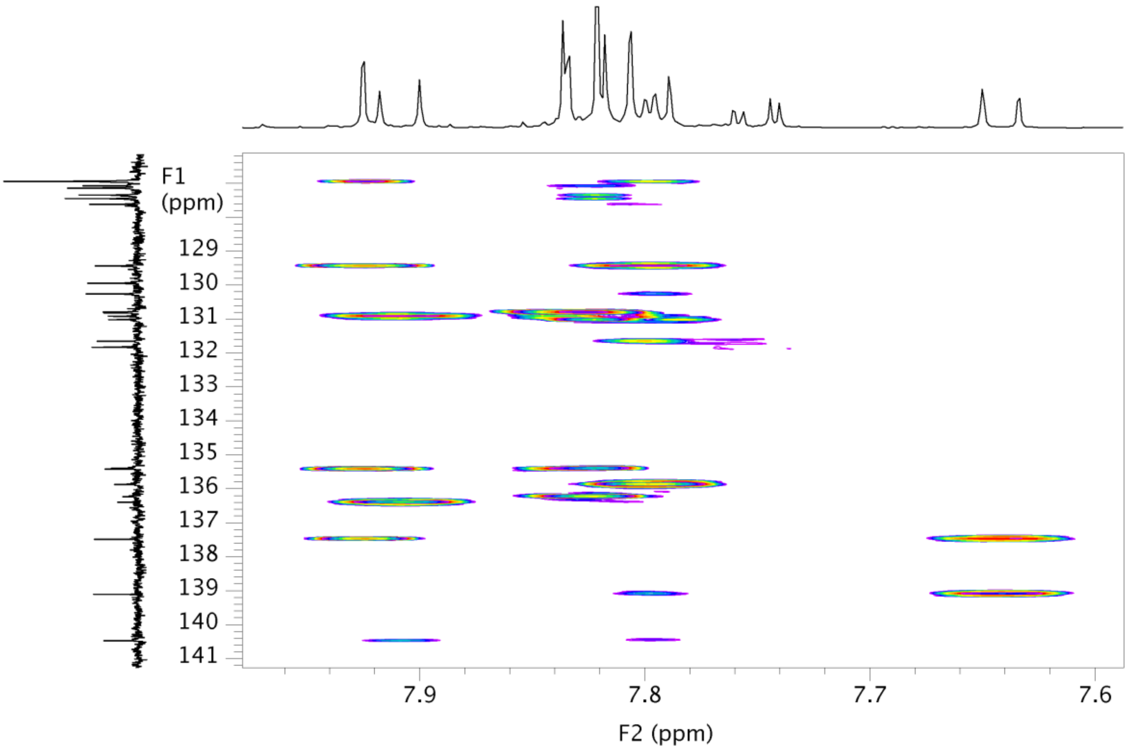

Figure S 134. ^{1}H-^{13}C bsgHMBC (500 MHz, $CDCl_3$) spectrum of compound **13-SH**.

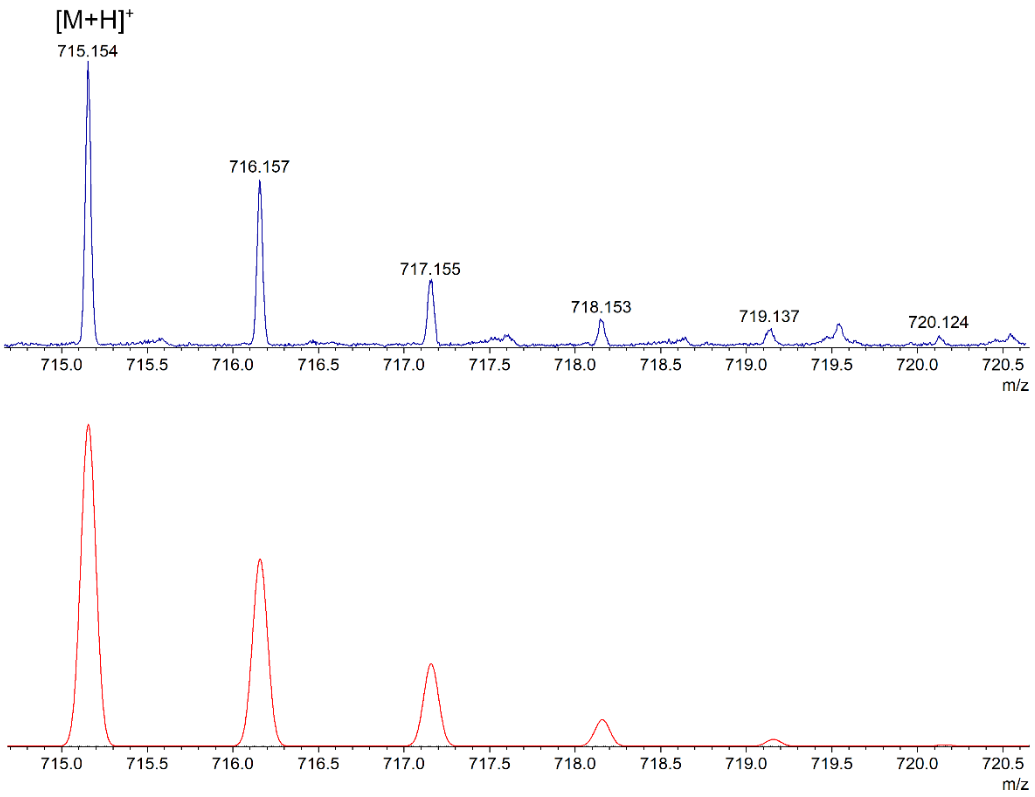

Figure S 135. HRMS (ESI-TOF), of compound **13-SH** $[M+H]^+$. Calculated (red), measured (blue).

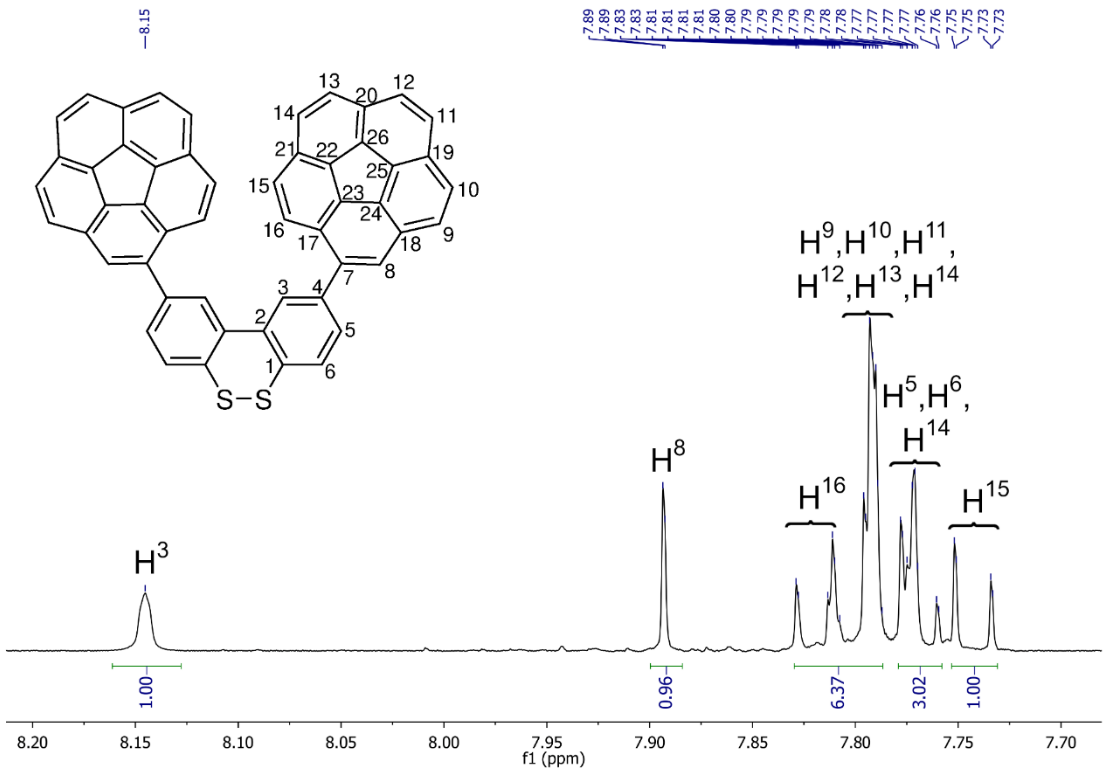

Figure S 136. ^{1}H-NMR (500 MHz, $CDCl_3$) spectrum of compound **13-SS**.

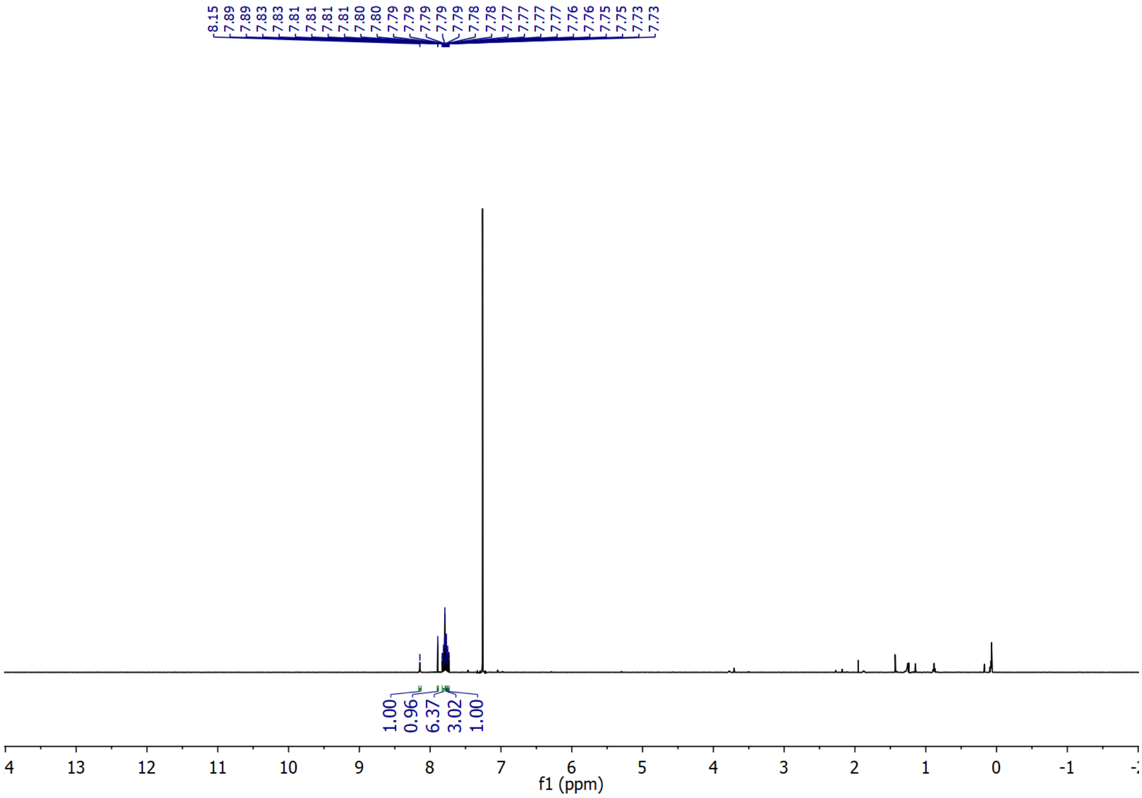

Figure S 137. Full ^{1}H-NMR (500 MHz, $CDCl_3$) spectrum of compound **13-SS**.

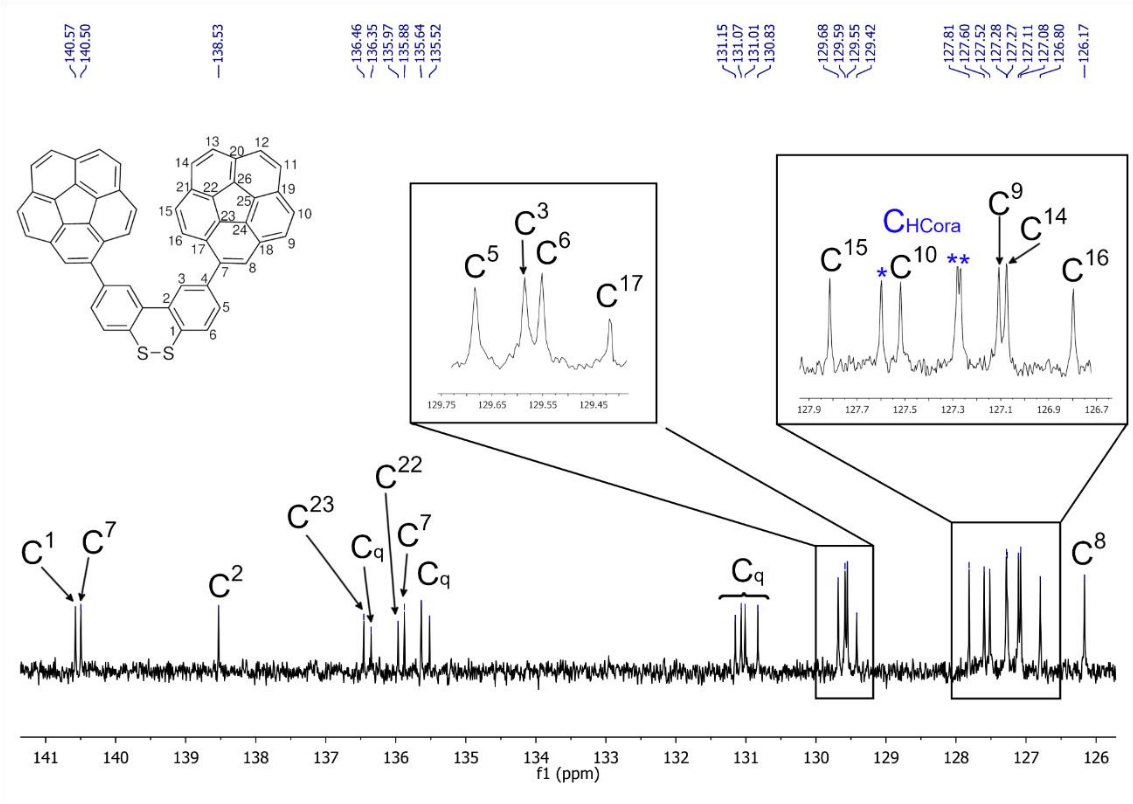

Figure S 138. $^{13}C\{^1H\}$-NMR (126 MHz, $CDCl_3$) spectrum of compound **13-SS**.

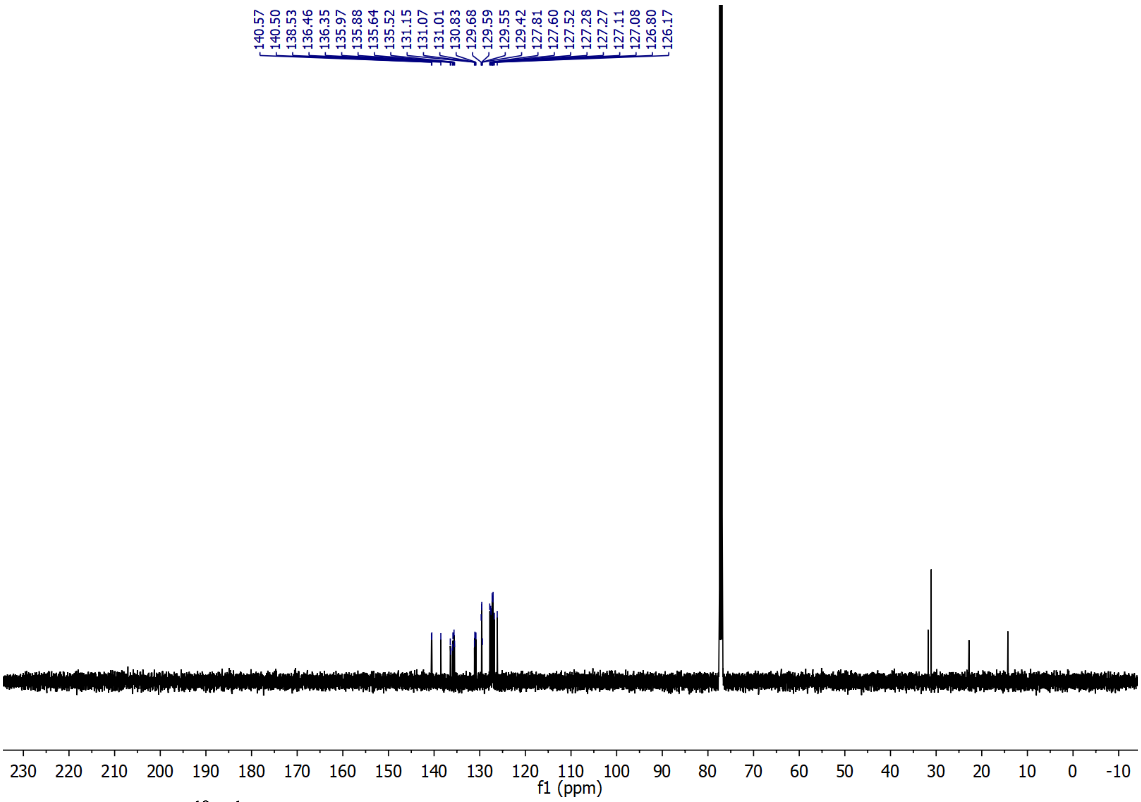

Figure S 139. Full $^{13}C\{^1H\}$-NMR (126 MHz, $CDCl_3$) spectrum of compound **13-SS**.

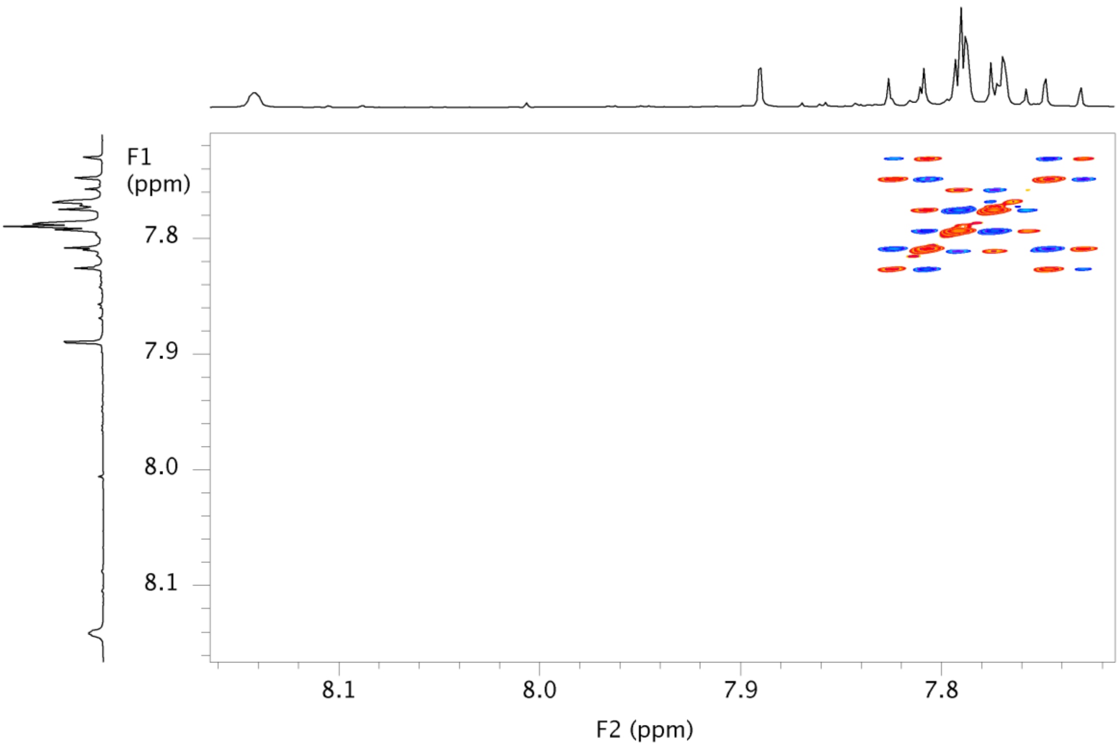

Figure S 140. ^{1}H-^{1}H gDQFCOSY (500 MHz, $CDCl_3$) spectrum of compound **13-SS**.

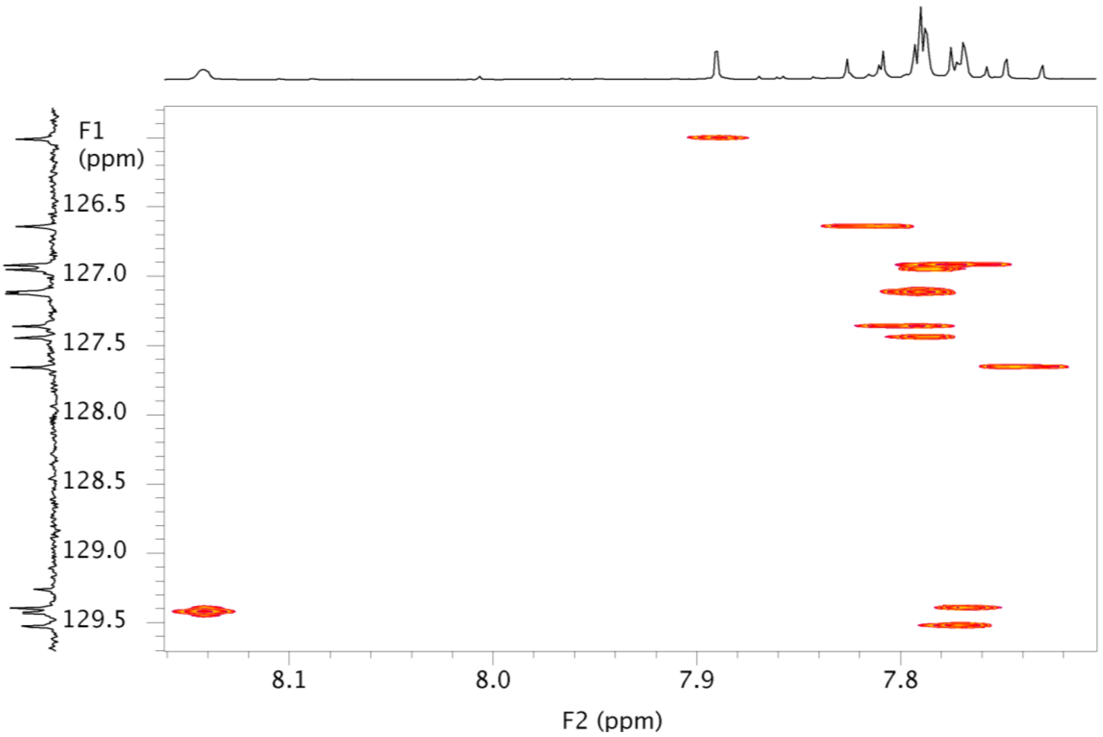

Figure S 141. ^{1}H-^{13}C bsgHSQCAD (500 MHz, $CDCl_3$) spectrum of compound **13-SS**.

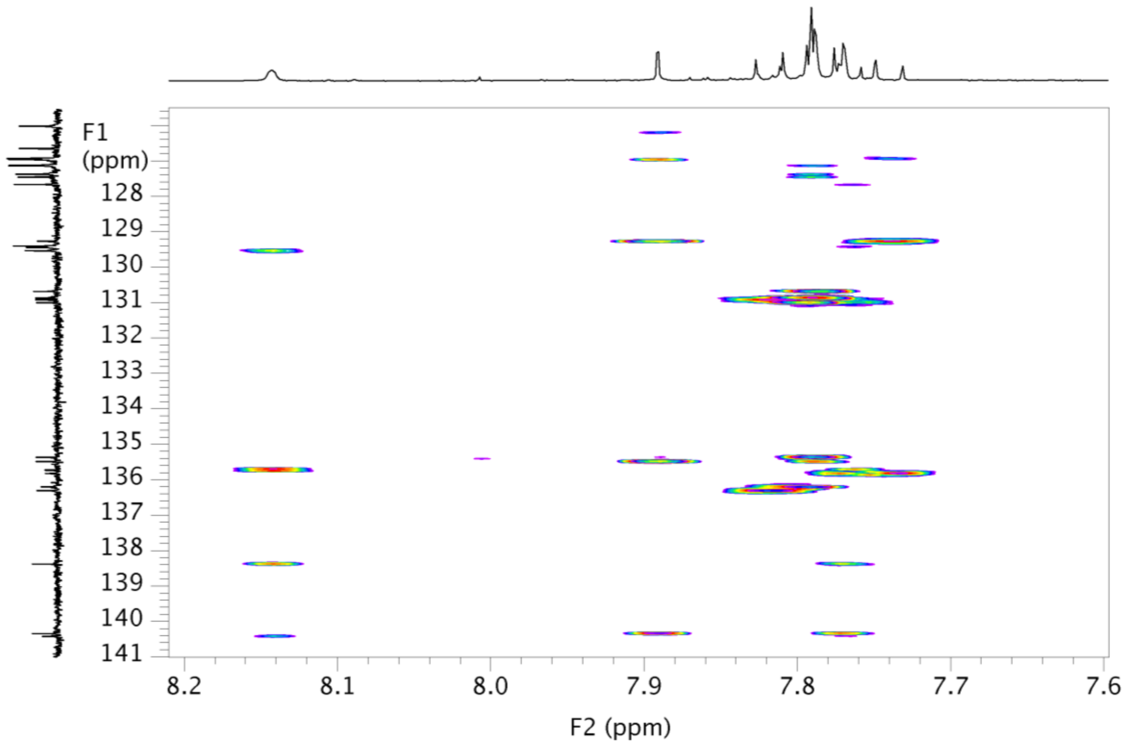

Figure S 142. ^{1}H-^{13}C bsgHMBC (500 MHz, $CDCl_3$) spectrum of compound **13-SS**.

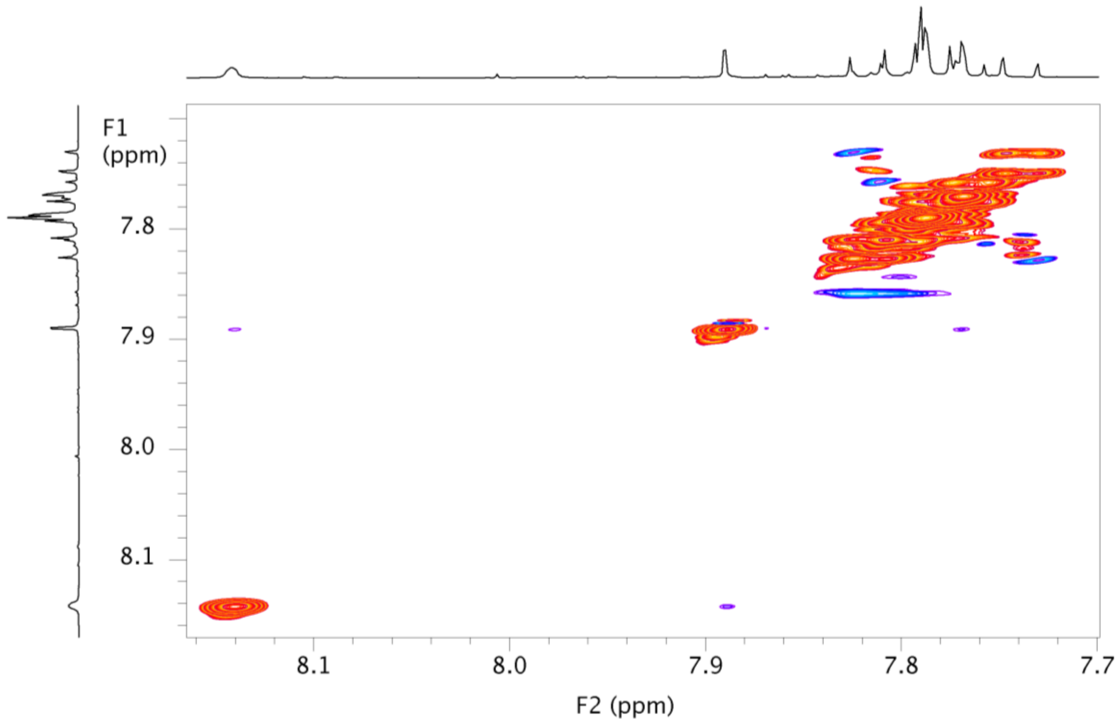

Figure S 143. ^{1}H-^{1}H NOESY (500 MHz, $CDCl_3$) spectrum of compound **13-SS**. Mixing time: 800 ms.

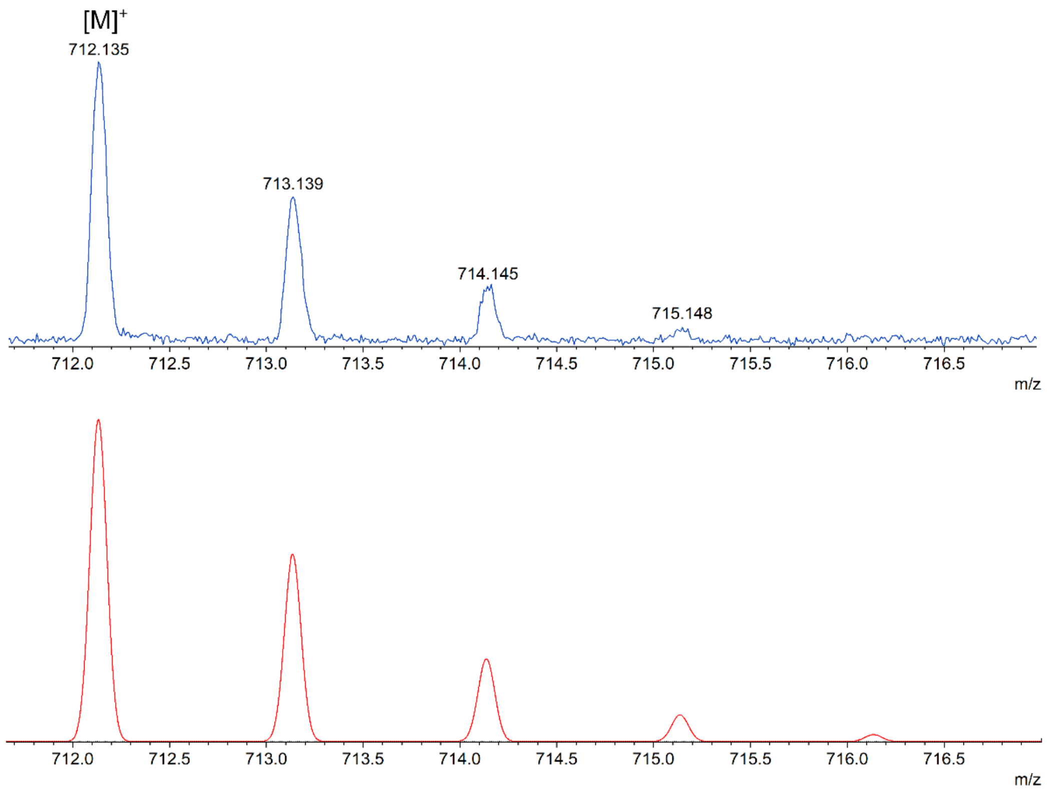

Figure S 144. HRMS (MALDI-TOF), of compound **13-SS** [M]$^+$. Calculated (red), measured (blue).

UV-Vis absorption and emission spectra

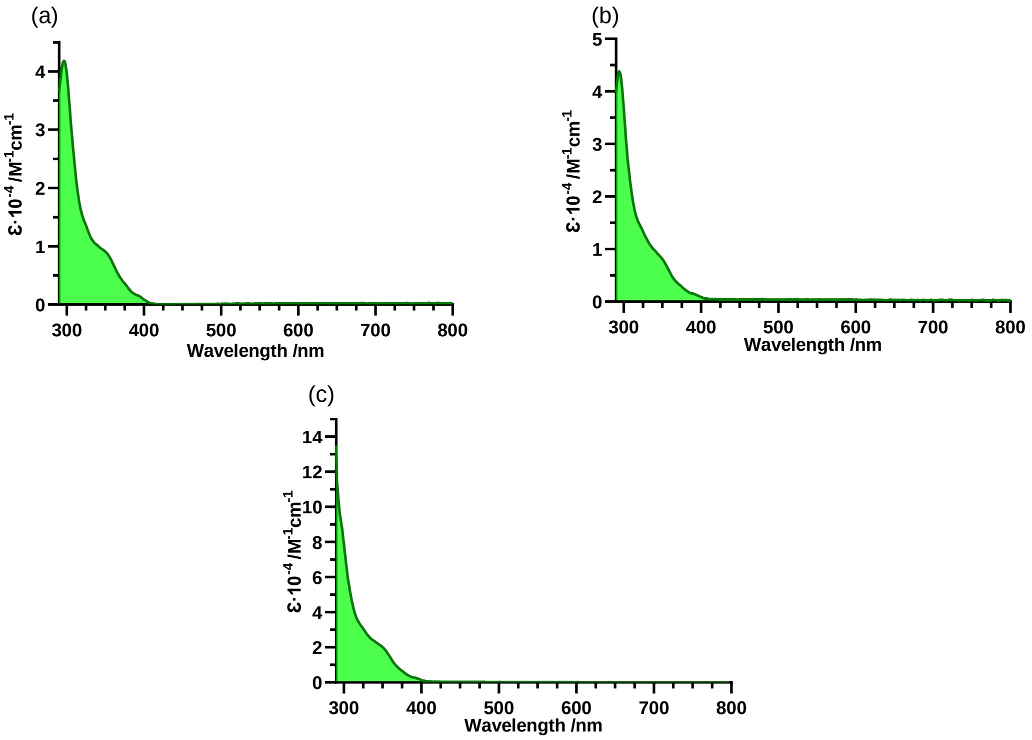

Figure S 145. UV-Vis absorption spectra of compounds (a) **4** (10^{-5} M), (b) **5-SH** (10^{-5} M) and (c) **5_2-SS** (10^{-5} M) in toluene.

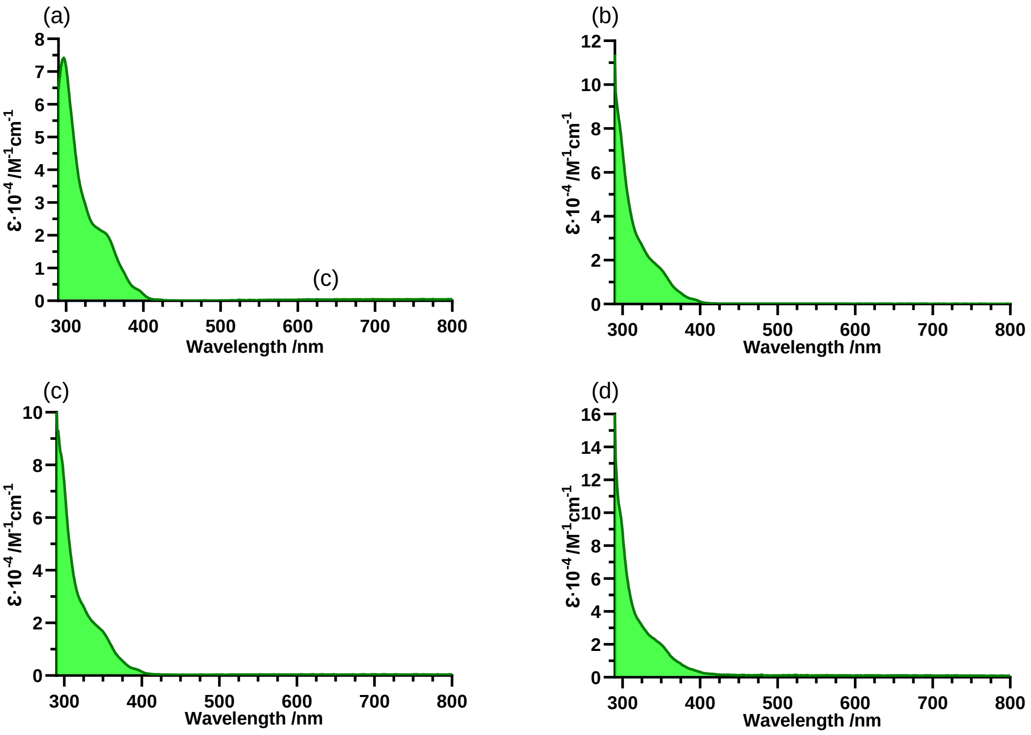

Figure S 146. UV-Vis absorption spectra of compounds (a) **13-SMe** (10^{-5} M), (b) **13-S^{t}Bu** (10^{-5} M), (c) **13-SH** (10^{-5} M) and (d) **13-SS** (10^{-5} M) in toluene.

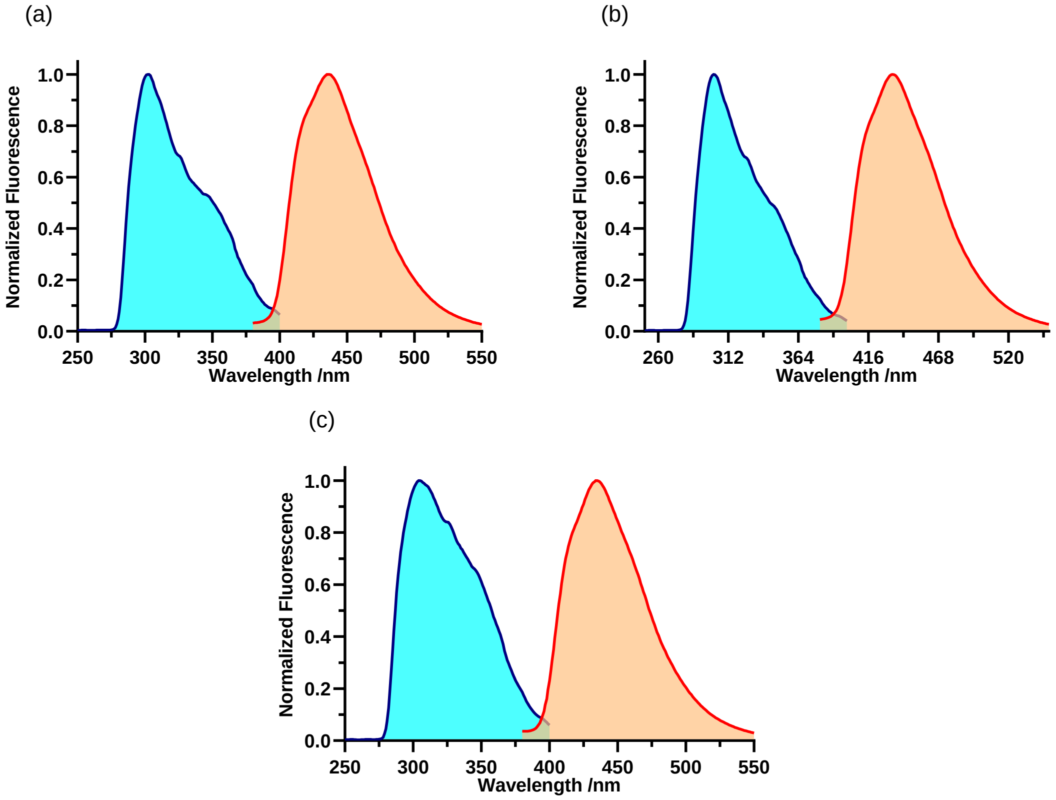

Figure S 147. Normalized excitation (blue) and emission (red) spectra in toluene of: (a) **4** (10^{-5} M) (λ_{exc} = 302 nm), (b) **5-SH** (10^{-5} M) (λ_{exc} = 301 nm) and (c) **5_2-SS** (10^{-5} M) (λ_{exc} = 305 nm).

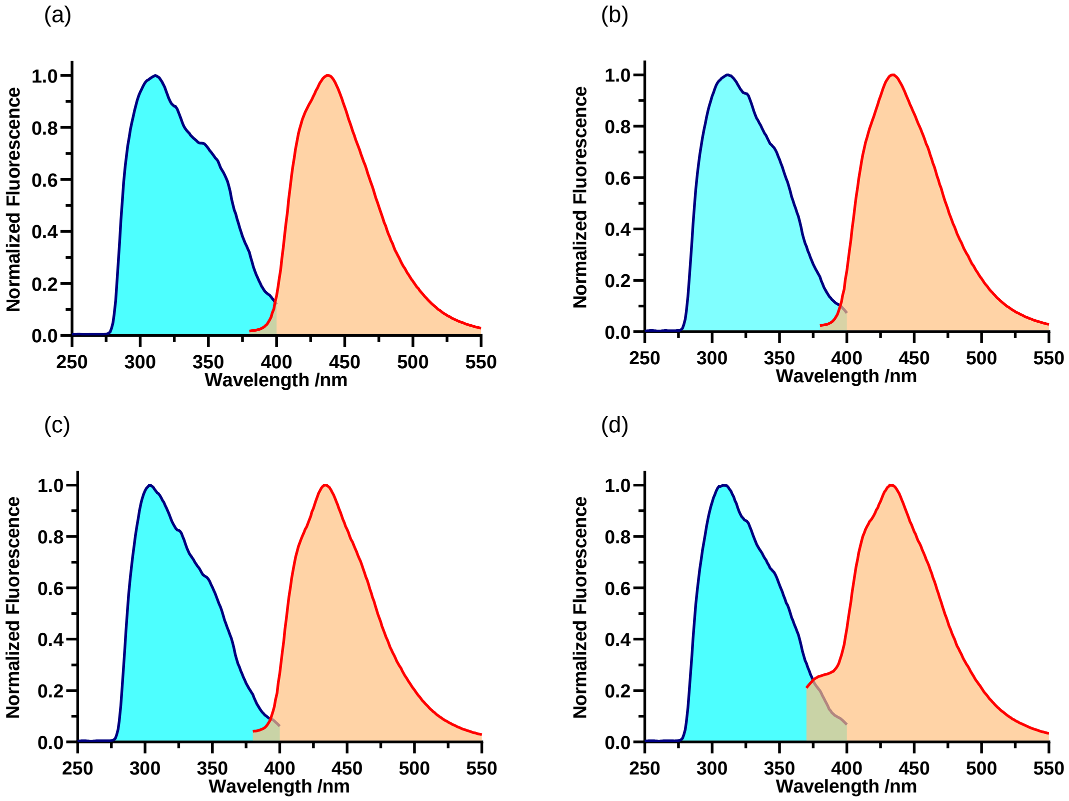

Figure S 148. Normalized excitation (blue) and emission (red) spectra in toluene of: (a) **13-SMe** (10^{-5} M) (λ_{exc} = 311 nm), (b) **13-S^{t}Bu** (10^{-5} M) (λ_{exc} = 311 nm), (c) **13-SH** (10^{-5} M) (λ_{exc} = 304 nm) and (d) **13-SS** (10^{-5} M) (λ_{exc} = 308 nm).

Fluorescence decay lifetimes and Quantum Yields

Table S 1. Quantum yields, fluorescence decay lifetimes in toluene at 10^{-5} M and χ^2 obtained from the exponential fitting of compounds **4**, **5-SH**, **5$_2$-SS**, **13-SMe**, **13-S^{t}Bu**, **13-SH**, **13-SS**.

Compound	Φ	τ /ns	χ^2
**4**	0.30	7.16	1.06
**5-SH**	0.21	8.87	1.02
**5$_2$-SS**	0.21	7.86	1.02
**13-SMe**	0.40	6.46	1.04
**13-S^{t}Bu**	0.32	8.26	1.02
**13-SH**	0.09	8.08	1.13
**13-SS**	0.05	7.51	1.15

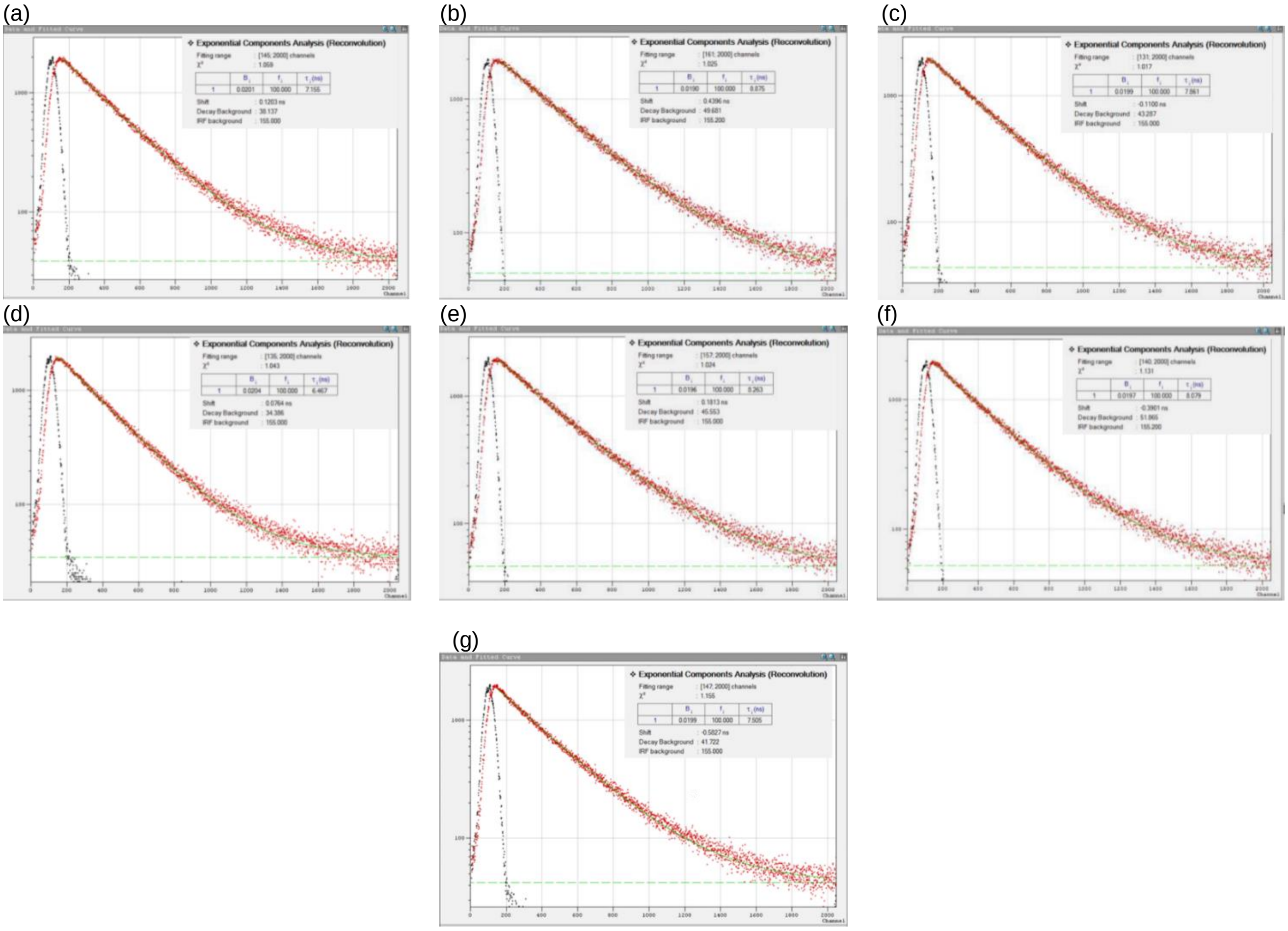

Figure S 149. Decay lifetimes of compounds (a) **4**, (b) **5-SH**, (c) **5$_2$-SS**, (d) **13-SMe**, (e) **13-S^{t}Bu**, (f) **13-SH** and (g) **13-SS**. Exponential fit corresponds to green line.

Cyclic Voltammograms

Recorded cyclic voltammograms show different reduction and oxidation processes. On one hand, we observe the redox processes of the sulfur-involved parts of the molecule (between -2.0 V and 1.0 V), whereas on the other hand, we observe the reduction potentials (below -2.0 V) of the aromatic parts of the molecule (i. e. corannulene).

Molecules containing thiol groups are involved in the following redox processes[14]:

$$\text{Ar-SH} + 1e^- \longrightarrow \text{Ar-S}^- + 1/2\text{H}_2 \qquad E^c$$

$$\text{Ar-S}^- \longrightarrow \text{Ar-S}^\bullet + 1e^- \qquad E^a$$

$$2\text{Ar-S}^\bullet \longrightarrow \text{Ar-SS-Ar}$$

Whereas molecules containing disulfide groups are involved in the following redox processes:

$$\text{Ar-SS-Ar} + 2e^- \longrightarrow 2\text{Ar-S}^- \qquad E^c$$

$$\text{Ar-S}^- \longrightarrow \text{Ar-S}^\bullet + 1e^- \qquad E^a$$

$$2\text{Ar-S}^\bullet \longrightarrow \text{Ar-SS-Ar}$$

The above equations are predominant in aprotic solvents. E^c refers to the reduction potential (cathodic scan) and E^a refers to the oxidation potential (anodic scan). In all cases, the redox processes are irreversible and diffusion controlled.[14] Further oxidations at higher positive voltages, possibly ascribed to the formation of sulfoxides, can be glimpsed but they are limited by the solvent electrochemical window. In the presence of protons, the mechanism is biased as any emerging thiolate is rapidly captured furnishing the corresponding thiol.

Table S 2. Reduction and oxidation potentials of selected compounds in deaerated DMF at a concentration of 1.0 mM containing a solution of NBu_4PF_6 (0.10 M). Scan rate of 100 mV · s^{-1}. Potentials are referenced against Fc/Fc^+.

	**E^c /V**	**E^a /V**	**Solvent**
**Ph-SH**[a]	-2.39	-0.61	AcCN
**5-SH**	-1.74	-0.47	DMF
**10-SH**	-1.59	-0.62	DMF
**13-SH**	-1.67	-0.57	DMF
**Ph-SS-Ph**[a]	-2.05	-0.64	AcCN
**5_2-SS**	-1.67	-0.58	DMF
**10-SS**	-1.69	-0.96	DMF
**13-SS**	-1.63	-0.45[b]	DMF

[a]Redox potentials from literature[14] in acetonitrile, NBu_4PF_6 (50 mM) and referenced against Fc/Fc^+. [b] Oxidation potential obtained from square-wave voltammetry (SWV).

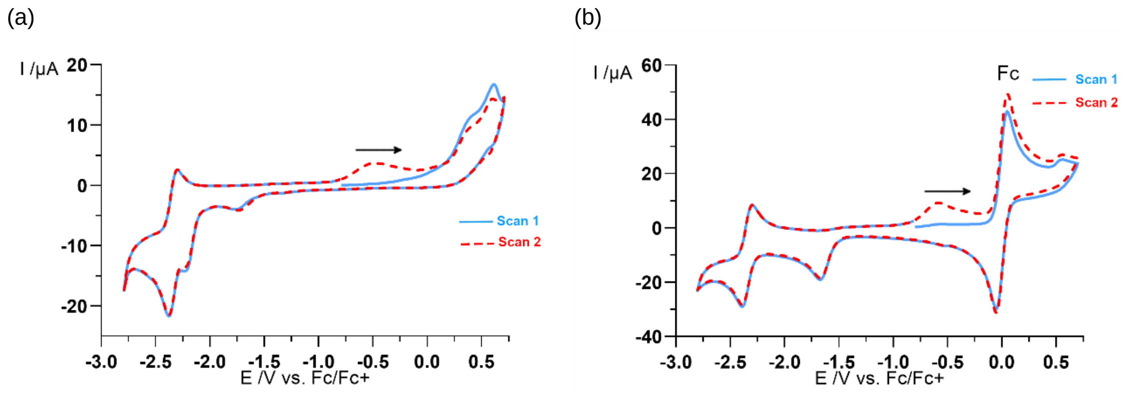

Figure S 150. Cyclic voltammograms of **5-SH** (a) and **5₂-SS** (b) in deaerated DMF at a concentration of 1.0 mM containing a solution of NBu_4PF_6 (0.10 M). Scan rate of 100 mV · s^{-1}. Potentials are referenced against Fc/Fc^+.

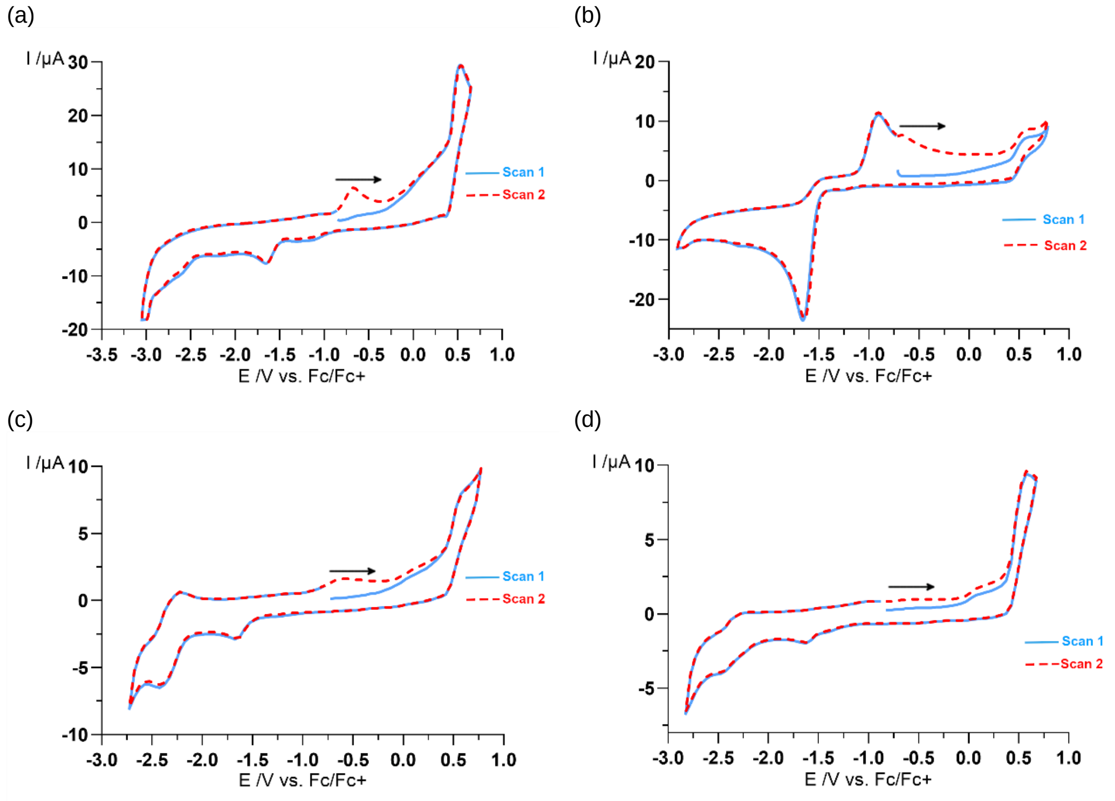

Figure S 151. Cyclic voltammograms of **10-SH** (a), **10-SS** (b), **13-SH** (c) and **13-SS** (d) in deaerated DMF at a concentration of 1.0 mM containing a solution of NBu_4PF_6 (0.10 M). Scan rate of 100 mV · s^{-1}. Potentials are referenced against Fc/Fc^+.

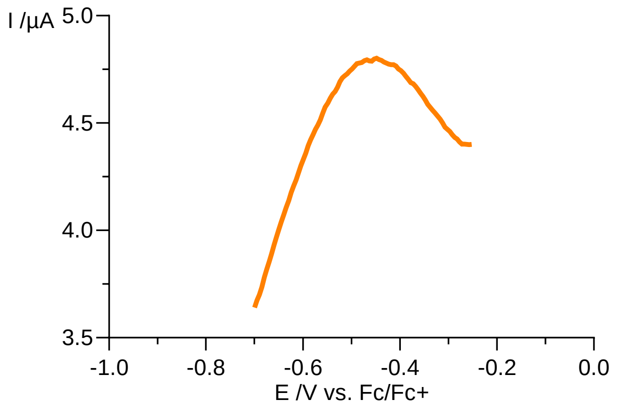

Figure S 152. Square-wave voltammetry (SWV) of the oxidation of **13-SS** (E^a).

Reduction peaks of corannulene motif are measured by square-wave voltammetry (SWV) and shown in the next Table S 3.

Table S 3. Reduction potentials of the aromatic groups of selected compounds in deareated DMF at a concentration of 1.0 mM containing a solution of NBu_4PF_6 (0.10 M). Scan rate of 100 mV · s^{-1}. Potentials are referenced against Fc/Fc^+. Values in parenthesis correspond to the difference with respect to the reference corannulene reductions (absolute value).

	**E^1 /V**	**E^2 /V**	**E^3 /V**	**E^4 /V**
**Corannulene**	-2.31	-2.86[a]	-	-
**4**	-2.24 (0.07)	-2.69 (0.17)	2.80	-3.32
**5-SH**	-2.19 (0.12)	-2.35 (0.51)	-2.89	-3.01
**5_2-SS**	-2.34 (0.03)	-2.93 (0.07)	-3.04	-
**13-SMe**	-2.23 (0.08)	-2.67 (0.19)	-	-
**13-S^{t}Bu**	-2.21 (0.10)	-2.63 (0.23)	-2.80	-3.04
**13-SH**	-2.31 (0.00)	-2.40 (0.46)	-2.82	-3.10
**13-SS**	--2.28 (0.03)	--2.37 (0.49)	-2.80	-

[a]Redox potential reported in the literature[3]

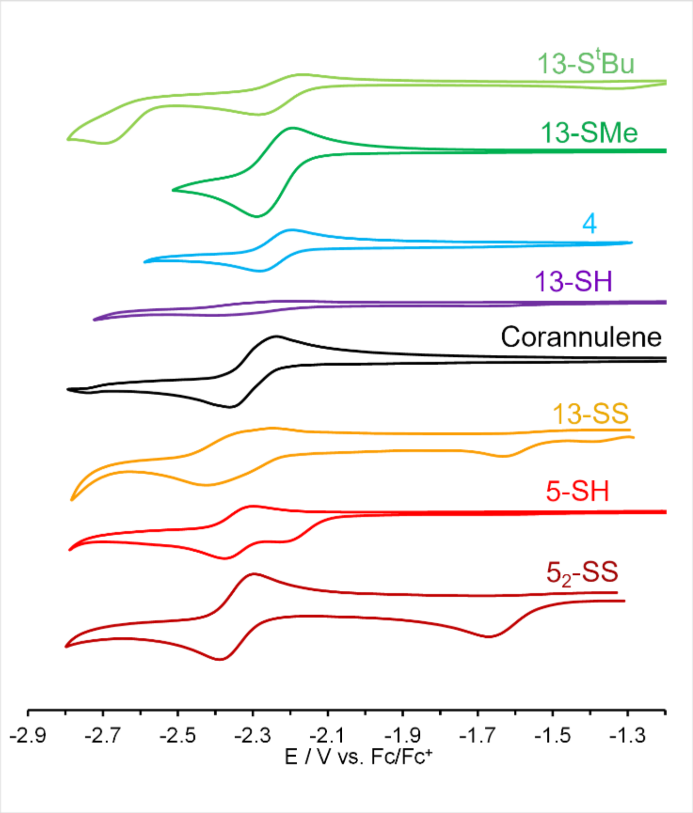

Figure S 153. Comparison of cyclic voltammograms of the first reduction peak of corannulene of a selection of reported compounds in deareated DMF at a concentration of 1.0 mM containing a solution of NBu_4PF_6 (0.10 M). Scan rate of 100 mV · s^{-1}. Potentials are referenced against Fc/Fc$^+$.

X-ray structures and Crystallographic Tables

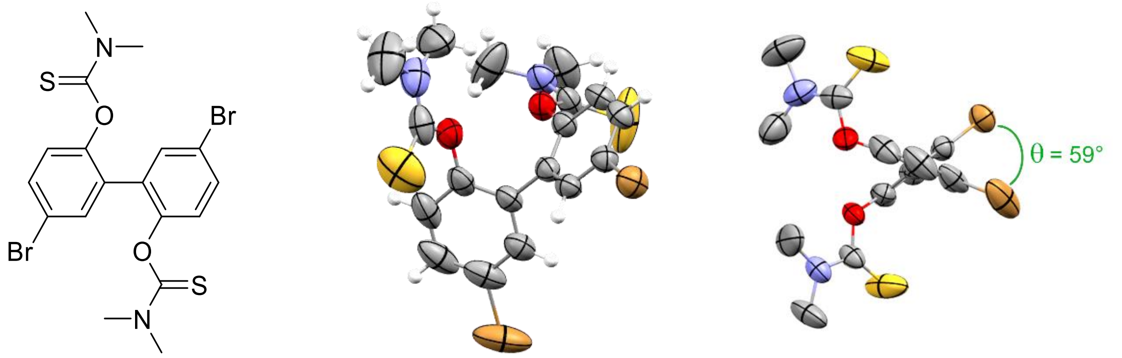

Figure S 154. Schematic representation and crystal structure of compound **8** (50% ellipsoid contour probability). Crystallization conditions: slow diffusion of hexane onto a saturated solution of the solute in CH_2Cl_2 at -20 ºC.

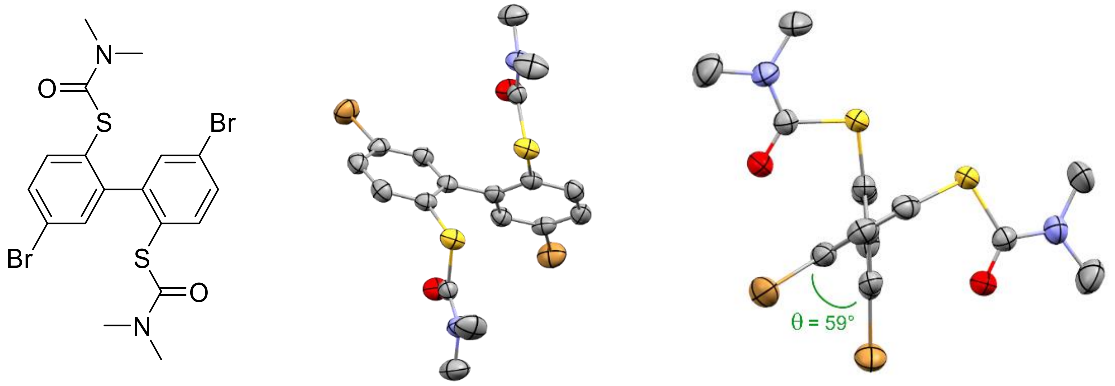

Figure S 155. Schematic representation and crystal structure of compound **9** (50% ellipsoid contour probability). Crystallization conditions: slow diffusion of hexane onto a saturated solution of the solute in CH_2Cl_2 at -20 ºC.

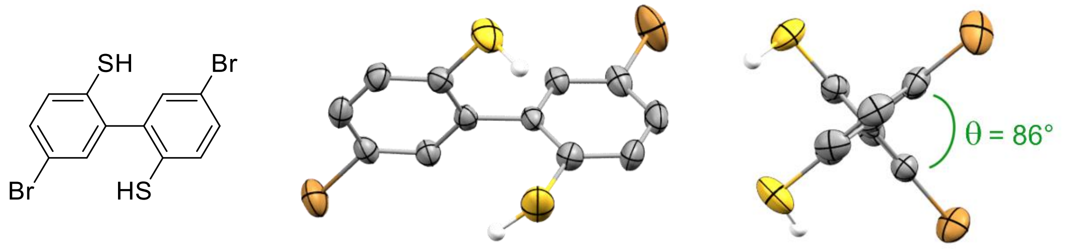

Figure S 156. Schematic representation and crystal structure of compound **10-SH** (50% ellipsoid contour probability). Crystallization conditions: slow evaporation of a solution of the compound in CH_2Cl_2 at room temperature.

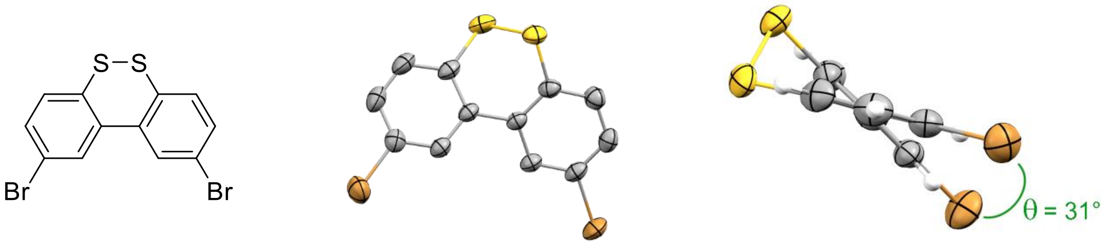

Figure S 157. Schematic representation and crystal structure of compound **10-SS** (50% ellipsoid contour probability). Crystallization conditions: slow diffusion of hexane onto a saturated solution of the solute in CH_2Cl_2 at -20 ºC.

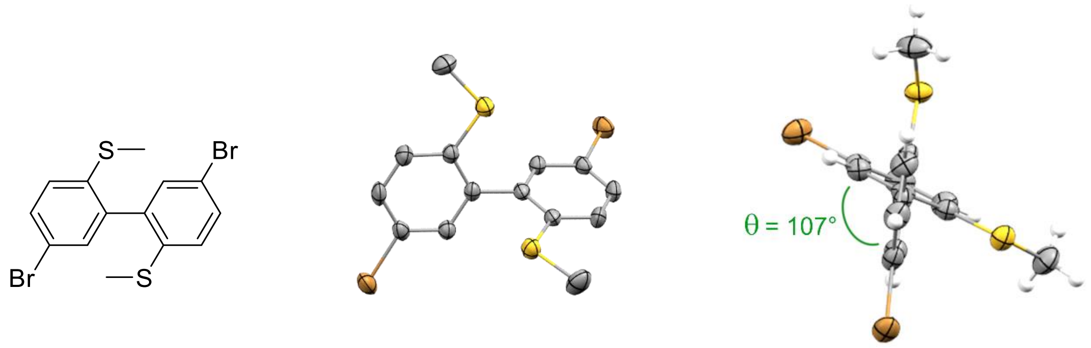

Figure S 158. Schematic representation and crystal structure of compound **11a** (50% ellipsoid contour probability). Crystallization conditions: slow diffusion of hexane onto a saturated solution of the solute in CH_2Cl_2 at -20 ºC.

Table S 4. Crystallographic data of compounds **8**, **9**, **10-SH**, **10-SS** and **11a**.

Identification code	**8**	**9**	**10-SH**	**10-SS**	**11a**
CCDC Number	2164882	2164883	2164886	2164885	2164884
Empirical formula	$C_{18}H_{18}Br_2N_2O_2S_2$	$C_{18}H_{18}Br_2N_2O_2S_2$	$C_{12}H_8Br_2S_2$	$C_{12}H_6S_2Br_2$	$C_{14}H_{12}Br_2S_2$
Formula weight	414.63	345.52	376.12	374.11	404.18
Temperature [K]	293(2)	293(2)	293(2)	293(2)	293(2)
Crystal system	monoclinic	monoclinic	monoclinic	monoclinic	triclinic
Space group	$P2_1/n$	I2/a	$P2_1/c$	C2/c	P-1
a [Å]	9.6592(5)	15.9840(12)	11.7473(5)	17.8218(6)	8.6212(6)
b [Å]	16.7813(11)	6.5237(4)	8.7219(5)	7.3394(2)	8.6939(6)
c [Å]	12.9763(8)	19.7055(13)	12.8782(8)	20.0050(7)	10.4780(8)
α [°]	90	90	90	90	90.896(6)
β [°]	93.664(6)	93.753(6)	90.299(5)	110.725(4)	103.761(6)
γ [°]	90	90	90	90	104.033(6)
V [$Å^3$]	2099.1(2)	2050.4(2)	1319.47(12)	2447.35(15)	737.79(10)
Z	4	4	4	8	2
ρcalc[g/cm^3]	1.640	1.679	1.893	2.031	1.819
μ [mm^{-1}]	4.075	4.172	6.429	6.932	5.756
F(000)	1032.0	1032.0	728.0	1440.0	396.0
Crystal size [mm^3]	0.46 × 0.25 × 0.151	0.54 × 0.31 × 0.09	0.43 × 0.28 × 0.18	0.414 × 0.141 × 0.128	0.57 × 0.42 × 0.34
Radiation	Mo Kα	Mo Kα	Mo Kα	Mo Kα	Mo Kα
2θ range for data collection [°]	6.744 to 59.61	6.748 to 59.134	6.614 to 59.666	6.73 to 59.576	6.746 to 59.044
Reflections collected	10566	5940	7219	26333	5630
Independent reflections	5001 [R_{int} = 0.0306, R_{sigma} = 0.0561]	2508 [R_{int} = 0.0257, R_{sigma} = 0.0331]	3166 [R_{int} = 0.0308, R_{sigma} = 0.0407]	3248 [R_{int} = 0.0305, R_{sigma} = 0.0166]	3460 [R_{int} = 0.0256, R_{sigma} = 0.0470]
Data/restraints/parameters	5001/0/239	2508/0/120	3166/0/147	3248/0/145	3460/0/165
GOF on F^2	1.024	1.033	1.049	1.036	1.015
Final R indexes [I>=2σ (I)]	R_1 = 0.0532, wR_2 = 0.1258	R_1 = 0.0392, wR_2 = 0.0831	R_1 = 0.0431, wR_2 = 0.0986	R_1 = 0.0307, wR_2 = 0.0651	R_1 = 0.0348, wR_2 = 0.0696
Final R indexes [all data]	R_1 = 0.1206, wR_2 = 0.1575	R_1 = 0.0590, wR_2 = 0.0937	R_1 = 0.0728, wR_2 = 0.1159	R_1 = 0.0460, wR_2 = 0.0714	R_1 = 0.0529, wR_2 = 0.0783
Max/min Δρ [eÅ-3]	0.58/-0.73	0.66/-0.59	0.78/-0.69	0.55/-0.72	0.36/-0.67
Flack parameter	-	-	-	-	-

In situ switching procedure

In the absence of fullerenes

A solution of compound **5_2-SS** or **13-SS** (0.50 mL, 1.0 mM) in $CDCl_3$ was placed in an NMR tube. A ^{1}H-NMR spectrum was recorded. Immediately after, a solution of $NaBH_4$ (0.10 M) in MeOD (20 μL, 4.0 eq) was added all at once before gently shaking the tube. Another ^{1}H-NMR spectrum was subsequently recorded. Quantitative conversion to **5-SH** or **13-SH** was observed. Thereafter, a solution of freshly sublimated I_2 (0.10 M) in $CHCl_3$ (10 μL, 2.0 eq) and NEt_3 (5.0 μL, 68 μmol) were subsequently added to the solution. The mixture was gently shaken again. A ^{1}H-NMR spectrum was acquired, showing the recovery of oxidized species **5_2-SS** or **13-SS** and completing the cycle. The full process could be repeated several times without evidence of decomposition or spectral distortions due to byproducts build-up.

In the presence of fullerenes

A solution of compound **5_2-SS** (0.50 mL, 1.0 mM) in the presence of C_{60} or C_{70} (10 eq) in toluene-d_8 was placed in an NMR tube. A ^{1}H-NMR spectrum was recorded. Immediately after, a solution of $LiAlH_4$ (0.10 M) in toluene-d_8/THF (9:1) (50 μL, 1.0 eq) and trifluoroacetic acid (5.0 μL, 65 μmol) were added all at once before gently shaking the tube. Another ^{1}H-NMR spectrum was subsequently recorded. Quantitative conversion to **5-SH** was observed. Thereafter, a solution of freshly sublimated I_2 (0.10 M) in toluene-d_8 (5.0 μL, 1 eq) and NEt_3 (5.0 μL, 68 μmol) were subsequently added to the solution. The mixture was gently shaken again. A ^{1}H-NMR spectrum was acquired, showing the recovery of oxidized species **5_2-SS** and completing the cycle. The full process could be repeated several times multiplying the equivalents of reducing and oxidizing reagents (including acid and basic media) by a factor of 1.5 after each cycle.[15]

A solution of compound **13-SS** (0.50 mL, 1.0 mM) in the presence of C_{60} or C_{70} (10 eq) in a mixture of toluene-d_8 and MeOD (4:1) was placed in an NMR tube. A ^{1}H-NMR spectrum was recorded. Immediately after, a solution of $NaBH_4$ (50 mM) in toluene-d_8/MeOD (4:1) (80 μL, 8.0 eq) was added all at once before gently shaking the tube. Another ^{1}H-NMR spectrum was subsequently recorded. Quantitative conversion to **13-SH** was observed. Thereafter, a solution of freshly sublimated I_2 (0.10 M) in toluene-d_8/MeOD (4:1) (20 μL, 4 eq) was added to the solution in one portion and the mixture was gently shaken again. A ^{1}H-NMR spectrum was acquired, showing the recovery of oxidized species **13-SS** and completing the cycle. The full process could be repeated several times multiplying the equivalents of reducing and oxidizing reagents by a factor of 1.5 after each cycle.[15]

Ex situ switching procedure

For species **5_2-SS** and **13-SS**, their reduction protocol already described in Synthetic procedures and characterization details section (Figure S 29-36 and Figure S 127-135) was applied. For species **5-SH** and **13-SH** their oxidation protocol is as follows: the compound (50 μmol) was dissolved under inert atmosphere in a mixture of THF/MeOH (5mL, 3:2) furnishing a light yellow color solution. $NaBH_4$ (30 mg, 0.80 mmol) was added. The solution color turned to brownish. After 30 min, the reaction was quenched with HCl (35%) (1.0 mL) and the solution turned yellow again. It was extracted with CH_2Cl_2/H_2O (3 x 10 mL), dried with $MgSO_4$, filtered, concentrated under vacuum and precipitated with hexane. A light-yellow powder appeared which was isolated by centrifugation with quantitative yield.

Additionally, all these procedures can be reversed by starting with an initial solution of compounds **5-SH** or **13-SH**, and altering the order of chemical effectors (i.e. I_2 followed by $NaBH_4$ or $LiAlH_4$).

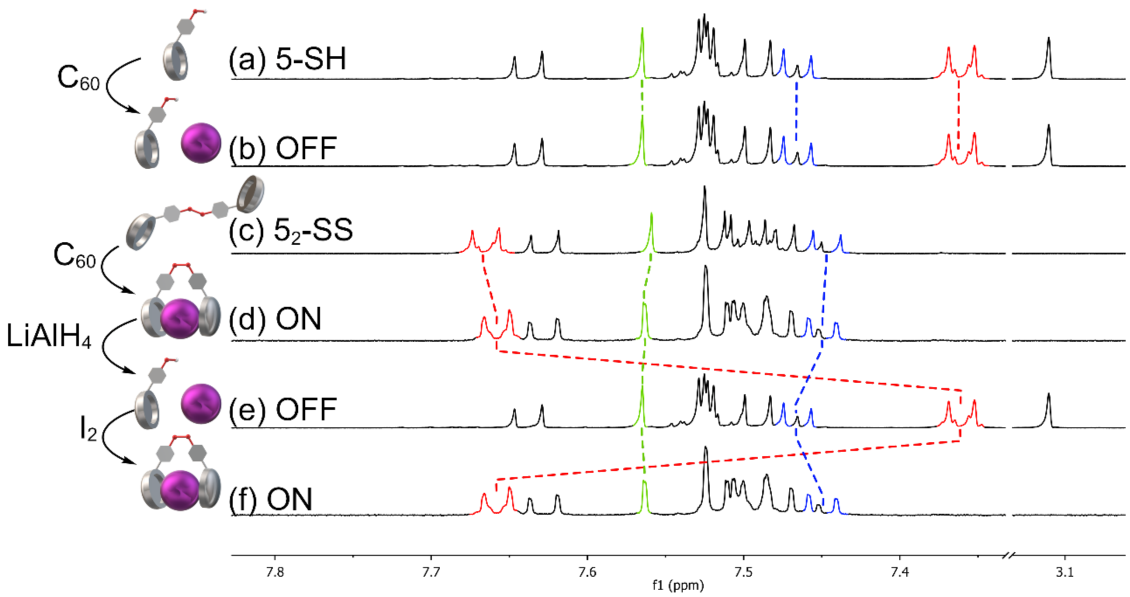

Figure S 159. Stacked ^{1}H-NMR (500 MHz, toluene-d$_8$) spectra of **5-SH**+C_{60}/**5$_2$-SS**+C_{60} red-ox experiments at 298 K. (a) **5-SH**, (b) **5-SH** + 10 eq of C_{60}, (c) **5$_2$-SS**, (d) **5$_2$-SS** + 10 eq of C_{60}, (e) **5-SH** + 10 eq of C_{60} after reducing host **5$_2$-SS**, (f) **5$_2$-SS** + 10 eq of C_{60} after oxidizing host **5-SH**. Red signals correspond to phenylene protons whereas green and blue signals belong to corannulene moieties.

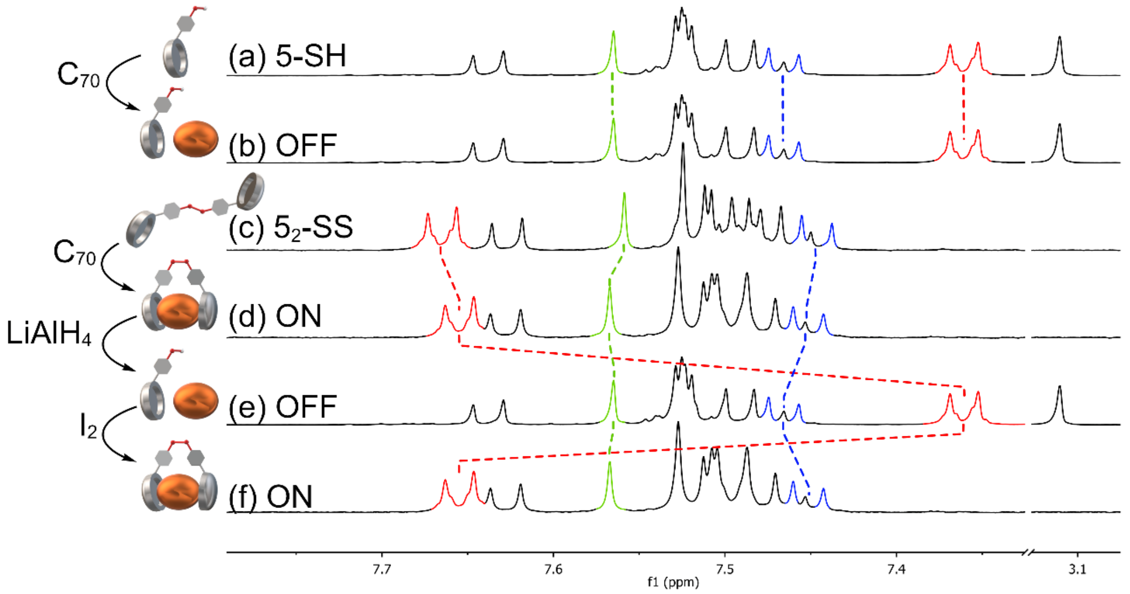

Figure S 160. Stacked ^{1}H-NMR (500 MHz, toluene-d$_8$) spectra of **5-SH**+C_{70}/**5$_2$-SS**+C_{70} red-ox experiments at 298 K.(a) **5-SH**, (b) **5-SH** + 10 eq of **C_{70}**, (c) **5$_2$-SS**, (d) **5$_2$-SS** + 10 eq of C_{70}, (e) **5-SH** + 10 eq of C_{70} after reducing host **5$_2$-SS**, (f) **5$_2$-SS** + 10 eq of C_{70} after oxidizing host **5-SH**. Red signals correspond to phenylene protons whereas green and blue signals belong to corannulene moieties.

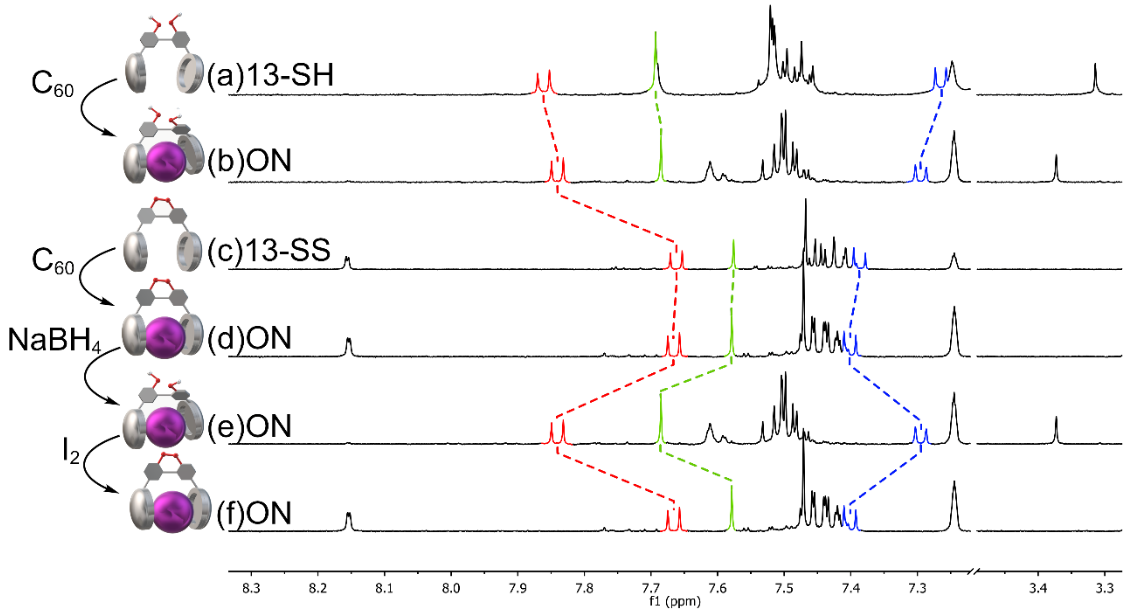

Figure S 161. Stacked ^{1}H-NMR (500 MHz, toluene-d_8) spectra of **13-SH**+C_{60}/**13-SS**+C_{60} red-ox experiments at 298 K.(a) **13-SH**, (b) **13-SH** + 10 eq of C_{60}, (c) **13-SS**, (d) **13-SS** + 10 eq of C_{60}, (e) **13-SH** + 10 eq of C_{60} after reducing host **13-SS**, (f) **13-SS** + 10 eq of C_{60} after oxidizing host **13-SH**. Blue signal correspond to phenylene protons whereas green and red signals belong to corannulene moieties.

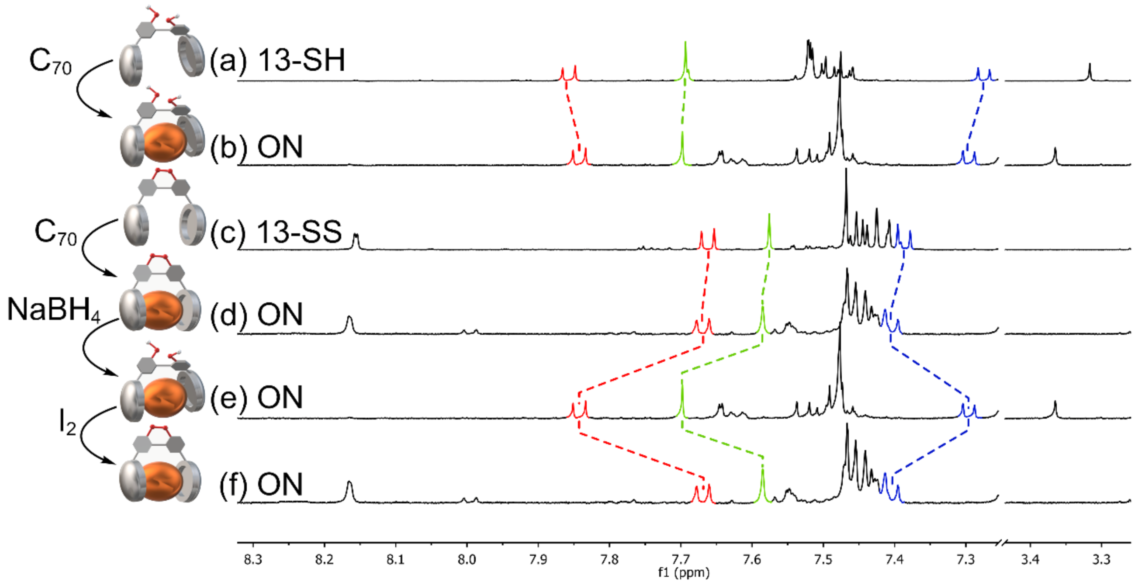

Figure S 162. Stacked ^{1}H-NMR (500 MHz, toluene-d_8) spectra of **13-SH**+C_{70}/**13-SS**+C_{70} red-ox experiments at 298 K.(a) **13-SH**, (b) **13-SH** + 10 eq of C_{70}, (c) **13-SS**, (d) **13-SS** + 10 eq of C_{70}, (e) **13-SH** + 10 eq of C_{70} after reducing host **13-SS**, (f) **13-SS** + 10 eq of C_{70} after oxidizing host **13-SH**. Blue signal correspond to phenylene protons whereas green and red signals belong to corannulene moieties.

Dilution experiments

In order to get an idea of the possible stacking properties of **4**, in solution, which might affect the binding capabilities of fullerenes upon aggregation, dilution experiments were carried out by adding pure toluene-d_8 to concentrated solutions of the compounds under study covering different concentrations in the range between 10^{-3} and 10^{-5} M. Stacked spectra of compound **4** (Figure S 163) shows no chemical shift change at all, therefore no stacking is observed within the concentrations utilized for association constants determination (see below). The same behavior was observed for compound **5-SH**.

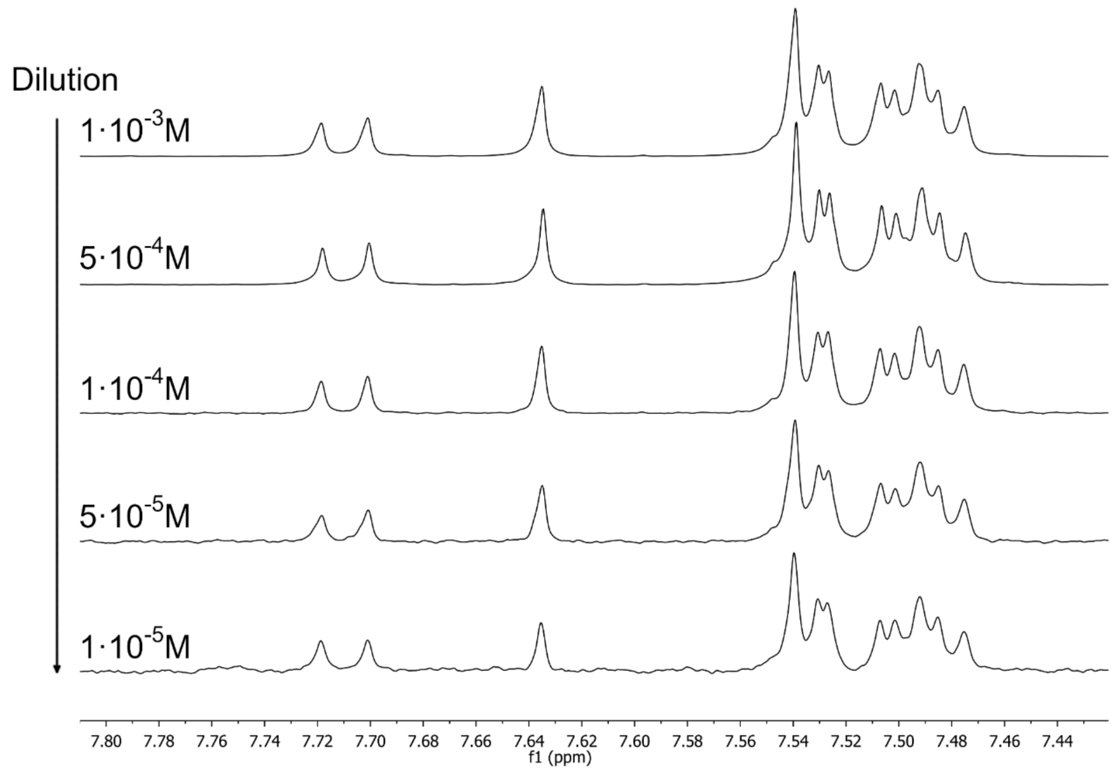

Figure S 163. Stacked ^1H-NMR (500 MHz, toluene-d_8) spectra of compound **4** in a range of concentration from 10^{-3} M to 10^{-5} M at 298 K.

Association constants measurements

In order to estimate the association constants (K_a) of compounds **5₂-SS**, **13-SMe**, **13-S^{t}Bu**, **13-SH** and **13-SS** with fullerenes C_{60} and C_{70}, the following procedure was carried out: a solution of each compound (1.0×10^{-4} M) in deuterated toluene was prepared and a known volume was transferred to an NMR tube capped with a septum (0.50 mL). The titration was carried out by injecting through the septum known portions of a stock solution of C_{60} (1.0×10^{-3} M) in deuterated toluene to cover a wide range of equivalents. The same protocol was followed for C_{70}. A ^{1}H-NMR spectrum was recorded at room temperature after each addition. Once all data had been obtained, the changes in chemical shifts (Δδ) of selected protons were plotted as a function of the molar fraction of the guest, and the resulting curve was fitted by a nonlinear method using the global analysis approach according to the following equations assuming a 1:1 equilibrium:

$$K_a = \frac{[HG]}{[H][G]} \qquad \textbf{eq. 1}$$

Changes in chemical shifts upon NMR titration are expressed:

$$\Delta\delta = \Delta\delta_{max}\left(\frac{[HG]}{H_0}\right) \qquad \textbf{eq. 2}$$

Where:

[HG] is the concentration of the guest of the complex, and is calculated using the following equation:

$$[HG] = \frac{1}{2}\left([G_0] + [H_0] + \frac{1}{K_a}\right) - \sqrt{\left([G_0] + [H_0] + \frac{1}{K_a}\right)^2 + 4[G_0][H_0]} \qquad \textbf{eq. 3}$$

Where:

$[G_0]$ is the total concentration of the guest (C_{60} or C_{70}).

$[H_0]$ is the total concentration of the host (**5₂-SS**, **13-SMe**, **13-S^{t}Bu**, **13-SH** or **13-SS**).

$\Delta\delta_{max}$ is Δδ at maximum complexation (100% supramolecular complex formation).

K_a is the estimated association constant for 1:1 equilibrium.

In each case, $\Delta\delta_{max}$ and K_a for a 1:1 equilibrium were extracted by using the non-linear fitting tool provided by the open access web portal Supramolecular.org (http://supramolecular.org/) applying equations 2 and 3. Links to all the fittings of the data are provided below.

5₂-SS Vs C_{60}

http://app.supramolecular.org/bindfit/view/c31c926f-e1fc-48ec-b3ef-4c59420f003f

5₂-SS Vs C_{70}

http://app.supramolecular.org/bindfit/view/1e29f8de-4a77-4a73-b0ed-4cae3b378010

13-SMe Vs C_{60}

http://app.supramolecular.org/bindfit/view/6ce33393-da33-494c-9353-05a5740b4843

13-SMe Vs C_{70}

http://app.supramolecular.org/bindfit/view/905907e1-3aaa-42c4-8ea6-3f4c2234fb58

13-S^{t}Bu Vs C_{60}

http://app.supramolecular.org/bindfit/view/5036e472-487a-4751-bfe6-1b29895f5aaa

13-S^{t}Bu Vs C_{70}

http://app.supramolecular.org/bindfit/view/f9b3c4b6-f28b-467c-b754-97adb6cc3330

13-SH Vs C_{60}

http://app.supramolecular.org/bindfit/view/ab2ff5cd-095f-4be0-bb5b-253b487b476e

13-SH Vs C_{70}

http://app.supramolecular.org/bindfit/view/af5df872-abc6-4d44-801c-7d933398cdc9

13-SS Vs C_{60}

http://app.supramolecular.org/bindfit/view/c4d2ae7a-cff2-40c2-8317-1da13ea140de

13-SS Vs C_{70}

http://app.supramolecular.org/bindfit/view/a7900167-1623-4760-91d8-a1af490ba5e5

Table S 5. Estimated K_a values calculated from selected protons in each compound (in M^{-1}).

	C_{60}	C_{70}
**5_2-SS**	$1.87 \pm 0.03 \times 10^2$	$7.24 \pm 0.17 \times 10^2$
**13-SMe**	$1.97 \pm 0.01 \times 10^3$	$2.34 \pm 0.02 \times 10^3$
**13-S^{t}Bu**	$5.64 \pm 0.04 \times 10^2$	$1.08 \pm 0.01 \times 10^3$
**13-SH**	$1.90 \pm 0.02 \times 10^3$	$2.03 \pm 0.01 \times 10^3$
**13-SS**	$3.93 \pm 0.04 \times 10^2$	$4.65 \pm 0.07 \times 10^2$

5_2-SS Vs C_{60}

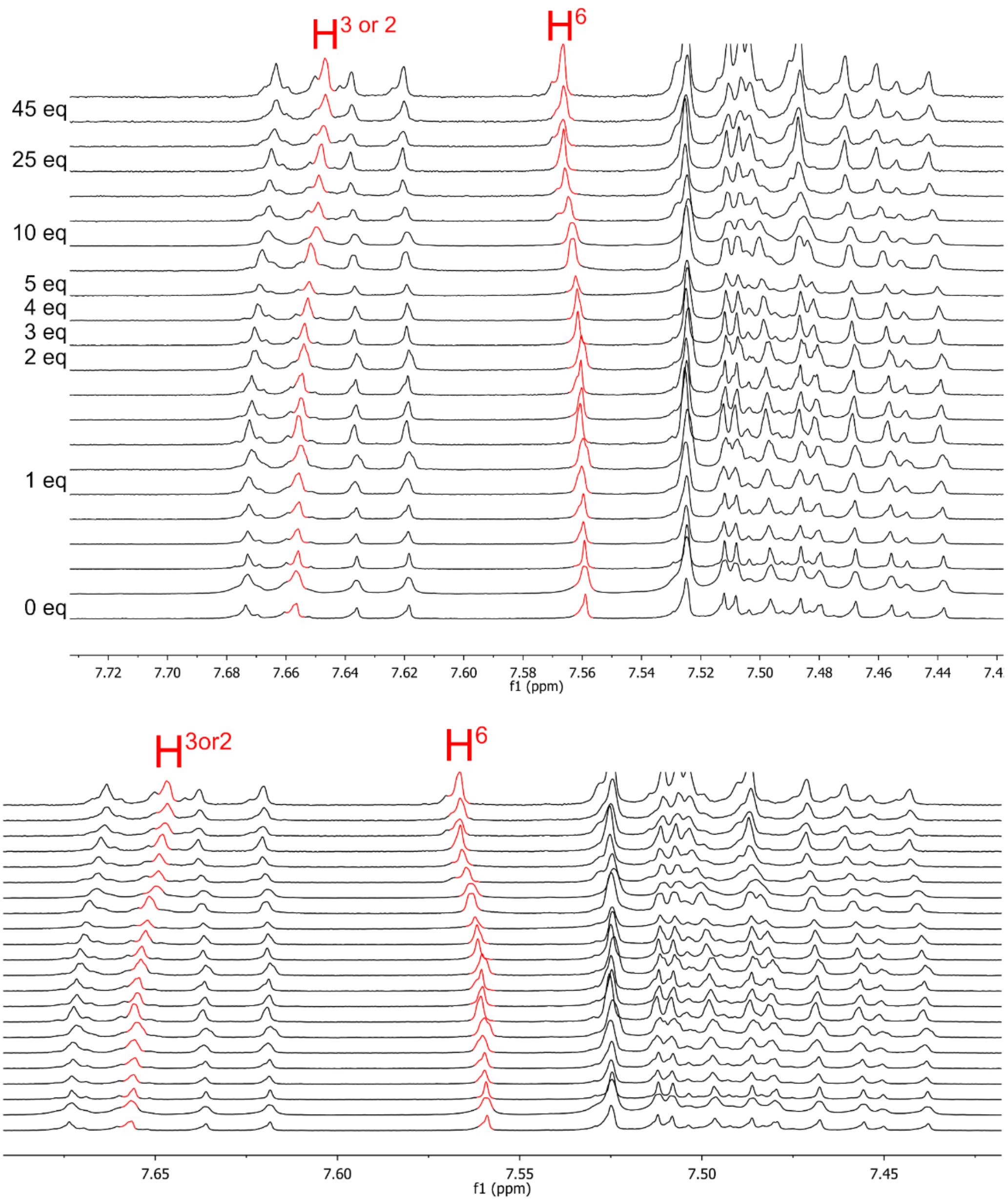

Figure S 164. Stacked ^{1}H-NMR (500 MHz, toluene-d_8) spectra for the titration of **5_2-SS** with variable concentrations of C_{60} at 298 K.

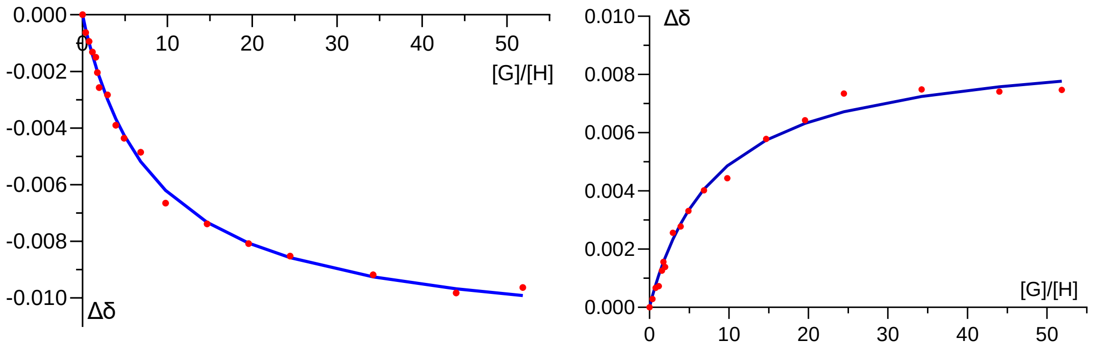

Figure S 165. Nonlinear regressions for selected protons (left plot: H_{3or2}, right plot: H_6) for the titration of **5_2-SS** with C_{60}.

5_2-SS Vs C_{70}

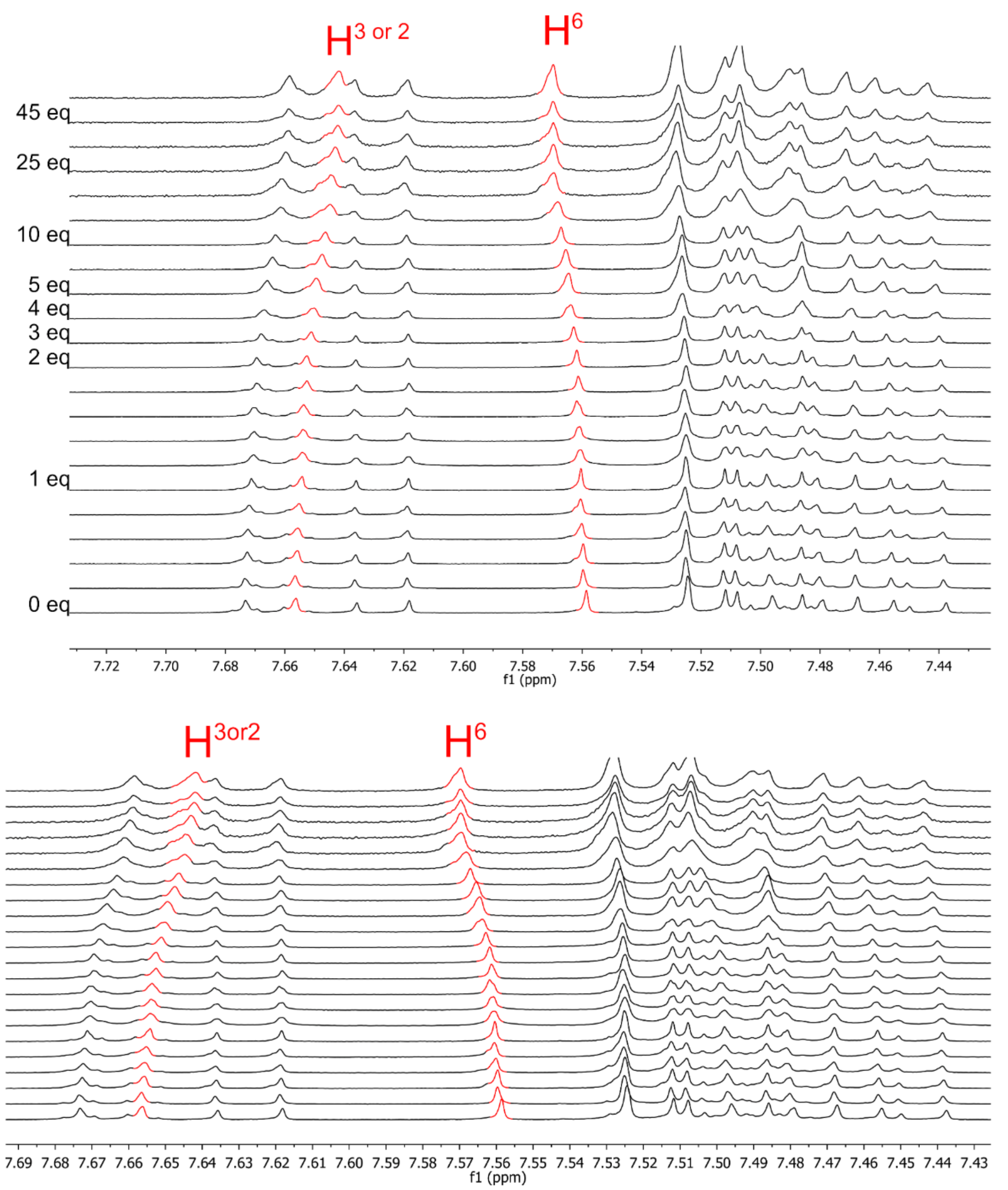

Figure S 166. Stacked ^{1}H-NMR (500 MHz, toluene-d_8) spectra for the titration of **5_2-SS** with variable concentrations of C_{70} at 298 K.

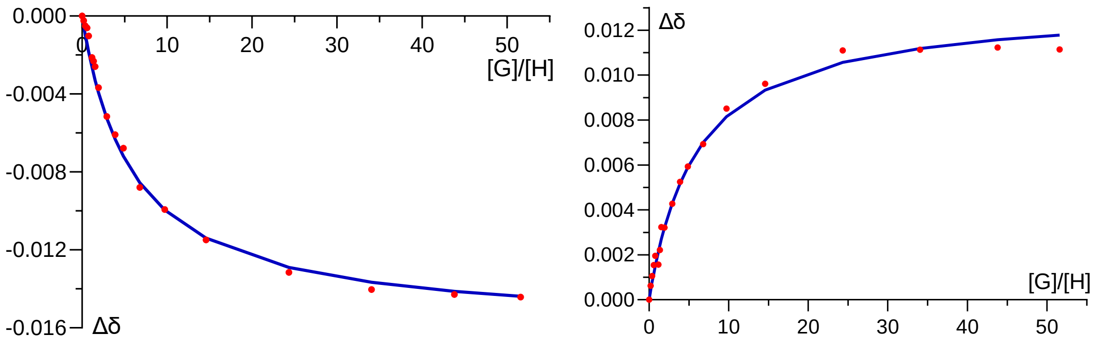

Figure S 167. Nonlinear regressions for selected protons (left plot: H_{3or2}, right plot: H_6) for the titration of **5_2-SS** with C_{70}.

13-SMe Vs C_{60}

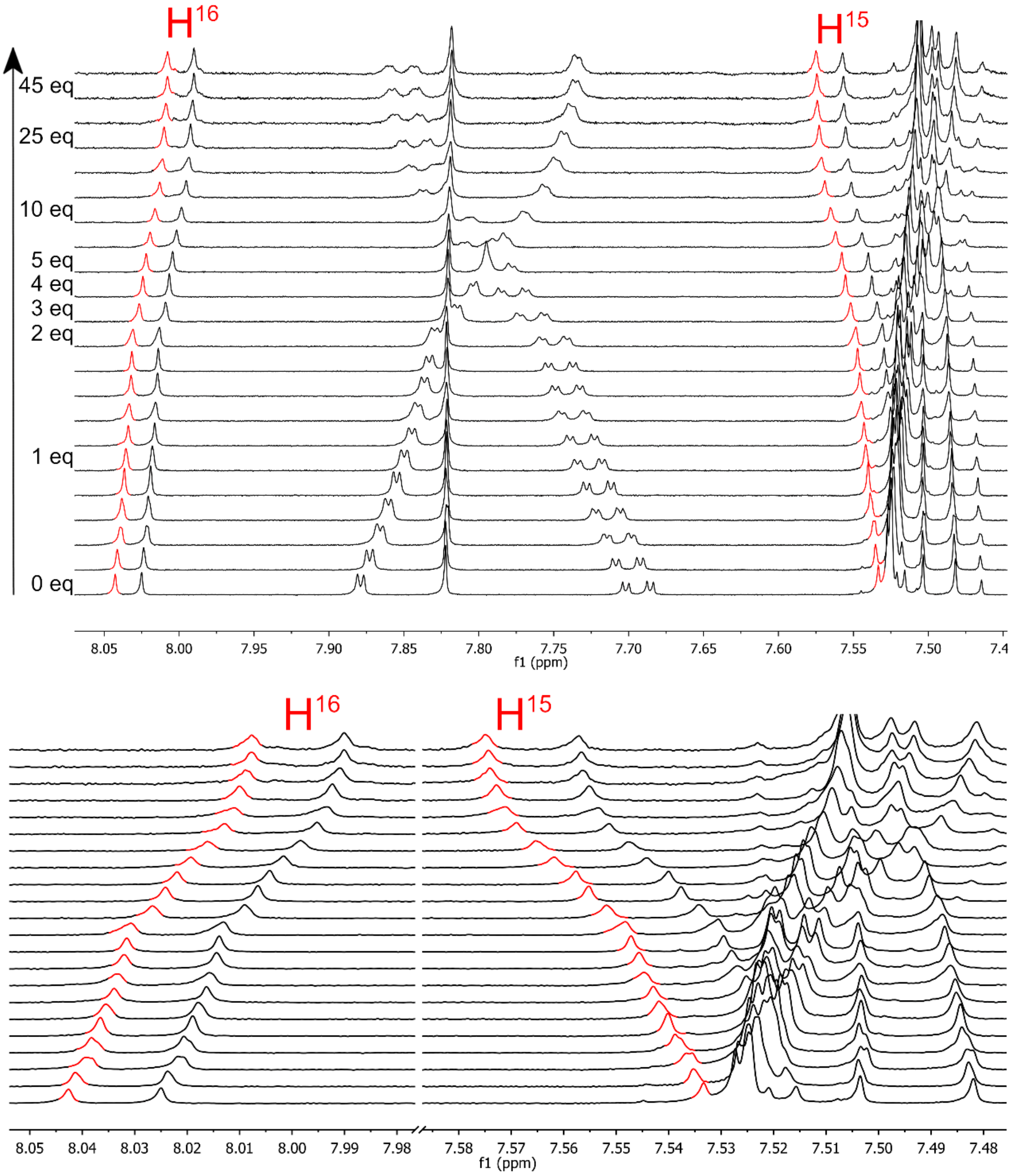

Figure S 168. Stacked ^{1}H-NMR (500 MHz, toluene-d_8) spectra for the titration of **13-SMe** with variable concentrations of C_{60} at 298 K.

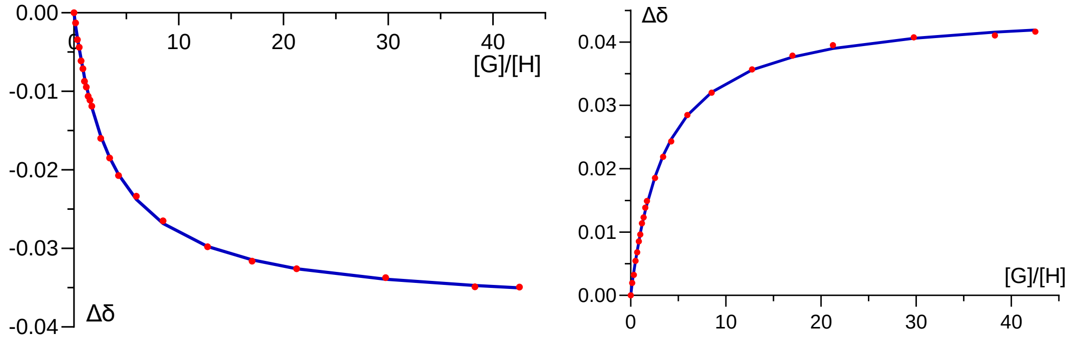

Figure S 169. Nonlinear regressions for selected protons (left plot: H_{16}, right plot: H_{15}) for the titration of **13-SMe** with C_{60}.

13-SMe Vs C_{70}

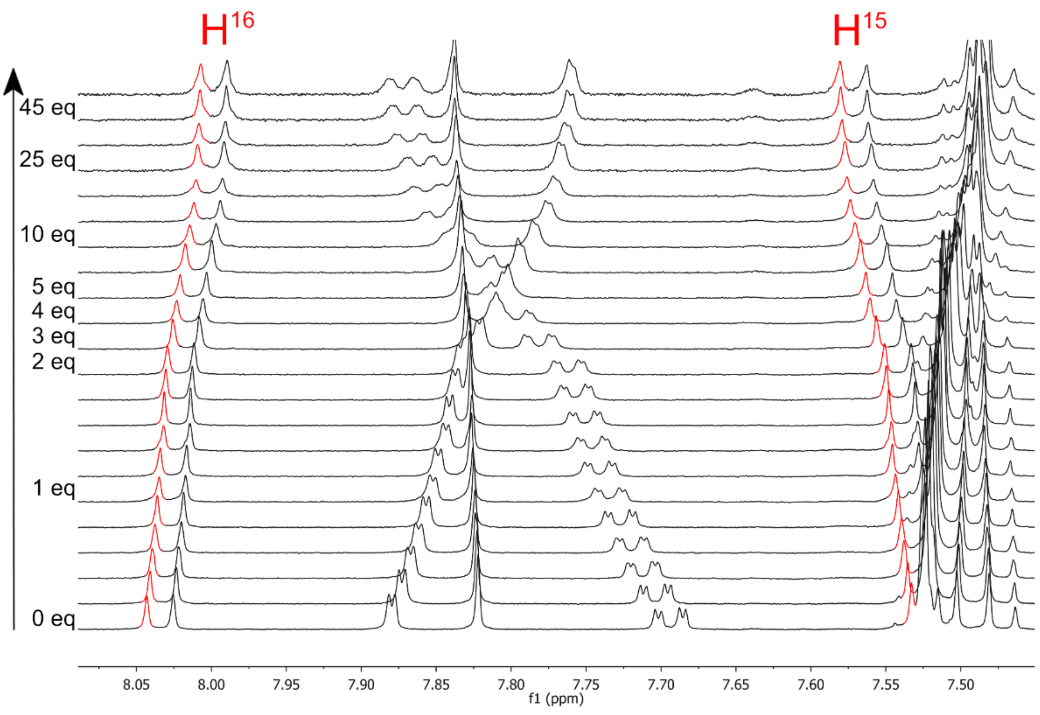

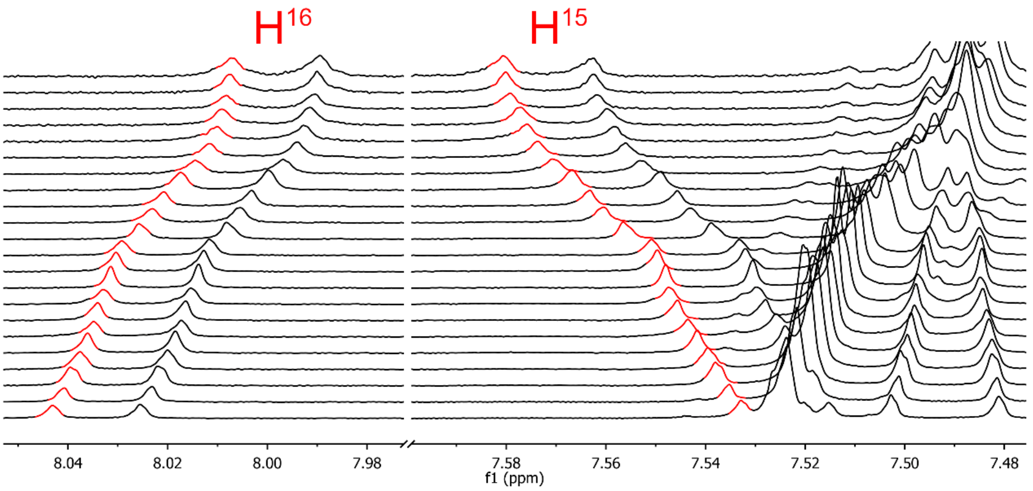

Figure S 170. Stacked ^{1}H-NMR (500 MHz, toluene-d$_8$) spectra for the titration of **13-SMe** with variable concentrations of C_{70} at 298 K.

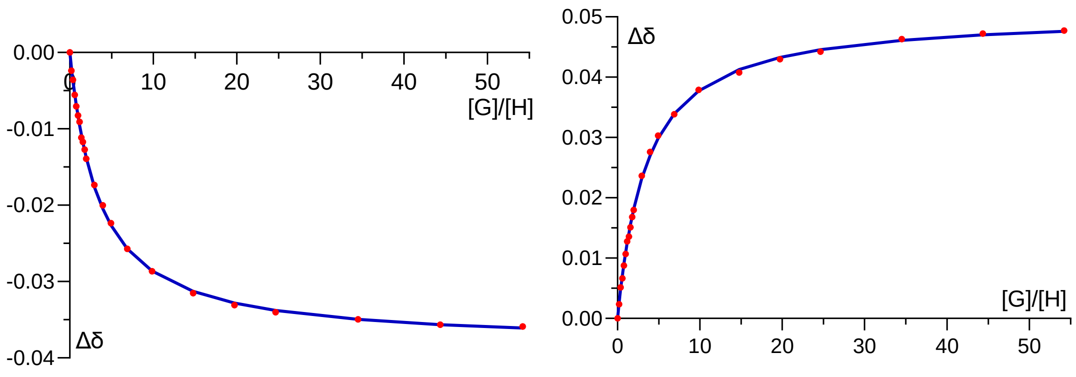

Figure S 171. Nonlinear regressions for selected protons (left plot: H_{16}, right plot: H_{15}) for the titration of **13-SMe** with C_{70}.

13-S^{t}Bu Vs C_{60}

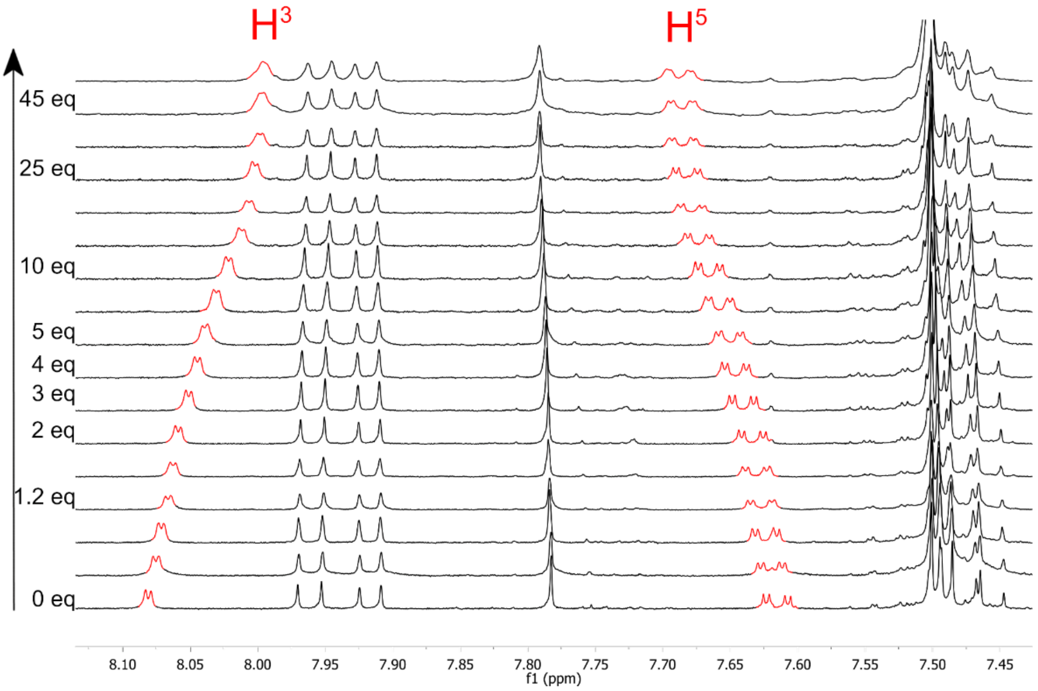

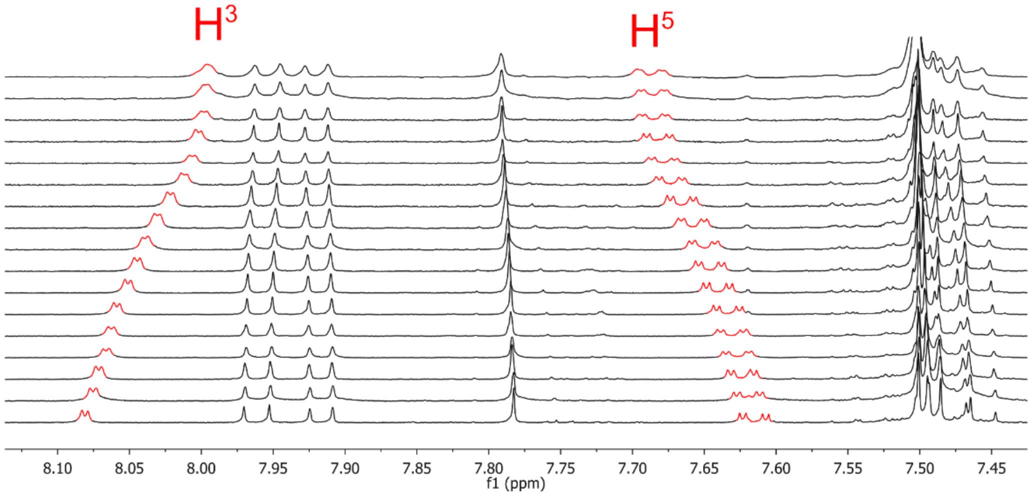

Figure S 172. Stacked ^{1}H-NMR (500 MHz, toluene-d_8) spectra for the titration of **13-S^{t}Bu** with variable concentrations of C_{60} at 298 K.

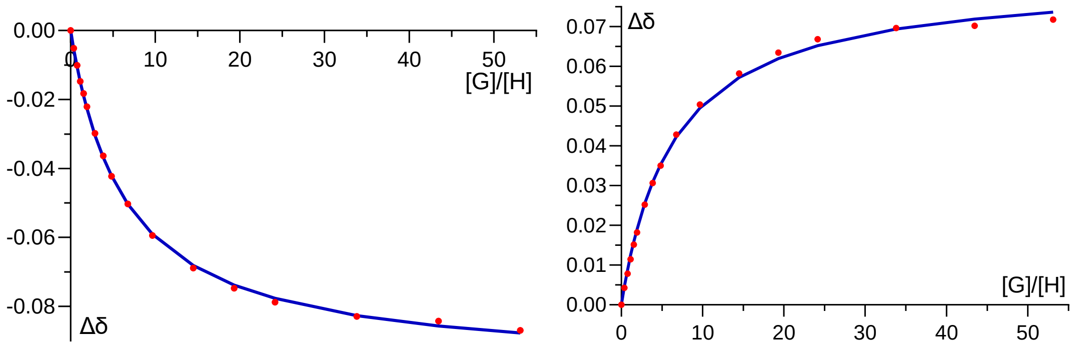

Figure S 173. Nonlinear regressions for selected protons (left plot: H_3, right plot: H_5) for the titration of **13-S^{t}Bu** with C_{60}.

13-S^{t}Bu Vs C_{70}

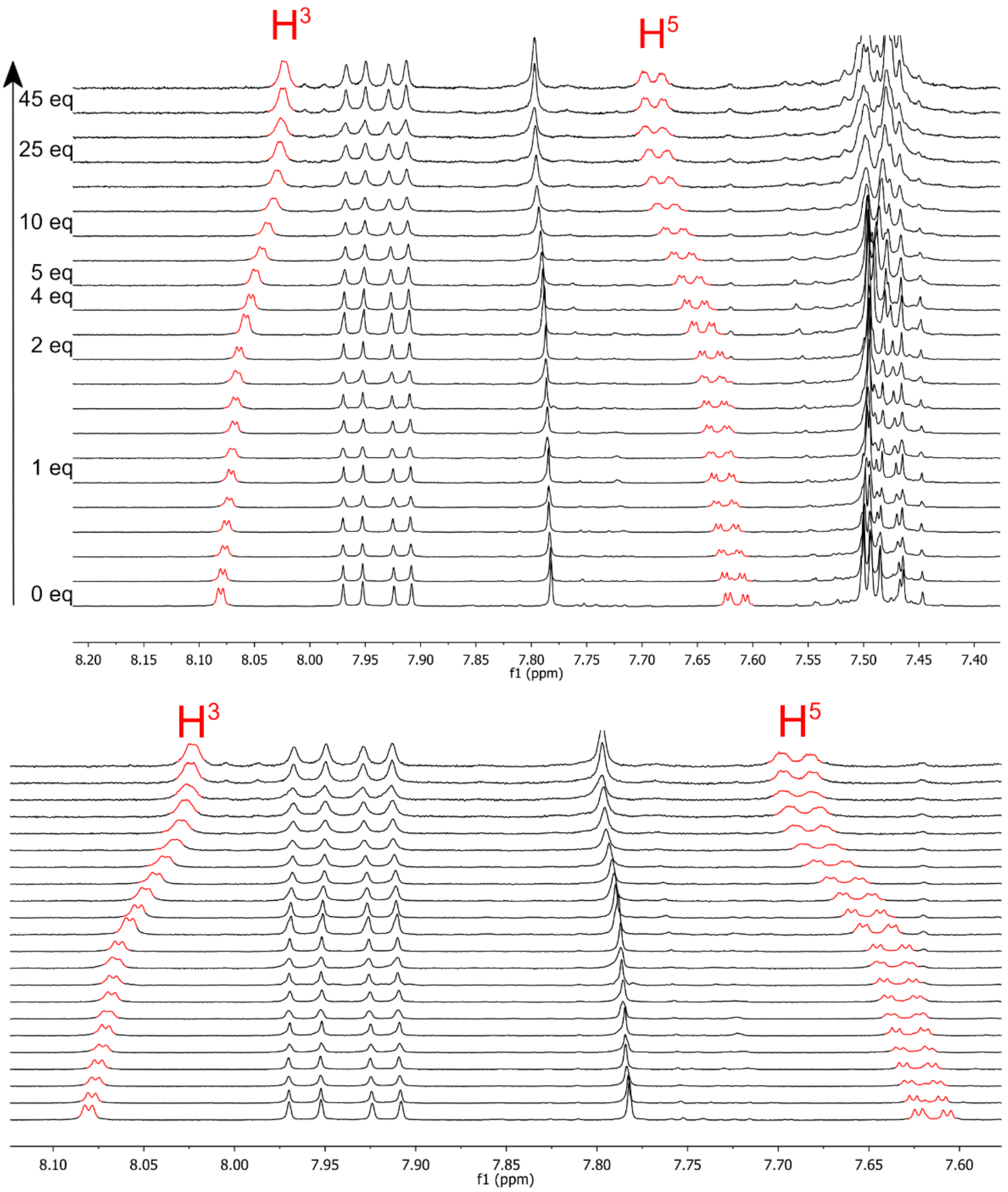

Figure S 174. Stacked ^{1}H-NMR (500 MHz, toluene-d$_8$) spectra for the titration of **13-S^{t}Bu** with variable concentrations of C_{70} at 298 K.

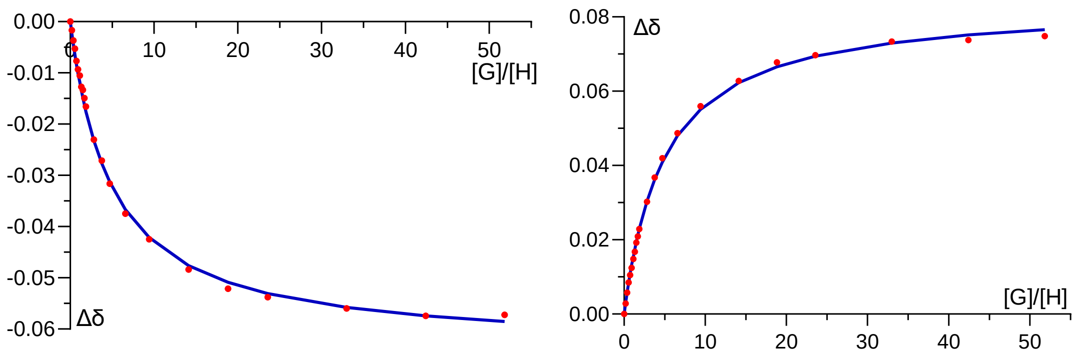

Figure S 175. Nonlinear regressions for selected protons (left plot: H_3, right plot: H_5) for the titration of **13-S^{t}Bu** with C_{70}.

13-SH Vs C_{60}

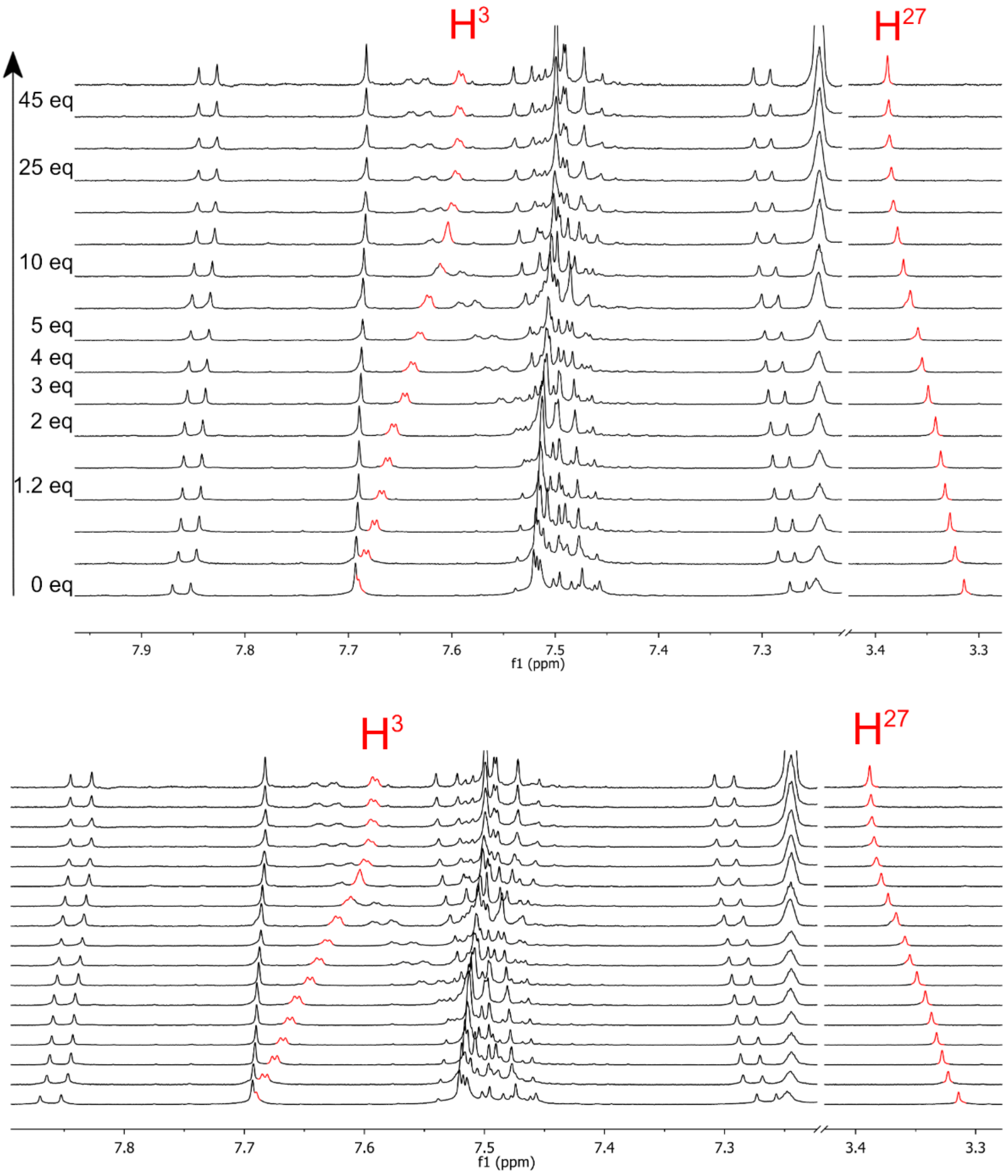

Figure S 176. Stacked ^{1}H-NMR (500 MHz, toluene-d_8) spectra for the titration of **13-SH** with variable concentrations of C_{60} at 298 K.

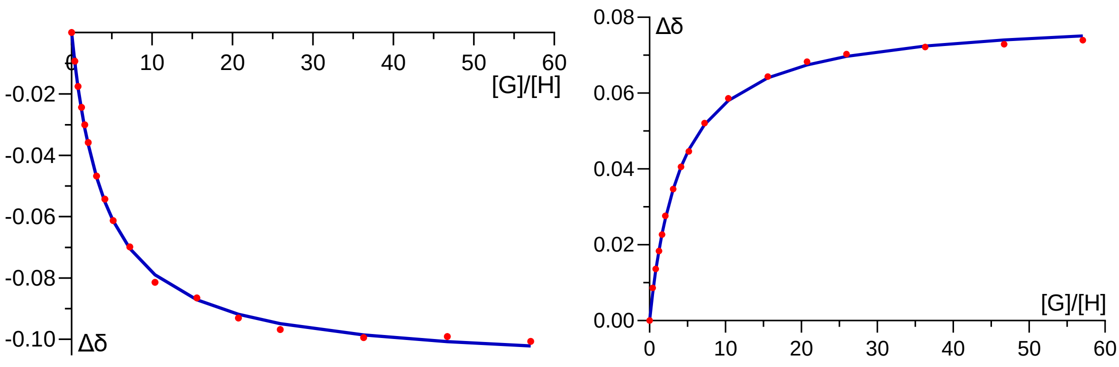

Figure S 177. Nonlinear regressions for selected protons (left plot: H_3, right plot: H_5) for the titration of **13-SH** with C_{60}.

13-SH Vs C_{70}

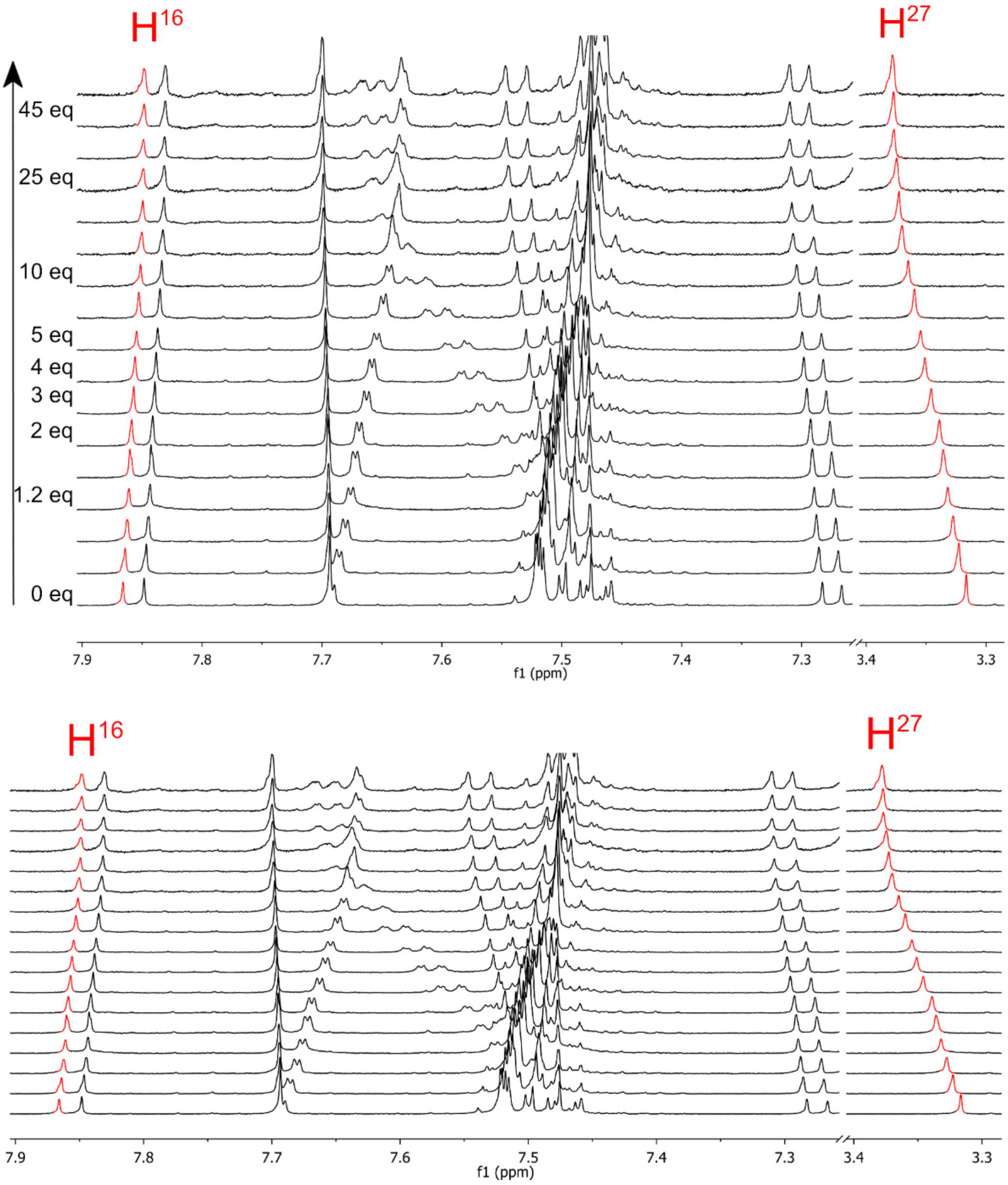

Figure S 178. Stacked ^{1}H-NMR (500 MHz, toluene-d_8) spectra for the titration of **13-SH** with variable concentrations of C_{70} at 298 K.

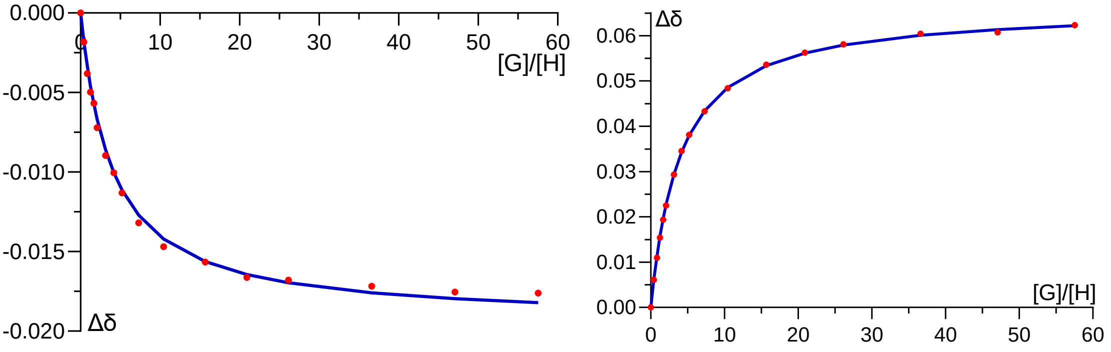

Figure S 179. Nonlinear regressions for selected protons (left plot: H_{16}, right plot: H_{27}) for the titration of **13-SH** with C_{70}.

13-SS Vs C_{60}

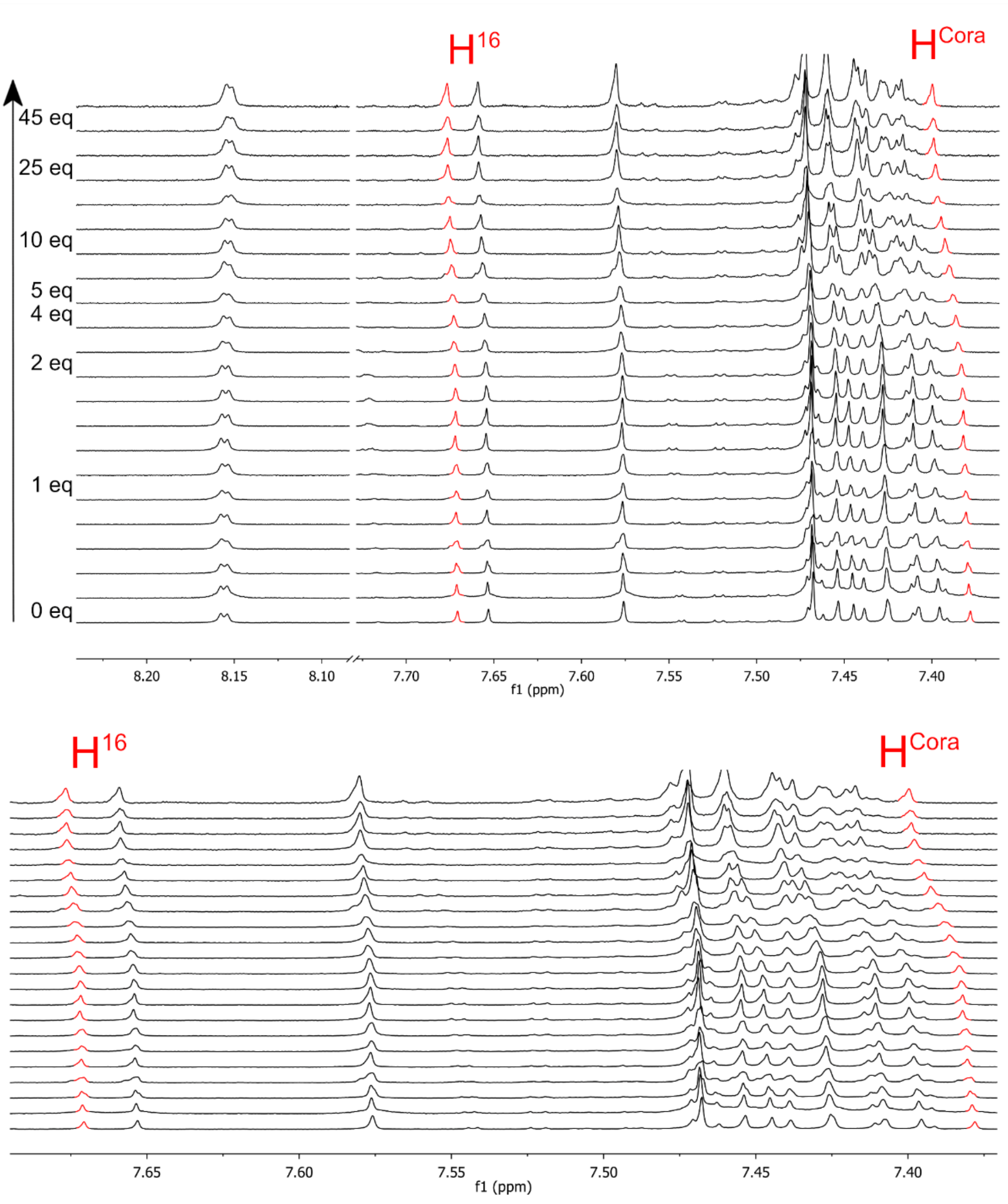

Figure S 180. Stacked ^{1}H-NMR (500 MHz, toluene-d_8) spectra for the titration of **13-SS** with variable concentrations of C_{60} at 298 K.

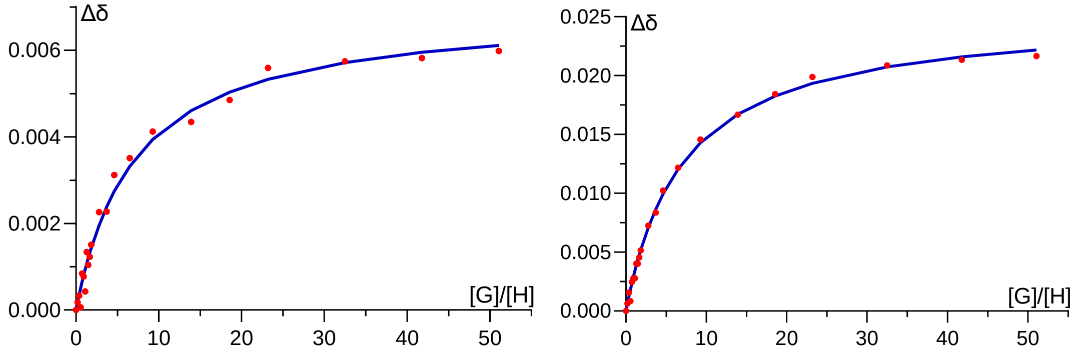

Figure S 181. Nonlinear regressions for selected protons (left plot: H_{16}, right plot: H_{Cora}) for the titration of **13-SS** with C_{60}.

13-SS Vs C_{70}

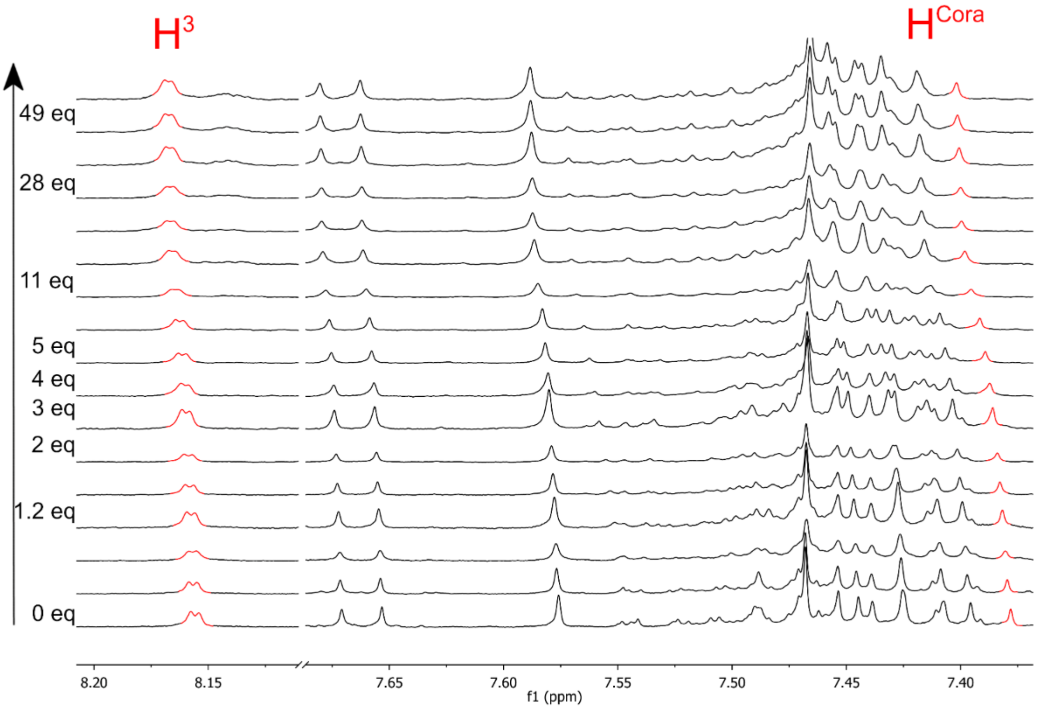

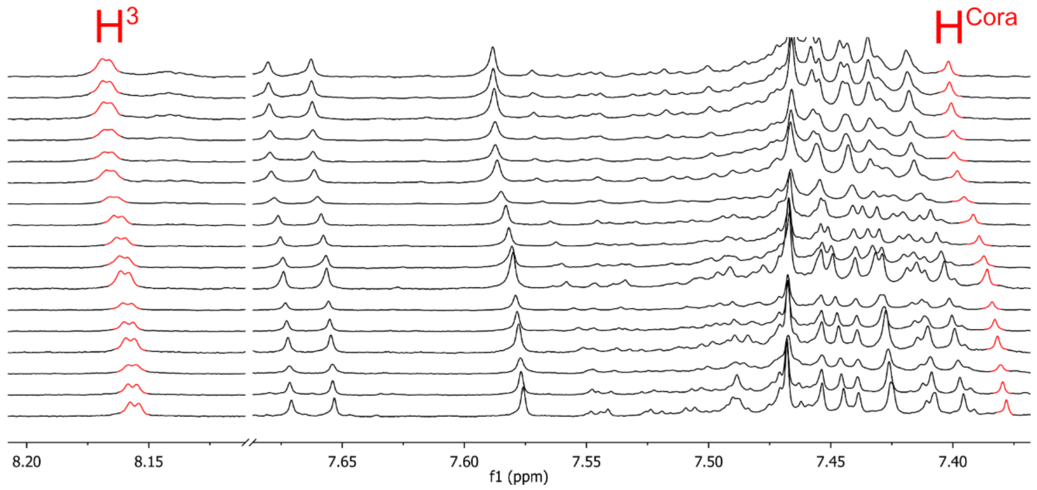

Figure S 182. Stacked ^{1}H-NMR (500 MHz, toluene-d_8) spectra for the titration of **13-SS** with variable concentrations of C_{70} at 298 K.

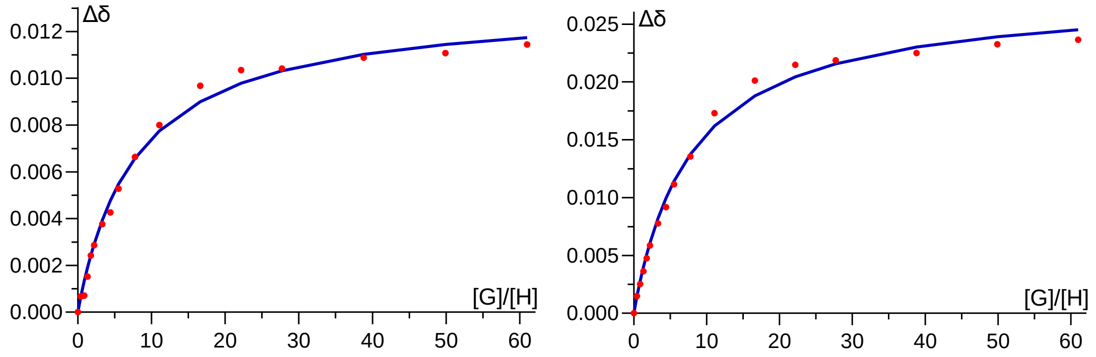

Figure S 183. Nonlinear regressions for selected protons (left plot: H_3, right plot: H_{Cora}) for the titration of **13-SS** with C_{70}.

Kinetic Studies

Solutions of **13-SH** and **C_{70}@13-SH** were prepared in toluene-d_8 at a concentration of 2.0 $\times 10^{-5}$ M and transferred to NMR tubes. A ^{1}H-NMR spectrum was immediately recorded. Both tubes were then uncapped to expose the solutions to environmental oxygen and monitored at regular intervals.

The oxidative rate constants (*k*) were detemined by linear fitting following first-order kinetics according to the following reaction (eq. 4):

$$\text{13-SH} \rightarrow \text{13-SS} \qquad \textbf{eq. 4}$$

Whose differential equation, according to the law of mass action is (eq. 5):

$$-\frac{d[\text{13-SH}]}{dt} = k[\text{13-SH}] \qquad \textbf{eq. 5}$$

Above equation provide a linearized integrated expression (eq. 6):

$$ln\frac{[\text{13-SH}]_t}{[\text{13-SH}]_0} = -kt \qquad \textbf{eq. 6}$$

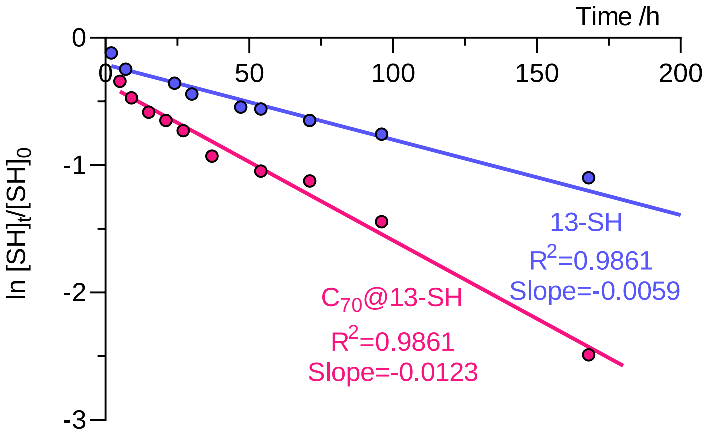

Figure S 184. Linear fitting plot of oxidation rate constant of compound **13-SH** in the presence (pink) or absence (blue) of C_{70}.

Table S 6. Obtained oxidative rate constants and half-life times of compound **13-SH** in the absence and presence of C_{70}.

	**13-SH**	**C_{70}@13-SH**
***k* / h^{-1}**	5,9 $\times$ 10^{-3}	1.2 $\times$ 10^{-2}
**$t_{1/2}$ / h**	117.4	56.3

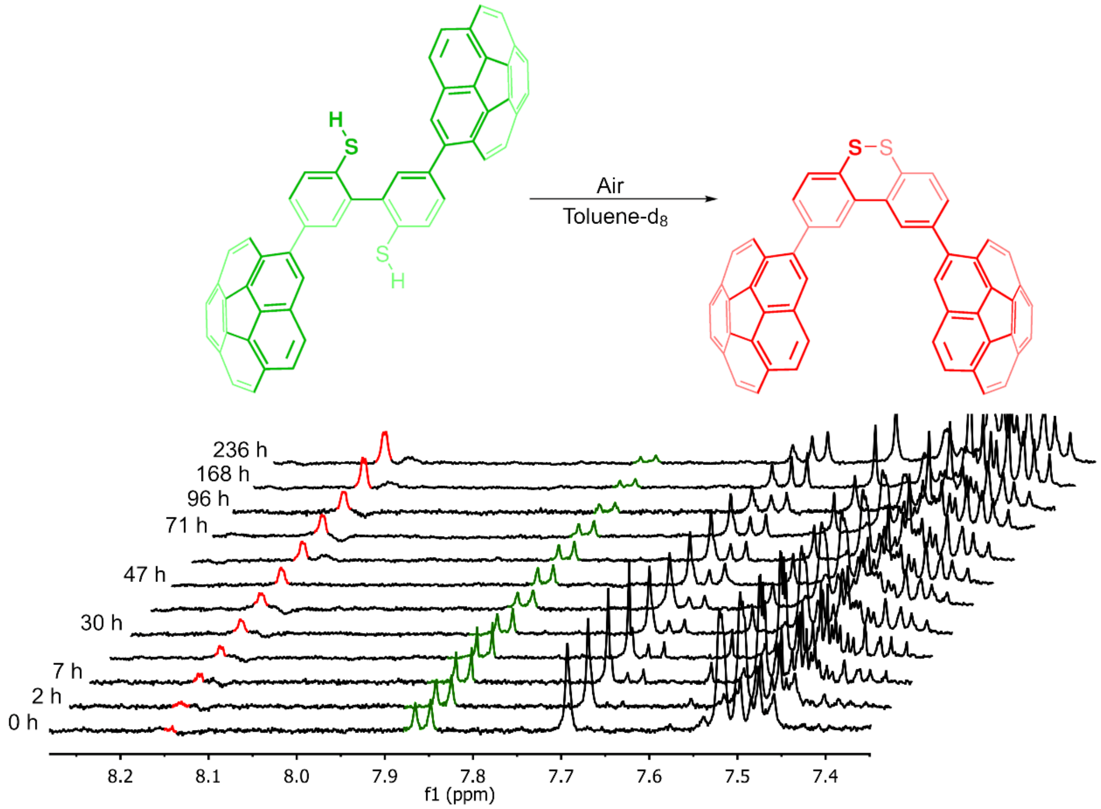

Figure S 185. Stacked ^{1}H NMR spectra of compound **13-SH** in toluene-d_8 monitored over time.

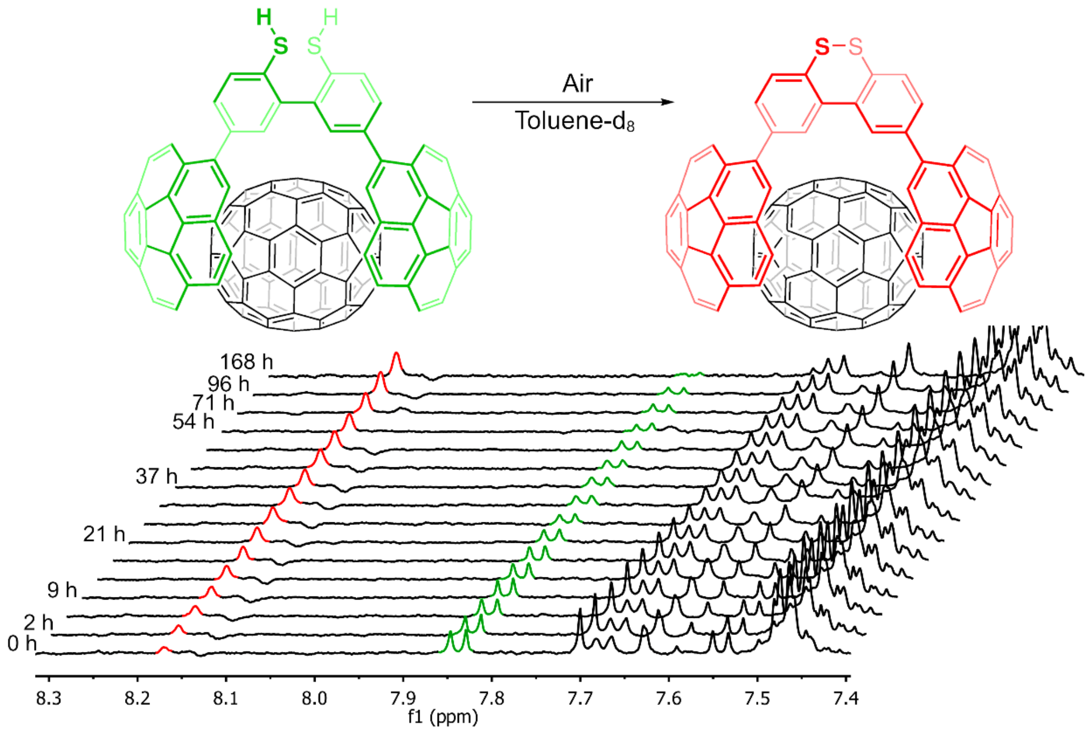

Figure S 186. Stacked ^{1}H NMR spectra of compound **13-SH** in toluene-d_8 in the presence of 10 eq of C_{70} monitored over time.

The graph below (Figure S 187) shows the evolution of the molar fraction of reduced species **13-SH**, according to above-described integrated rate equation, over the time exposed to the air in the absence and presence of C_{70}. Once the molar fraction of oxidized species **13-SS** was approximately steady, the system was switched back (vertical lines) with the appropriate chemical input as reported in the previous section. It is clearly shown that the oxidation reaction rate in the presence fullerene C_{70} is doubled when compared to that without fullerene since it completes twice as many cycles as the other system within the same time span.

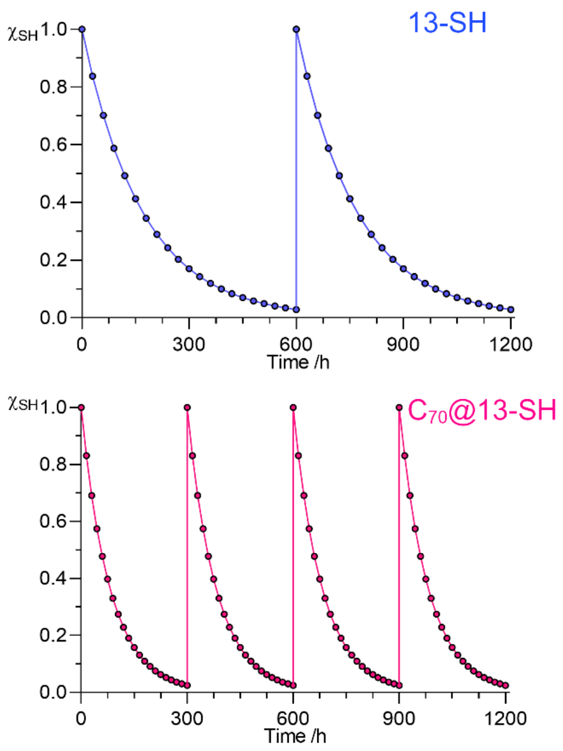

Figure S 187. Molar fraction variation of compound **13-SH** throughout different self-resetting redox cycles. Blue solid lines correspond to free host **13-SH** whereas pink lines to adduct C_{70}@**13-SH**. Vertical lines correspond to the addition of the appropriate reducing agent as described in the previous section.

In the case of compound **5-SH** the oxidation has not been observed within the experimental timeframe (ca. 1200 h). Thus, the only way to achieve dimer **5**$_2$**-SS** was by oxidation with the appropriate oxidizing reagent (see previously described method). Likewise, reduction to obtain compound **5-SH** back, was carried out with the suitable chemical effector, giving rise a purely horizontal linear trend which ultimately leads to an ON/OFF behavior (Figure S 188). This outcome was also observed for compound **13-SH** if the process is performed under inert atmosphere.

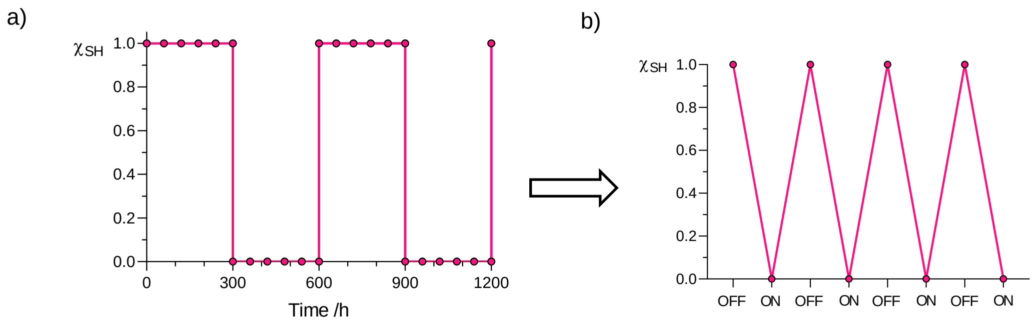

Figure S 188 (a) Molar fraction variation of compound **5-SH** throughout different redox cycles carried out at regular intervals of 300h. As commented in the text, no spontaneous transformation of one species to another has been observed. Vertical lines correspond to the addition of the appropriate oxidizing/reducing agent as described in the previous section. (b) Resulting ON/OFF behavior of the molecular switch **5-SH**/**5**$_2$**-SS**.

Computational Calculation Details

Generally, calculations were carried out by DFT methods with Grimme's B97D3 functional containing the Becke-Johnson damping empirical dispersion correction.[16-18] Ahlrichs' and Weigend's split valence triple zeta basis sets Def2TZVP was chosen.[19-20] The impact of the solvent was taken into account via the implicit Polarizable Continuum Model (PCM) [21] with toluene (ε=2.37) as the solvent of choice. Gaussian 16 package was used.[22]

Geometries of hosts **5-SS**, **13-SH**, **13-SS**, **13-SMe** and **13-S^{t}Bu** were optimized with above-described level of theory starting with single-crystal X-ray structures of the corresponding intermediates. Corannulene moieties were then grafted at their corresponding positions. In order to explore the most relevant conformers, several bonds (S-S, aryl C-C, aryl C-S) were systematically rotated followed by optimization of the whole molecule to provide a variety of geometries that were selected according to an energy criterion. Chemically significant conformers below 5 kcal/mol, with respect to the most stable one, are shown below.

Compound **5-SS** conformer 1 (E = -2793.0108789 a.u.)

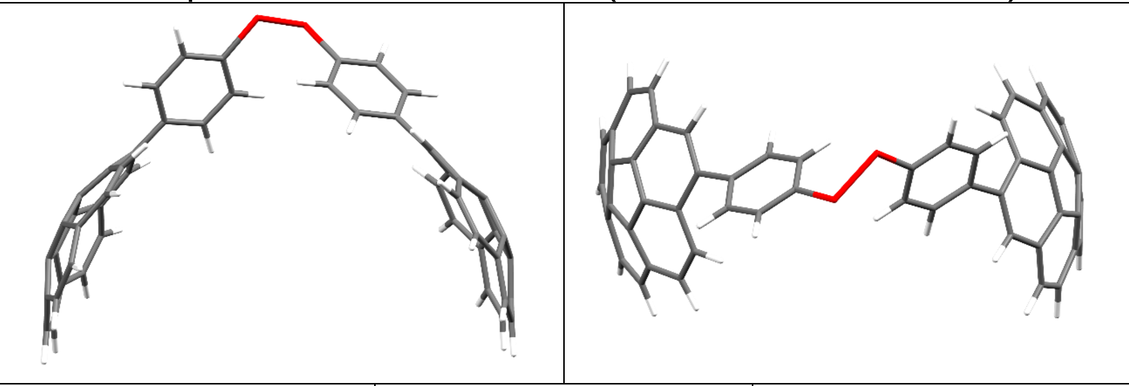

C	3.838077	2.215165	0.123077	C	6.155291	-0.212537	1.978705	C	-5.493229	0.502392	0.769326
C	2.650312	2.247635	0.871416	C	6.344626	-1.703379	-2.487977	C	-6.155848	-0.211908	-1.978453
C	1.677534	3.217875	0.648798	C	7.436314	-2.830662	1.960103	C	-6.343894	-1.704221	2.487778
C	1.878804	4.191373	-0.332206	C	7.554241	-3.752078	-0.803737	C	-7.436911	-2.830011	-1.960338
C	3.058400	4.179919	-1.090070	C	4.971602	0.323045	-2.105188	C	-7.554064	-3.752328	0.803229
C	4.017773	3.199122	-0.865016	C	5.207757	0.827121	1.671061	C	-4.878312	1.192835	-0.361260
C	4.878224	1.192725	0.361589	C	6.268068	-1.014583	3.175924	C	-4.970919	0.322282	2.105242
H	2.470276	1.481290	1.620493	C	5.378739	-0.724252	-2.923695	C	-5.208210	0.827639	-1.670755
S	0.688988	5.457047	-0.749740	C	6.528991	-3.060285	-2.950126	C	-6.268971	-1.013565	-3.175900
H	3.230027	4.941525	-1.847127	C	6.878306	-2.262953	3.166225	C	-5.377834	-0.725277	2.923520
H	4.936858	3.211262	-1.444174	C	7.651179	-4.219747	1.627827	C	-6.528149	-3.061273	2.949535
C	-1.677736	3.218133	-0.648775	C	7.105286	-4.036734	-2.147377	C	-6.879224	-2.261928	-3.166432
C	-2.650547	2.247917	-0.871376	C	7.708206	-4.659049	0.310199	C	-7.651706	-4.219201	-1.628458
C	-3.838121	2.215239	-0.122742	H	4.160276	0.957901	-2.449403	C	-7.104714	-4.037441	2.146639
C	-4.017616	3.198990	0.865591	H	4.672435	1.311872	2.484428	C	-7.708366	-4.658933	-0.310958
C	-3.058206	4.179755	1.090642	H	5.774687	-0.682970	4.087262	H	-4.159455	0.956982	2.449414
C	-1.878791	4.191394	0.332502	H	4.871435	-0.863833	-3.876157	H	-4.673135	1.312658	-2.484123
H	-2.470676	1.481749	-1.620673	H	6.114535	-3.354989	-3.912094	H	-5.775835	-0.681662	-4.087265
H	-4.936568	3.210995	1.444959	H	6.841842	-2.866601	4.070855	H	-4.870239	-0.865202	3.875777
H	-3.229669	4.941189	1.847909	H	7.667844	-4.960948	2.424289	H	-6.113401	-3.356306	3.911276
S	-0.688945	5.457058	0.750017	H	7.122870	-5.063303	-2.507719	H	-6.843023	-2.865289	-4.071265
C	6.556222	-0.332321	-0.446675	H	7.767774	-5.728841	0.120493	H	-7.668611	-4.960141	-2.425157
C	6.886685	-0.677400	0.893416	C	-6.556082	-0.332470	0.447002	H	-7.122212	-5.064129	2.506645
C	6.974454	-1.403465	-1.286182	C	-6.886943	-0.677102	-0.893106	H	-7.767900	-5.728786	-0.121587
C	7.508593	-1.954974	0.882819	C	-6.974083	-1.403889	1.286276	H	-0.763869	3.216691	-1.234089
C	7.564538	-2.404145	-0.465276	C	-7.508872	-1.954671	-0.882749	H	0.763524	3.216266	1.233887
C	5.493485	0.502665	-0.769045	C	-7.564436	-2.404282	0.465215				

Compound **5-SS** conformer 2 (E = -2793.0108789 a.u.)

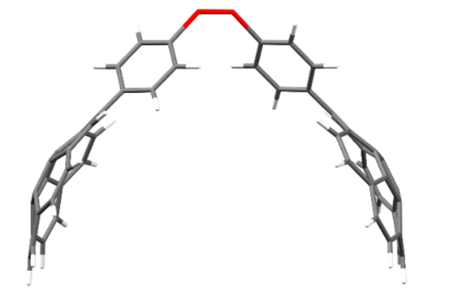

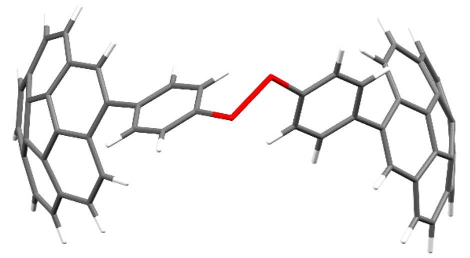

C	-3.838296	-2.214900	0.123065	C	-6.155697	0.212500	1.978860	C	5.494002	-0.502560	0.769050
C	-2.650420	-2.247141	0.871250	C	-6.345893	1.703143	-2.487841	C	6.155979	0.212239	-1.978759
C	-1.677555	-3.217284	0.648596	C	-7.437296	2.830344	1.960322	C	6.345525	1.703529	2.487753
C	-1.878852	-4.190919	-0.332267	C	-7.555756	3.751635	-0.803534	C	7.437518	2.830114	-1.960418
C	-3.058558	-4.179717	-1.089951	C	-4.972387	-0.322969	-2.105147	C	7.555591	3.751800	0.803325
C	-4.018021	-3.199004	-0.864868	C	-5.207991	-0.826979	1.671145	C	4.878660	-1.192652	-0.361522
C	-4.878567	-1.192588	0.361645	C	-6.268494	1.014569	3.176062	C	4.972100	-0.322656	2.105153
H	-2.470376	-1.480688	1.620212	C	-5.379837	0.724218	-2.923634	C	5.208257	-0.827220	-1.671028
S	-0.688924	-5.456485	-0.749832	C	-6.530595	3.059993	-2.950013	C	6.268918	1.014142	-3.176058
H	-3.230222	-4.941449	-1.846870	C	-6.879004	2.262804	3.166394	C	5.379419	0.724654	2.923548
H	-4.937199	-3.211347	-1.443872	C	-7.652509	4.219370	1.628024	C	6.530146	3.060446	2.949760
C	1.677750	-3.217476	-0.648668	C	-7.107010	4.036344	-2.147230	C	6.879400	2.262392	-3.166485
C	2.650628	-2.247345	-0.871322	C	-7.709788	4.658613	0.310389	C	7.652662	4.219190	-1.628288
C	3.838375	-2.214946	-0.122942	H	-4.160946	-0.957643	-2.449423	C	7.106660	4.036692	2.146920
C	4.017958	-3.198879	0.865189	H	-4.672492	-1.311613	2.484462	C	7.709758	4.658622	-0.310707
C	3.058478	-4.179570	1.090284	H	-5.774924	0.683093	4.087348	H	4.160620	-0.957293	2.449406
C	1.878899	-4.190929	0.332402	H	-4.872667	0.863893	-3.876154	H	4.672880	-1.311978	-2.484352
H	2.470689	-1.481027	-1.620448	H	-6.116310	3.354757	-3.912036	H	5.775477	0.682527	-4.087364
H	4.937039	-3.211102	1.444349	H	-6.842557	2.866495	4.070996	H	4.872113	0.864458	3.875976
H	3.230025	-4.941156	1.847378	H	-7.669238	4.960597	2.424460	H	6.115723	3.355340	3.911684
S	0.688952	-5.456483	0.749975	H	-7.124858	5.062897	-2.507606	H	6.843063	2.865953	-4.071178
C	-6.556963	0.332101	-0.446475	H	-7.769612	5.728385	0.120654	H	7.669487	4.960306	-2.424826
C	-6.887327	0.677160	0.893645	C	6.556905	0.332197	0.446614	H	7.124442	5.063295	2.507154
C	-6.975523	1.403130	-1.285965	C	6.887448	0.677070	-0.893510	H	7.769538	5.728422	-0.121119
C	-7.509517	1.954601	0.883080	C	6.975328	1.403355	1.286009	H	0.763748	-3.215821	-1.233773
C	-7.565725	2.403710	-0.465024	C	7.509609	1.954526	-0.883042	H	-0.763458	-3.215499	1.233549
C	-5.494091	-0.502682	-0.768941	C	7.565626	2.403827	0.465006				

Compound **5-SS** conformer 3 (E = -2793.010842 a.u.)

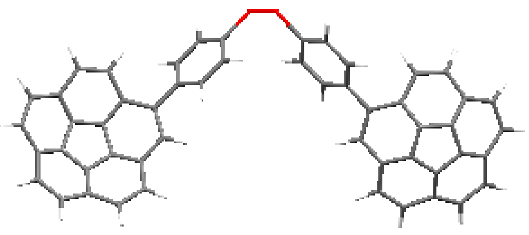

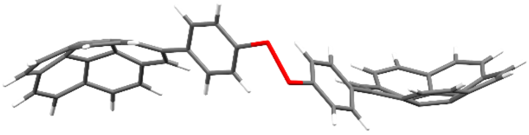

C	-3.733062	1.252309	-0.642791	C	-5.366060	-2.189040	-0.199612	C	6.160428	0.530752	0.886538
C	-2.749566	1.333835	0.356034	C	-9.040804	0.751214	-0.441133	C	5.366091	-2.189068	0.200107
C	-1.769694	2.322107	0.332057	C	-7.743377	-3.653581	0.636348	C	9.040846	0.751280	0.440378
C	-1.759378	3.264889	-0.697818	C	-10.017005	-1.834407	0.485440	C	7.743261	-3.653721	-0.636082
C	-2.735533	3.207305	-1.702570	C	-6.818494	1.808023	-0.728395	C	10.016897	-1.834490	-0.485945
C	-3.706921	2.213187	-1.668927	C	-4.407165	-1.119618	-0.300102	C	4.762398	0.191613	0.634479
C	-4.762314	0.191575	-0.634263	C	-5.277665	-3.421513	0.550339	C	6.818578	1.808106	0.727908
H	-2.763138	0.623089	1.177837	C	-8.188887	1.912914	-0.519660	C	4.407202	-1.119642	0.300610
S	-0.533888	4.554882	-0.866282	C	-10.319027	0.609779	0.218300	C	5.277562	-3.421673	-0.549610
H	-2.733169	3.934885	-2.510896	C	-6.411479	-4.118830	0.949250	C	8.188928	1.912980	0.518878
H	-4.449374	2.168057	-2.460713	C	-8.982518	-3.922583	1.327934	C	10.318940	0.609747	-0.219282
C	1.769560	2.321948	-0.331598	C	-10.784313	-0.622485	0.659946	C	6.411306	-4.119044	-0.948630
C	2.749441	1.333686	-0.355607	C	-10.066813	-3.055762	1.255443	C	8.982268	-3.922826	-1.327868
C	3.733131	1.252331	0.643042	H	-6.218797	2.711334	-0.663516	C	10.784154	-0.622588	-0.660809
C	3.707175	2.213373	1.669029	H	-3.368279	-1.315956	-0.044343	C	10.066565	-3.055978	-1.255746
C	2.735788	3.207491	1.702693	H	-4.306004	-3.761617	0.902689	H	6.218857	2.711399	0.662993
C	1.759434	3.264901	0.698124	H	-8.605790	2.894477	-0.303456	H	3.368267	-1.316039	0.045095
H	2.762862	0.622810	-1.177300	H	-10.888863	1.502041	0.469447	H	4.305834	-3.761856	-0.901701
H	4.449779	2.168380	2.460679	H	-6.288816	-4.982022	1.600337	H	8.605771	2.894513	0.302421
H	2.733584	3.935215	2.510890	H	-9.039904	-4.771533	2.006172	H	10.888718	1.501971	-0.470695
S	0.533931	4.554869	0.866628	H	-11.703166	-0.653908	1.241947	H	6.288521	-4.982353	-1.599538
C	-7.021861	-0.553539	-1.000141	H	-10.935594	-3.255996	1.879231	H	9.039530	-4.771893	-2.005971
C	-6.635374	-1.883172	-0.673061	C	7.022032	-0.553405	1.000016	H	11.702894	-0.654099	-1.242985
C	-8.425606	-0.445364	-0.788512	C	6.635498	-1.883099	0.673243	H	10.935224	-3.256310	-1.879672
C	-7.794931	-2.594823	-0.263116	C	8.425733	-0.445247	0.788083	H	1.025731	2.371535	-1.120436
C	-8.903016	-1.706533	-0.335701	C	7.794982	-2.594804	0.263187	H	-1.026009	2.371837	1.121023
C	-6.160295	0.530649	-0.886668	C	8.903069	-1.706487	0.335394				

Compound **13-SH** conformer 1 (E = -2793.009015 a.u.)

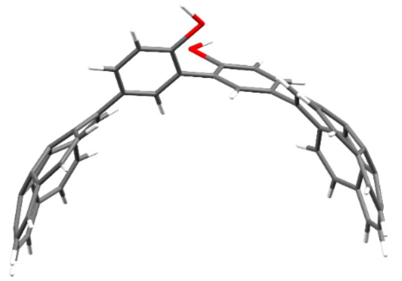

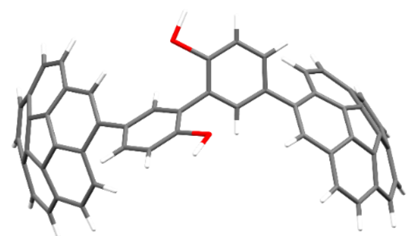

C	-2.912417	1.887779	0.817507	C	-5.631424	0.508248	-1.503969	C	4.858932	0.322091	-1.300654
C	-1.746476	1.913626	0.040251	C	-6.108376	-2.078016	2.406607	C	5.631429	0.508373	1.503917
C	-0.604079	2.613946	0.434284	C	-7.372770	-1.744994	-2.125201	C	6.108317	-2.078193	-2.406510
C	-0.617514	3.315696	1.657587	C	-7.668916	-3.344121	0.294912	C	7.372876	-1.744796	2.125255
C	-1.772548	3.286001	2.452091	C	-4.383141	-0.303219	2.513859	C	7.668989	-3.344102	-0.294767
C	-2.897846	2.584532	2.037425	C	-4.509476	1.239316	-0.975309	C	4.122932	1.170690	-0.367765
C	-4.122981	1.170708	0.367710	C	-5.885112	0.093571	-2.865200	C	4.383060	-0.303411	-2.513856
H	-1.712980	1.358571	-0.892909	C	-4.979357	-1.442795	3.041614	C	4.509473	1.239402	0.975235
S	0.832519	4.221438	2.129178	C	-6.541591	-3.453794	2.498066	C	5.885229	0.093824	2.865165
H	-1.796641	3.827364	3.393819	C	-6.714812	-0.981213	-3.160668	C	4.979284	-1.443019	-3.041539
H	-3.790254	2.596738	2.656868	C	-7.840763	-3.110753	-2.166037	C	6.541614	-3.453954	-2.497896
C	0.604030	2.613933	-0.434369	C	-7.285279	-4.057239	1.491686	C	6.714971	-0.980922	3.160686
C	1.746433	1.913636	-0.040312	C	-7.982826	-3.873056	-1.012287	C	7.840958	-3.110524	2.166160
C	2.912366	1.887759	-0.817580	H	-3.468476	0.057909	2.975199	C	7.285364	-4.057301	-1.491502
C	2.897792	2.584471	-2.037516	H	-3.890505	1.822407	-1.653721	C	7.983006	-3.872910	1.012460
C	1.772487	3.285920	-2.452205	H	-5.334439	0.565599	-3.676288	H	3.468397	0.057682	-2.975227
C	0.617454	3.315637	-1.657702	H	-4.508982	-1.924270	3.896594	H	3.890493	1.822536	1.653605
H	1.712950	1.358628	0.892877	H	-6.192118	-4.067069	3.326178	H	5.334621	0.565919	3.676260
H	3.790197	2.596660	-2.656963	H	-6.786159	-1.314586	-4.194031	H	4.508904	-1.924533	-3.896495
H	1.796578	3.827251	-3.393952	H	-7.990255	-3.593264	-3.129840	H	6.192169	-4.067299	-3.325968
S	-0.832584	4.221347	-2.129322	H	-7.493309	-5.122720	1.565784	H	6.786394	-1.314158	4.194087
C	-6.056903	-0.190150	0.817432	H	-8.238793	-4.925891	-1.112531	H	7.990548	-3.592958	3.129987
C	-6.440463	-0.094559	-0.549305	C	6.056824	-0.190227	-0.817452	H	7.493460	-5.122772	-1.565564
C	-6.668142	-1.356361	1.359233	C	6.440421	-0.094525	0.549261	H	8.239055	-4.925718	1.112783
C	-7.286737	-1.195328	-0.851145	C	6.668066	-1.356463	-1.359182	H	0.292091	4.769302	3.233736
C	-7.429455	-1.975493	0.329302	C	7.286725	-1.195251	0.851157	H	-0.292165	4.769173	-3.233903
C	-4.859015	0.322188	1.300616	C	7.429429	-1.975498	-0.329232				

Compound **13-SH** conformer 2 (E = -2793.0097517 a.u.)

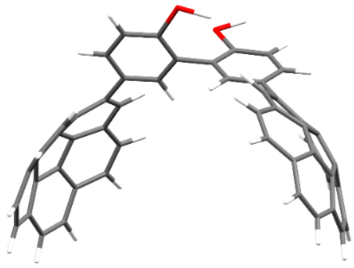

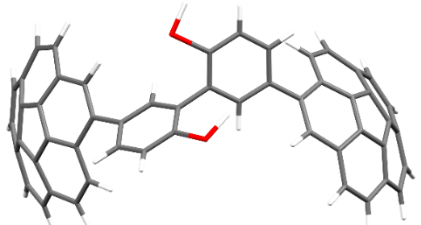

C	2.916705	1.965828	-0.766094	C	5.530294	0.405865	1.562814	C	-4.804468	0.323353	1.272483
C	1.732772	1.999862	-0.017841	C	6.005524	-2.052281	-2.429635	C	-5.544904	0.450534	-1.544574
C	0.619029	2.747523	-0.411129	C	7.173997	-1.931475	2.138320	C	-6.001851	-2.095541	2.394686
C	0.689228	3.501088	-1.602258	C	7.468901	-3.451279	-0.332511	C	-7.215325	-1.857740	-2.156874
C	1.866271	3.466578	-2.366098	C	4.348609	-0.212291	-2.509274	C	-7.499195	-3.431998	0.280850
C	2.956079	2.709882	-1.957556	C	4.448121	1.196459	1.036798	C	-4.080221	1.179520	0.337358
C	4.090842	1.190688	-0.315978	C	5.736837	-0.067154	2.912953	C	-4.327559	-0.272484	2.500084
H	1.658769	1.410054	0.891546	C	4.915613	-1.353093	-3.065648	C	-4.450663	1.219244	-1.011270
S	-0.632381	4.515245	-2.199381	C	6.390485	-3.438948	-2.562597	C	-5.769077	0.011091	-2.903164
H	1.930214	4.052371	-3.279571	C	6.519913	-1.181959	3.186579	C	-4.899174	-1.421065	3.035375
H	3.863933	2.716991	-2.554092	C	7.591072	-3.314262	2.138630	C	-6.398229	-3.481422	2.499451
C	-0.605162	2.726466	0.434308	C	7.087875	-4.105810	-1.563118	C	-6.565092	-1.089983	-3.194267
C	-1.720989	1.987608	0.032790	C	7.732485	-4.038748	0.960702	C	-7.645086	-3.236344	-2.185067
C	-2.896849	1.937036	0.793907	H	3.459143	0.199468	-2.977412	C	-7.111797	-4.118390	1.491959
C	-2.920165	2.650180	2.004891	H	3.834765	1.777226	1.722250	C	-7.781226	-3.986777	-1.022900
C	-1.822256	3.389386	2.426734	H	5.184893	0.395176	3.728780	H	-3.429393	0.119656	2.968489
C	-0.656541	3.442189	1.648115	H	4.448842	-1.785208	-3.948494	H	-3.838852	1.808981	-1.690557
H	-1.657593	1.426901	-0.895644	H	6.038338	-4.008197	-3.420496	H	-5.221108	0.487249	-3.713641
H	-3.821407	2.643355	2.611422	H	6.554912	-1.554988	4.208189	H	-4.427133	-1.878022	3.902753
H	-1.875193	3.940334	3.361701	H	7.700262	-3.836792	3.086863	H	-6.042815	-4.073736	3.340182
S	0.765252	4.386724	2.124830	H	7.258660	-5.174936	-1.671918	H	-6.613749	-1.438928	-4.223757
C	5.984942	-0.222799	-0.772990	H	7.947671	-5.103302	1.027212	H	-7.768513	-3.735576	-3.144005
C	6.339314	-0.191318	0.604602	C	-5.981177	-0.228355	0.780785	H	-7.291340	-5.188150	1.577691
C	6.566298	-1.390313	-1.344055	C	-6.349252	-0.161595	-0.591965	H	-8.006735	-5.047528	-1.111938
C	7.137910	-1.332964	0.884006	C	-6.567482	-1.403513	1.330614	H	-1.459372	4.323267	-1.155505
C	7.280145	-2.074325	-0.321219	C	-7.161322	-1.288991	-0.889457	H	0.202655	4.929153	3.221073
C	4.817889	0.350383	-1.263264	C	-7.298057	-2.056932	0.299627				

Compound **13-SH** conformer 3 (E = -2793.0097516 a.u.)

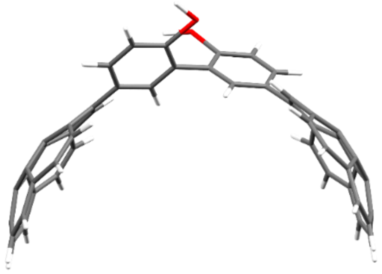
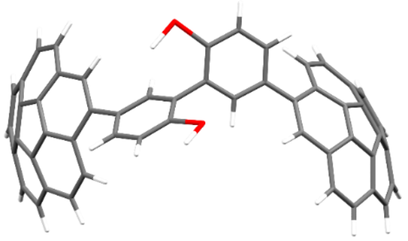

C	-2.916618	1.966011	0.765800	C	-5.529738	0.405368	-1.563190	C	4.804408	0.323503	-1.272227
C	-1.732577	2.000089	0.017717	C	-6.005362	-2.052086	2.429631	C	5.544516	0.449992	1.544950
C	-0.619017	2.747972	0.411051	C	-7.173227	-1.932171	-2.138499	C	6.001496	-2.095380	-2.394756
C	-0.689487	3.501759	1.602029	C	-7.468377	-3.451548	0.332565	C	7.214448	-1.858703	2.156999
C	-1.866675	3.467254	2.365663	C	-4.348564	-0.211989	2.509162	C	7.498333	-3.432526	-0.281002
C	-2.956296	2.710323	1.957097	C	-4.447691	1.196126	-1.037169	C	4.080187	1.179603	-0.337016
C	-4.090602	1.190645	0.315667	C	-5.736068	-0.067909	-2.913272	C	4.327541	-0.272009	-2.500000
H	-1.658326	1.410155	-0.891566	C	-4.915574	-1.352727	3.065660	C	4.450471	1.218992	1.011667
S	0.631895	4.516168	2.199161	C	-6.390258	-3.438753	2.562791	C	5.768446	0.010241	2.903480
H	-1.930838	4.053266	3.278983	C	-6.519041	-1.182809	-3.186804	C	4.899019	-1.420585	-3.035447
H	-3.864261	2.717420	2.553460	C	-7.590227	-3.314979	-2.138616	C	6.397629	-3.481314	-2.499744
C	0.605339	2.726999	-0.434173	C	-7.087477	-4.105836	1.563340	C	6.564224	-1.091034	3.194463
C	1.721002	1.987910	-0.032637	C	-7.731757	-4.039263	-0.960576	C	7.643954	-3.237391	2.184971
C	2.896999	1.937408	-0.793562	H	-3.459176	0.199887	2.977340	C	7.110956	-4.118612	-1.492290
C	2.920616	2.650893	-2.004336	H	-3.834298	1.776829	-1.722639	C	7.780100	-3.987617	1.022672
C	1.822871	3.390356	-2.426173	H	-5.184039	0.394305	-3.729107	H	3.429499	0.120379	-2.968434
C	0.657013	3.443051	-1.647768	H	-4.448887	-1.784670	3.948636	H	3.838681	1.808711	1.690986
H	1.657385	1.426960	0.895636	H	-6.038190	-4.007827	3.420838	H	5.220464	0.486336	3.713986
H	3.821971	2.644160	-2.610700	H	-6.553876	-1.556026	-4.208351	H	4.427003	-1.877291	-3.902971
H	1.876050	3.941573	-3.360968	H	-7.699261	-3.837686	-3.086770	H	6.042203	-4.073395	-3.340634
S	-0.764657	4.387757	-2.124454	H	-7.258212	-5.174953	1.672310	H	6.612688	-1.440193	4.223889
C	-5.984671	-0.222893	0.772666	H	-7.946876	-5.103840	-1.026925	H	7.767171	-3.736836	3.143825
C	-6.338855	-0.191688	-0.604981	C	5.980961	-0.228507	-0.780493	H	7.290309	-5.188388	-1.578204
C	-6.566031	-1.390344	1.343860	C	6.348875	-0.162088	0.592319	H	8.005401	-5.048428	1.111525
C	-7.137343	-1.333431	-0.884288	C	6.567124	-1.403661	-1.330481	H	1.459114	4.323976	1.155507
C	-7.279699	-2.074585	0.321051	C	7.160708	-1.289689	0.889688	H	-0.201916	4.930379	-3.220529
C	-4.817719	0.350450	1.262998	C	7.297452	-2.057418	-0.299533				

Compound **13-SS** (E = -2791.8080856 a.u.)

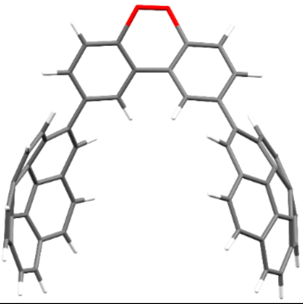
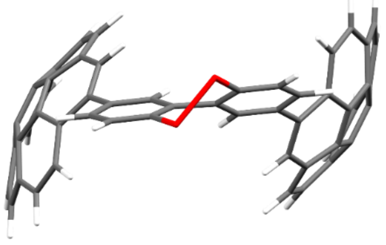

C	-2.822193	2.371506	-0.367276	C	-4.596151	0.604787	0.074605	C	5.237442	-2.567674	1.459404
C	-1.424239	2.465921	-0.381064	C	-3.614519	-0.883860	-2.232895	C	5.989645	-2.678039	0.257721
C	-0.740718	3.637077	-0.025094	C	-6.110201	-1.252553	1.748256	C	4.596151	0.604785	-0.074606
C	-1.520145	4.775095	0.293919	C	-4.538075	-3.638308	-2.004578	C	3.614518	-0.883855	2.232897
C	-2.916153	4.698024	0.313534	C	-6.083374	-3.865931	0.457585	C	6.110201	-1.252559	-1.748252
C	-3.561532	3.504887	0.008936	C	-4.874803	0.876488	1.466991	C	4.538072	-3.638305	2.004588
C	-3.507295	1.118557	-0.751261	C	-3.049930	0.384201	-1.850224	C	6.083372	-3.865934	-0.457574
H	-0.849387	1.590985	-0.667275	C	-3.009212	-1.955662	-2.990506	C	3.507296	1.118558	0.751259
S	-0.732733	6.299949	0.736739	C	-5.597697	-0.005500	2.261918	C	4.874805	0.876484	-1.466992
H	-3.494763	5.585588	0.554158	C	-6.434154	-2.462783	2.468774	C	3.049930	0.384205	1.850223
H	-4.646778	3.462750	0.011703	C	-3.449644	-3.268907	-2.880491	C	3.009210	-1.955655	2.990512
C	0.740720	3.637077	0.025083	C	-4.814413	-4.917324	-1.393146	C	5.597699	-0.005507	-2.261916
C	1.424240	2.465922	0.381057	C	-6.421116	-3.708399	1.853728	C	6.434154	-2.462790	-2.468766
C	2.822194	2.371506	0.367270	C	-5.551750	-5.026000	-0.219769	C	3.449641	-3.268901	2.880500
C	3.561533	3.504885	-0.008946	H	-4.421839	1.741983	1.941933	C	4.814410	-4.917322	1.393159
C	2.916155	4.698022	-0.313548	H	-2.188165	0.764495	-2.394171	C	6.421115	-3.708406	-1.853718
C	1.520148	4.775093	-0.293933	H	-2.120745	-1.751957	-3.584563	C	5.551748	-5.026001	0.219782
H	0.849388	1.590987	0.667272	H	-5.683191	0.207695	3.325508	H	4.421842	1.741978	-1.941936
H	4.646780	3.462748	-0.011713	H	-6.593131	-2.414638	3.544080	H	2.188164	0.764501	2.394169
H	3.494766	5.585584	-0.554176	H	-2.890644	-4.049492	-3.392524	H	2.120743	-1.751948	3.584568
S	0.732735	6.299946	-0.736759	H	-4.350581	-5.813196	-1.800829	H	5.683193	0.207686	-3.325507
C	-5.260562	-0.500131	-0.443301	H	-6.570414	-4.593918	2.468189	H	6.593132	-2.414649	-3.544072
C	-4.788131	-1.224447	-1.573083	H	-5.639997	-6.003232	0.250488	H	2.890640	-4.049484	3.392534
C	-6.002432	-1.402055	0.371008	C	5.260562	-0.500132	0.443304	H	4.350577	-5.813193	1.800843
C	-5.237444	-2.567675	-1.459397	C	4.788130	-1.224445	1.573087	H	6.570412	-4.593926	-2.468176
C	-5.989646	-2.678038	-0.257713	C	6.002432	-1.402057	-0.371003	H	5.639994	-6.003234	-0.250473

Compound **13-SMe** conformer 1 (E = -2871.6156105 a.u.)

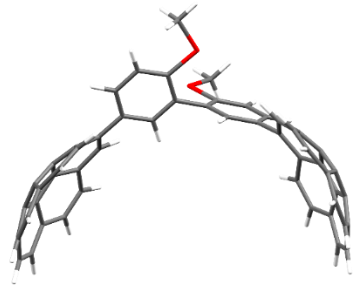

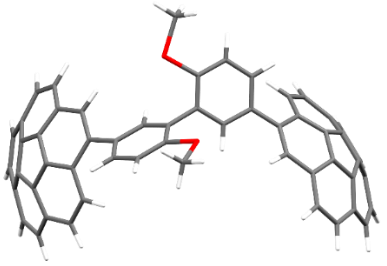

C	2.911032	1.709968	-0.778841	C	7.228017	-2.108373	2.145248	C	-7.550773	-3.632779	0.319210
C	1.727552	1.740559	-0.025509	C	7.550774	-3.632778	-0.319209	C	-4.095180	0.953816	0.324581
C	0.613785	2.478156	-0.422077	C	4.377768	-0.455621	-2.510472	C	-4.377767	-0.455621	2.510473
C	0.664476	3.222949	-1.624023	C	4.451011	0.969473	1.028826	C	-4.451011	0.969472	-1.028825
C	1.838372	3.189287	-2.391069	C	5.758345	-0.265838	2.910835	C	-5.758344	-0.265839	-2.910834
C	2.937372	2.444741	-1.973708	C	4.964065	-1.589099	-3.061995	C	-4.964064	-1.589099	3.061996
C	4.095180	0.953816	-0.324580	C	6.473941	-3.647465	-2.550015	C	-6.473940	-3.647465	2.550017
H	1.660968	1.162562	0.891973	C	6.559959	-1.366064	3.189806	C	-6.559958	-1.366065	-3.189806
S	-0.772532	4.156223	-2.057526	C	7.668230	-3.483885	2.151385	C	-7.668228	-3.483886	-2.151384
H	1.911272	3.753749	-3.313383	C	7.181757	-4.298514	-1.547439	C	-7.181756	-4.298515	1.547440
H	3.843063	2.455768	-2.574019	C	7.823047	-4.210632	0.976485	C	-7.823046	-4.210633	-0.976484
C	-0.613785	2.478156	0.422078	H	3.480802	-0.061481	-2.979412	H	-3.480802	-0.061481	2.979413
C	-1.727552	1.740559	0.025510	H	3.827118	1.542243	1.711508	H	-3.827118	1.542242	-1.711508
C	-2.911032	1.709967	0.778842	H	5.197087	0.189686	3.724177	H	-5.197087	0.189685	-3.724176
C	-2.937372	2.444740	1.973708	H	4.504192	-2.033444	-3.942434	H	-4.504191	-2.033443	3.942435
C	-1.838372	3.189287	2.391069	H	6.131856	-4.226156	-3.405701	H	-6.131855	-4.226157	3.405702
C	-0.664476	3.222950	1.624023	H	6.599674	-1.734731	4.212860	H	-6.599673	-1.734732	-4.212859
H	-1.660968	1.162562	-0.891972	H	7.785070	-4.000800	3.101810	H	-7.785068	-4.000802	-3.101809
H	-3.843063	2.455768	2.574019	H	7.370415	-5.365097	-1.651796	H	-7.370414	-5.365097	1.651797
H	-1.911272	3.753749	3.313383	H	8.055949	-5.271181	1.047449	H	-8.055947	-5.271183	-1.047447
S	0.772531	4.156224	2.057526	C	-6.013346	-0.431058	0.773825	C	-0.253633	5.044517	-3.548680
C	6.013346	-0.431058	-0.773824	C	-6.366074	-0.388125	-0.603878	H	0.604877	5.691226	-3.347818
C	6.366074	-0.388124	0.603879	C	-6.614401	-1.591211	1.339714	H	-1.110707	5.662995	-3.827231
C	6.614402	-1.591210	-1.339713	C	-7.182991	-1.515363	-0.888546	H	-0.028595	4.357971	-4.369389
C	7.182992	-1.515362	0.888547	C	-7.338750	-2.259135	0.313608	C	0.253623	5.044537	3.548665
C	7.338751	-2.259134	-0.313607	C	-4.837154	0.120694	1.267079	H	1.110716	5.662976	3.827247
C	4.837154	0.120695	-1.267078	C	-5.545360	0.198761	-1.558711	H	-0.604851	5.691286	3.347777
C	5.545361	0.198761	1.558712	C	-6.065606	-2.266921	2.423008	H	0.028526	4.357999	4.369365
C	6.065607	-2.266921	-2.423007	C	-7.228016	-2.108374	-2.145247				

Compound **13-SMe** conformer 2 (E = -2871.6151483 a.u.)

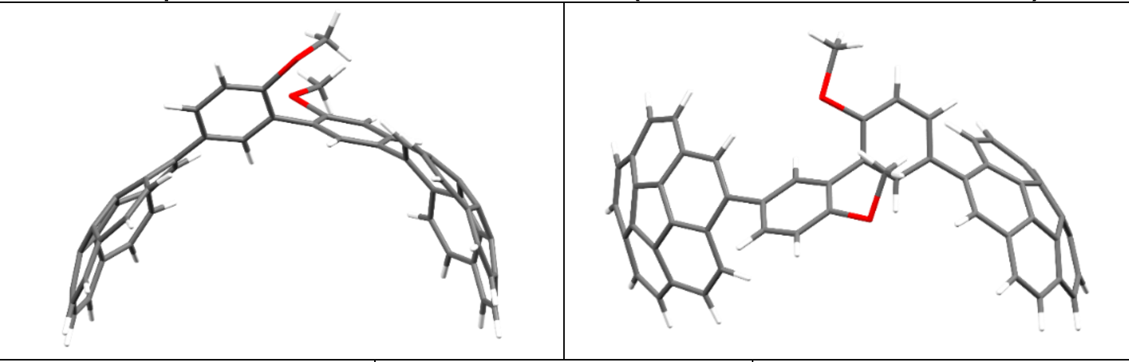

C	-2.848188	1.678191	0.702892	C	-7.434011	-2.026991	-1.941268	C	7.725641	-3.519351	0.013184
C	-1.707842	1.655645	-0.112655	C	-7.696471	-3.495303	0.563927	C	4.136430	0.912970	-0.639288
C	-0.557288	2.378341	0.196720	C	-4.310702	-0.401446	2.546356	C	4.289856	-0.911016	-2.508971
C	-0.521856	3.152682	1.380007	C	-4.496608	0.962449	-1.013574	C	4.597084	1.214215	0.647206
C	-1.646907	3.159059	2.218314	C	-5.935029	-0.254822	-2.809304	C	6.089334	0.415780	2.625001
C	-2.787505	2.436837	1.882250	C	-4.911760	-1.502497	3.145656	C	4.866730	-2.125103	-2.863663
C	-4.079199	0.956627	0.322302	C	-6.517688	-3.511816	2.742561	C	6.476580	-4.010889	-2.068735
H	-1.709388	1.057906	-1.018824	C	-6.788753	-1.328767	-3.029662	C	6.945156	-0.591125	3.054588
S	0.931902	4.113386	1.686964	C	-7.924255	-3.384907	-1.900919	C	8.034206	-2.861283	2.382182
H	-1.652488	3.746123	3.129219	C	-7.294811	-4.153126	1.786132	C	7.281909	-4.430041	-1.017183
H	-3.656806	2.486092	2.532242	C	-8.050289	-4.084884	-0.706527	C	8.118238	-3.812680	1.372143
C	0.602899	2.322233	-0.731675	H	-3.378984	-0.033171	2.966103	H	3.347559	-0.634875	-2.973316
C	1.780878	1.704921	-0.299855	H	-3.883017	1.497322	-1.735545	H	4.009972	1.905291	1.248200
C	2.895122	1.542404	-1.133684	H	-5.395823	0.164973	-3.656004	H	5.580602	1.021564	3.372104
C	2.798768	2.014194	-2.453141	H	-4.427991	-1.948297	4.012458	H	4.354140	-2.747966	-3.593955
C	1.648347	2.655133	-2.895738	H	-6.157324	-4.087992	3.592388	H	6.086542	-4.758820	-2.756085
C	0.540116	2.825943	-2.050398	H	-6.889598	-1.713734	-4.042443	H	7.078116	-0.739405	4.124363
H	1.810493	1.304819	0.709477	H	-8.104384	-3.913313	-2.834954	H	8.242582	-3.168965	3.404935
H	3.650670	1.919290	-3.120620	H	-7.517144	-5.209973	1.919150	H	7.495857	-5.492180	-0.916444
H	1.607521	3.058423	-3.903819	H	-8.324937	-5.137017	-0.746393	H	8.389815	-4.832061	1.638758
S	-0.882674	3.617933	-2.760206	C	6.055304	-0.504513	-0.956223	C	0.498134	5.064049	3.166715
C	-6.023284	-0.346245	0.886135	C	6.514382	-0.172627	0.348970	H	-0.380508	5.691487	2.994071
C	-6.438225	-0.314021	-0.474470	C	6.646714	-1.747141	-1.321161	H	1.363445	5.703504	3.359191
C	-6.639403	-1.472956	1.501138	C	7.387635	-1.203932	0.788411	H	0.337193	4.413940	4.030821
C	-7.308748	-1.414353	-0.699518	C	7.471079	-2.177594	-0.244696	C	-1.395309	4.833855	-1.502594
C	-7.435031	-2.130840	0.522446	C	4.827063	-0.084226	-1.451872	H	-1.962071	5.595723	-2.044830
C	-4.806002	0.169637	1.314378	C	5.754900	0.587294	1.229324	H	-2.023957	4.382630	-0.734129
C	-5.642402	0.224801	-1.477484	C	6.035805	-2.640130	-2.193190	H	-0.514685	5.291403	-1.046804
C	-6.065711	-2.149837	2.570717	C	7.551121	-1.523434	2.131550				

Compound **13-SMe** conformer 3 (E = -2871.6081727 a.u.)

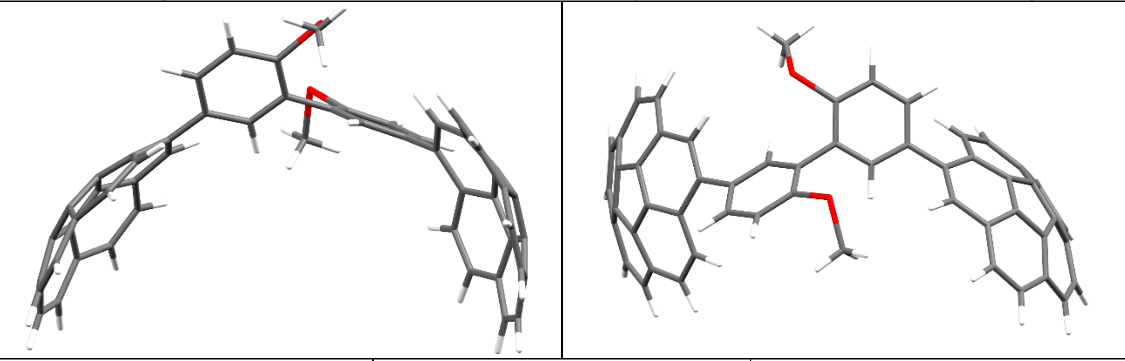

C	-2.839247	1.702063	1.007767	C	-7.566244	-1.569367	-1.933201	C	7.673297	-3.432420	-0.307423
C	-1.750452	1.793943	0.135760	C	-7.672842	-3.432841	0.307100	C	4.094227	1.043375	-0.591168
C	-0.562860	2.447138	0.485527	C	-4.190693	-0.657800	2.575689	C	4.191343	-0.657114	-2.575988
C	-0.463870	3.038509	1.761217	C	-4.591148	1.266126	-0.697438	C	4.590549	1.265692	0.697958
C	-1.552395	2.948950	2.643814	C	-6.131534	0.344019	-2.582288	C	6.130525	0.343120	2.582920
C	-2.714532	2.281562	2.283571	C	-4.750857	-1.850200	3.019348	C	4.751834	-1.849258	-3.019926
C	-4.094409	1.043400	0.591453	C	-6.367961	-3.789535	2.382360	C	6.369086	-3.788556	-2.383199
H	-1.809026	1.315970	-0.836906	C	-6.991377	-0.691694	-2.927097	C	6.990432	-0.692585	2.927596
S	0.977761	3.978167	2.227149	C	-8.047112	-2.922433	-2.088828	C	8.046779	-2.922842	2.088805
H	-1.490766	3.435872	3.612959	C	-7.197040	-4.276385	1.379477	C	7.197955	-4.275641	-1.380258
H	-3.552787	2.244946	2.973821	C	-8.098706	-3.810598	-1.020730	C	8.098835	-3.810599	1.020391
C	0.562657	2.447104	-0.485683	H	-3.239581	-0.347355	2.998836	H	3.240301	-0.346680	-2.999296
C	1.750189	1.793841	-0.135807	H	-4.024170	1.923785	-1.352651	H	4.023265	1.923002	1.353264
C	2.839124	1.702064	-1.007636	H	-5.647917	0.907066	-3.378033	H	5.646594	0.905800	3.378734
C	2.714645	2.281728	-2.283395	H	-4.216933	-2.423726	3.774384	H	4.218220	-2.422591	-3.775326
C	1.552528	2.949069	-2.643783	H	-5.956215	-4.492043	3.104093	H	5.957672	-4.490862	-3.105319
C	0.463838	3.038490	-1.761367	H	-7.152412	-0.904845	-3.981951	H	7.151196	-0.906099	3.982417
H	1.808624	1.315841	0.836857	H	-8.280636	-3.293042	-3.084896	H	8.280075	-3.293785	3.084802
H	3.553039	2.245231	-2.973479	H	-7.406716	-5.343725	1.349861	H	7.407808	-5.342956	-1.350984
H	1.490971	3.436054	-3.612905	H	-8.370990	-4.845524	-1.217525	H	8.371236	-4.845553	1.216881
S	-0.977792	3.977943	-2.227828	C	5.997603	-0.356117	-1.045970	C	1.542148	3.053134	3.692582
C	-5.997450	-0.356534	1.046306	C	6.491802	-0.107304	0.264818	H	0.785615	3.050031	4.480775
C	-6.492069	-0.107317	-0.264246	C	6.572010	-1.576312	-1.502083	H	2.433876	3.572289	4.055390
C	-6.571522	-1.576994	1.502135	C	7.369576	-1.167723	0.617291	H	1.803582	2.026453	3.421003
C	-7.369772	-1.167748	-0.616857	C	7.420647	-2.076169	-0.475631	C	-1.542947	3.051011	-3.691799
C	-7.420373	-2.076611	0.475742	C	4.759334	0.098735	-1.482549	H	-2.434605	3.569926	-4.055122
C	-4.759123	0.098352	1.482689	C	5.760535	0.600941	1.209711	H	-0.786567	3.046577	-4.480136
C	-5.761188	0.601392	-1.209092	C	5.933802	-2.410724	-2.411762	H	-1.804615	2.024772	-3.418761
C	-5.932907	-2.411638	2.411314	C	7.565731	-1.569798	1.933543				

Compound **13-S^{t}Bu** conformer 1 (E = -3107.4379205 a.u.)

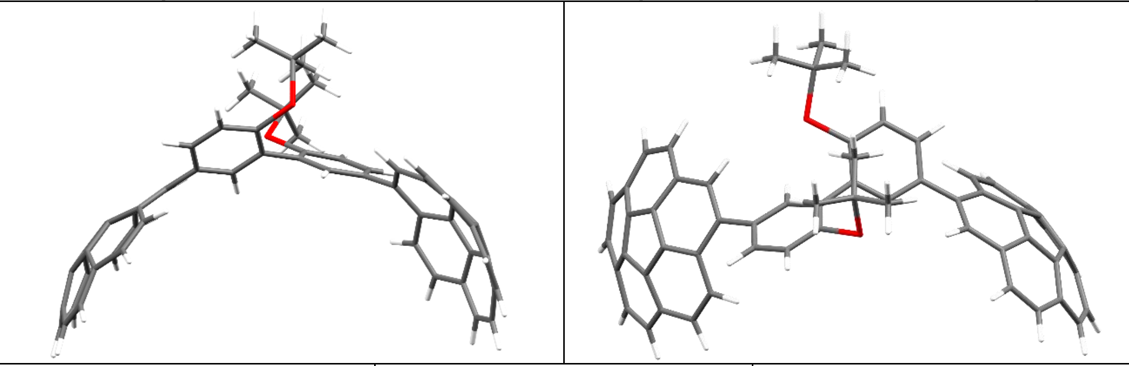

C	2.866081	0.736107	-1.290977	C	6.811868	-4.597375	-2.028931	H	-4.016131	0.980111	-1.572566
C	1.772116	0.862544	-0.426661	C	7.166722	-0.864021	2.879953	H	-5.733479	-0.111743	-3.473295
C	0.546434	1.405948	-0.830203	C	8.398406	-3.086650	2.302303	H	-4.531338	-2.753101	3.995120
C	0.396377	1.805323	-2.180986	C	7.671951	-4.897773	-0.980103	H	-6.448145	-4.722390	3.557520
C	1.481756	1.648432	-3.057668	C	8.523934	-4.086493	1.344843	H	-7.393398	-1.842180	-3.877204
C	2.698565	1.133782	-2.626574	H	3.427700	-1.505931	-3.027805	H	-8.714680	-4.010058	-2.726181
C	4.162720	0.220786	-0.804621	H	4.009899	1.305528	1.029254	H	-7.981833	-5.632573	1.906502
H	1.867354	0.503986	0.593529	H	5.696969	0.663308	3.147882	H	-8.922217	-5.336645	-0.700177
S	-1.127623	2.444355	-2.850455	H	4.567112	-3.575733	-3.559811	C	-0.978190	4.301736	-2.635584
H	1.358496	1.950755	-4.093045	H	6.458263	-5.409220	-2.661204	C	-0.892233	4.655305	-1.152585
H	3.530007	1.054926	-3.321354	H	7.337058	-0.941740	3.951859	C	-2.276976	4.837478	-3.249008
C	-0.542392	1.510769	0.175211	H	8.654782	-3.319224	3.333921	C	0.239934	4.832725	-3.390658
C	-1.762717	0.883050	-0.083601	H	7.963367	-5.934827	-0.826435	C	0.931289	4.452081	2.871966
C	-2.843916	0.935225	0.803583	H	8.874465	-5.067321	1.659672	C	2.241255	5.235877	2.678534
C	-2.649830	1.607038	2.020158	C	-6.157409	-0.845676	1.061603	C	-0.254795	5.323930	2.459488
C	-1.434845	2.211355	2.317192	C	-6.640717	-0.699141	-0.268681	C	0.836953	3.993617	4.332356
C	-0.369417	2.202971	1.402639	C	-6.828616	-1.956454	1.647366	H	0.187940	4.579247	-4.453938
H	-1.865094	0.325366	-1.007779	C	-7.607637	-1.713441	-0.504028	H	1.168391	4.425410	-2.979871
H	-3.464457	1.669912	2.736477	C	-7.725920	-2.490553	0.681223	H	0.280689	5.925750	-3.295283
H	-1.325150	2.704969	3.272630	C	-4.880933	-0.453145	1.446142	H	-0.870172	5.747572	-1.040436
S	1.184437	3.008424	1.703558	C	-5.858352	-0.164324	-1.284532	H	0.017390	4.253210	-0.698780
C	6.169854	-1.071107	-1.101473	C	-6.255087	-2.739060	2.642332	H	-1.751053	4.265483	-0.599868
C	6.637495	-0.632974	0.168968	C	-7.848209	-2.238415	-1.768786	H	-3.153959	4.448378	-2.720924
C	6.839304	-2.287931	-1.414332	C	-8.094388	-3.830604	0.661559	H	-2.287783	5.931076	-3.170226
C	7.593917	-1.573194	0.638708	C	-4.146810	0.333073	0.459000	H	-2.358106	4.569927	-4.307854
C	7.720004	-2.596670	-0.340665	C	-4.367972	-1.134341	2.613234	H	3.115073	4.619347	2.916386
C	4.902066	-0.767863	-1.582694	C	-4.633055	0.452259	-0.848202	H	2.242323	6.099320	3.353854
C	5.848620	0.120869	1.028475	C	-6.259579	-0.538551	-2.621837	H	2.339309	5.597750	1.650753
C	6.271473	-3.270261	-2.216663	C	-5.023209	-2.219411	3.184323	H	0.849534	4.873430	4.988568
C	7.814223	-1.802598	1.992126	C	-6.805870	-4.069702	2.763720	H	1.687516	3.357024	4.593640
C	8.076249	-3.900266	-0.016053	C	-7.207813	-1.527855	-2.852062	H	-0.078876	3.438280	4.543755
C	4.398726	-1.688793	-2.576657	C	-8.443306	-3.554315	-1.776023	H	-1.205008	4.788894	2.511016
C	4.632424	0.627962	0.448820	C	-7.682426	-4.590007	1.819826	H	-0.315163	6.185751	3.136091
C	6.229819	0.053741	2.420973	C	-8.561782	-4.313260	-0.617335	H	-0.126908	5.694918	1.439909
C	5.051910	-2.877687	-2.880298	H	-3.387977	-0.865827	2.997165				

Compound **13-S^tBu** conformer 2 (E = -3107.4391628 a.u.)

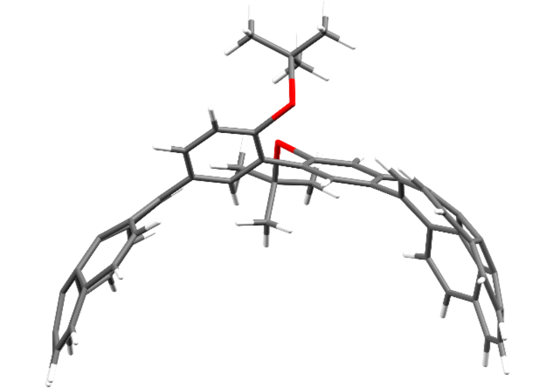

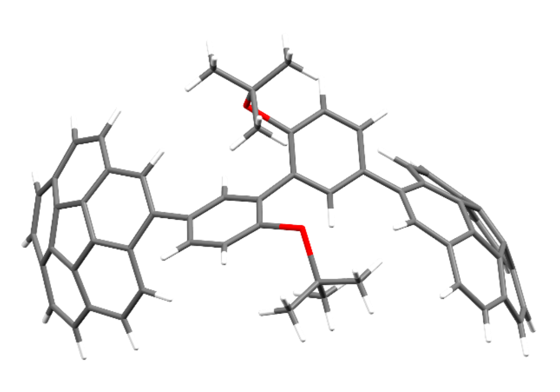

C	-2.906898	1.105144	0.875520	C	-6.773377	-4.111543	2.409458	H	4.042538	1.470365	0.999722
C	-1.802078	1.134554	0.018609	C	-7.098547	-1.236633	-3.049777	H	5.754334	0.566480	3.009932
C	-0.590258	1.743449	0.365493	C	-8.300358	-3.361311	-2.136570	H	4.175094	-3.052954	-3.969488
C	-0.466404	2.328769	1.647925	C	-7.607292	-4.596862	1.409833	H	5.980967	-5.055664	-3.281747
C	-1.588499	2.337587	2.494290	C	-8.427180	-4.193927	-1.030643	H	7.318164	-1.189368	3.627950
C	-2.779643	1.726066	2.129066	H	-3.463780	-0.828947	2.943367	H	8.473246	-3.581041	2.780417
C	-4.184462	0.495066	0.455310	H	-4.020213	1.276895	-1.525882	H	7.506379	-5.810666	-1.546565
H	-1.874207	0.650940	-0.949760	H	-5.653286	0.258824	-3.539823	H	8.539950	-5.198170	0.967674
S	1.020112	3.174356	2.143426	H	-4.577135	-2.807168	3.789342	C	1.661004	2.254211	3.656530
H	-1.531520	2.869350	3.436517	H	-6.418748	-4.801804	3.172213	C	0.780649	2.490967	4.886932
H	-3.634552	1.767801	2.798398	H	-7.247452	-1.490697	-4.097324	C	3.042181	2.886350	3.877174
C	0.492297	1.766431	-0.654112	H	-8.532345	-3.764507	-3.120272	C	1.785707	0.757113	3.374993
C	1.717451	1.157838	-0.366064	H	-7.877743	-5.650795	1.423701	C	-0.931654	4.944825	-2.039790
C	2.773858	1.117657	-1.282339	H	-8.754233	-5.219906	-1.186795	C	-2.262721	5.600549	-2.425076
C	2.571302	1.678229	-2.553297	C	5.993224	-0.847773	-1.376505	C	-0.648109	5.125614	-0.549847
C	1.357070	2.275615	-2.867277	C	6.524643	-0.539505	-0.092834	C	0.214502	5.507009	-2.880617
C	0.310609	2.346929	-1.932632	C	6.580799	-2.070998	-1.807126	H	2.518198	0.554500	2.590102
H	1.838953	0.677843	0.596208	C	7.437153	-1.567180	0.268548	H	0.824072	0.327049	3.078341
H	3.374097	1.661054	-3.285309	C	7.473538	-2.514032	-0.791894	H	2.119405	0.243094	4.285454
H	1.203822	2.708173	-3.851041	C	4.729516	-0.439403	-1.786737	H	1.290730	2.097103	5.775830
S	-1.211111	3.130058	-2.432763	C	5.809558	0.187859	0.850059	H	-0.176149	1.970570	4.801117
C	-6.174730	-0.771833	0.927318	C	5.932417	-2.952409	-2.663876	H	0.591317	3.557407	5.040476
C	-6.625423	-0.556513	-0.404976	C	7.684000	-1.916225	1.591483	H	2.959292	3.958326	4.085530
C	-6.828604	-1.934818	1.424216	C	7.761960	-3.857419	-0.582901	H	3.523094	2.408697	4.738991
C	-7.555363	-1.580584	-0.730101	C	4.070589	0.516959	-0.905532	H	3.688605	2.753786	3.004549
C	-7.682567	-2.432820	0.401231	C	4.144039	-1.246389	-2.833163	H	-2.488341	5.450650	-3.486365
C	-4.921770	-0.368401	1.372304	C	4.607113	0.803382	0.353645	H	-2.200130	6.679134	-2.238214
C	-5.833734	0.063781	-1.363028	C	6.227033	-0.012262	2.219231	H	-3.089036	5.196944	-1.830785
C	-6.259719	-2.760883	2.386067	C	4.718464	-2.440498	-3.252806	H	0.323226	6.580687	-2.678314
C	-7.745478	-2.031985	-2.031203	C	6.399733	-4.318431	-2.599803	H	0.023769	5.372884	-3.949780
C	-8.009710	-3.778935	0.288666	C	7.120460	-1.016422	2.571954	H	1.163026	5.022485	-2.631413
C	-4.422234	-1.103796	2.512148	C	8.200177	-3.252050	1.779794	H	0.287540	4.637065	-0.264684
C	-4.638525	0.684779	-0.854606	C	7.271565	-4.749957	-1.607913	H	-0.547809	6.195041	-0.323414
C	-6.187713	-0.236528	-2.731871	C	8.238179	-4.177171	0.743074	H	-1.454049	4.712955	0.062259
C	-5.060368	-2.240027	2.996258	H	3.172660	-0.972308	-3.234981				

Compound **13-S^{t}Bu** conformer 3 (E = -3107.4414468 a.u.)

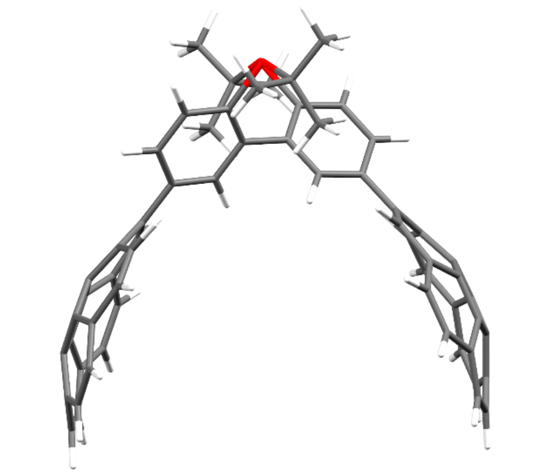

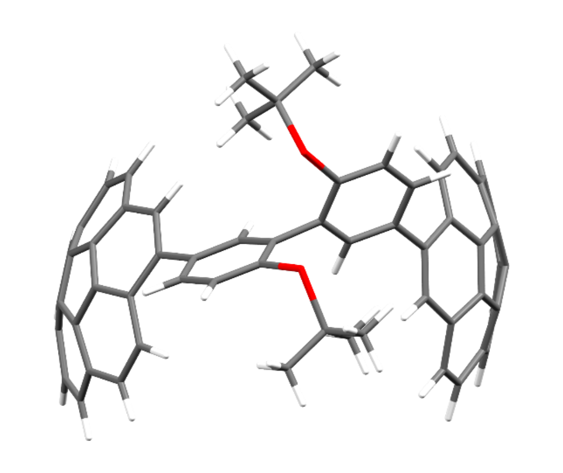

C	2.875691	1.540641	-0.263783	C	5.975932	-3.813995	-2.749137	H	-2.965500	0.506124	-2.183950
C	1.535215	1.620629	0.136529	C	4.662703	-3.242947	3.260724	H	-3.451094	-1.613455	-3.923625
C	0.701303	2.675580	-0.246187	C	5.702235	-5.203071	1.893745	H	-4.791366	-1.466175	3.980063
C	1.213784	3.673174	-1.104916	C	6.241128	-4.857374	-1.871022	H	-5.827918	-4.044200	3.802253
C	2.556973	3.602277	-1.500254	C	6.094176	-5.602969	0.621477	H	-4.335696	-3.871995	-4.086569
C	3.377392	2.556060	-1.092889	H	3.830672	0.358957	-2.709555	H	-5.447309	-5.968772	-2.623790
C	3.747375	0.439465	0.197260	H	2.966015	0.506601	2.183960	H	-6.291729	-5.869906	2.266072
H	1.125338	0.840830	0.771810	H	3.451848	-1.612737	3.923856	H	-6.133164	-6.667643	-0.399146
S	0.208846	5.025367	-1.677200	H	4.790820	-1.466597	-3.980074	C	-0.367832	4.449230	-3.369685
H	2.957516	4.392020	-2.128036	H	5.827308	-4.044627	-3.802077	C	0.813384	4.295467	-4.328130
H	4.420837	2.536925	-1.394940	H	4.336403	-3.871286	4.086965	C	-1.294377	5.580702	-3.830932
C	-0.701231	2.675554	0.246160	H	5.447687	-5.968300	2.624286	C	-1.140127	3.135478	-3.250038
C	-1.535065	1.620531	-0.136539	H	6.291318	-5.870138	-2.265717	C	0.367516	4.449275	3.369648
C	-2.875574	1.540523	0.263659	H	6.133155	-6.667499	0.399622	C	1.293997	5.580748	3.831027
C	-3.377387	2.556000	1.092631	C	-5.484359	-1.178437	0.195606	C	-0.813830	4.295492	4.327935
C	-2.557040	3.602270	1.500000	C	-5.406014	-1.590693	-1.163870	C	1.139837	3.135531	3.250081
C	-1.213822	3.673188	1.104770	C	-6.048993	-2.250845	0.942594	H	-1.983483	3.233436	-2.561778
H	-1.125098	0.840703	-0.771724	C	-5.921689	-2.911521	-1.257864	H	-0.492726	2.330734	-2.889295
H	-4.420865	2.536868	1.394567	C	-6.320672	-3.320087	0.044520	H	-1.523515	2.844320	-4.236954
H	-2.957665	4.392038	2.127698	C	-4.608987	-0.255213	0.754024	H	0.442600	4.029727	-5.327055
S	-0.208954	5.025411	1.677105	C	-4.433779	-1.106256	-2.029371	H	1.489521	3.500623	-3.999968
C	5.484449	-1.178352	-0.195771	C	-5.759797	-2.462234	2.285210	H	1.382435	5.226804	-4.406237
C	5.406314	-1.590418	1.163774	C	-5.495235	-3.813210	-2.226205	H	-0.756888	6.532766	-3.895059
C	6.048924	-2.250883	-0.942704	C	-6.316309	-4.651540	0.442937	H	-1.688307	5.342882	-4.826260
C	5.921963	-2.911252	1.257866	C	-3.747187	0.439292	-0.197365	H	-2.140090	5.702695	-3.146452
C	6.320719	-3.320009	-0.044526	C	-3.675823	0.009929	-1.526688	H	2.139810	5.702741	3.146670
C	4.609004	-0.255185	-0.754173	C	-4.156434	-1.952615	-3.167699	H	1.687782	5.342927	4.826412
C	4.434235	-1.105835	2.029368	C	-5.019825	-1.397521	2.918390	H	0.756501	6.532812	3.895074
C	5.759497	-2.462452	-2.285241	C	-5.976356	-3.813707	2.749256	H	1.523023	2.844319	4.237060
C	5.495635	-3.812794	2.226400	C	-4.662114	-3.243531	-3.260469	H	1.983336	3.233546	2.562006
C	6.316243	-4.651517	-0.442758	C	-5.701943	-5.203434	-1.893394	H	0.492518	2.330803	2.889151
C	4.469624	-0.349355	-2.189944	C	-6.241438	-4.857200	1.871241	H	-1.489938	3.500675	3.999647
C	3.676220	0.010296	1.526655	C	-6.094106	-5.603145	-0.621135	H	-0.443183	4.029704	5.326897
C	4.157050	-1.952027	3.167860	C	-4.662114	-3.243531	-3.260469	H	-1.382873	5.226837	4.406001
C	5.019451	-1.397804	-2.918448	H	-3.830959	0.359174	2.709450				

Compound **13-S^{t}Bu** conformer 4 (E = -3107.4391628 a.u.)

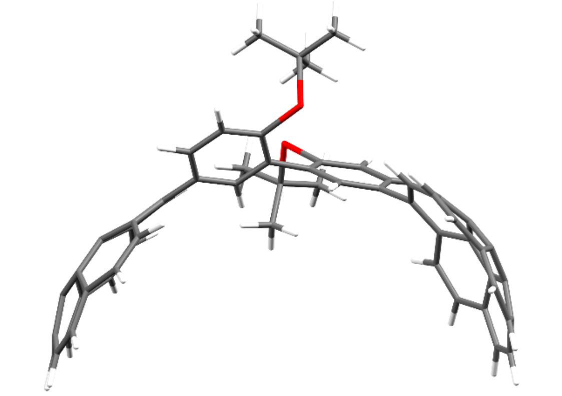

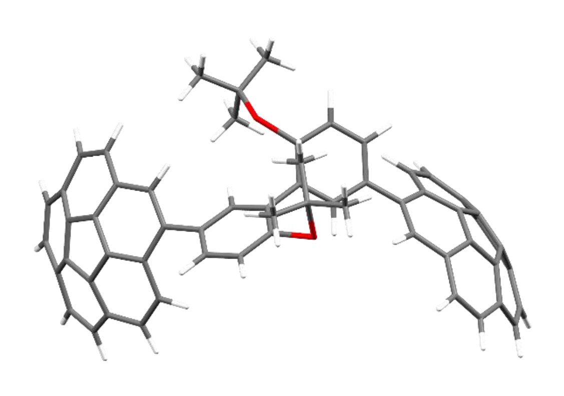

C	2.773782	1.117264	-1.282386	C	6.400415	-4.318468	-2.599407	H	-4.020364	1.276983	-1.525725
C	1.717430	1.157524	-0.366051	C	7.121427	-1.015359	2.571624	H	-5.653712	0.259334	-3.539676
C	0.492229	1.766024	-0.654100	C	8.201410	-3.250939	1.779684	H	-4.577609	-2.807536	3.789138
C	0.310466	2.346412	-1.932664	C	7.272508	-4.749641	-1.607592	H	-6.419606	-4.801807	3.171938
C	1.356846	2.274966	-2.867388	C	8.239413	-4.176241	0.743125	H	-7.248209	-1.489864	-4.097243
C	2.571096	1.677617	-2.553423	H	3.172573	-0.973093	-3.234625	H	-8.533417	-3.763558	-3.120325
C	4.070613	0.516785	-0.905614	H	4.042679	1.470472	0.999501	H	-7.878850	-5.650389	1.423439
H	1.839011	0.677678	0.596287	H	5.755146	0.567421	3.009559	H	-8.755430	-5.219105	-1.186974
S	-1.211239	3.129630	-2.432724	H	4.175281	-3.053681	-3.968920	C	-0.931637	4.944363	-2.039729
H	1.203535	2.707405	-3.851193	H	5.981675	-5.055907	-3.281147	C	-0.648022	5.125119	-0.549796
H	3.373839	1.660363	-3.285491	H	7.319403	-1.188040	3.627612	C	-2.262673	5.600198	-2.424955
C	-0.590302	1.743099	0.365533	H	8.474716	-3.579695	2.780320	C	0.214528	5.506485	-2.880594
C	-1.802160	1.134279	0.018672	H	7.507542	-5.810292	-1.546091	C	1.661047	2.253607	3.656529
C	-2.906966	1.104891	0.875616	H	8.541413	-5.197143	0.967858	C	3.042463	2.885305	3.876913
C	-2.779621	1.725725	2.129191	C	-6.175034	-0.771672	0.927400	C	0.780968	2.490779	4.887043
C	-1.588434	2.337173	2.494397	C	-6.625774	-0.556150	-0.404846	C	1.785205	0.756448	3.375073
C	-0.466375	2.328378	1.647993	C	-6.829073	-1.934594	1.424227	H	0.023719	5.372436	-3.949752
H	-1.874343	0.650692	-0.949706	C	-7.555908	-1.580032	-0.730009	H	1.163019	5.021850	-2.631477
H	-3.634486	1.767450	2.798578	C	-7.683184	-2.432355	0.401249	H	0.323365	6.580140	-2.678238
H	-1.531397	2.868854	3.436667	C	-4.921987	-0.368479	1.372353	H	-0.547641	6.194538	-0.323364
S	1.020173	3.173871	2.143461	C	-5.834043	0.064103	-1.362887	H	0.287614	4.636515	-0.264670
C	5.993433	-0.847671	-1.376665	C	-6.260273	-2.760842	2.385970	H	-1.453959	4.712507	0.062344
C	6.525016	-0.539065	-0.093140	C	-7.746185	-2.031274	-2.031142	H	-3.089002	5.196645	-1.830645
C	6.581178	-2.070856	-1.807166	C	-8.010566	-3.778403	0.288579	H	-2.199997	6.678773	-2.238071
C	7.437797	-1.566492	0.268264	C	-4.184600	0.494970	0.455399	H	-2.488336	5.450336	-3.486240
C	7.474183	-2.513529	-0.792013	C	-4.422504	-1.104071	2.512093	H	3.688734	2.752387	3.004233
C	4.729585	-0.439613	-1.786760	C	-4.638706	0.684867	-0.854477	H	3.523308	2.407648	4.738763
C	5.809943	0.188314	0.849749	C	-6.188167	-0.236007	-2.731736	H	2.959964	3.957347	4.085102
C	5.932818	-2.952551	-2.663644	C	-5.060799	-2.240242	2.996136	H	2.118910	0.242400	4.285511
C	7.684943	-1.915247	1.591218	C	-6.774162	-4.111415	2.409268	H	2.517479	0.553542	2.590052
C	7.762905	-3.856821	-0.582827	C	-7.099190	-1.235929	-3.049680	H	0.823379	0.326678	3.078630
C	4.144078	-1.246909	-2.832927	C	-8.301297	-3.360497	-2.136599	H	-0.176012	1.970668	4.801469
C	4.607281	0.803505	0.353432	C	-7.608220	-4.596501	1.409648	H	1.291047	2.096888	5.775934
C	6.227733	-0.011442	2.218880	C	-8.428190	-4.193197	-1.030744	H	0.591973	3.557294	5.040484
C	4.718661	-2.440987	-3.252450	H	-3.463975	-0.829433	2.943277				

To explore in more detail the conformational landscape of hosts **13-SH**, **13-SMe** and **13-S^{t}Bu** along the aryl torsion, a relaxed Potential Energy Scan (PES) has been carried out with previously described level of theory. It consisted of a systematic variation of the torsion angle between both aryl units by 12 º and an optimization of resulting structure. A total of 30 datapoints per compound were collected covering the full cycle of 360 º. The graphs below (Figure S 189) depict the energy changes along the molecular coordinate starting from the most stable structure (synclinal for compounds **13-SH** and **13-SMe** whereas anticlinal for **13-S^{t}Bu**). Two clear regions of minimum energy are observed in all cases as well as two maxima that correspond to the synperiplanar (higher in energy) and antiperiplanar (lower in energy) conformations.

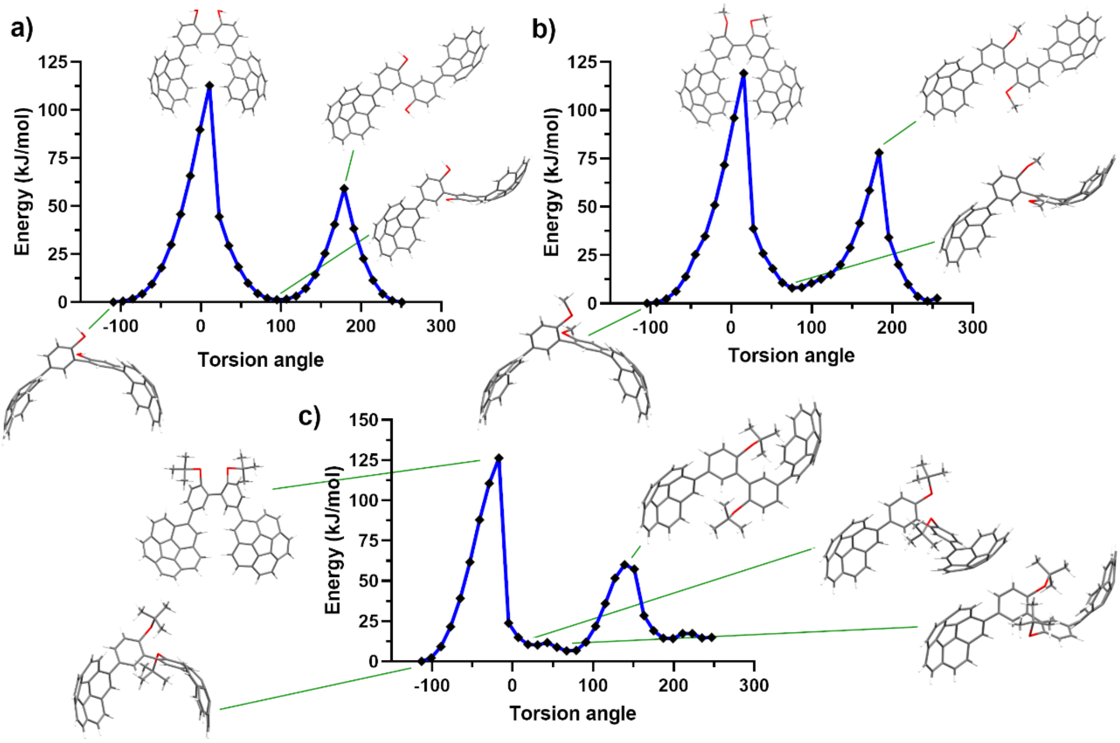

Figure S 189. PES landscape at the B97D3/def2-TZVP/PCM(toluene) level of theory belonging to corannulene-derived compounds **13-SH** (a), **13-SMe** (b) and **13-S^{t}Bu** (c) along the torsion angle between biphenyl moieties. Key structures of each species are shown.

Table S 7. Relevant stationary points observed in calculated PES landscape, their CCCC torsion angles and associated electronic energies for hosts **13-SH**, **13-SMe** and **13-StBu**.

Stationary point	Torsion angle^a	Energy (kJ/mol)^{a,b}
Minimum 1	109.9, 104.5, -112.8	-
Maximum 1	10.9, 15.5, -16.8	112.14, 119.08, 126.34
Minimum 2	94.9, 75.5, 31.2 (67.2)^c	1.34, 7.98, 10.18 (6.56)^c
Maximum 2	178.9, 183.5, 139.2	59.13, 77.94, 60.06

^{a}: for species **13-SH**, **13-SMe** and **13-S^{t}Bu**, respectively.
^{b}: referenced to the most stable structure (Minimum 1).
^{c}: value in parenthesis corresponds to the secondary minimum observed for compound **13-S^{t}Bu**.

The modeled structures of inclusion complexes with C_{60} (namely, C_{60}@**5_2-SS**, C_{60}@**13-SH**, C_{60}@**13-SS**, C_{60}@**13-SMe** and C_{60}@**13-SMe**) were obtained from the most stable conformers of parent hosts with the appropriate cavity formed between corannulene units. A molecule of the fullerene was placed and the whole system was freely relaxed a the above-described level of theory. Resulting adducts structures are shown below.

C_{60}@**5_2-SS**

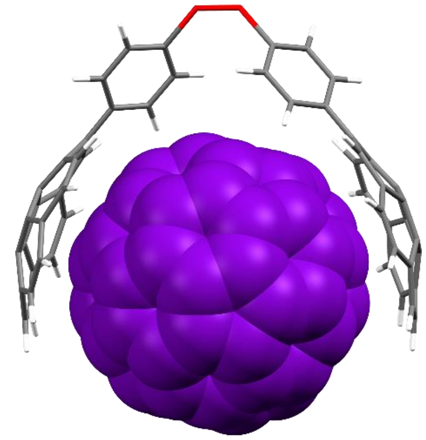

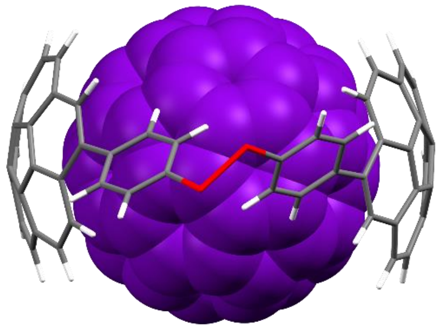

C	-3.689550	4.201139	-0.263189	H	-6.188486	-3.169617	2.890653	C	2.704319	-3.175626	-2.013061
C	-2.520948	4.316930	-1.033165	H	-6.631844	-4.120875	0.308928	C	-0.505544	-5.392095	0.268174
C	-1.618357	5.355949	-0.837162	C	6.159643	1.410893	-0.150440	C	-1.870529	-4.888826	0.312041
C	-1.872572	6.322148	0.138248	C	6.275340	0.955329	1.192234	C	-2.244171	-4.482845	-1.035754
C	-3.046448	6.244427	0.901501	C	6.555931	0.343199	-1.005609	C	-1.109593	-4.735410	-1.911944
C	-3.936069	5.194798	0.701084	C	6.733851	-0.388735	1.168317	C	-0.035500	-5.297324	-1.105992
C	-4.619676	3.068156	-0.442027	C	6.907243	-0.768148	-0.190835	C	-3.486775	-2.570771	-0.083647
H	-2.289484	3.555943	-1.770476	C	5.254882	2.391321	-0.540616	C	-3.461557	-1.163066	-0.454876
S	-0.748325	7.646959	0.553807	C	5.484760	1.456536	2.217130	C	-2.988448	-1.070341	-1.827973
H	-3.263427	7.001676	1.651289	C	6.066387	0.193698	-2.296912	C	-2.727672	-2.419228	-2.308052
H	-4.848430	5.153833	1.289073	C	6.428070	-1.302708	2.169440	C	-3.035920	-3.347438	-1.228644
C	1.528747	5.332712	0.655281	C	6.782783	-2.083873	-0.619950	C	-1.748270	0.978864	-1.233152
C	2.425646	4.309965	0.940244	C	4.583196	3.087067	0.556207	C	-0.367555	1.349118	-1.507325
C	3.655295	4.200076	0.270549	C	4.905613	2.353202	-1.943877	C	0.085019	0.573743	-2.654094
C	3.964400	5.178797	-0.690534	C	4.714350	2.620422	1.869987	C	-1.018253	-0.272495	-3.088828
C	3.079369	6.211773	-0.980393	C	5.352265	0.584804	3.362310	C	-2.151425	-0.019703	-2.210509
C	1.848311	6.286986	-0.313113	C	5.297316	1.314028	-2.779713	C	0.036530	1.519443	0.925453
H	2.145098	3.557825	1.670199	C	6.141245	-1.150447	-2.823191	C	1.111560	0.955065	1.731576
H	4.922784	5.143494	-1.200415	C	5.800548	-0.730475	3.338287	C	2.244136	0.697384	0.853055
H	3.346810	6.961468	-1.721305	C	6.519419	-2.687932	1.769671	C	1.870030	1.103296	-0.492963
S	0.739973	7.585907	-0.836367	C	6.479794	-2.235348	-2.024403	C	0.507558	1.611650	-0.449710
C	-6.122085	1.410767	0.449913	C	6.689628	-3.059624	0.441120	C	3.035670	-0.435981	1.046022
C	-6.356420	0.919203	-0.864137	H	4.219904	3.093810	-2.342862	C	2.726904	-1.362604	2.123932
C	-6.440705	0.367710	1.365511	H	4.137226	3.117446	2.645725	C	2.990356	-2.712856	1.646644
C	-6.812300	-0.422179	-0.762339	H	4.786929	0.925585	4.226942	C	3.458674	-2.618854	0.271783
C	-6.869267	-0.763094	0.616249	H	4.902895	1.291586	-3.793361	C	3.489572	-1.211428	-0.098180
C	-5.182517	2.396732	0.728866	H	5.814439	-1.338951	-3.843392	C	2.154714	-3.764853	2.030126
C	-5.657093	1.387984	-1.967922	H	5.570964	-1.372054	4.186194	C	1.021217	-3.510918	2.907614
C	-5.833365	0.247994	2.609013	H	6.341464	-3.467112	2.507545	C	-0.081375	-4.354860	2.471252
C	-6.595360	-1.364906	-1.760870	H	6.407837	-3.234633	-2.448160	C	0.370930	-5.131515	1.325528
C	-6.712758	-2.067231	1.069172	H	6.636561	-4.116020	0.187647	C	1.753203	-4.767500	1.053048
C	-4.693695	2.386834	2.089937	H	0.577843	5.378637	1.174922	C	-1.391601	-3.870372	2.512937
C	-4.861063	2.561977	-1.724165	H	-0.710899	5.405918	-1.430027	C	-1.653342	-2.520360	2.991206
C	-5.631908	0.486675	-3.097046	C	-1.639149	-2.663283	-3.150630	C	-2.725864	-1.957772	2.184057
C	-5.006547	1.369372	2.983948	C	-0.766886	-1.567766	-3.550240	C	-3.128664	-2.959486	1.208326
C	-5.872745	-1.078608	3.179616	C	0.597868	-2.072664	-3.592887	C	-2.303903	-4.141757	1.410801
C	-6.076897	-0.825929	-2.997503	C	0.568986	-3.479912	-3.221116	C	-2.697876	-0.608319	1.828463
C	-6.650881	-2.737399	-1.317071	C	-0.813266	-3.844943	-2.947925	C	-1.597864	0.237710	2.265544
C	-6.289718	-2.182024	2.445981	C	2.303989	0.357671	-1.590813	C	-1.290824	1.167825	1.186812
C	-6.707171	-3.071727	0.031776	C	3.128018	-0.823899	-1.388785	C	-2.200130	0.890435	0.084740
H	-3.965966	3.130366	2.399463	C	2.729578	-1.824264	-2.367202	C	-3.071803	-0.203589	0.481357
H	-4.359560	3.039873	-2.562090	C	1.657302	-1.261677	-3.175105	C	-0.566679	-0.300659	3.040988
H	-5.147151	0.802071	-4.018234	C	1.395251	0.088996	-2.695501	C	-0.594822	-1.709093	3.410572
H	-4.514760	1.366477	3.954174	C	3.077938	-3.581360	-0.665177	C	0.769678	-2.213987	3.366175
H	-5.457346	-1.241849	4.171506	C	2.204613	-4.676159	-0.266804	C	1.642053	-1.118142	2.969619
H	-5.926879	-1.490568	-3.845481	C	1.291938	-4.947027	-1.368145	C	0.816097	0.064976	2.768481
H	-6.534601	-3.537257	-2.045082	C	1.600591	-4.019521	-2.447102				

C_{60}@**13-SH**

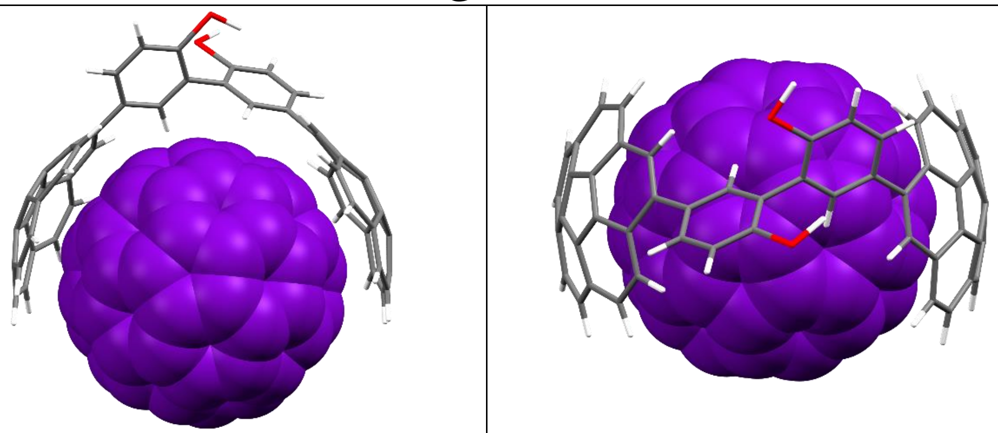

C	2.967305	4.334529	-0.446912	H	6.544929	-3.069213	-2.049437	C	-2.762917	-0.992533	-1.900793
C	1.703777	4.391581	0.159660	H	6.716963	-3.572587	0.689392	C	0.626149	1.340869	-0.042875
C	0.691517	5.229513	-0.306207	C	-5.739365	1.808241	0.384141	C	1.973789	0.794370	-0.013754
C	0.943192	6.061726	-1.415827	C	-5.829174	1.560791	-1.013746	C	2.260543	0.235389	-1.324309
C	2.211989	6.041118	-2.012942	C	-6.299501	0.685852	1.058892	C	1.091807	0.437762	-2.168255
C	3.201916	5.190037	-1.536781	C	-6.441997	0.293129	-1.203740	C	0.079330	1.121489	-1.374957
C	3.993147	3.381806	0.022112	C	-6.732295	-0.249164	0.078219	C	3.497393	-1.601308	-0.235350
H	1.480283	3.742249	0.998676	C	-4.750138	2.606653	0.946681	C	3.410510	-3.038481	-0.448473
S	-0.377662	7.083174	-2.004837	C	-4.925201	2.097111	-1.921392	C	2.866284	-3.263536	-1.779897
H	2.425441	6.691335	-2.856904	C	-5.897341	0.297808	2.331088	C	2.616481	-1.963879	-2.388211
H	4.179796	5.198536	-2.009469	C	-6.183670	-0.502602	-2.313626	C	3.006308	-0.939133	-1.431759
C	-0.646155	5.218974	0.349732	C	-6.776806	-1.617849	0.316249	C	1.602201	-5.196667	-0.902530
C	-1.637998	4.349539	-0.104302	C	-3.941632	3.361037	-0.007884	C	0.196714	-5.539266	-1.062999
C	-2.912972	4.301411	0.478600	C	-4.478656	2.327376	2.339692	C	-0.294325	-4.874991	-2.261921
C	-3.171217	5.174654	1.549035	C	-4.045538	3.098083	-1.379255	C	0.807129	-4.121987	-2.842895
C	-2.197087	6.045057	2.022236	C	-4.831122	1.386385	-3.175993	C	1.979180	-4.320315	-2.002206
C	-0.923735	6.074003	1.435567	C	-5.029907	1.235625	2.998984	C	-0.080883	-5.428944	1.389977
H	-1.393820	3.677743	-0.921082	C	-6.152409	-1.087754	2.654365	C	-1.092845	-4.747081	2.183304
H	-4.156949	5.184017	2.005208	C	-5.430477	0.146279	-3.361400	C	-2.265390	-4.550171	1.342939
H	-2.428908	6.709599	2.850055	C	-6.442656	-1.910283	-2.121830	C	-1.977705	-5.110511	0.030622
S	0.372078	7.136215	2.007322	C	-6.569898	-2.000539	1.694394	C	-0.627648	-5.653656	0.059632
C	5.747716	1.789176	-0.404542	C	-6.726922	-2.441062	-0.869339	C	-3.012440	-3.373746	1.450801
C	5.849468	1.528905	0.990254	H	-3.733261	2.915504	2.865554	C	-2.618058	-2.346002	2.405040
C	6.275567	0.660844	-1.095012	H	-3.368460	3.621794	-2.049545	C	-2.863335	-1.049508	1.793586
C	6.431762	0.244461	1.162023	H	-4.182486	1.772962	-3.958938	C	-3.410695	-1.272507	0.464793
C	6.697727	-0.292273	-0.127310	H	-4.692698	1.020468	4.010541	C	-3.501376	-2.709164	0.252263
C	4.771755	2.614937	-0.950196	H	-5.905448	-1.454877	3.648094	C	-1.977666	0.006862	2.016156
C	4.972818	2.082146	1.913804	H	-5.228435	-0.394529	-4.283336	C	-0.807392	-0.188917	2.859195
C	5.859679	0.295210	-2.369088	H	-6.306035	-2.594186	-2.956611	C	0.295343	0.567238	2.280089
C	6.166423	-0.556228	2.266587	H	-6.634949	-3.050891	1.969713	C	-0.197204	1.228923	1.079431
C	6.716872	-1.658450	-0.379470	H	-6.800057	-3.521590	-0.768389	C	-1.601731	0.883956	0.918232
C	4.483859	2.354370	-2.344397	H	0.338763	7.691681	-2.968161	C	1.591083	0.043346	2.307539
C	4.114336	3.110811	1.390331	H	-0.362830	7.753604	2.950863	C	1.839058	-1.256166	2.918336
C	4.871733	1.358759	3.162004	C	1.493575	-1.773394	-3.198057	C	2.848619	-1.940089	2.122798
C	5.008107	1.259276	-3.020607	C	0.568791	-2.874663	-3.427611	C	3.223741	-1.063766	1.022552
C	6.090869	-1.090878	-2.708818	C	-0.780891	-2.330310	-3.458169	C	2.446533	0.158918	1.136009
C	5.438870	0.101450	3.328595	C	-0.691105	-0.892373	-3.244933	C	2.765497	-3.319636	1.920188
C	6.398698	-1.967159	2.059413	C	0.714441	-0.547679	-3.085237	C	1.662356	-4.073025	2.498809
C	6.498289	-2.021370	-1.761332	C	-2.448900	-4.471845	-1.120224	C	1.268514	-5.099459	1.544531
C	6.661951	-2.491963	0.799962	C	-3.227173	-3.247363	-1.006878	C	2.127316	-4.980414	0.374946
H	3.746622	2.962323	-2.858918	C	-2.851229	-2.370812	-2.106534	C	3.053125	-3.880924	0.606433
H	3.459277	3.645432	2.073909	C	-1.838569	-3.053676	-2.899483	C	0.690441	-3.417292	3.260428
H	4.242190	1.754586	3.955848	C	-1.590182	-4.352336	-2.289993	C	0.780585	-1.980184	3.474943
H	4.659324	1.063423	-4.032331	C	-3.052764	-0.430165	-0.589050	C	-0.569705	-1.435453	3.445940
H	5.833267	-1.442988	-3.705252	C	-2.126961	0.668523	-0.357390	C	-1.493782	-2.536293	3.213811
H	5.232975	-0.442900	4.247575	C	-1.269769	0.790163	-1.528036	C	-0.714749	-3.760863	3.098815
H	6.256661	-2.656185	2.888964	C	-1.663055	-0.237664	-2.482663				

C_{60}@**13-SS**

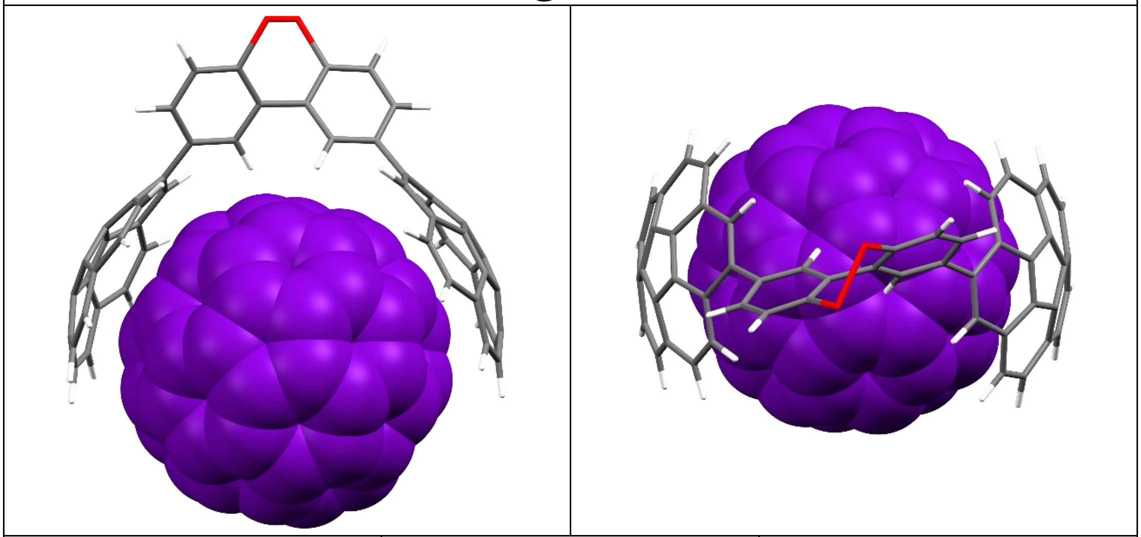

C	2.806678	4.518536	-0.290155	H	6.213786	-2.599847	2.874643	C	-1.671776	0.672808	0.762974
C	1.457478	4.562212	0.086932	H	6.648486	-2.796930	-2.071996	C	1.841737	-1.263274	2.836543
C	0.642170	5.665294	-0.203585	H	6.783668	-3.400842	0.657278	C	2.872695	-1.942919	2.066755
C	1.242636	6.784313	-0.826524	C	-5.588170	1.795133	0.371515	C	3.215638	-1.099714	0.931271
C	2.565086	6.723780	-1.273217	C	-5.685752	1.479683	-1.010984	C	2.397237	0.098369	0.996812
C	3.330969	5.588222	-1.034644	C	-6.146877	0.713202	1.106300	C	1.547716	0.000431	2.174253
C	3.718887	3.441217	0.154523	C	-6.316821	0.212518	-1.133018	C	3.475147	-3.123870	-0.457046
H	1.033588	3.725643	0.632998	C	-6.598876	-0.263536	0.177200	C	2.939169	-3.422040	-1.776939
S	0.306216	8.279335	-1.019733	C	-4.606346	2.628058	0.887900	C	2.643303	-2.157452	-2.437508
H	3.003940	7.585956	-1.767639	C	-4.780650	1.960523	-1.947855	C	2.997287	-1.080588	-1.525326
H	4.370700	5.562032	-1.348516	C	-5.733608	0.391412	2.392887	C	3.509952	-1.676859	-0.303275
C	-0.792775	5.650110	0.165760	C	-6.084419	-0.636988	-2.207124	C	0.627752	-3.182305	-3.432941
C	-1.577122	4.513255	-0.081506	C	-6.657216	-1.617259	0.483384	C	-0.739979	-2.687888	-3.482218
C	-2.923330	4.447381	0.302823	C	-3.817150	3.340981	-0.108143	C	-0.700649	-1.240092	-3.327648
C	-3.473376	5.525638	1.015605	C	-4.328549	2.432937	2.292177	C	0.691651	-0.839084	-3.186006
C	-2.739744	6.690217	1.210303	C	-3.899166	2.992453	-1.459127	C	1.513097	-2.040239	-3.250784
C	-1.422833	6.773129	0.751511	C	-4.711498	1.190821	-3.168661	C	-1.476508	-4.688272	-2.230819
H	-1.132953	3.670615	-0.602896	C	-4.864409	1.366986	3.007349	C	-2.329051	-4.789920	-1.054700
H	-4.510296	5.479804	1.336501	C	-5.996508	-0.975373	2.785915	C	-3.149572	-3.590402	-0.989024
H	-3.200562	7.555877	1.677923	C	-5.335366	-0.045592	-3.292229	C	-2.805423	-2.746539	-2.124580
S	-0.530943	8.301417	0.887331	C	-6.361837	-2.030333	-1.943995	C	-1.770542	-3.425004	-2.891787
C	5.538428	1.934534	-0.266111	C	-6.436733	-1.930629	1.877462	C	-3.440668	-3.011036	0.247803
C	5.598779	1.622222	1.119091	C	-6.635007	-2.496437	-0.663391	C	-2.929281	-3.608217	1.471500
C	6.147827	0.866908	-0.981322	H	-3.595392	3.068863	2.781251	C	-2.571515	-2.528742	2.382438
C	6.253156	0.369224	1.262190	H	-3.231078	3.496693	-2.153829	C	-2.863919	-1.266633	1.720244
C	6.590754	-0.099471	-0.037299	H	-4.067030	1.529980	-3.976404	C	-3.399986	-1.564522	0.401709
C	4.555869	2.744566	-0.815887	H	-4.525846	1.208526	4.028877	C	-1.440891	-2.646292	3.195175
C	4.655653	2.085353	2.026602	H	-5.742746	-1.296537	3.793747	C	-0.618804	-3.846757	3.127906
C	5.792209	0.538136	-2.282932	H	-5.154724	-0.628379	-4.192565	C	0.773086	-3.447549	3.272015
C	6.006879	-0.486117	2.328283	H	-6.247128	-2.755977	-2.746094	C	0.811725	-2.000206	3.426835
C	6.696375	-1.450377	-0.341516	H	-6.508929	-2.964770	2.207137	C	-0.556650	-1.504391	3.380000
C	4.334683	2.544884	-2.230052	H	-6.721168	-3.569795	-0.510657	C	1.766974	-4.098941	2.535180
C	3.770416	3.099741	1.509148	C	2.210891	0.071338	-1.463942	C	1.409493	-5.176717	1.624390
C	4.562372	1.310707	3.244171	C	1.034690	0.196851	-2.311769	C	2.262432	-5.074727	0.448646
C	4.923335	1.494992	-2.927327	C	-0.000848	0.876909	-1.544179	C	3.148224	-3.934633	0.632440
C	6.105942	-0.820461	-2.667093	C	0.538781	1.170480	-0.223126	C	2.841373	-3.330954	1.922794
C	5.207507	0.087948	3.387927	C	1.905521	0.673578	-0.176087	C	1.744624	-5.361192	-0.817906
C	6.333638	-1.871527	2.075843	C	-2.763417	-1.359746	-1.973757	C	0.352088	-5.759819	-0.960562
C	6.536322	-1.765974	-1.743585	C	-3.071158	-0.755218	-0.685602	C	-0.163053	-5.163225	-2.184720
C	6.660407	-2.330439	0.805311	C	-2.186917	0.384623	-0.502062	C	0.910402	-4.395609	-2.798420
H	3.604416	3.162895	-2.745542	C	-1.336611	0.489418	-1.678818	C	2.089482	-4.517811	-1.953285
H	3.071303	3.591178	2.182270	C	-1.693277	-0.590102	-2.589455	C	-0.466613	-5.857819	0.167860
H	3.884975	1.635402	4.030622	C	-2.016528	-0.170668	1.897422	C	0.072686	-5.560452	1.486990
H	4.626620	1.331862	-3.961164	C	-0.838894	-0.291200	2.744446	C	-0.962055	-4.882763	2.254447
H	5.899086	-1.146351	-3.684089	C	0.235976	0.479167	2.131696	C	-2.141118	-4.761259	1.409253
H	5.010774	-0.498860	4.282258	C	-0.280008	1.074033	0.905796	C	-1.835078	-5.364227	0.120254

C_{60}@**13-SMe**

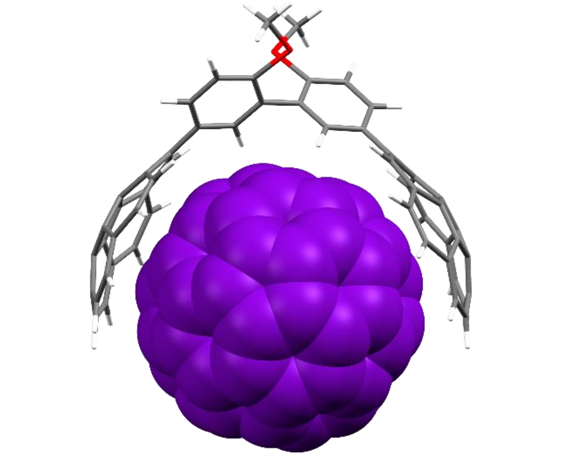

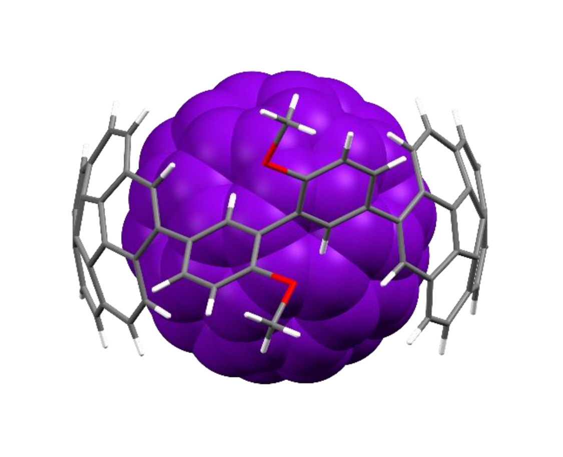

C	2.959123	4.162424	-0.446628	C	-5.742614	1.622997	0.368474	C	3.416639	-3.207910	-0.440073
C	1.694168	4.222184	0.160634	C	-5.826364	1.377993	-1.030253	C	2.875649	-3.431746	-1.773061
C	0.682726	5.055208	-0.307704	C	-6.304611	0.498737	1.038789	C	2.626281	-2.131515	-2.380335
C	0.927126	5.886766	-1.425671	C	-6.436834	0.109950	-1.225119	C	3.013103	-1.107668	-1.421678
C	2.198268	5.863622	-2.018746	C	-6.732235	-0.434995	0.054612	C	3.501965	-1.770954	-0.225003
C	3.189870	5.013744	-1.537888	C	-4.756097	2.421022	0.936576	C	0.581689	-3.042563	-3.425592
C	3.985646	3.213588	0.026688	C	-4.918191	1.916497	-1.932591	C	-0.768345	-2.499196	-3.458625
H	1.472099	3.578538	1.004548	C	-5.907585	0.108609	2.312031	C	-0.680122	-1.061417	-3.243479
S	-0.415230	6.884150	-1.989383	C	-6.173174	-0.683673	-2.335338	C	0.724783	-0.715852	-3.080090
H	2.425477	6.505518	-2.861985	C	-6.776460	-1.804225	0.289906	C	1.505115	-1.940867	-3.192594
H	4.167658	5.023430	-2.011222	C	-3.943348	3.178322	-0.012554	C	-1.578811	-4.523280	-2.294808
C	-0.653572	5.041809	0.353360	C	-4.490125	2.138820	2.330033	C	-2.440185	-4.644874	-1.127179
C	-1.641314	4.173882	-0.103917	C	-4.041585	2.916984	-1.385068	C	-3.219670	-3.421123	-1.014151
C	-2.917436	4.117465	0.479883	C	-4.818124	1.207883	-3.187987	C	-2.841795	-2.542962	-2.111819
C	-3.174817	4.984221	1.553061	C	-5.043498	1.045614	2.985180	C	-1.826788	-3.224068	-2.903284
C	-2.202276	5.856661	2.031839	C	-6.162577	-1.277818	2.631579	C	-3.497271	-2.884671	0.245024
C	-0.927092	5.894312	1.448576	C	-5.415772	-0.032304	-3.378488	C	-3.010513	-3.550380	1.443851
H	-1.396157	3.509498	-0.926539	C	-6.431549	-2.091903	-2.147170	C	-2.619124	-2.523496	2.400281
H	-4.160445	4.990690	2.009987	C	-6.574980	-2.189290	1.668176	C	-2.863975	-1.226462	1.789845
H	-2.449944	6.510293	2.860192	C	-6.720676	-2.625296	-0.896856	C	-3.408138	-1.448212	0.459564
S	0.386808	6.937083	1.996362	H	-3.746563	2.726062	2.859358	C	-1.496565	-2.713967	3.211483
C	5.745890	1.623804	-0.392486	H	-3.361538	3.441873	-2.051408	C	-0.716335	-3.937795	3.096734
C	5.844392	1.365568	1.002943	H	-4.165740	1.595981	-3.967105	C	0.688236	-3.593377	3.262039
C	6.277864	0.495494	-1.080028	H	-4.709724	0.828348	3.997459	C	0.776792	-2.156467	3.478577
C	6.428417	0.082353	1.177948	H	-5.919166	-1.646599	3.625597	C	-0.573853	-1.612728	3.447093
C	6.699141	-0.455586	-0.109943	H	-5.208869	-0.571409	-4.300378	C	1.662426	-4.247449	2.501860
C	4.769453	2.446661	-0.941863	H	-6.290449	-2.774193	-2.982557	C	1.271550	-5.272975	1.545395
C	4.963204	1.917968	1.922867	H	-6.639931	-3.240266	1.941214	C	2.132980	-5.151848	0.377955
C	5.866455	0.127311	-2.354882	H	-6.793037	-3.706098	-0.798128	C	3.057467	-4.051972	0.612957
C	6.161812	-0.717613	2.282813	C	2.266107	0.066070	-1.314422	C	2.766355	-3.492531	1.926740
C	6.721975	-1.822114	-0.360248	C	1.099168	0.268640	-2.160838	C	1.610993	-5.366951	-0.901005
C	4.485855	2.183208	-2.336386	C	0.084408	0.950711	-1.369062	C	0.206129	-5.710410	-1.065162
C	4.103917	2.943924	1.395789	C	0.628004	1.169024	-0.035511	C	-0.282616	-5.045021	-2.264405
C	4.860143	1.195679	3.171638	C	1.975878	0.623329	-0.003908	C	0.819626	-4.290485	-2.841900
C	5.014485	1.088532	-3.009937	C	-2.754851	-1.164894	-1.904086	C	1.989868	-4.488956	-1.998742
C	6.101408	-1.258777	-2.692043	C	-3.048213	-0.604311	-0.592374	C	-0.620772	-5.826802	0.055419
C	5.429630	-0.060119	3.341739	C	-2.123883	0.494893	-0.357267	C	-0.077260	-5.603296	1.387307
C	6.397540	-2.128267	2.078060	C	-1.264032	0.618586	-1.525721	C	-1.091581	-4.923199	2.179132
C	6.508030	-2.187254	-1.742237	C	-1.654234	-0.408455	-2.482449	C	-2.262349	-4.726147	1.336309
C	6.665486	-2.654240	0.820032	C	-1.979508	-0.169723	2.015632	C	-1.971194	-5.284660	0.023960
H	3.747975	2.788605	-2.852905	C	-0.811062	-0.365687	2.861158	C	0.318183	7.841188	-3.340958
H	3.444927	3.477202	2.076634	C	0.292409	0.392104	2.285570	H	1.147022	8.459806	-2.986124
H	4.226885	1.590814	3.962919	C	-0.197842	1.054995	1.084659	H	-0.483517	8.490240	-3.702337
H	4.668347	0.890200	-4.022122	C	-1.601692	0.709113	0.919769	H	0.648948	7.194671	-4.158425
H	5.847125	-1.612773	-3.688688	C	1.835972	-1.430937	2.925231	C	-0.378550	7.908258	3.319825
H	5.221964	-0.603786	4.260759	C	2.847888	-2.113137	2.131171	H	0.404502	8.585701	3.669769
H	6.254318	-2.816564	2.908044	C	3.224848	-1.235165	1.032893	H	-1.220153	8.497114	2.945041
H	6.557439	-3.235407	-2.028828	C	2.446507	-0.013218	1.146080	H	-0.698456	7.273874	4.150989
H	6.722865	-3.734914	0.711009	C	1.588483	-0.130841	2.315474				

C_{60}@**13-S^{t}Bu**

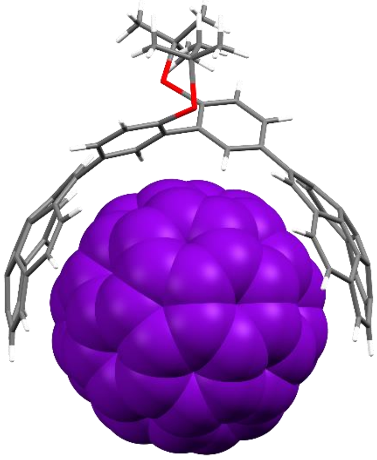 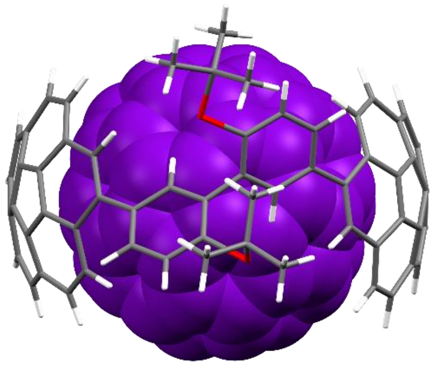

C	3.411012	-3.114461	-0.914502	C	1.671365	4.970618	-1.889492	C	-4.637648	-2.823167	0.680408
C	3.601369	-1.920471	-0.205737	C	-0.070272	5.881377	2.407623	C	-4.197631	-2.460231	2.020954
C	4.345461	-0.850464	-0.709216	C	-0.884190	6.330357	-2.221157	C	-4.960787	-1.294187	2.442757
C	4.913797	-0.962009	-1.998226	C	-1.959243	6.892186	0.431945	C	-5.872086	-0.937478	1.364960
C	4.775083	-2.176828	-2.689616	C	2.888160	3.852277	-0.017907	C	-1.883250	-3.055457	1.390833
C	4.055596	-3.239615	-2.156904	C	1.883647	4.363379	2.354674	C	-0.647483	-2.310588	1.574306
C	2.502752	-4.156110	-0.391193	C	2.635530	4.032868	-1.385426	C	-0.845609	-1.368527	2.665740
H	3.109030	-1.785065	0.749818	C	0.950678	4.944055	-3.142463	C	-2.207840	-1.530324	3.156775
S	5.668512	0.416394	-2.836347	C	0.827121	4.949514	3.040602	C	-2.848582	-2.574907	2.368997
H	5.227036	-2.269947	-3.672240	C	-1.441486	6.186366	2.747881	C	0.525001	0.288982	1.443021
H	3.979108	-4.168062	-2.714373	C	-0.269291	5.590091	-3.298337	C	0.250263	1.688416	1.149941
C	4.432595	0.387661	0.113305	C	-2.279016	6.640102	-2.012373	C	-0.716360	2.169248	2.124253
C	3.732542	1.515563	-0.304080	C	-2.342390	6.666547	1.806165	C	-1.038874	1.069960	3.022337
C	3.726661	2.718366	0.416523	C	-2.790650	6.910360	-0.749002	C	-0.272321	-0.094614	2.600847
C	4.507957	2.762292	1.581952	H	2.425893	3.568256	2.854244	C	-1.778737	3.529598	0.358122
C	5.188475	1.639529	2.038845	H	3.126553	3.362691	-2.086053	C	-3.182735	3.649730	-0.004224
C	5.147923	0.424504	1.336136	H	1.311514	4.309951	-3.949297	C	-3.980976	3.269213	1.152038
H	3.138647	1.429368	-1.205862	H	0.600920	4.589007	4.041685	C	-3.068387	2.910399	2.229300
H	4.585718	3.687518	2.145033	H	-1.812134	5.928433	3.737501	C	-1.709718	3.070242	1.736802
H	5.749958	1.722251	2.958289	H	-0.822858	5.438003	-4.222311	C	-5.608149	2.190046	-0.361886
S	5.898293	-1.092217	1.862108	H	-2.972567	6.555522	-2.846216	C	-6.203552	0.863575	-0.294949
C	0.800493	-5.834219	-0.692365	H	-3.387600	6.767091	2.090398	C	-6.132515	0.407223	1.085630
C	0.684649	-5.967918	0.720099	H	-3.866562	7.026140	-0.638417	C	-5.493210	1.451420	1.872224
C	-0.419010	-6.292523	-1.269114	C	7.507576	0.246542	-2.505935	C	-5.168740	2.553350	0.977345
C	-0.601922	-6.492682	1.015439	C	7.794116	0.440054	-1.018726	C	-5.213016	0.338949	-2.497589
C	-1.284164	-6.697560	-0.214188	C	8.123108	1.381696	-3.332400	C	-4.450061	-0.824823	-2.922787
C	1.604303	-4.873578	-1.298307	C	8.018710	-1.112200	-2.983010	C	-4.775589	-1.925978	-2.027312
C	1.377336	-5.151027	1.602199	C	7.167077	-0.755263	3.190412	C	-5.740814	-1.443071	-1.050116
C	-0.900670	-5.825441	-2.485389	C	7.849830	-2.128238	3.322858	C	-6.010875	-0.043022	-1.340867
C	-1.267790	-6.216940	2.204406	C	8.199197	0.282836	2.742851	C	-3.780590	-2.825553	-1.637670
C	-2.668867	-6.647401	-0.318153	C	6.509352	-0.396273	4.528447	C	-2.419452	-2.661516	-2.126355
C	1.203938	-4.532674	-2.648107	H	7.812564	-1.262389	-4.047222	C	-1.509864	-3.016122	-1.047845
C	2.380238	-4.320750	0.994540	H	7.558363	-1.928668	-2.418958	C	-2.305195	-3.400440	0.106168
C	0.796017	-5.047229	2.921598	H	9.104308	-1.167368	-2.828532	C	-3.709480	-3.281551	-0.256626
C	0.022797	-4.993148	-3.215381	H	8.878896	0.410562	-0.848515	C	-0.323707	-2.303512	-0.873748
C	-2.323294	-5.988276	-2.680410	H	7.338501	-0.353110	-0.421036	C	0.005834	-1.205189	-1.771459
C	-0.466649	-5.551687	3.206510	H	7.411126	1.399878	-0.661787	C	0.643995	-0.160542	-0.984947
C	-2.702405	-6.373827	2.144829	H	7.742203	2.356930	-3.011007	C	0.712998	-0.616182	0.397051
C	-3.166184	-6.377350	-1.647556	H	9.210861	1.377799	-3.193979	C	0.115120	-1.941120	0.464461
C	-3.369605	-6.581409	0.943750	H	7.912502	1.257860	-4.399785	C	0.382598	1.183060	-1.263464
H	1.776452	-3.800514	-3.206659	H	7.132029	-2.906122	3.604038	C	-0.527027	1.540513	-2.342974
H	3.013670	-3.714275	1.636697	H	8.613317	-2.069733	4.107057	C	-1.291860	2.704453	-1.917768
H	1.306307	-4.460570	3.682292	H	8.336053	-2.422808	2.387807	C	-0.851427	3.069039	-0.578373
H	-0.265250	-4.605602	-4.190080	H	7.281918	-0.334500	5.305538	C	0.181333	2.126679	-0.173527
H	-2.766182	-5.689292	-3.627666	H	5.788709	-1.166024	4.820343	C	-2.639301	2.820654	-2.267085
H	-0.902056	-5.341807	4.181129	H	5.986909	0.561513	4.502115	C	-3.279237	1.774709	-3.052778
H	-3.291588	-6.218793	3.046024	H	7.759115	1.264929	2.561828	C	-4.640154	1.612227	-2.561873
H	-4.239710	-6.369762	-1.822460	H	8.958945	0.392481	3.526977	C	-4.840920	2.556296	-1.471745
H	-4.457287	-6.581770	0.947347	H	8.696472	-0.039727	1.824559	C	-3.604676	3.302380	-1.289626
C	1.423746	5.711939	0.445361	C	-3.381214	1.855119	3.090916	C	-2.545817	0.656527	-3.461003
C	1.172947	5.860084	-0.947260	C	-4.617738	1.109266	2.906803	C	-1.140281	0.536572	-3.098990
C	0.328830	6.299462	1.144365	C	-4.346094	-0.291118	3.197807	C	-0.867319	-0.864314	-2.809037
C	-0.072126	6.524069	-1.109507	C	-2.941792	-0.411290	3.561790	C	-2.105884	-1.609773	-2.990418
C	-0.594505	6.797317	0.183612	C	-2.345212	0.915120	3.495556	C	-3.142737	-0.669305	-3.393543
C	2.176343	4.665293	0.969683	C	-5.672333	-1.881647	0.275528				

All modeled supramolecular complexes show the same parallel-like arrangement of corannulene bowls facilitated by an appropriate twist of the biphenyl tethers. The difference in binding affinities can be then ascribed to slight, yet significant, variations in conformational accessibility due to the fact that no great differences in Charge-Transfer (CT) interactions are found and Non-Covalent Interaction (NCI) Plots are located in the same spatial regions (see below).

On the one hand, the expected biphenyl torsion angle for an efficient arrangement of corannulene units should be close to 90 º to avoid repulsion of hydrogens in ortho positions. The availability of such a conformation heavily depends on the bulkiness of the sulfur substituent. Hence, hosts **13-SMe** and **13-SH** are capable to adapt their geometry that way (see above PES exploration) as they modify their torsion angle from ca. 75 º to ca. 90 º (Table S 8, entries 1, 2, 6, 7). This allows a corannulene distance (defined as the distance between cyclopentane centroids) around 12 Å, which is the typical optimum distance for a well-organized cavity.[23-26] On the other hand, compound **13-S^{t}Bu** is unable to open the angle further than 65 º from the parent host (Table S 8, entries 3, 8) owing to the great repulsion of tert-butyl groups, resulting in a slightly longer corannulene distance.

The case of hosts **13-SS** and **5$_2$-SS** show additional features. The former possesses a structural hindrance (the SS bond) that prevents it to have access to the correct torsion angle as it only reaches 42 º (Table S 8, entries 4, 9). This effect forces the molecule of fullerene to be further away from the expected cavity left by both corannulene moieties, resulting in a poorer interaction (corroborated by NEDA analysis, see below). The distance between bowls is shorter as well. Dimer **5$_2$-SS**, conversely, has virtually free access to all the minima within its conformational manifold and, therefore, is capable to arrange its structure in such a way the binding towards fullerenes is optimized. However, experimental values are similar to those for host **13-SS**. This can be ascribed to a deformation energy penalty (i. e. the cavity is vaguely preorganized) which is not shown by all the other examples of the series. Additionally, from a geometrical point of view, corannulene groups distance is significantly longer than usual (Table S 8, entry 10).

Interaction energies were calculated within the Natural Energy Decomposition Analysis (NEDA) formalism based of the Natural Bond Orbitals (NBO) method[27-28] as implemented in Gaussian with Perdew, Burke and Ernzerhof's PBE0 functional on previously optimized supramolecular adducts geometries with Pople and collaborators' split valence basis set 6-31G+(d,p)[29-32] that includes diffuse functions. The fragments of the supramolecular complexes were defined so that the first one corresponded to the host whereas the second one was C_{60} in all cases. NEDA includes the classical (electrostatic) Coulombic interaction (EL), the Charge-Transfer (CT) component accounting for the attractive interaction between filled orbitals (from the donor moiety) and unfilled orbitals (from the acceptor moiety), and steric exchange (EX) Pauli repulsion between filled orbitals. Additionally, Becke-Johnson damping empirical dispersion correction GD3BJ[18] was used to estimate the dispersion energy (DE) component. Thus, the electronic interaction energy is defined as follows (eq 7):

$$E_{int} = EL + CT + EX + DE \qquad \textbf{eq. 7}$$

All results are gathered in Table S 8:

Table S 8. Summary of computed CCCC dihedral angles. corannulene units distances, interaction energies and NEDA values pertaining to modeled hosts **13-SH**, **13-SMe**, **13-S^{t}Bu**,**13-SS** and **5$_2$-SS** end their inclusion complexes with C_{60}.

Entry	Compound	Dihedral angle	Corannulenes distance [b]	E_{int} (kcal/mol)[c]	EL (kcal/mol)[d]	CT (kcal/mol)[d]	EX (kcal/mol)	DE (kcal/mol)[d]
1	13-SH [a]	75.2	13.3	-	-	-	-	-
2	13-SMe [a]	75.5	13.4	-	-	-	-	-
3	13-S^{t}Bu [a]	65.4	11.7	-	-	-	-	-
4	13-SS [a]	33.8	11.0	-	-	-	-	-
5	5$_2$-SS [a]	-	15.6	-	-	-	-	-
6	C_{60}@13-SH	88.3	12.4	-34.33	-72.17 (33.1)	-102.55 (47.1)	183.39	-43.00 (19.7)
7	C_{60}@13-SMe	87.7	12.4	-34.47	-72.64 (33.3)	-102.31 (46.9)	183.60	-43.12 (19.8)
8	C_{60}@13-S^{t}Bu	65.3	12.5	-35.35	-72.79 (33.2)	-102.55 (46.8)	183.96	-43.97 (20.1)
9	C_{60}@13-SS	42.1	12.1	-32.83	-71.96 (33.6)	-100.75 (47.0)	181.40	-41.52 (19.4)
10	C_{60}@5$_2$-SS	-	13.0	-33.99	-69.68 (33.1)	-98.35 (46.7)	176.82	-42.78 (20.3)

[a]: most stable conformer.
[b]: distance between cyclopentane centroids.
[c]: calculated according to eq. 7.
[d]: value in parenthesis corresponds to the percentage of contribution to the attractive interaction energies (EL, CT and DE).

Table S 8 shows that all energy components are very similar across the series. Interaction energy (E_{int}) slightly increases with thiol substitution, being the most negative for complex C_{60}@**13-S^{t}Bu**. CT component is essentially equal in entries 6 - 8 so the effect is due to small variations in EL and DE components. Regarding adduct C_{60}@**13-SS**, as commented above, the fullerene molecule is placed moderately further away from the tether, resulting in a lesser contact with the biaryl tether and, therefore, E_{int} drops by 2.5 kcal/mol owing to a lower CT and DE

components, concomitant to a slight decrease in EX repulsion. A similar rationale can be applied to assembly C_{60}@**5₂-SS**, however a larger E_{int} is found (1.1 kcal/mol more negative than adduct C_{60}@**13-SS**) despite EL and CT components are notably smaller. On the other hand, Pauli repulsion is much lower (4.6 kcal/mol with respect to adduct C_{60}@**13-SS**) than all the other cases since the proximity to the tether is the least of all modeled inclusion complexes. The reason for such a low binding affinity to fullerenes cannot be found in terms of electronic interaction energies but, as commented above, deformation energy penalty due to the conformational flexibility of host **5_2-SS**.

Non-covalent interactions were obtained by the location of critical points where the reduced density gradient decreases at low electronic density values according to Yang and collaborators' scheme with the help of the NCIPlot package.[33-34] Calculations were performed with promolecular densities, and gradient isosurfaces were plotted with an isovalue of 0.3 a.u. and colored on a blue-green-red scale according to values of the sign of λ_2 (second eigenvalue of the electron-density Hessian). Red indicates repulsion, green means weak attraction, and blue represents strong attraction. Graphics were visualized in Chimera[35] with the help of Tangram NCIPlot GUI built by Insilichem Group.[36]

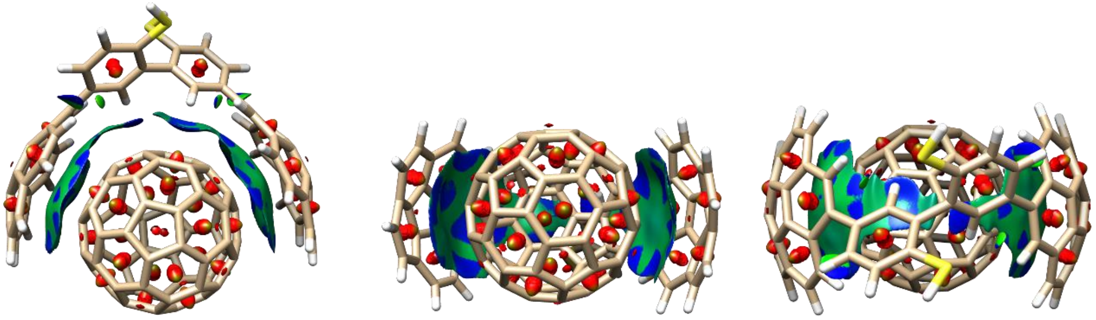

Figure S 190. Non-covalent interactions represented as gradient isosurfaces for supramolecular assembly C_{60}@**13-SH**.

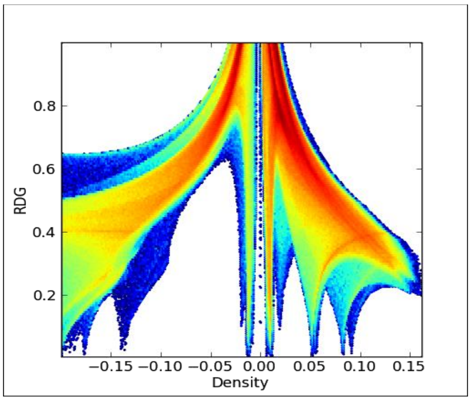

Figure S 191. Plot of the reduced density gradient versus the electron density multiplied by the sign of the second Hessian eigenvalue (λ_2) of supramolecular assembly C_{60}@**13-SH**.

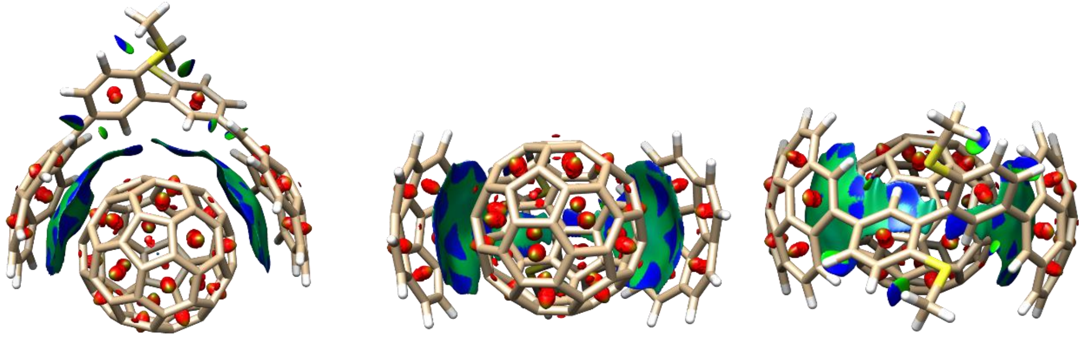

Figure S 192. Non-covalent interactions represented as gradient isosurfaces for supramolecular assembly C_{60}@**13-SMe**.

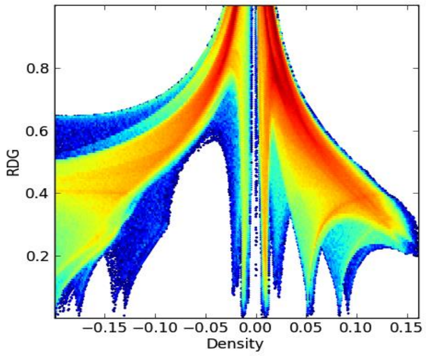

Figure S 193. Plot of the reduced density gradient versus the electron density multiplied by the sign of the second Hessian eigenvalue (λ_2) of supramolecular assembly C_{60}@**13-SMe**.

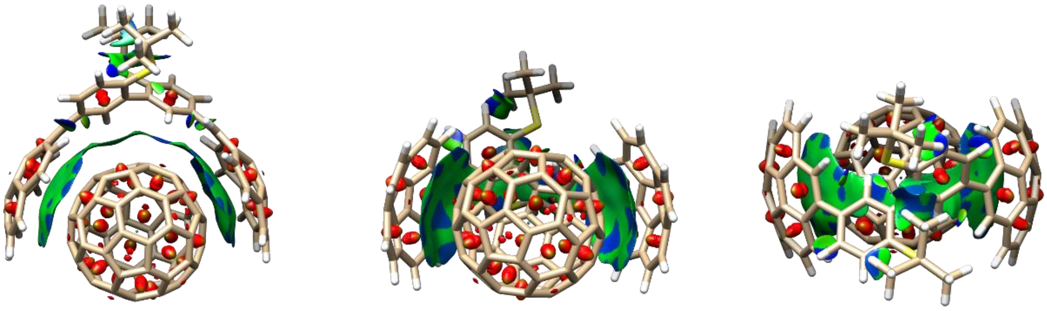

Figure S 194. Non-covalent interactions represented as gradient isosurfaces for supramolecular assembly C_{60}@**13-S^{t}Bu**.

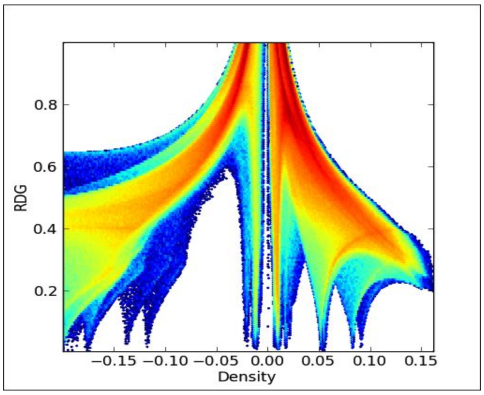

Figure S 195. Plot of the reduced density gradient versus the electron density multiplied by the sign of the second Hessian eigenvalue (λ_2) of supramolecular assembly C_{60}@**13-S^{t}Bu**.

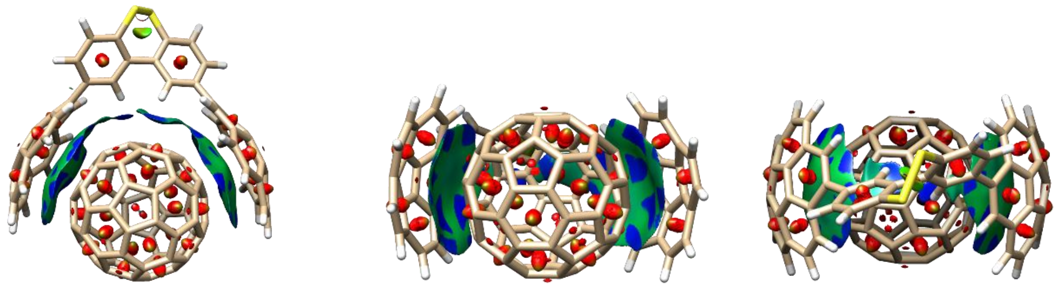

Figure S 196. Non-covalent interactions represented as gradient isosurfaces for supramolecular assembly C_{60}@**13-SS**.

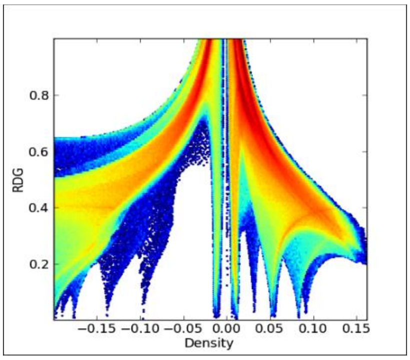

Figure S 197. Plot of the reduced density gradient versus the electron density multiplied by the sign of the second Hessian eigenvalue (λ_2) of supramolecular assembly C_{60}@**13-SS**.

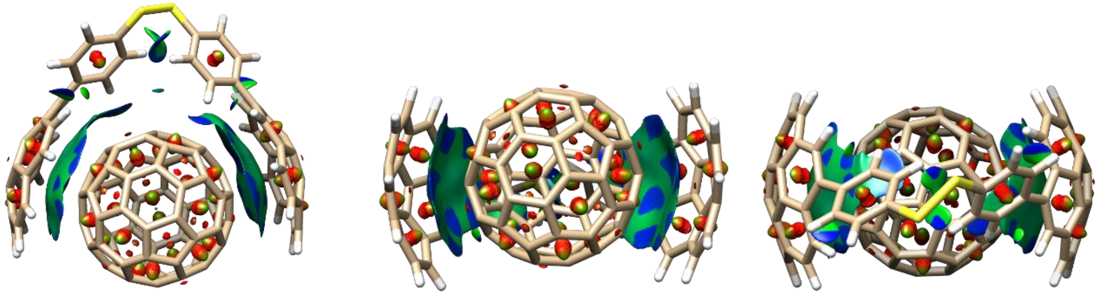

Figure S 198. Non-covalent interactions represented as gradient isosurfaces for supramolecular assembly C_{60}@**5$_2$-SS**.

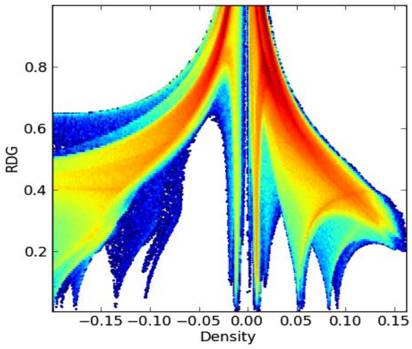

Figure S 199. Plot of the reduced density gradient versus the electron density multiplied by the sign of the second Hessian eigenvalue (λ_2) of supramolecular assembly C_{60}@**5$_2$-SS**.

Notes and References

1. Armarego, W. L. F.; Chai, C. L. L., *Purification of Laboratory Chemicals (Fifth Edition)*. Butterworth-Heinemann: Burlington, 2003.
2. Williams, D. B. G.; Lawton, M., *J. Org. Chem.* **2010,** *75*, 8351-8354.
3. Barat, V.; Budanovic, M.; Halilovic, D.; Huh, J.; Webster, R. D.; Mahadevegowda, S. H.; Stuparu, M. C., *Chem. Commun.* **2019,** *55*, 3113-3116.
4. Pinchart, A.; Dallaire, C.; Van Bierbeek, A.; Gingras, M., *Tetrahedron Lett.* **1999,** *40*, 5479-5482.
5. Sonnenschein, C.; Ender, C. P.; Wang, F.; Schollmeyer, D.; Feng, X.; Narita, A.; Müllen, K., *Chem. Eur. J.* **2020,** *26*, 8007-8011.
6. Zabula, A. V.; Spisak, S. N.; Filatov, A. S.; Rogachev, A. Y.; Petrukhina, M. A., *Acc. Chem. Res.* **2018,** *51*, 1541-1549.
7. Pastuszak, J. J.; Chimiak, A., *J. Org. Chem.* **1981,** *46*, 1868-1873.
8. Kocieński, P. J., Thioether Derivatives. In *Protecting Groups*, Thieme Verlagsgruppe: 2005; pp 366-393.
9. Buchanan, G. W.; Reyes-Zamora, C.; Clarke, D. E., *Can. J. Chem.* **1974,** *52*, 3895-3904.
10. Arakawa, Y.; Kang, S.; Tsuji, H.; Watanabe, J.; Konishi, G.-i., *RSC Adv* **2016,** *6*, 92845-92851.
11. Marom, H.; Antonov, S.; Popowski, Y.; Gozin, M., *J. Org. Chem.* **2011,** *76*, 5240-5246.
12. Wang, J.-M.; He, E.-F.; Wang, H.-L.; Hou, W.-L.; Xu, J.; Yu, L.; Zhao, L.-L.; Zhang, Z.-L.; Zhang, H.-Q., *Chin. Chem. Lett.* **2017,** *28*, 383-387.
13. Jia, X.; Liang, Z.; Chen, J.; Lv, J.; Zhang, K.; Gao, M.; Zong, L.; Xie, C., *Org. Lett.* **2019,** *21*, 2147-2150.
14. Borsari, M.; Cannio, M.; Gavioli, G., *Electroanalysis* **2003,** *15*, 1192-1197.
15. Due to the fact that there is an increasing amount of solvents distinct from Toluene-d8 throughout the switching process, chemical shifts changes are substantially different in those cases were they should be equal. Given that situation, we considered to provide the spectral array in Figures S 159-S 162 in pure Toluene-d8 to clearly show the spectral variations exclusively pertaining to conformational changes and supramolecular interactions with fullerenes through this back and forth redox process.
16. Grimme, S., *J. Comput. Chem.* **2006,** *27*, 1787-1799.
17. Grimme, S.; Antony, J.; Ehrlich, S.; Krieg, H., *J. Chem. Phys.* **2010,** *132*, 154104.
18. Grimme, S.; Ehrlich, S.; Goerigk, L., *J. Comput. Chem.* **2011,** *32*, 1456-1465.
19. Weigend, F.; Ahlrichs, R., *Phys. Chem. Chem. Phys.* **2005,** *7*, 3297-3305.
20. Weigend, F., *Phys. Chem. Chem. Phys.* **2006,** *8*, 1057-1065.
21. Tomasi, J.; Mennucci, B.; Cammi, R., *Chem. Rev.* **2005,** *105*, 2999-3094.
22. Gaussian 16, R. C., M. J. Frisch, G. W. Trucks, H. B. Schlegel, G. E. Scuseria, M. A. Robb, J. R. Cheeseman, G. Scalmani, V. Barone, G. A. Petersson, H. Nakatsuji, X. Li, M. Caricato, A. V. Marenich, J. Bloino, B. G. Janesko, R. Gomperts, B. Mennucci, H. P. Hratchian, J. V. Ortiz, A. F. Izmaylov, J. L. Sonnenberg, D. Williams-Young, F. Ding, F. Lipparini, F. Egidi, J. Goings, B. Peng, A. Petrone, T. Henderson, D. Ranasinghe, V. G. Zakrzewski, J. Gao, N. Rega, G. Zheng, W. Liang, M. Hada, M. Ehara, K. Toyota, R. Fukuda, J. Hasegawa, M. Ishida, T. Nakajima, Y. Honda, O. Kitao, H. Nakai, T. Vreven, K. Throssell, J. A. Montgomery, Jr., J. E. Peralta, F. Ogliaro, M. J. Bearpark, J. J. Heyd, E. N. Brothers, K. N. Kudin, V. N. Staroverov, T. A. Keith, R. Kobayashi, J. Normand, K. Raghavachari, A. P. Rendell, J. C. Burant, S. S. Iyengar, J. Tomasi, M. Cossi, J. M. Millam, M. Klene, C. Adamo, R. Cammi, J. W. Ochterski, R. L. Martin, K. Morokuma, O. Farkas, J. B. Foresman, and D. J. Fox, Gaussian, Inc., Wallingford CT, 2016.
23. Sygula, A.; Fronczek, F. R.; Sygula, R.; Rabideau, P. W.; Olmstead, M. M., *J. Am. Chem. Soc.* **2007,** *129*, 3842-3843.
24. Álvarez, C. M.; García-Escudero, L. A.; García-Rodríguez, R.; Martín-Álvarez, J. M.; Miguel, D.; Rayón, V. M., *Dalton Trans.* **2014,** *43*, 15693-15696.
25. Yanney, M.; Fronczek, F. R.; Sygula, A., *Angew. Chem. Int. Ed.* **2015,** *54*, 11153-11156.
26. Barbero, H.; Ferrero, S.; Álvarez-Miguel, L.; Gómez-Iglesias, P.; Miguel, D.; Álvarez, C. M., *Chem. Commun.* **2016,** *52*, 12964-12967.
27. *NBO 7.0.*, Glendening, E. D.; J, K. B.; Reed, A. E.; Carpenter, J. E.; Bohmann, J. A.; Morales, C. M.; Karafiloglou, P.; Landis, C. R.; Weinhold, F. Theoretical Chemistry Institut: University of Wisconsin, Madison, (2018).
28. Glendening, E. D.; Landis, C. R.; Weinhold, F., *J. Comput. Chem.* **2019,** *40*, 2234-2241.
29. Ditchfield, R.; Hehre, W. J.; Pople, J. A., *J. Chem. Phys.* **1971,** *54*, 724-728.
30. Hehre, W. J.; Ditchfield, R.; Pople, J. A., *J. Chem. Phys.* **1972,** *56*, 2257-2261.
31. Francl, M. M.; Pietro, W. J.; Hehre, W. J.; Binkley, J. S.; Gordon, M. S.; DeFrees, D. J.; Pople, J. A., *J. Chem. Phys.* **1982,** *77*, 3654-3665.
32. Frisch, M. J.; Pople, J. A.; Binkley, J. S., *J. Chem. Phys.* **1984,** *80*, 3265-3269.
33. Johnson, E. R.; Keinan, S.; Mori-Sánchez, P.; Contreras-García, J.; Cohen, A. J.; Yang, W., *J. Am. Chem. Soc.* **2010,** *132*, 6498-6506.
34. Contreras-García, J.; Johnson, E. R.; Keinan, S.; Chaudret, R.; Piquemal, J.-P.; Beratan, D. N.; Yang, W., *J. Chem. Theory Comput.* **2011,** *7*, 625-632.
35. Pettersen, E. F.; Goddard, T. D.; Huang, C. C.; Couch, G. S.; Greenblatt, D. M.; Meng, E. C.; Ferrin, T. E., *J. Comput. Chem.* **2004,** *25*, 1605-1612.
36. Rodríguez-Guerra Pedregal, J. M., Jean-Didier, dir.; Cairó Badillo, Jordi Joan, dir. Development and application of a computational platform for complex molecular design. 1 recurs en línia (167 pàgines). ISBN 9788449082382. <https://ddd.uab.cat/record/201498> [Consulta: 10 maig 2022]. .
